# Modified Guilu Erxian Glue regulates Treg immune function to suppress bone marrow failure in aplastic anemia mice

**DOI:** 10.1186/s13020-025-01266-z

**Published:** 2025-11-20

**Authors:** Wei Liu, Pingxin Zhang, Jingmin Niu, Yingkai Zhang, Song Sun, Jinghao Sang, Weihua Gao, Boyang Meng, Limin Chai

**Affiliations:** 1 Key Laboratory of Chinese Internal Medicine of Ministry of Education and Dongzhimen Hospital, Beijing University of Chinese Medicine, Beijing, 100700 China; 2 The Third Affiliated Hospital of Beijing University of Chinese Medicine, Beijing, 100029 China

**Keywords:** Modified Guilu Erxian Glue, Regulatory T cells, Aplastic anemia, MiR-17-5p/Eos signaling pathway, IL-2/STAT5 signaling pathway, Apoptosis

## Abstract

**Background:**

Aplastic anemia (AA) is an autoimmune disorder characterized by impaired immunosuppressive function and abnormal differentiation of regulatory T (Treg) cells. Immunosuppressive treatment (IST) is a primary treatment for AA. Our previous studies have suggested that Modified Guilu Erxian Glue (MGEG) could improve hematopoietic function through immune modulation. These results indicated that it should serve as an adjunct therapy in boosting the efficacy of IST for AA treatment. Nevertheless, the regulatory mechanisms of MGEG on the Treg cells were unclear. In this study, we aimed to investigate the mechanisms of the combination therapy of IST and MGEG on the function and differentiation of Treg cells, contributing to alleviate the hematopoietic dysfunction in immune-mediated AA mice.

**Methods:**

An AA mouse model was established using 3.5 Gy ^60^Coγ irradiation followed by allogeneic lymphocyte infusion via the tail vein. The combination of IST + MGEG was used as therapeutic treatment. The combination of IST + EP was used as positive control. The chemical composition of MGEG was analyzed by HPLC-ESI/MS. Hematological parameters, histopathological staining, and flow cytometry were used to evaluate bone marrow hematopoiesis. The differentiation of CD4^+^ T and Treg cell subsets were analysed by CyTOF-2 mass cytometry. Inflammatory factor levels and Fas/FasL pathway protein expression were measured by ELISA and Western blot. Flow cytometry was also used to examine proliferation and differentiation of naïve T, effector T, and Treg cells. The regulatory effects of IST combined with MGEG on the IL-2/STAT5 and miR-17-5p/Eos signaling pathways were verified by qPCR and Western blot.

**Results:**

HPLC-ESI/MS identified 30 compounds from the aqueous extract of MGEG, including amentoflavone, berberine, and ononin. The combined treatment of IST + MGEG improved the hematopoietic function of AA mice, as indicated by restored blood cell counts and reduced bone marrow adiposity. It also rebalanced the Th1/Th2 and Th17/Treg ratios, increased the proportion of Treg B cells, and ameliorated bone marrow inflammatory status. Furthermore, the combination treatment could inhibit Treg cell apoptosis through downregulating the expression of Fas and levels of Cleaved-Caspase-3/8 while upregulating p-Bcl-2. It also enhanced p-STAT5 and Foxp3 protein levels, contributed to promoting naïve T cell differentiation into Treg cells. Additionally, IST combined with MGEG reduced miR-17-5p and HIF-1α expression in CD4^+^ T cells, accompanied by the increase in the protein expression of Eos.

**Conclusions:**

Compared with the IST + EP or IST alone, the combination treatment of IST + MGEG further improved hematopoietic function in AA mice. These effects should involve regulating the differentiation of Treg cells through intervening in the activation of IL-2/STAT5 signaling pathway, improving the immune regulatory function of Treg cells by intervening in the miR-17-5p/Eos signaling pathway, and inhibiting Fas/FasL mediated apoptosis.

**Graphical Abstract:**

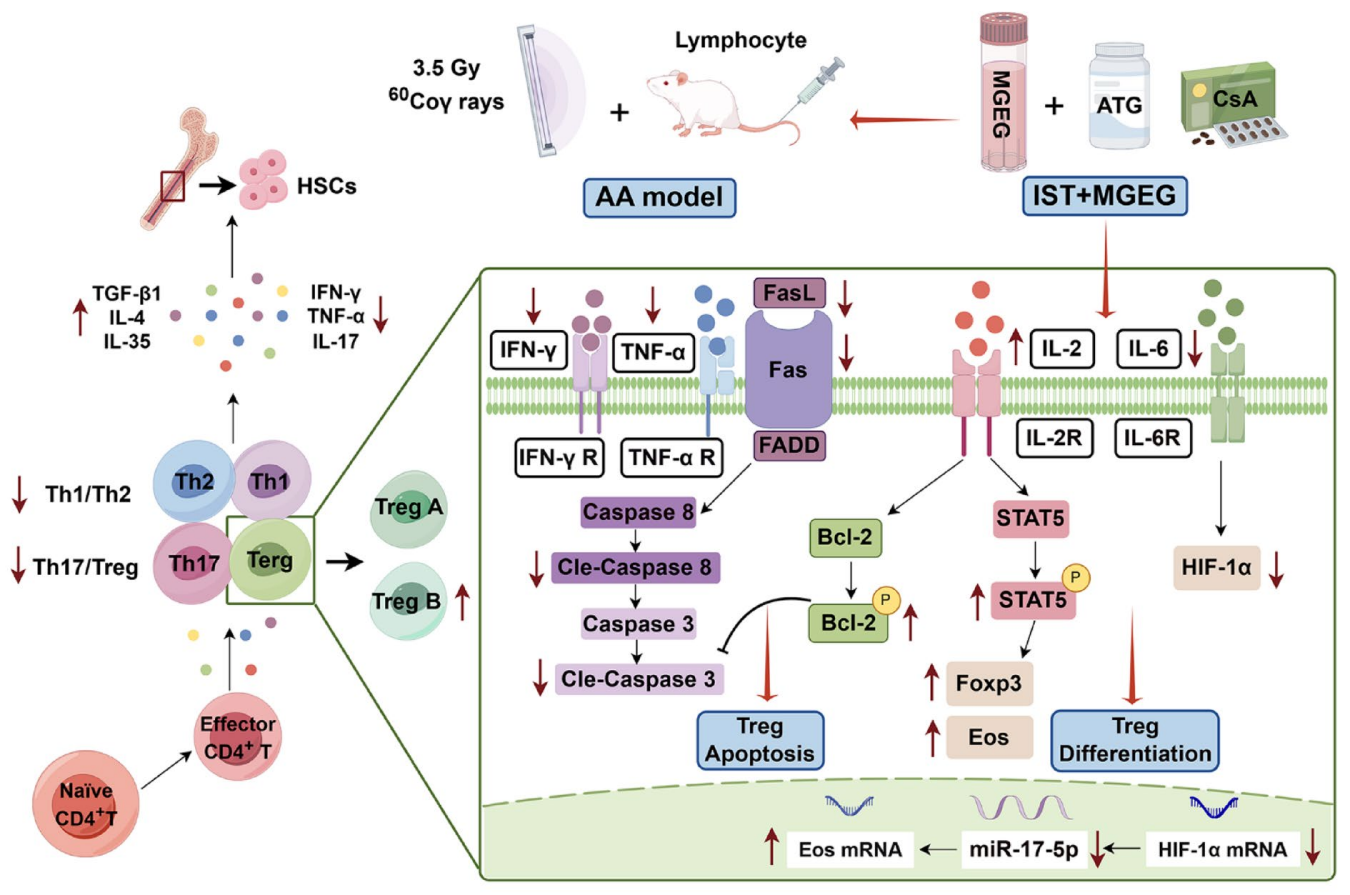

**Supplementary Information:**

The online version contains supplementary material available at 10.1186/s13020-025-01266-z.

## Introduction

Aplastic anemia (AA) is a bone marrow failure disorder marked by a quantitative and qualitative deficiency of hematopoietic stem and progenitor cells (HSPCs) [1]. Aberrant activation of T lymphocytes plays a central role in mediating immune attack against HSPCs, contributing to pancytopenia. Allogeneic hematopoietic stem cell transplantation (HSCT) is the first-line treatment for AA. However, due to limitations such as patient age and donor availability, immunosuppressive therapy (IST), consisting of anti-thymocyte globulin (ATG) and cyclosporine A (CsA), is the standard clinical treatment approach for patients who are unsuitable transplantation [2]. However, a substantial proportion of patients exhibit poor response to IST, and approximately 35% experience relapse, with 15–20% progressing to secondary myeloid malignancies [3–5]. Eltrombopag (EP), thrombopoietin receptor agonist, can stimulate the proliferation of residual HSCs. Its combination with IST can improve hematopoietic recovery in AA, but this regimen is still associated with significant risks of clonal evolution and disease relapse. Meanwhile, EP may impair patients’ liver and gallbladder function [6–8].

Deficiency in regulatory T (Treg) cells is recognized as a key factor in AA pathogenesis, contributing to loss of immune tolerance [9]. Patients often display impaired Treg function and numerical deficits, leading to overactivation of effector T-cell and disrupting the balance of multiple T cell subpopulations [4]. Hyperactivated CD4⁺ T cells secrete excessive inflammatory cytokines such as IFN-γ and TNF-α, which can activate the Fas (Apo-1, CD95)/Fas ligand (FasL) apoptotic pathway, thereby damaging HSCs [10, 11]. Notably, Treg cells, particularly Treg B, are highly susceptible to Fas-mediated apoptosis, which may underlie the limited efficacy of IST in some cases [12]. Transcription factor forkhead box protein 3 (Foxp3) is essential for Treg differentiation and function. Inter-leukin-2 (IL-2)/transcription factor signal transducer and activator of transcription 5 (STAT5) signaling pathway promotes Treg development by inducing Foxp3 expression [12–14]. However, Foxp3 does not work alone, it needs to collaborate with other proteins and epigenetic modifications. Among them, microRNA-17-5p (miR-17-5p) post-transcriptionally targets Eos (Ikzf4), a Foxp3 cofactor critical for Treg stability and function [15]. Meanwhile, IL-6 can enhance miR-17-5p expression, which inversely correlates with Eos levels, suggesting a regulatory pathway that may influence Treg function and development in inflammatory settings [16]. Based on traditional Chinese medicine principles, we developed Modified Guilu Erxian Glue (MGEG) by integrating principles from Guilu Erxian Glue (GEG) and Danggui Buxue Tang (DBT). Our previous studies have shown that DBT can rebalance immunity in AA mice by modulating the JAK/STAT pathway and transcription factors involved in Th and Treg cells differentiation [17, 18]. We further reported that MGEG attenuated Fas-mediated apoptosis of HSCs by regulating the SLAM-SAP signaling pathway and T-bet expression in T cells [19]. Based on MGEG’s multi-target immunomodulatory properties, we attempt to determine whether it enhances the efficacy of IST in AA by regulating the immunosuppressive function of Treg cells. Specifically, we investigated the dual mechanisms of promoting Treg function and development through the IL-2/STAT5 and miR-17-5p/Eos signaling pathways to inhibit effector T cell activation and Treg apoptosis through the Fas/FasL pathway in an AA mouse model.

## Materials and methods

### Preparation of MGEG decoction

MGEG comprises seven herbs, including 10 g *Tortoise-shell Glue* (guijiajiao, 20,231,130), 10 g *Deer-Horn Glue* (lujiaojiao, 21,010,203), 10 g *Ginseng Radix et Rhizoma* (renshen, 2,307,080), 20 g *Lycium barbarum L* (gouqi, 20,231,130), 30 g *Astragalus membranaceus* (haungqi, 23,103,001), 6 g *Angelica Sinensis Radix* (danggui, 23,102,801), and 6 g *Coptidis Rhizoma* (huanglian, 2,310,015). All herbs were supplied by Dongzhimen Hospital Pharmacy Department, Beijing University of Chinese Medicine (Beijing, China). Apart from the *tortoise-shell glue and deer-horn glue*, the herbs were soaked in 8 times their volume of deionized water for 30 min. Following this initial soak, the herbs were brought to a boil under high heat, then simmered gently for another 30 min. The first extract was then filtered and collected. Next, 6 times the volume of deionized water was added to the remaining herbs, and they were decocted for another 20 min, during which the *tortoise-shell glue and deer-horn glue* were dissolved. The two extracts were combined thoroughly and concentrated by heating, resulting in an MGEG decoction with a concentration of 2.36 g/mL.

### HPLC-ESI/MS analysis of MGEG

MGEG extracts were lyophilized into powder, then dissolved in methanol/water (1:1, v/v), followed by centrifugation at 13,000 rpm for 15 min and filtration. HPLC-ESI/MS analysis was performed using a Waters ACQUITY HSS T3 column. The mobile phase consisted of solvent A (0.05% formic acid in ultrapure water) and solvent B (0.05% formic acid in acetonitrile). We employed a gradient elution system at a flow rate of 0.3 mL/min, maintaining the column temperature at 40 °C. A SCIEX X500R Q-TOF mass spectrometer was utilized for electrospray ionization in both positive and negative ion modes, with the positive ion mode set at 5500. The Turbo V nebulizer operates at a sizzling 500 °C, with atomizing gas and assist gas pressures set at 50 psi and 60 psi, respectively. The impact gas pressure is kept moderate, while the sheath gas pressure stands at a steady 30 psi. The data was meticulously analyzed using the SCIEX Peak View Software^™^.

### AA model establishment and grouping

Female BALB/c mice and male DBA/2 mice (8 weeks, 18 ± 2 g) were procured from Beijing Weitonglihua Laboratory Animal Co., Ltd. (SCXK [2021]-0006). All animals were maintained under SPF conditions with free access to food and water. All experimental procedures involving animals were reviewed and approved by the Animal Ethics Committee of Dongzhimen Hospital, Beijing University of Chinese Medicine (Approval No. 22–24) and performed in compliance with the relevant animal welfare guidelines. Single-cell suspensions were prepared from the thymus and lymph nodes of DBA/2 mice. BALB/c mice were irradiated with a 3.5 Gy sublethal dose of ^60^Coγ rays, and then received an infusion of allogeneic lymphocytes via the tail vein within 4 h.

The mice were randomized into five groups (n = 10 per group): control, model, IST, IST + EP, and IST + MGEG. Drug doses for mice were converted from clinical doses for AA patients based on body surface area (BSA), with a conversion factor of 9.1. The IST group received ATG (6 mg/kg/day) by tail vein injection for 5 days, combined with CsA (1.5 mg/kg/day) administered orally for 28 days. Based on the IST treatment, AA mice in the IST + EP group were treated with EP (22.5 mg/day), and those in the IST + MGEG group were given MGEG (11.96 g/kg/day). The control and model groups were given an equal volume of saline. The dose of MGEG used was selected based on our previous dose-finding experiments [20].

### Hematological parameters analysis

After drug intervention treatment on days 0, 7, 14, 21, and 28, mice tail vein blood was collected, and peripheral blood was analyzed using the Nihon Kohden DXH 900 blood analyzer. The levels of white blood cells (WBC), red blood cells (RBC), platelet (PLT)and hemoglobin (HGB) were quantified.

### Hematoxylin and eosin (HE) and Wright-Giemsa staining

After anesthesia, mouse femurs were fixed in 4% PFA, decalcified in 10% EDTA, and finally embedded in paraffin. Standard 5 μm sections were dewaxed, rehydrated, and H&E stained. Bone marrow cells from right femurs were collected and smeared. Smears were stained with Wright-Giemsa (Solarbio, G1020, China). After rinsing and air-drying, the smears were observed and photographed under a microscope (Leica, DM RAS2, Germany).

### Flow cytometry analysis

To assess the proportion of HSCs and the apoptosis rate of bone marrow cells (BMCs) in mouse bone marrow by flow cytometry, femoral BMCs were collected and prepared into single-cell suspensions. We then took 5 × 10^5 washed cells and mixed them with 20 μL of Mouse Hematopoietic Lineage Antibody Cocktail (eFlour 450, 2,324,728, Invitrogen) to eliminate T, B, and NK cells. Next, we added 0.3 μL PE anti-mouse CD117 antibody and 1.25 μL APC anti-mouse CD45 antibody (BD Bioscience, 561,075, 561,018, USA), and incubated the mixture for 30 min. After incubation, the cells were centrifuged and washed, then analyzed using a flow cytometer. Additionally, the apoptosis rate of BMCs in mice was detected using an Annexin V-FITC kit (BOSTER, MK1028, China).

Half of the spleen tissues were homogenized and filtered to create a single-cell suspension. Subsequently, 5 µL each of anti-mouse CD4 FITC, anti-mouse CD62L APC, and anti-mouse CD44 PE antibodies (BioLegend, 100,406, 104,411, 103,024, USA) were added for labeling, to determine the proportions of naïve T and effector T cells. Furthermore, the single-cell suspensions were incubated with 5 µL each of anti-mouse CD4 FITC and anti-mouse CD25 PE antibodies (BioLegend, 100,406, 102,008, USA). After CD4/CD25 staining, cells underwent blocking, fixation, and permeabilization using a dedicated kit. (Beckman, A07803, USA). Subsequently, 5 µL of anti-mouse Foxp3-APC antibody (Invitrogen, 17–5773-82, USA) was added for intracellular staining. Samples were analyzed with a FACS Calibur flow cytometer, and data processed by the Cell Quest software version 1.5 (Beckman Coulter, B73416, USA).

### CyTOF-2 mass spectrometry flow cytometry analysis

Mononuclear cells from peripheral blood were separated using a commercially available kit (Haoyang Biological, LDS1090, China). The isolated cells fixed with 1.6% paraformaldehyde were stained with 2 μM cisplatin (Invitrogen, 00-4975-93, USA) for four hours. Surface antibodies were subsequently conjugated with metal tags using the maxPar × 8 Polymer Kit. Cells were first labeled with pre-mixed solution of metal-conjugated surface antibodies, followed by a 30-min incubation on ice. Afterward, the cells underwent fixation and permeabilization using 500 μM Fix Buffer and Perm Buffer. An intracellular antibody cocktail was then introduced for cell labeling, with a subsequent 30-min incubation at room temperature. Finally, the cells were resuspended in 10% EQ beads and thoroughly mixed before analysis.

Utilizing Cytobank for FCS data analysis, we subjected the data to clustering and dimensionality reduction via X-shift and t-SNE algorithms, complemented by viSNE for visualization. The analysis of cell marker expression discern the proliferation and differentiation of CD4^+^ T cell subsets and Treg cell subsets. The CD4^+^ T cell subsets encompassed Th1 cells (CD4^+^ IFN-γ^+^ T-bet^+^), Th2 cells (CD4^+^ IL-4^+^ GATA3^+^), Th17 cells (CD4^+^ IL-17^+^ RORγt^+^) and Treg cells (CD4^+^ CD25^+^ Foxp3^+^). Within the Treg cell subset, we also identified Treg A cells (CD4^+^ CD25^+^ Foxp3^+^ CD95^−^CD127^−^ CCR4^−^) and Treg B cells (CD4^+^ CD25^+^ Foxp3^+^ CD95^+^ CD127^−^ CCR4^−^).

### Enzyme-linked immunosorbent assay (ELISA)

The serum concentrations of FasL, IFN-γ, TNF-α, IL-17, IL-4, IL-35, IL-2, IL-6, and TGF-β1 (Elabscience, E-EL-M0028c, E-EL-M0048c, E-EL-M3063, E-EL-M0006, E-EL-M0043, E-EL-M0733, E-EL-M0042c, E-EL-M0044; Invitrogen, BMS608-4, USA) were quantified using commercially available ELISA kits. All assays were performed according to the manufacturers’ instructions.

### Quantitative real-time PCR (qPCR)

Half of the spleen tissue was harvested and processed into a single-cell suspension. Subsequently, CD4^+^ T lymphocytes underwent purification using magnetic-activated cell sorting (MACS), strictly adhering to the manufacturer’s instructions (Miltenyi, 130–117-043, Germany). The purity of the sorted CD4^+^T cells was validated using flow cytometry. Total RNA and miRNA isolation from CD4^+^ T cells employed the MagZol Reagent Kit (Magen, R4310-02, China), followed by concentration assessment via NanoDrop 2000 (Thermo Scientific, USA). MiRNA was converted to cDNA using a miRNA 1 st Strand cDNA Synthesis Kit (Vazyme, MR201-02, China) via the tailing method. The RNA samples were first cleaned of genomic DNA, and then they were reverse transcribed into cDNA using the HiScript^®^IIIAll-in-one RT SuperMix Perfect for qPCR (Vazyme, R333-01, China). The qPCR was performed using the Taq Pro Universal SYBR qPCR Master Mix (Vazyme, Q712-02, China). The results were assessed via 2^−ΔΔCT^. *MiR-17-5p* levels were normalized to *U6*, while *HIF-1α* and *Eos* mRNA levels were normalized to *GAPDH*. The primer sequences for *miR-17-5p*, *HIF-1α* mRNA, and *Eos* mRNA were detailed in Table 1.

**Table 1:** qPCR primer sequences

Name	Primer sequences
*U6*	F:5′CTCGCTTCGGCAGCACA3′ R:5′AACGCTTCACGAATTTGCGT3′
*miR-17-5p*	F: 5′CCGACTGCAGTGAGGGCACTTGTAG3′
*GAPDH*	F:5′AGGTCGGTGTGAACGGATTTG3′ R:5′GGGGTCGTTGATGGCAACA3′
*HIF-1α*	F:5′GGGCACCGATTCGCCAT 3′ R:5′TCGACGTTCAGAACTCATCCT′
*Eos*	F: 5′CTTTATTTTGTGAAAGCCTTTGCG3′ R:5′GAGTCCCCGCTACCTACTGG3′

### Western blot

Treg cells underwent purification using MACS (Miltenyi, 130-091-041, Germany). Total proteins were then extracted from the sorted Treg cells by lysing with RIPA buffer containing protease and phosphatase inhibitors (Epizyme, GRF103, China). Protein concentrations were quantified by BCA reagent protocol (Epizyme, ZJ102, China). Samples with equal protein content were loaded onto a sodium dodecyl-sulfate polyacrylamide gel electrophoresis (SDS-PAGE) gel. Following SDS-PAGE separation, the proteins were transferred to a nitrocellulose (NC) membrane. The membrane was blocked with 5% non-fat milk and incubated with primary antibodies overnight at 4 °C, including Fas (R&D, AF435, USA), Caspase-8 (R&D, AF705, USA), Cleaved Caspase-8 (Cell Signaling Technology, CST8592, USA), Caspase-3 (Novus, NB100-56708, USA), Cleaved Caspase-3 (Cell Signaling Technology, CST9664, USA), Bcl-2 (R&D, AF810, USA), phospho-Bcl-2 (Affinity, AF3138, UK), with GAPDH (Proteintech, hp-60004, USA) serving as the loading control. Subsequently, the membrane then underwent a 1-h room temperature incubation with HRP-linked secondary antibodies for anti-mouse, anti-rabbit, and anti-goat IgG (Proteintech, SA00001-2, SA00001-1, SA00001-4, USA). In addition, the extraction of complete protein from the sorted CD4^+^ T lymphocytes followed the aforementioned technique, and the expression of the following specific proteins was determined: Foxp3 (BOSTER, MA00011, China), STAT5, phospho-STAT5, HIF-1α (Cell Signaling Technology, CST94205, CST4322, CST36169, USA), and Eos (Abcam, ab220329, USA). ECL chemiluminescence reagents were used to visualize protein bands. The bands were quantificated by Image J (Version 1.8.0, USA).

### Statistical analysis

Data were analyzed using SPSS 20.0 and visualized with Graph Pad Prism 8.3. Results are presented as mean ± standard deviation (SD). For normally distributed data with identical variances, a one-way ANOVA was conducted, alongside the Tukey–Kramer test to compare various groups. Otherwise, non-parametric tests were utilized. *P* < 0.05 indicates statistical significance.

## Results

### Identification of chemical compositions of MGEG

**Fig. 1:**
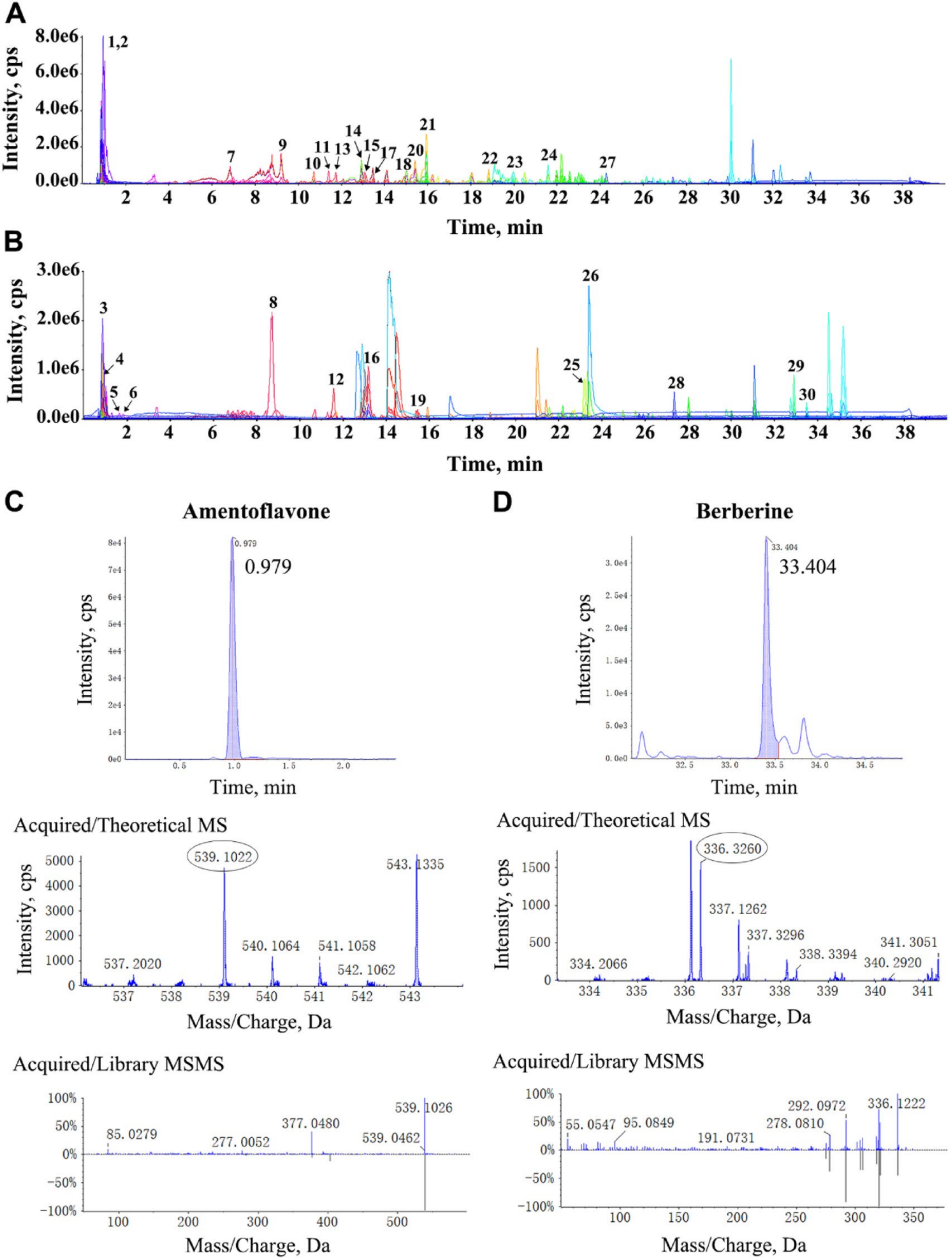
The chemical components of MGEG identified by HPLC-ESI/MS. Total ion chromatogram of MGEG extract in negative (**A**) and positive (**B**) ion modes. The retention time spectra, primary mass spectra, and secondary mass spectra of (**C**) amentoflavone and (**D**) berberine in MGEG

The MGEG water extract was subjected to chemical profiling using HPLC-ESI/MS. As shown in Fig. 1A-B, a total of thirty compounds were identified in both positive and negative ionization modes, including flavonoids, alkaloids, saponins, organic acids, and other constituents. Retention times were determined by comparison with a blank solvent. Molecular formulas and structures were elucidated by comparing acquired MS and MS/MS data with theoretical and library spectra. As shown in Fig. 1C-D and Table S1, amentoflavone and berberine were detected at retention times of 0.979 and 33.404 min, respectively. The mass error in the primary MS data was within 5 ppm, and both Fit and RFit scores were above 85%. These results supported the reliability of the analytical method and the consistency of the MGEG preparation. The full list of identified compounds was presented in Table 2.

**Table 2:** Chemical components of MGEG identified by HPLC-ESI/MS

Peak	*t*_R_(time)	Formula	Identification	Area
1	0.82	‌C_6_H_9_N_3_O_2_	Histidine	5.83E + 05
2	0.82	C_6_H_14_N_4_O_2_	Arginine	1.47E + 06
3	0.94	C_7_H_7_NO_2_	Trigonelline	4.95E + 05
4	0.97	C_30_H_18_O_10_	Amentoflavone	2.85E + 05
5	1.84	C_10_H_13_N_5_O_4_	Adenosine	9.45E + 05
6	1.98	C_10_H_13_N_5_O_5_	Guanosine	1.46E + 05
7	6.98	C_7_H_12_O_6_	Quinic acid	1.27E + 06
8	8.73	C_20_H_24_NO_4_^+^	Magnoflorine	2.39E + 07
9	9.49	C_11_H_12_N_2_O_2_	Tryptophan	3.46E + 05
10	10.72	C_22_H_22_O_10_	Calycosin-7-o-glucoside	4.51E + 06
11	11.41	C_27_H_30_O_16_	Rutin	4.28E + 06
12	11.70	C_21_H_22_NO_4_	Palmatine	9.44E + 05
13	11.75	C_20_H_24_O_9‌‌_	Nodakenin	6.74E + 06
14	12.93	C_22_H_22_O_10_	Calycosin	8.58E + 06
15	13.03	C_22_H_26_O_8‌‌_	Syringaresinol	1.70E + 05
16	13.19	C_20_H_20_NO_4_^+^	Jatrorrhizine	8.93E + 06
17	13.45	C_22_H_22_O_9‌‌_	Ononin	6.49E + 06
18	15.18	C_47_H_80_O_18_‌‌	Notoginsenoside R1	9.51E + 05
19	15.36	C_12_H_8_O_4‌‌_	Methoxsalen	8.84E + 04
20	15.43	C_16_H_12_O_4_‌‌	Formononetin	8.72E + 06
21	15.95	C_42_H_72_O_14_	Ginsenoside Rg1	2.46E + 07
22	18.98	C_41_H_68_O_14_	Astragaloside A	4.03E + 04
23	19.95	C_42_H_72_O_13‌‌_	Ginsenoside Rg2	1.81E + 06
24	21.77	C_26_H_43_NO_6_	Glycocholic acid	1.88E + 05
25	23.12	C_53_H_90_O_22_	Ginsenoside Rb2	4.44E + 05
26	23.35	C_19_H_20_O_5‌‌_	Decursin	6.16E + 06
27	24.14	C_41_H_66_O_12_	α-Hederin	2.03E + 04
28	27.48	C_33_H_58_O_14_	Gingerglycolipid B	2.65E + 04
29	33.41	C_20_H_18_NO_4_^+^	Berberine	1.58E + 05
30	33.74	C_32_H_50_O_13‌‌_	Rubusoside‌‌	2.01E + 04

### MGEG alleviated immune-mediated hematopoietic dysfunction and pathological injury in AA mice

**Fig. 2:**
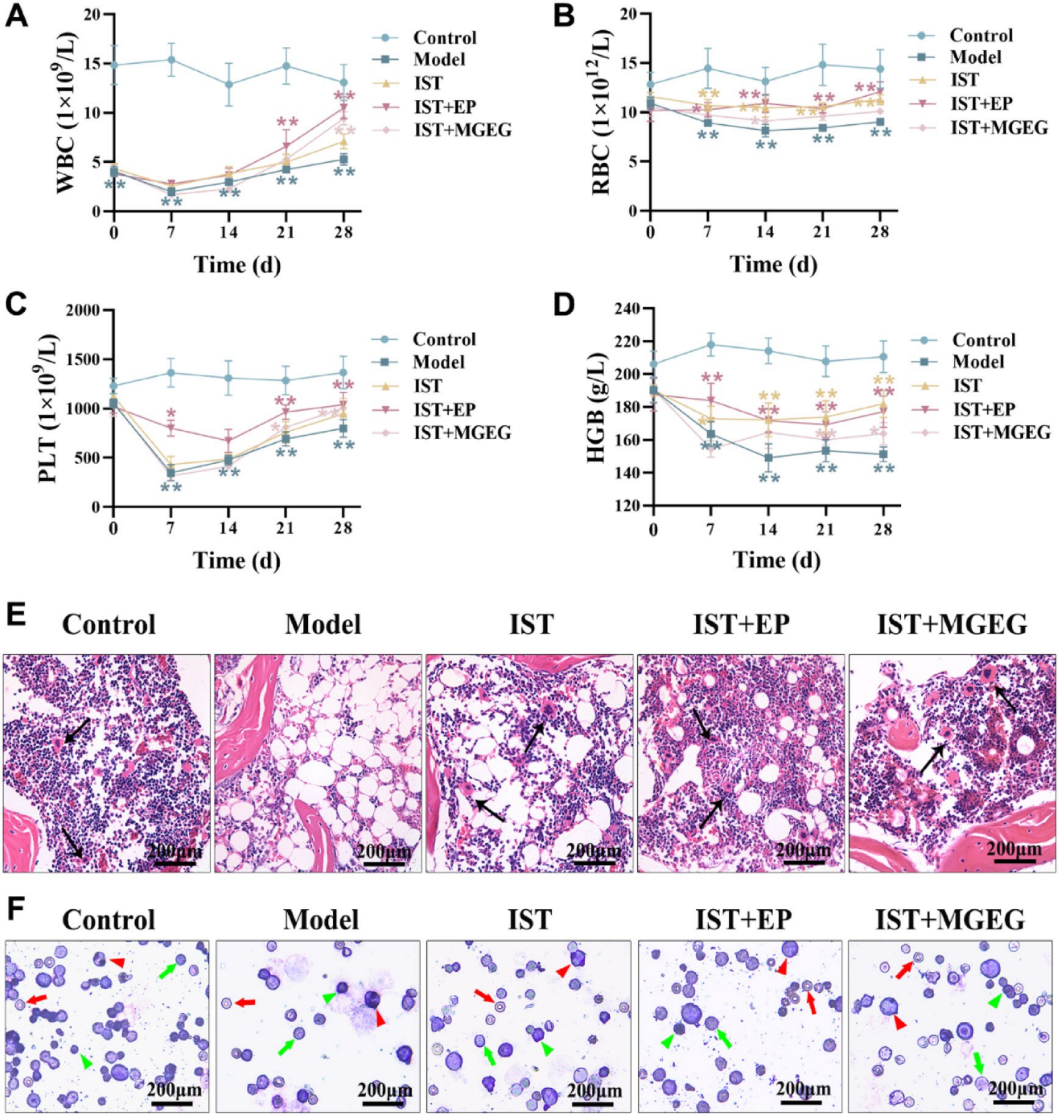
MGEG improved hematopoietic function and pathological immune injury in AA mice. **A-D** Quantify the hematological parameters of peripheral blood, *n* = 6. (**A**) WBC, white blood cell; (**B**) RBC, red blood cell; (**C**) PLT, platelet; (**D**) HGB. hemoglobin. **E** HE staining of femoral bone marrow. The arrows indicate nucleated cells. 40 × images scale bar = 200 µm. **F** Wright-Giemsa staining of bone marrow cells. Red arrows indicate RBC, green arrows indicate PLT, red triangles indicate monocytes, and green triangles indicate lymphocytes. 40 × images scale bar = 200 µm. All data are shown as mean ± SD. Significance was indicated as *P < 0.05, **P < 0.01

Hematological analysis indicated the alterations of hematopoietic function in AA mice. Before the treatment begins, the WBC count in the AA model group significantly decreased compared to the control group. On the 7th day after modeling, PLT count significantly decreased, and on the 14th day, RBC and HGB levels significantly decreased. After 28 days of treatment, compared with the model group, the IST + MGEG group showed a significant increase in WBC, PLT, and HGB counts. Although the increase in RBC count was not statistically significant, an upward trend was observed (Fig. 2A-D). Moreover, the combination of IST + MGEG resulted in a greater improvement in WBC levels compared to IST alone.

To evaluate the effect of IST combined with MGEG therapy on bone marrow pathology following immune-mediated injury, we performed HE staining on mouse femurs and Wright Giemsa staining on bone marrow cells. Compared to the control group, AA mice exhibited significant vacuolization of adipocytes and a reduction in nucleated cells in bone marrow. Treatment with IST alone reduced fat vacuolization, and this effect was further enhanced in the IST + EP and IST + MGEG groups. Notably, the IST + MGEG group showed a progressive replacement of adipocytes by dense clusters of nucleated cells (Fig. 2E). Wright-Giemsa staining confirmed a decrease in nucleated cells and increased morphological variation in RBC in the bone marrow. PLT counts were significantly increased in the IST + EP group, and RBC levels were significantly elevated in the IST + MGEG group (Fig. 2F). These results suggested that the combination of IST + MGEG alleviated immune-mediated bone marrow injury and supported the recovery of hematopoietic function in AA mice.

### MGEG promoted the proliferation of HSCs and attenuated the apoptosis of BMCs

**Fig. 3:**
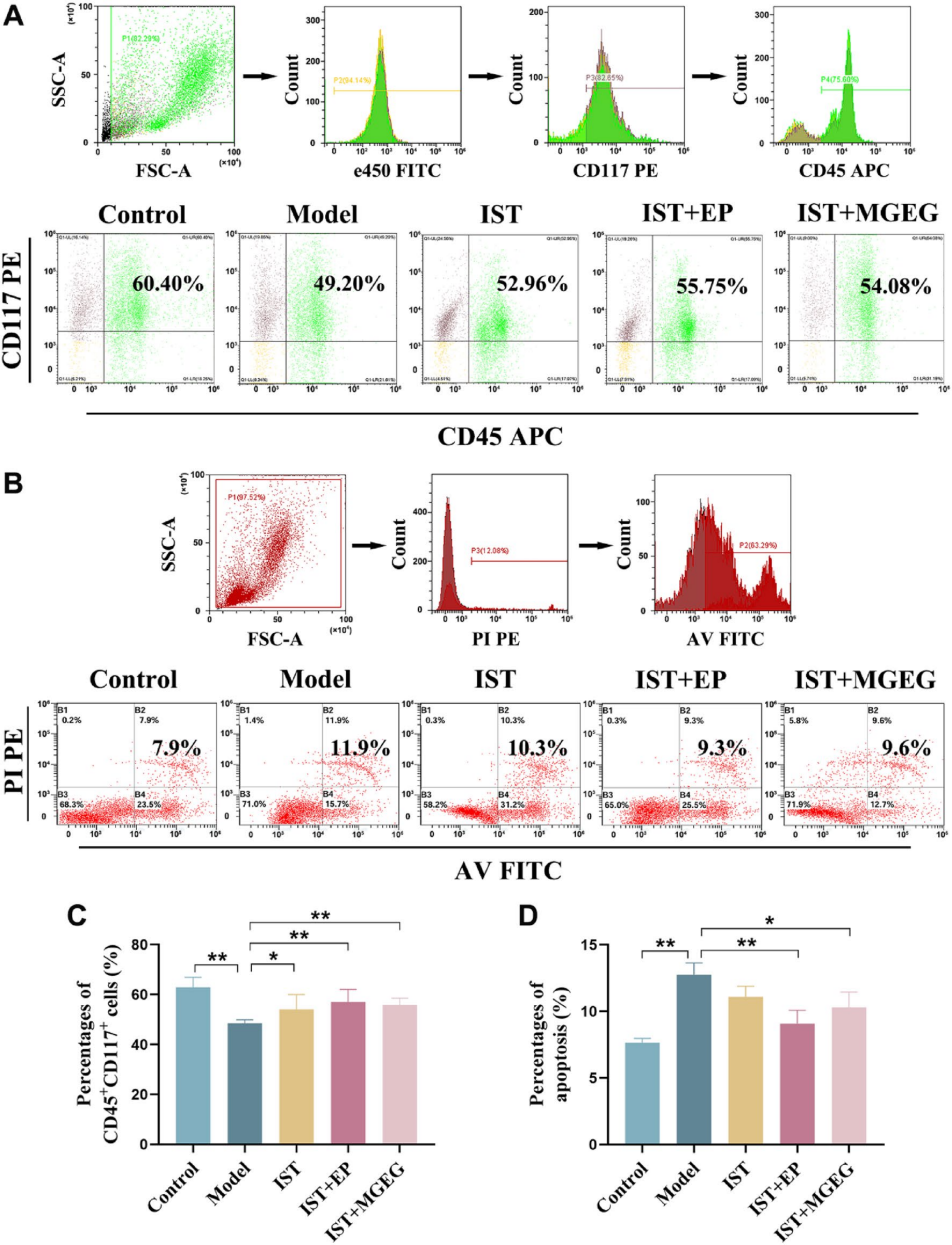
MGEG promoted the proliferation of HSCs and attenuated the apoptosis of BMCs. **A, C** Analysis of the proliferation of CD117^+^CD45^+^HSCs and its quantitative assessment by flow cytometry, *n* = 6. **B, D** Analysis of the apoptosis of BMCs and its quantitative assessment by flow cytometry, *n* = 6. All data are shown as mean ± SD. Significance was indicated as *P < 0.05, **P < 0.01

Flow cytometry analysis showed that the proportion of HSCs in the bone marrow of AA mice was significantly reduced (Fig. 3A, C), while the apoptosis rate of BMCs was significantly increased (Fig. 3B, D). After treatment, the proportion of HSCs in all intervention groups significantly increased compared to the model group. The combination of IST + EP or IST + MGEG led to a significant reduction in BMCs apoptosis, whereas IST alone did not show a statistically significant effect in suppressing apoptosis. These findings indicated that the combination of IST + MGEG was associated with enhanced HSCs proliferation and reduced apoptosis of BMCs.

### MGEG restored Th1/Th2 and Th17/Treg cell balance

**Fig. 4:**
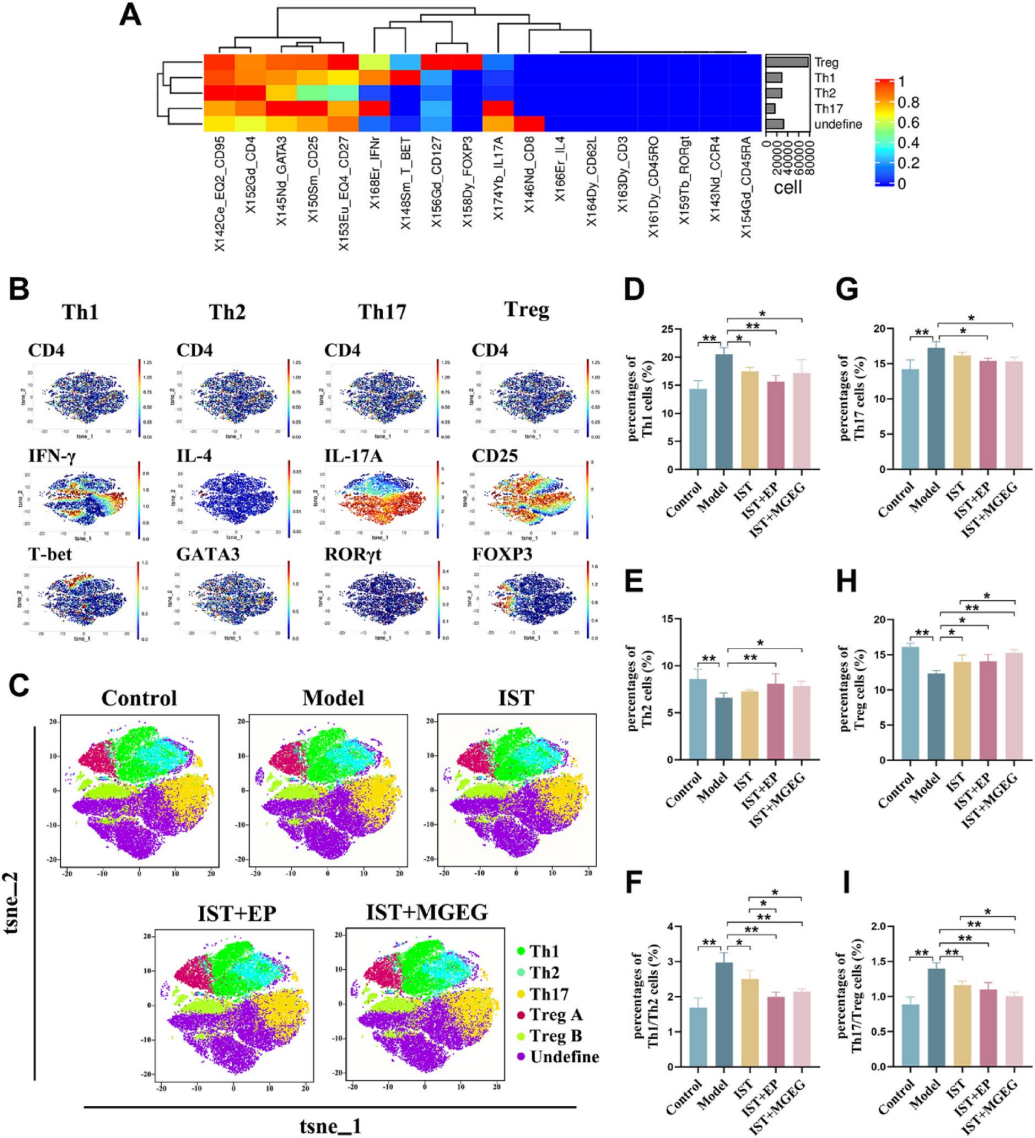
MGEG restored Th1/Th2 and Th17/Treg cells balance in peripheral blood. **A** Heat map of the expression of markers related to CD4^+^T lymphocyte subsets. **B** *t*-SNE visualization of surface markers and specific transcription factors in Th1, Th2, Th17, and Treg cells. **C** Cluster analysis and *t*-SNE visualization of Th1, Th2, Th17, and Treg cells. **D-F** Quantitative analysis for Th1, Th2 and Th1/Th2 cells, *n* = 3. **G-I** Quantitative analysis for Th17, Treg, and Th17/Treg cells, *n* = 3. All data are shown as mean ± SD. Significance was indicated as *P < 0.05, **P < 0.01

We investigated the regulatory effects of combined IST + MGEG therapy on the Th1/Th2 and Th17/Treg cell differentiation balance. Utilizing the high-throughput, full-spectrum capabilities of CyTOF-2 mass spectrometry flow cytometry, we simultaneously analyzed multiple cellular parameters in CD4^+^ T lymphocytes (Fig. 4A). Based on expression signals of marker antibodies specific for Th1, Th2, Th17, and Treg cells, we performed t-SNE clustering for dimensionality reduction and viSNE visualization analysis of cell subsets (Fig. 4B-C). Quantitative analysis showed an imbalance in the Th1/Th2 and Th17/Treg ratios in the model group, characterized by increased differentiation of Th1 and Th17 cells and decreased differentiation of Th2 and Treg cells (Fig. 4D-I). After treatment, all drug-intervention groups showed a trend toward rebalancing CD4⁺ T cell subsets compared to the model group, with increased proportions of Th2 and Treg cells and reduced activation of Th1 and Th17 cells. Notably, the combination of IST + MGEG resulted in a greater increase in Treg cell percentage and a more pronounced restoration of the Th1/Th2 and Th17/Treg balance than IST alone.

### MGEG modulated the proliferation and differentiation of Treg cell subsets

**Fig. 5:**
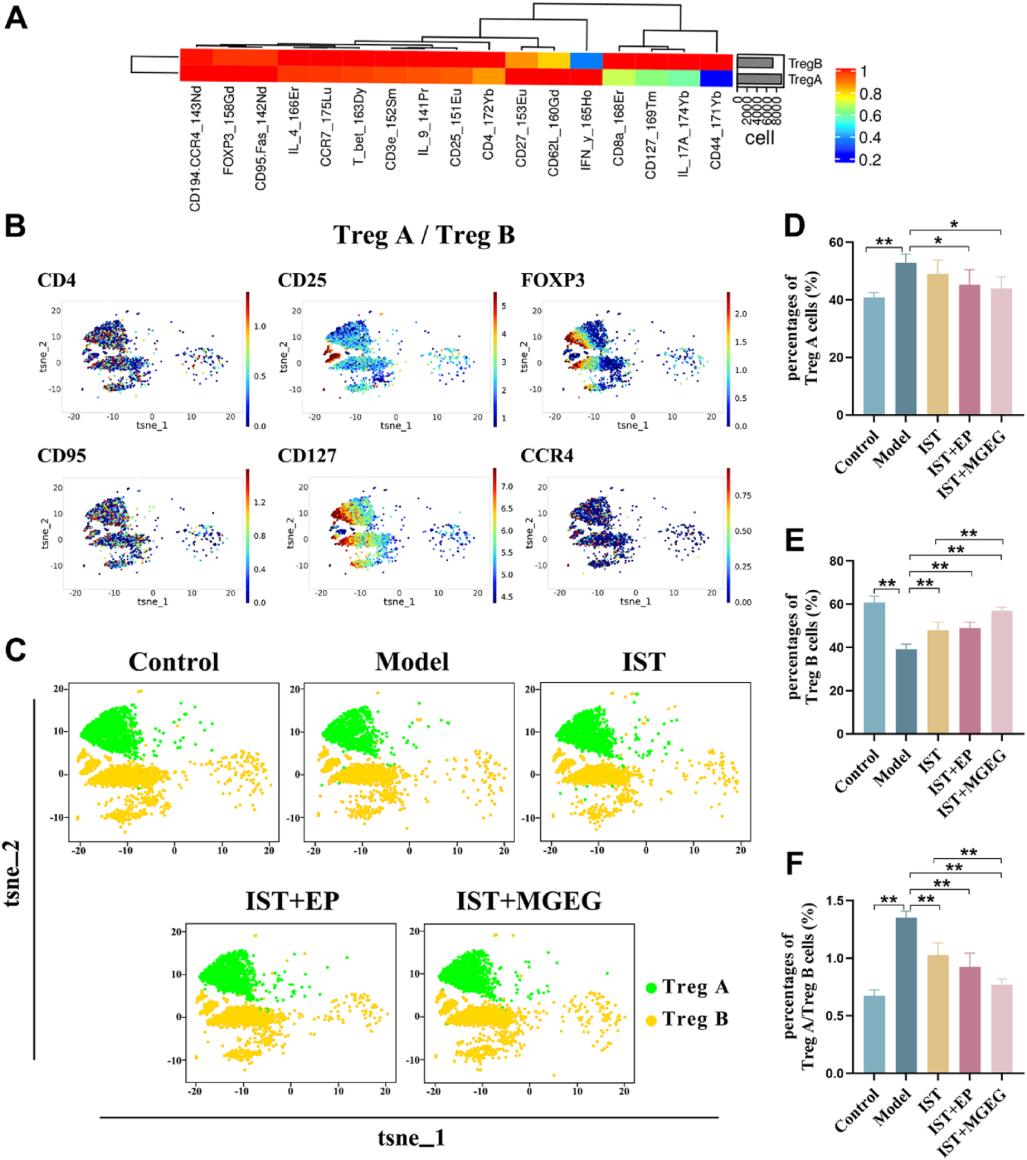
MGEG modulated the proliferation and differentiation of Treg A and Treg B cells. **A** Heat map of the expression of markers in Treg A and Treg B cells. **B** *t*-SNE visualization of surface markers and specific transcription factors Treg A and Treg B cells. **C** Cluster analysis and *t*-SNE visualization of Treg A and Treg B cells. **D-F** Quantitative analysis for Treg A, Treg B, and Treg A/Treg B cells, *n* = 3. All data are shown as mean ± SD. Significance was indicated as *P < 0.05, **P < 0.01

Based on the expression of Treg cell-specific markers, we identified two distinct Treg cell subsets utilizing t-SNE dimensionality reduction and viSNE visualization techniques: Treg A cells (CD4^+^ CD25^+^ FOXP3^+^ CD95^−^ CD127^−^ CCR4^+^) and Treg B cells (CD4^+^ CD25^+^ FOXP3^+^ CD95^+^ CD127^−^ CCR4^−^) (Fig. 5A-C). A significant shift in the distribution of Treg subsets was observed in the AA model group. Specifically, the proportion of Treg A cells increased while that of Treg B cells decreased, resulting in a significantly higher Treg A/B ratio compared to controls (Fig. 5D-F). After treatment, all groups exhibited a significant decrease in the Treg A/B ratio and a significant increase in the percentage of Treg B cells. Notably, the combination of IST + MGEG resulted in a more pronounced increase in the overall proportion of Treg cells and a greater reduction in the Treg A/B ratio compared to IST alone. These results indicated that the enhanced efficacy of IST combined with MGEG could be associated with the modulation of Treg cell subset distribution and dynamics.

### MGEG improved the inflammatory hematopoietic microenvironment in AA mice

**Fig. 6:**
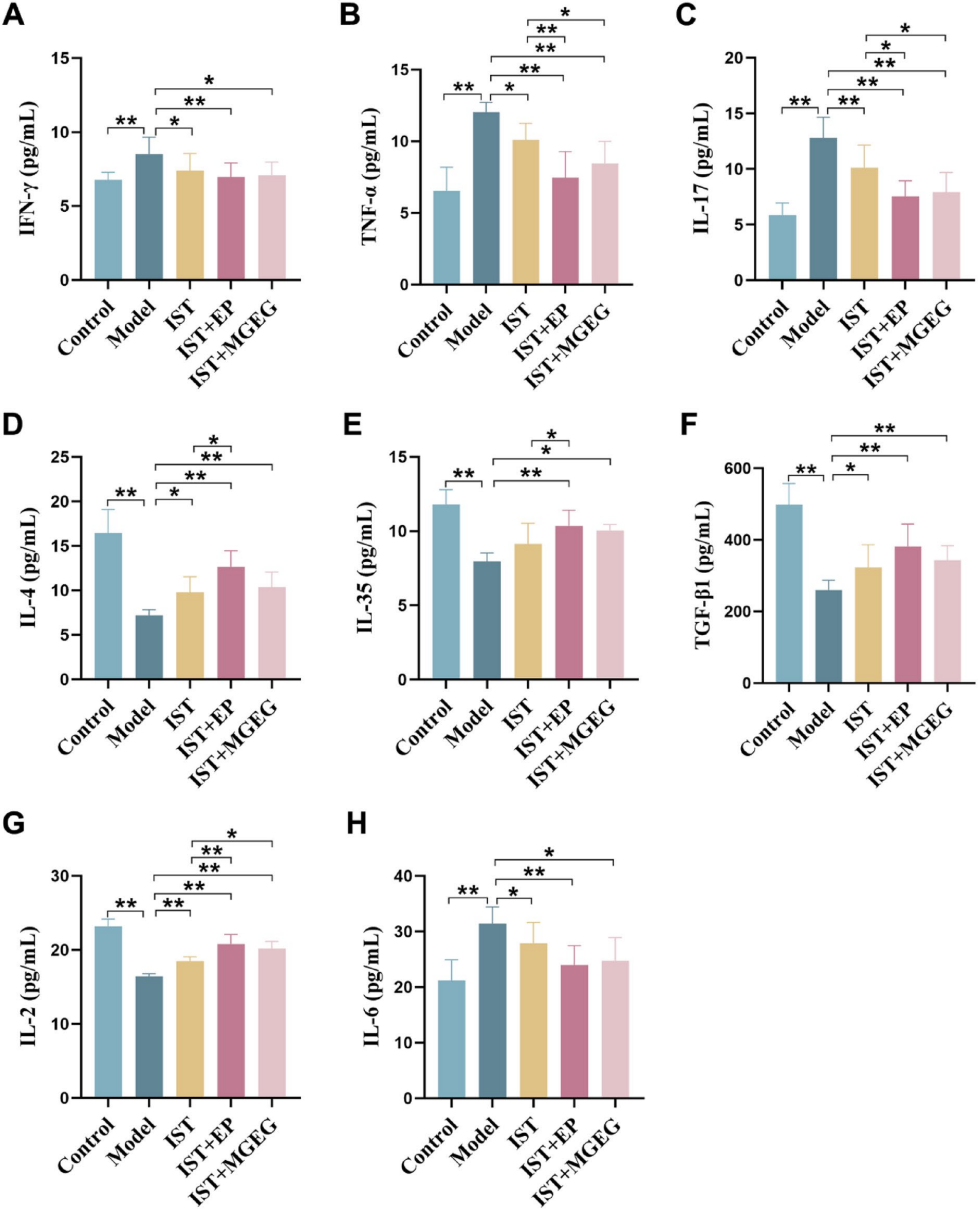
MGEG regulated the levels of cytokines. **A–H** The levels of IFN-γ, TNF-α, IL-17, IL-4, IL-35, TGF-β1, IL-2, and IL-6 in serum. All data are shown as mean ± SD, *n* = 6. Significance was indicated as *P < 0.05, **P < 0.01

Serum cytokine levels were determined by ELISA. The model group showed significantly elevated levels of IFN-γ, TNF-α, IL-17, and IL-6 compared to the control group (Fig. 6A-C, G), while TGF-β1, IL-35, IL-4, and IL-2 levels were significantly reduced (Fig. 6D-F, H). After treatment, the alterations in these cytokine levels were partially normalized across the treatment groups. Compared with the model group, significant reductions in serum levels of pro-inflammatory cytokines INF-γ, TNF-α, and IL-17 were observed in the IST + MGEG group, while serum levels of anti-inflammatory cytokines IL-35 and IL-4 were significantly increased. Compared with the IST group alone, the inflammatory status of the IST + MGEG and IST + EP groups was significantly improved, with significantly reduced levels of TNF-α and IL-17, and significantly increased levels of IL-2. A trend toward increased levels of IL-4, IL-35, and TGF-β1 was observed in the IST + MGEG group. The difference from the IST group was not statistically significant. These results demonstrated that the combination of IST + MGEG could alleviate the inflammatory state in the bone marrow hematopoietic microenvironment.

### MGEG inhibited Treg cells apoptosis mediated by the Fas/FasL signaling pathway

**Fig. 7:**
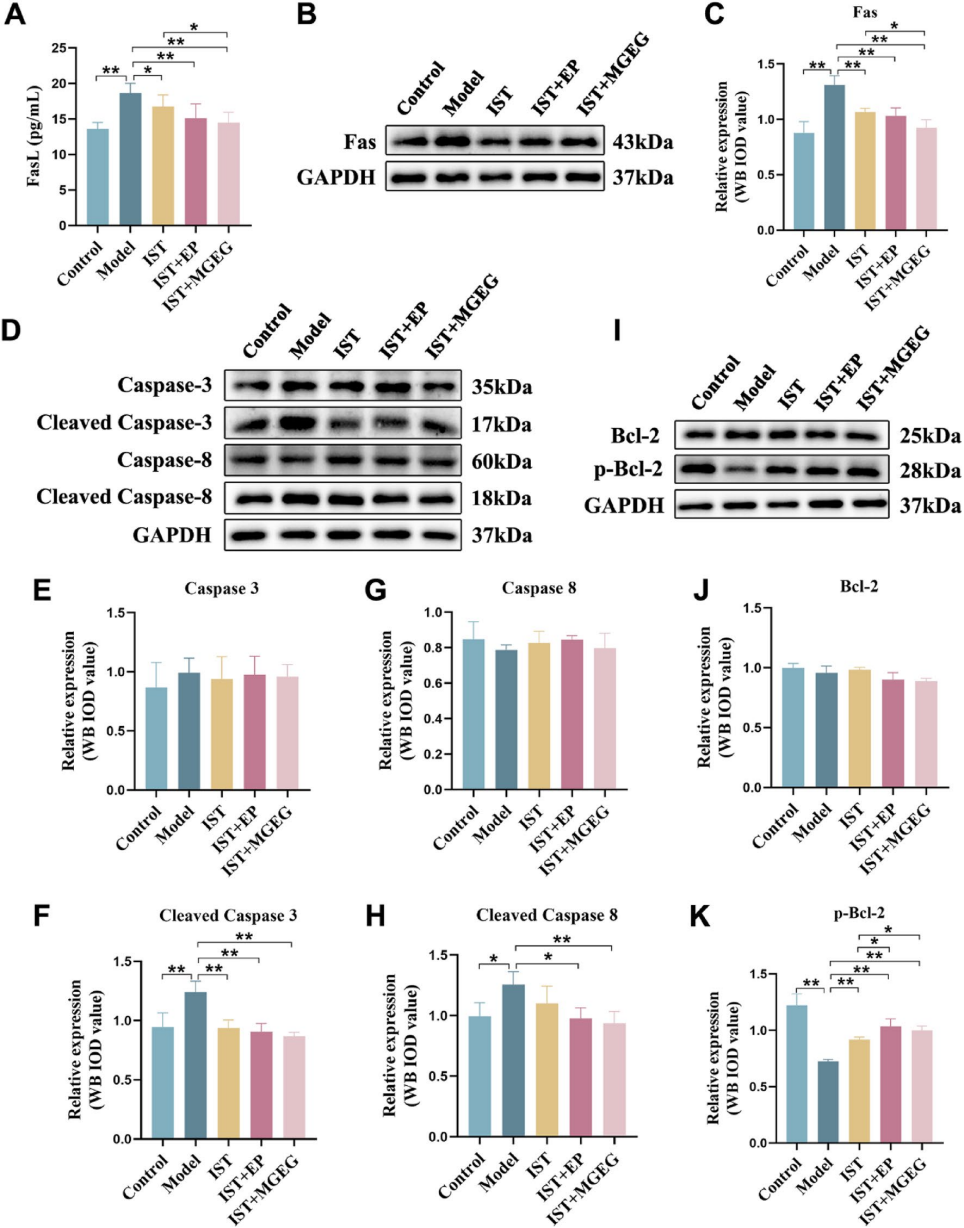
MGEG inhibited Fas/FasL-mediated Treg cell apoptosis. **A** The level of FasL in serum, *n* = 6. **B**, **C** The protein expression of Fas in Treg cells,* n* = 3. **D–H** The expressions of Caspase-3, Cleaved Caspase-3, Caspase-8, and Cleaved Caspase-8 in Treg cells,* n* = 3. **I-K** The expressions of Bcl-2 and p-Bcl-2 in Treg cells,* n* = 3. All data are shown as mean ± SD. Significance was indicated as *P < 0.05, **P < 0.01

Treg cells were enriched using immunomagnetic beads (Fig. S1), and quantitative Western blot analysis was performed. The results indicated that serum levels of FasL were significantly elevated in the model group compared to those of the control group (Fig. 7A). Furthermore, the protein expression levels of Fas, Cleaved Caspase-3, and Cleaved Caspase-8 were markedly increased in Treg cells (Fig. 7B–H), while p-Bcl-2 expression was notably decreased (Fig. 7I-K). After treatment, these protein expression levels tended to recover across all treatment groups. Notably, the combined treatment of IST with MGEG led to a greater reduction in serum FasL level and Fas expression in Treg cells, compared to IST treatment alone, along with a concurrent increase in p-Bcl-2 expression. These results suggested that the combination of IST + MGEG could more effectively inhibit the Fas/FasL signaling pathway than IST alone, thereby contributing to a reduction in Treg cell apoptosis.

### MGEG promoted the proliferation and differentiation of Treg cells

**Fig. 8:**
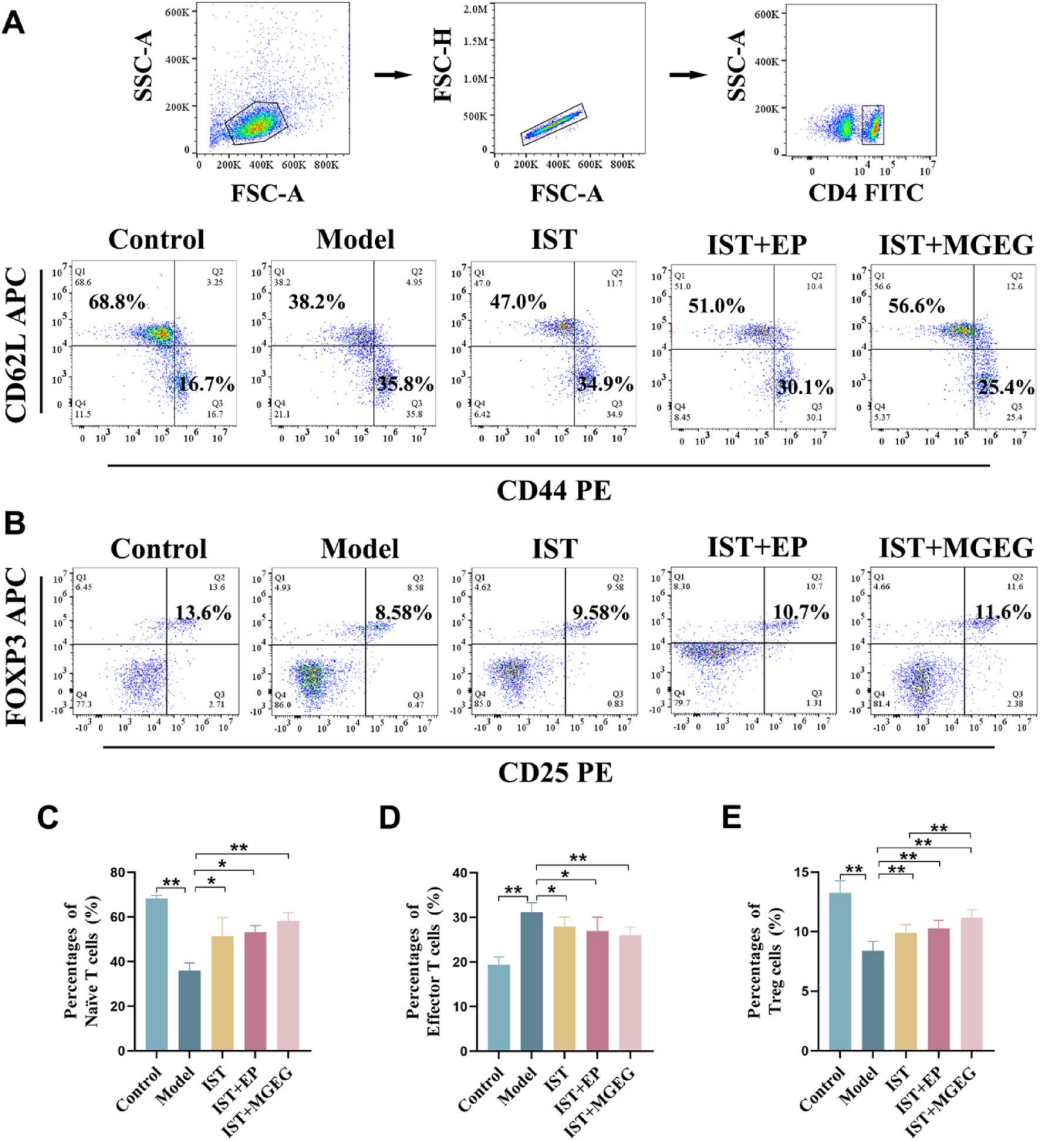
MGEG promoted the proliferation and differentiation of naïve T into Treg cells. **A, B** The proliferation and differentiation of naïve T (Q1:CD4^+^ CD44^low^ CD62L^+^), effector T (Q3:CD4^+^ CD44^high^ CD62L^−^), and Treg cells. **C–E** Quantification analysis of naïve T, effector T, and Treg cells. All data are shown as mean ± SD,* n* = 6. Significance was indicated as *P < 0.05, **P < 0.01

The proportions of naïve T, effector T, and Treg cells in the spleen were quantified by flow cytometry (Fig. 8A, B). As shown in Fig. 8C-E, the model group had a significantly lower proportion of naïve T cells and Treg cells, and a higher proportion of effector T cells than the control group. After drug treatment, all treatment groups showed a significant increase in naïve T cells and Treg cells, and a significant decrease in effector T cells, with the most substantial changes occurring in the IST + MGEG group. Furthermore, the IST + MGEG group exhibited a significantly higher proportion of Treg cells than the IST group. These results indicated that MGEG could enhance the regulatory effect of IST on the dynamics of T-cell subsets, potentially facilitating an increase in Treg cell populations.

### MGEG regulated the IL-2/STAT5 and miR-17-5p/Eos pathways contributing the differentiation of Treg cells

**Fig. 9:**
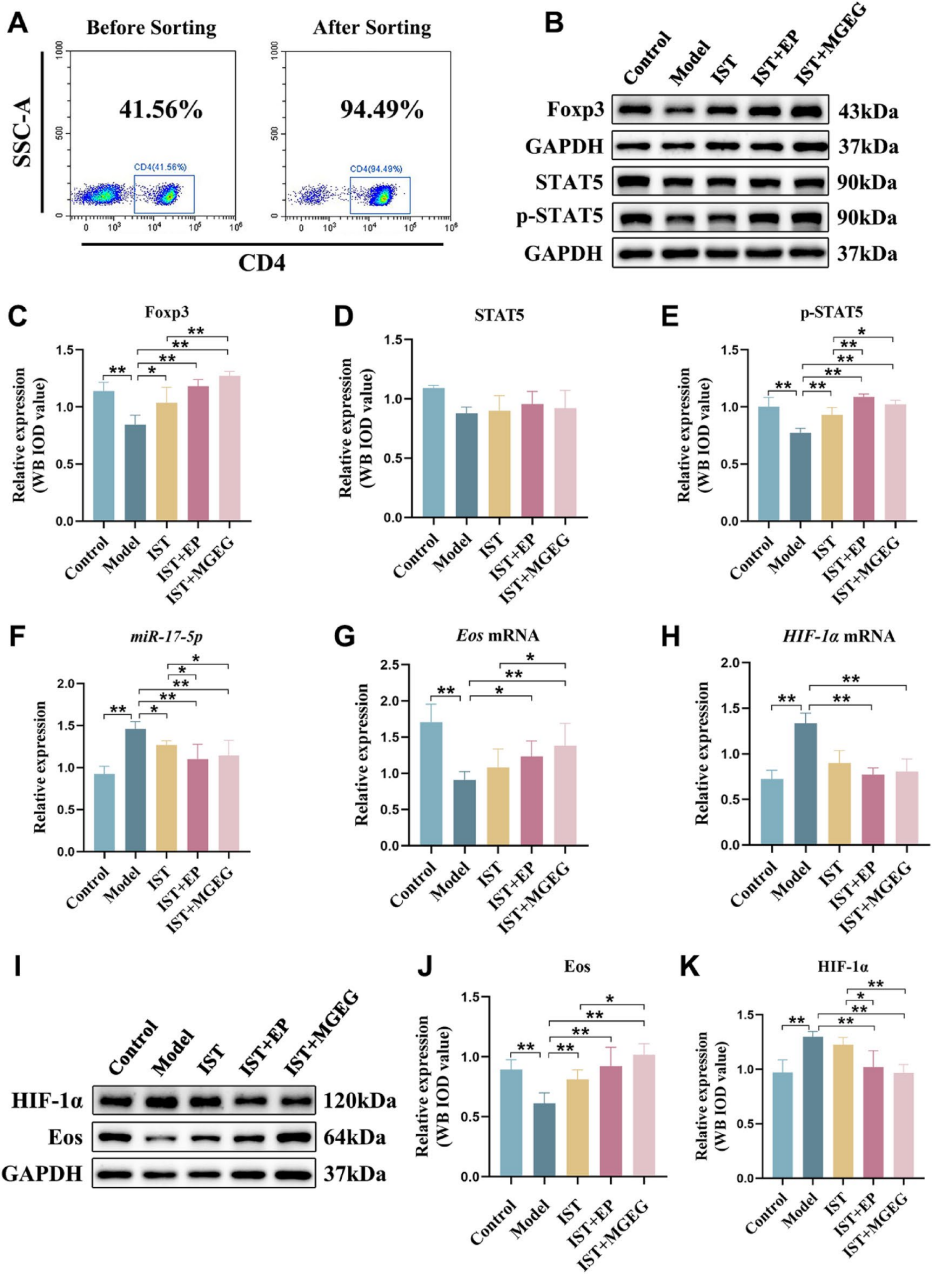
MGEG regulated the IL-2/STAT5 signaling pathway and miR-17-5p to promote the differentiation of Treg cells. **A** Identification of MACS-sorted CD4^+^T by flow cytometry. **B-E** The protein expressions of Foxp3, STAT5, and p-STAT5 in CD4^+^T cells,* n* = 3. **F–H** The levels of miR-17-5p, Eos mRNA, and HIF-1α mRNA in CD4^+^T cells, *n* = 6. **I-K** The protein expressions of Eos and HIF-1α in CD4^+^T cells,* n* = 3. All data are shown as mean ± SD. Significance was indicated as *P < 0.05, **P < 0.01

High-purity CD4⁺ T cells (> 90%) were obtained through immunomagnetic bead enrichment (Fig. 9A). Quantitative analysis of proteins associated with the IL-2/STAT5 signaling pathway revealed that the model group exhibited a significant reduction in both Foxp3 and p-STAT5 expression (Fig. 9B-E), accompanied by decreased serum IL-2 levels (Fig. 6G). After treatment, all drug-administered groups showed significant elevations in Foxp3 and p-STAT5 expressions as well as IL-2 levels in serum compared to the model group. Notably, the combined IST + MGEG treatment resulted in a more pronounced upregulation of these markers than IST alone. The upregulatory function was better than that of IST + EP.

Furthermore, as shown in Fig. 9F-H, *miR-17-5p* and *HIF-1α* mRNA levels were significantly elevated in CD4^+^ T cells in AA mice, whereas *Eos* mRNA expression was markedly reduced. After treatment, *miR-17-5p* expressions were significantly suppressed in all treatment groups. Both the IST + EP and IST + MGEG groups exhibited significantly lower *miR-17-5p* levels than the IST-alone group. Compared to the model group, the IST + MGEG group showed reduced *HIF-1α* mRNA and increased *Eos* mRNA expressions significantly. In contrast, the IST-alone group did not show a statistically significant difference in *Eos* mRNA expression relative to the model group. Western blot analysis further confirmed that the combination of IST + MGEG significantly downregulated HIF-1α protein and upregulated Eos protein expression in CD4⁺ T cells, with a more substantial effect than IST alone (Fig. 9I-K). These findings suggested that MGEG may enhance the regulatory effect of IST on Treg differentiation and function by modulating the IL-2/STAT5 and miR-17-5p/Eos signaling pathways.

## Discussion

AA is an autoimmune disorder of the hematopoietic system characterized by severe depletion of HSCs, primarily mediated by aberrant T-cell activation [21]. While IST is a standard treatment for AA, its efficacy is often limited, and relapse remains a significant concern. In this study, we evaluated whether the combination of IST + MGEG could improve hematopoietic function in AA mice. Our results demonstrated that the combination of IST + MGEG significantly improved peripheral blood counts and pathological state of the bone marrow, reduced adipocyte infiltration, and increased nucleated cell numbers. Moreover, flow cytometry analysis revealed that this combination promoted HSCs proliferation and inhibited BMSs apoptosis. Although the improvement over IST alone was not always statistically significant, a consistent positive trend was observed. Compared with IST used alone, experimental results indicated that MGEG or EP could augment IST efficacy in mitigating immune-mediated bone marrow injury, and the efficacy of the two was comparable.

Treg cells are pivotal to maintaining immune tolerance, and their numerical or functional deficiency is central in AA pathogenesis [22]. In humans, Treg cells can be subdivided into Treg A and Treg B subsets based on immunophenotype and function. Patients with AA often exhibit a reduced total Treg cells count, an increased Treg A/B ratio, and a marked loss of Treg B cells, and the low response rate of IST alone may be related to this [23, 24]. We confirmed a similar imbalance in AA mice through CyTOF-2 analysis, which was significantly reversed by IST combined with MGEG treatment. The combination therapy of MGEG or EP notably increased the proportion of Treg B cells and restored the Treg A/B ratio more effectively than IST alone. At the same time, we found that the improvement effect of MGEG was better than that of EP in this regard, indicating that MGEG has special benefits in regulating Treg subsets.

The maintenance of a functional Treg cells pool depends not only on survival but also critically on their proper differentiation, a process orchestrated by a transcriptional network centered on Foxp3. In peripheral lymphoid tissues, TGF-β1 promotes the differentiation of naïve T cells into induced Treg (ïTreg) cells by inducing Foxp3 expression following TCR stimulation [25]. We observed an increase in effector T cells and a decrease in naïve T cells and Treg cells in AA mice. This means that the depletion of naïve cell pools in AA mice, coupled with sustained T cell immune activation, inhibits the differentiation of Treg. IST combined with MGEG treatment significantly increased the proportion of Treg cells and reduced effector T cells. The limitation in our study was the lack of clear demonstration of the differentiation of naïve T precursors. The concurrent upregulation of p-STAT5 and Foxp3 regulates Treg differentiation through the IL-2/STAT5 pathway. STAT5 deficiency in humans affects the Treg cell activity, while STAT5 knockout in mice inhibits the activation of peripheral T cell differentiation into Treg cells [26, 27]. Mechanistically, IL-2 binding to the IL-2R (CD25) on Treg precursors triggers STAT5 phosphorylation. Its nuclear translocation leads direct binding to the CNS2 enhancer region of the Foxp3 locus, promotes Foxp3 transcription and Treg differentiation [28, 29]. IST combined with MGEG treatment elevated serum IL-2, boosted p-STAT5 and Foxp3 protein levels in CD4⁺ T cells, with a more pronounced effect than IST alone, suggesting that MGEG could enhance IL-2 availability and STAT5 signaling to foster Treg differentiation.

In addition to the IL-2/STAT5 pathway, the stability of Treg is also regulated by the miR-17-5p/Eos pathway. The miR-17-5p post-transcriptionally regulates Treg cell function by targeting the transcript encoding Eos (Ikzf4) [30]. Eos, a Foxp3 cofactor highly expressed in Treg cells, binds to the 148–198 deletion region of *Foxp3* (ΔFoxp3) to maintain Treg cells suppressive function [15, 31]. Inflammatory cues such as IL-6 can upregulate miR-17-5p, which inhibit the transcription and expression of Eos. HIF-1α can further promote miR-17-5p expression under inflammatory conditions [15]. The combination of IST + MGEG reduced *miR-17-5p* and *HIF-1α* expression in CD4⁺ T cells, increased *Eos* mRNA and protein levels, and down-decreased the levels of IL-6. These results suggested that the IST + MGEG treatment had a multi-level regulation of Treg function via the miR-17-5p/Eos pathway. Although MEGE may not be as effective in regulating the IL-2/STAT5 signaling pathway as IST + EP, improving the miR-17-5p/Eos signaling pathway was better to IST + EP or IST alone. An important consideration was the potential interaction among these signaling pathways. Our data suggested that the IL-2/STAT5, and miR-17-5p/Eos pathways could regulate the differentiation and function of Treg cells synergistically. MGEG enhanced Treg differentiation primarily by elevating IL-2 levels, which activated the function of STAT5 and upregulated the expression of Foxp3, reinforced Bcl-2 activity to suppress apoptosis. MGEG intervened in the transcription of miR-17-5p in Treg cells, modulated the genetic transcription of *Eos*. The detection found that the expression of Eos and Foxp3 proteins in Treg cells increased, the secretion of anti-inflammatory factor IL-35 increased, the proportion of effector T cells decreased, and the pro-inflammatory factors TNF-α and IFN-γ significantly decreased after treatment by IST + MGEG in mice. This indicates that MEGE effectively promotes Treg function and development and inhibits effector T cells activation through the IL-2/STAT5 and miR-17-5p/Eos signaling pathways. The anti-apoptotic effect represents a convergent outcome of signaling through these cascades. MGEG, as a multi-component formulation, appeared to orchestrate a system-level modulation for this network, exemplifying a key advantage of herbal medicine.

Treg depletion is primarily mediated by the Fas/FasL pathway. The elevated expression of Fas on Treg B cells renders them highly susceptible to Fas/FasL-mediated apoptosis, thereby accounting for their selective depletion in AA [24]. Activated CD4⁺ T cells in AA overproduce inflammatory cytokines such as IFN-γ and TNF-α, which can upregulate Fas expression in HSCs and Treg cells, leading to apoptosis [32, 33]. Our data showed that the IST + MGEG or IST + EP treatment could inhibit the proliferation of effector T cells, and rebalance Th1/Th2 and Th17/Treg ratios, reduced pro-inflammatory cytokines while enhancing anti-inflammatory factors such as IL-4, IL-35, and TGF-β1 significantly. IL-35, as an inhibitory cytokine mainly secreted by Treg cells, can inhibit effector T cells proliferation and promote Treg expansion [34]. The above results indicated that IST + MGEG or IST + EP could promote the secretion of anti-inflammatory factors IL-35 and TGF-β1 in Treg, inhibited the activation of effector T cells, reduced the secretion of inflammatory cytokines IFN-γ, TNF-α, IL-17, and improved the inflammatory environment. It also indirectly demonstrated the indispensable role of Eos in maintaining the immune function stability of Treg. Furthermore, Western blot analysis showed that these treatments could elevate the expression of Fas and the actions of Cleaved Caspase-3, and Cleaved Caspase-8 in Treg cells of AA mice and increase the serum level of FasL. The IST + MGEG treatment suppressed these apoptotic markers and enhanced the level of p-Bcl-2, inhibited Caspase-3 activation, antagonizing the Treg cells apoptosis [35]. Notably, these effects were more pronounced than those of IST alone.

Notably, HPLC-ESI/MS analysis revealed that several components identified in MGEG have been reported to modulate pathways relevant to this study. For instance, amentoflavone and calycosin have been associated with the regulation of apoptotic proteins (e.g., Fas, FasL, Caspases), while berberine and calycosin are known to influence miR-17-5p signaling [36–39]. Furthermore, amentoflavone, rutin, and ononin have demonstrated effects on HIF-1α expression [40, 41]. While direct evidence linking specific components to the observed bioactivities remains limited, this provides a plausible basis for its multi-targeted effects. When compared with IST + EP treatment, a current clinical standard, IST + MGEG treatment achieved comparable improvements in hematological recovery and Treg reconstitution. However, EP acts primarily as a TPO receptor agonist to stimulate hematopoiesis and may indirectly promote residual Treg cells proliferation by improving the inflammatory milieu, whereas MGEG directly targets the function and differentiation of Treg cells through immune regulatory pathways, enhancing their immunosuppressive function and inhibiting the activation of effector T cells. This multi-target action could help the reestablish of immune homeostasis and potentially reduce relapse risk during IST tapering. Nevertheless, long-term safety and species-specific pharmacokinetic differences warrant further investigation.

## Conclusions

In conclusion, our results indicated that MGEG could promote the Treg cells differentiation through the IL-2/STAT5 and functional recovery through the miR-17-5p/Eos pathways, inhibit the activation of effector T cells, and suppress Fas/FasL mediated Treg apoptosis, thereby enhances the therapeutic effect of IST for AA treatment. This study integrated traditional herbal medicine with modern immunosuppressive therapy, offered a novel combinatorial strategy for IST treatment.

## Supplementary Information

Additional file 1.

Additional file 2.

## Data Availability

Data is provided within the supplementary information files.

## Abbreviations

AA: Aplastic anemia
AML: Acute myelogenous leukemia
ATG: Anti-thymocyte globulins
CsA: Cyclosporine A
EP: Eltrombopag
FasL: Fas ligand
Foxp3: Transcription factor forkhead box protein 3
HBG: Hemoglobin
HE: Hematoxylin-eosin
HIF-1α: Hypoxia inducible factor-1α
HSCs: Hematopoietic stem cells
HSCT: Hematopoietic stem cell transplantation
IFN-γ: Interferon-γ
IL-2: Interleukin-2
IST: Immunosuppressive therapy
MDS: Myelodysplastic syndrome
MGEG: Modified Guilu Erxian Glue
PLTs: Platelets
RBC: Red blood cells
STAT5: Signal transductor and transcriptional activator 5
TNF-α: Tumor necrosis factor-α
Treg: Regulatory T cells
WBC: White blood cells
