# Supplementary figures and images for: Modified Guilu Erxian Glue regulates Treg immune function to suppress bone marrow failure in aplastic anemia mice (part 2 of 2)
Source: Chin Med. 2025 Nov 20;20:197. doi: 10.1186/s13020-025-01266-z (PMC12632029; doi:10.1186/s13020-025-01266-z)

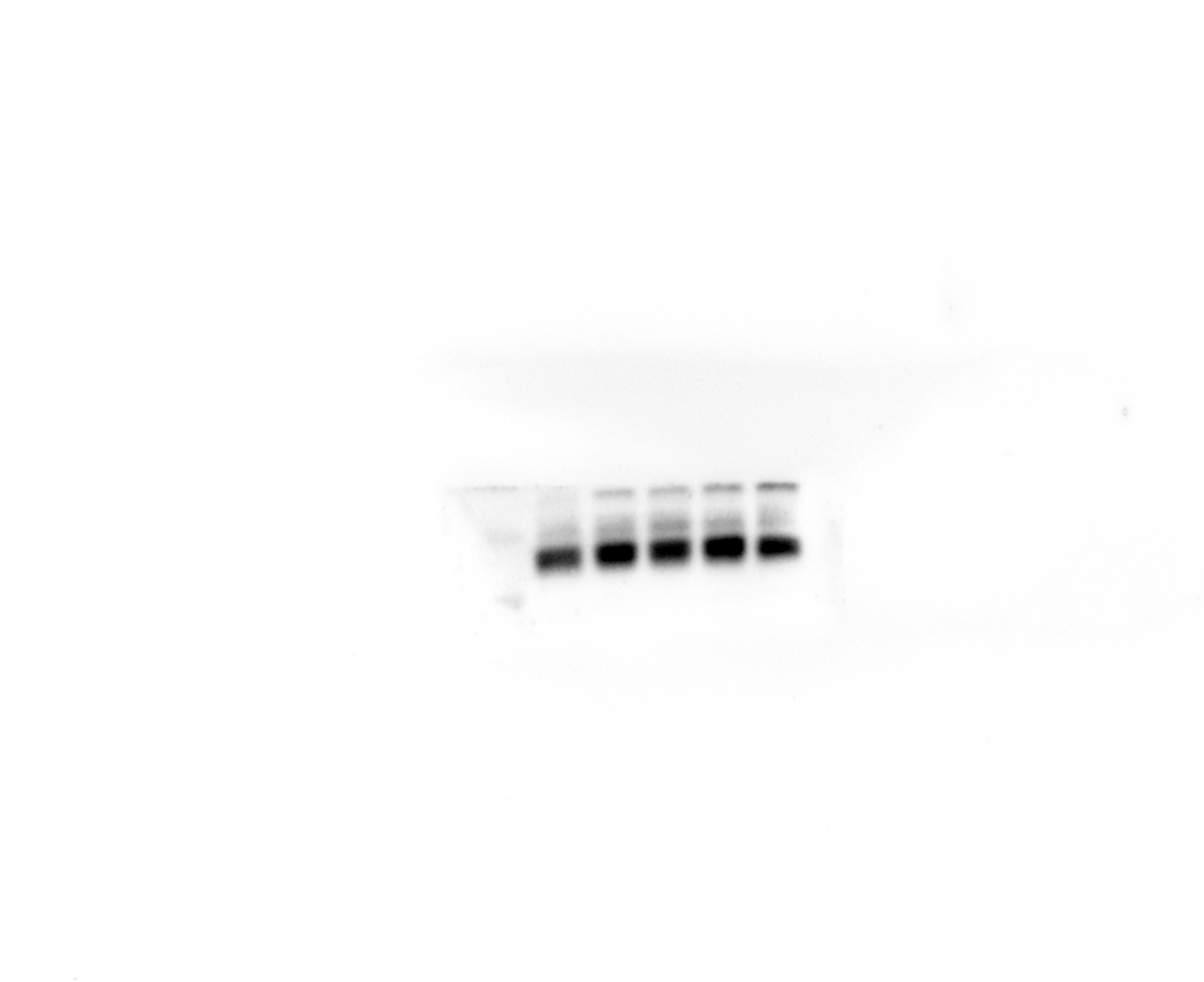

Supplement: Supplementary file 1 — Additional file 1. [file 13020_2025_1266_MOESM1_ESM.zip › Figure 7/bands/Cle-Caspase 3-2.Tif]

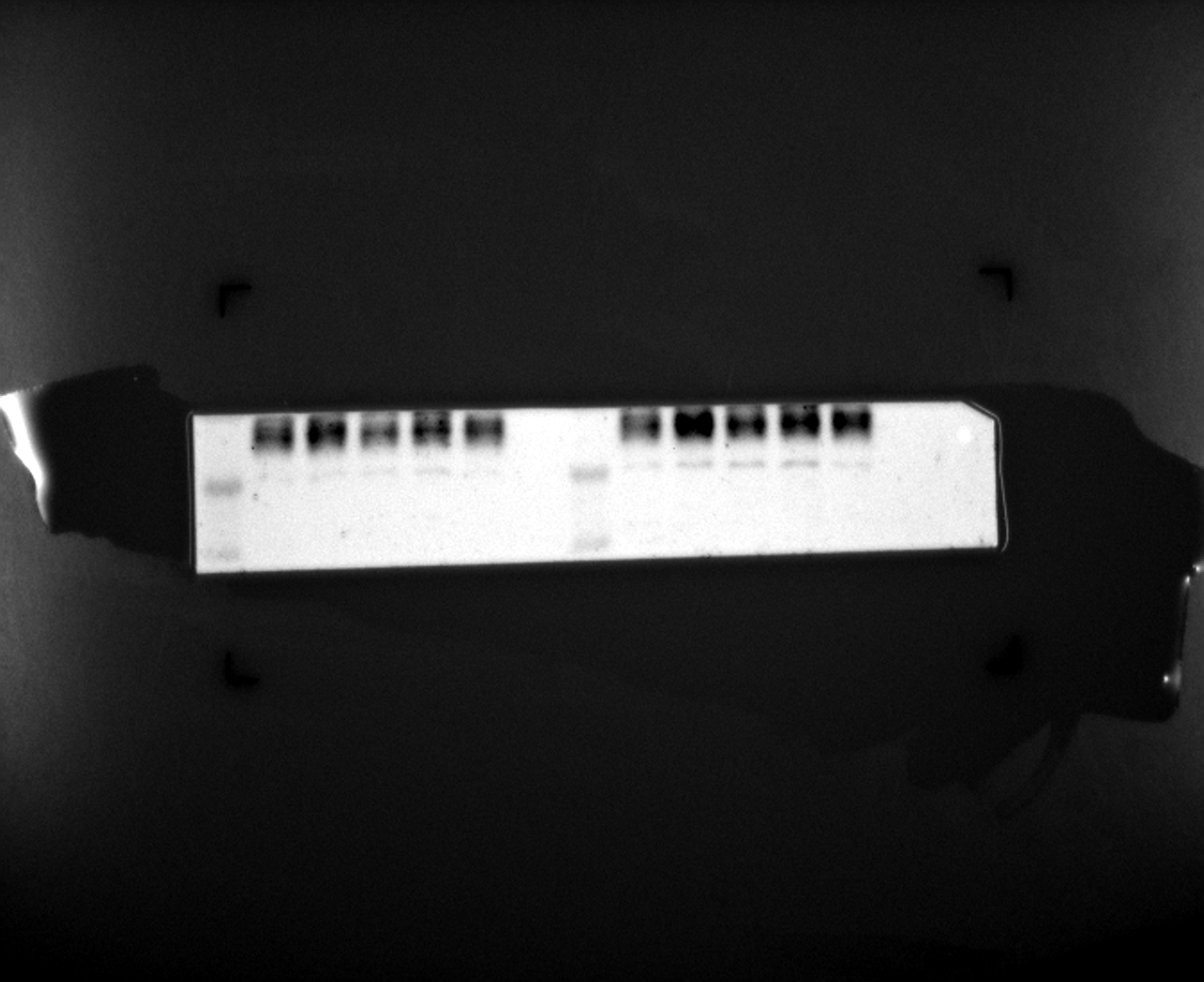

Supplement: Supplementary file 1 — Additional file 1. [file 13020_2025_1266_MOESM1_ESM.zip › Figure 7/bands/Cle-Caspase 3-3 R HC.Tif]

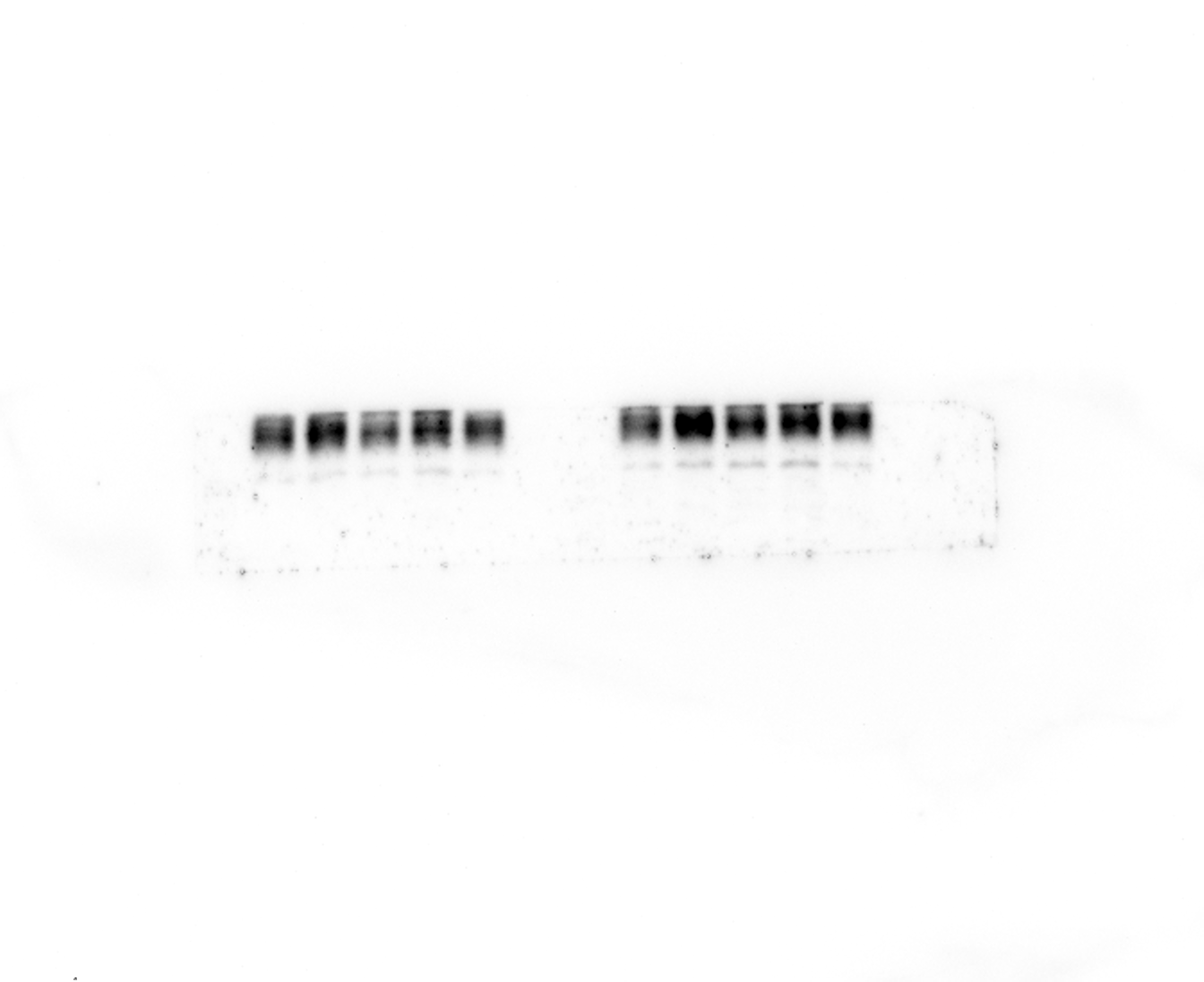

Supplement: Supplementary file 1 — Additional file 1. [file 13020_2025_1266_MOESM1_ESM.zip › Figure 7/bands/Cle-Caspase 3-3 R.Tif]

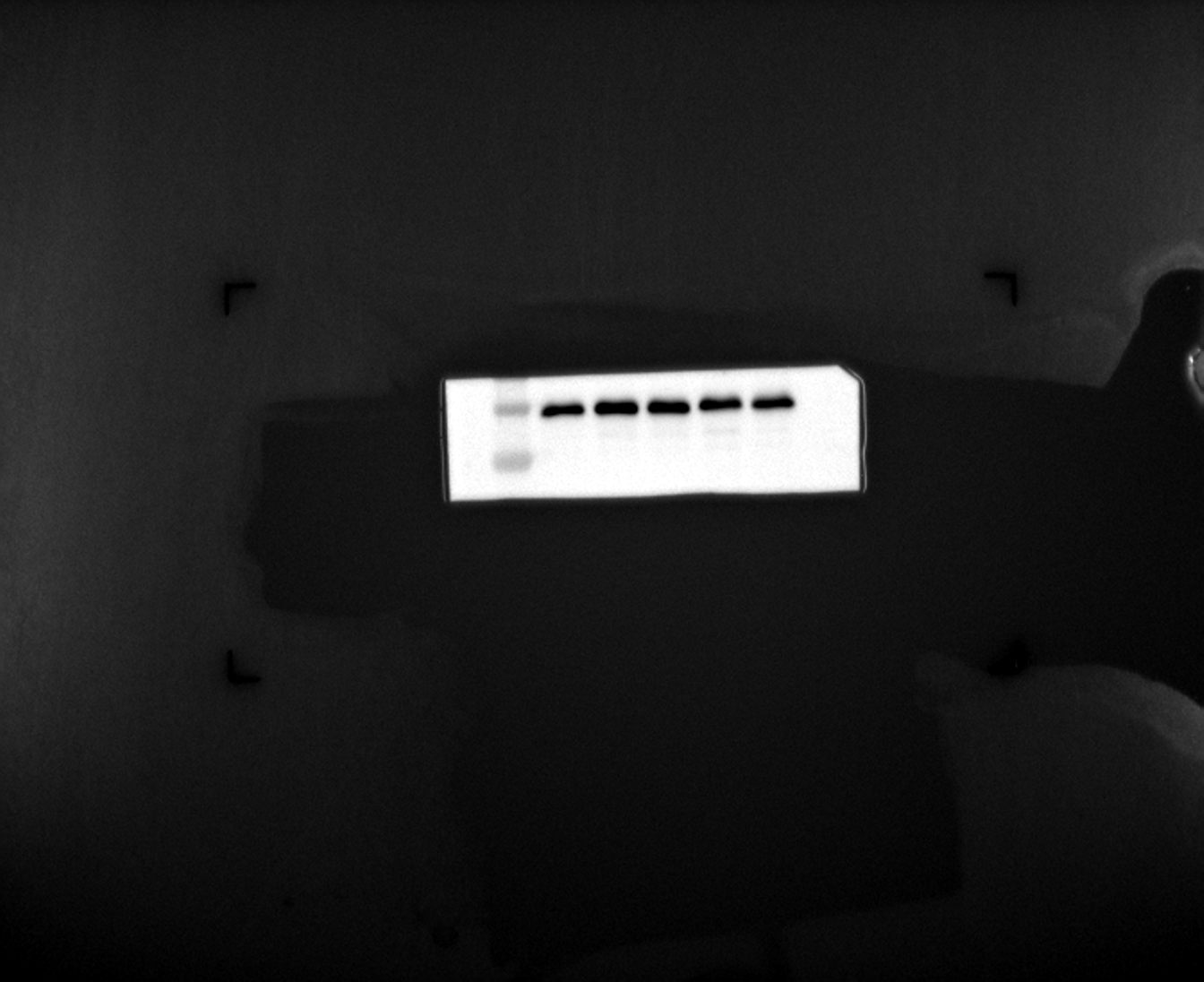

Supplement: Supplementary file 1 — Additional file 1. [file 13020_2025_1266_MOESM1_ESM.zip › Figure 7/bands/Cle-Caspase 8-1(Figure) HC.Tif]

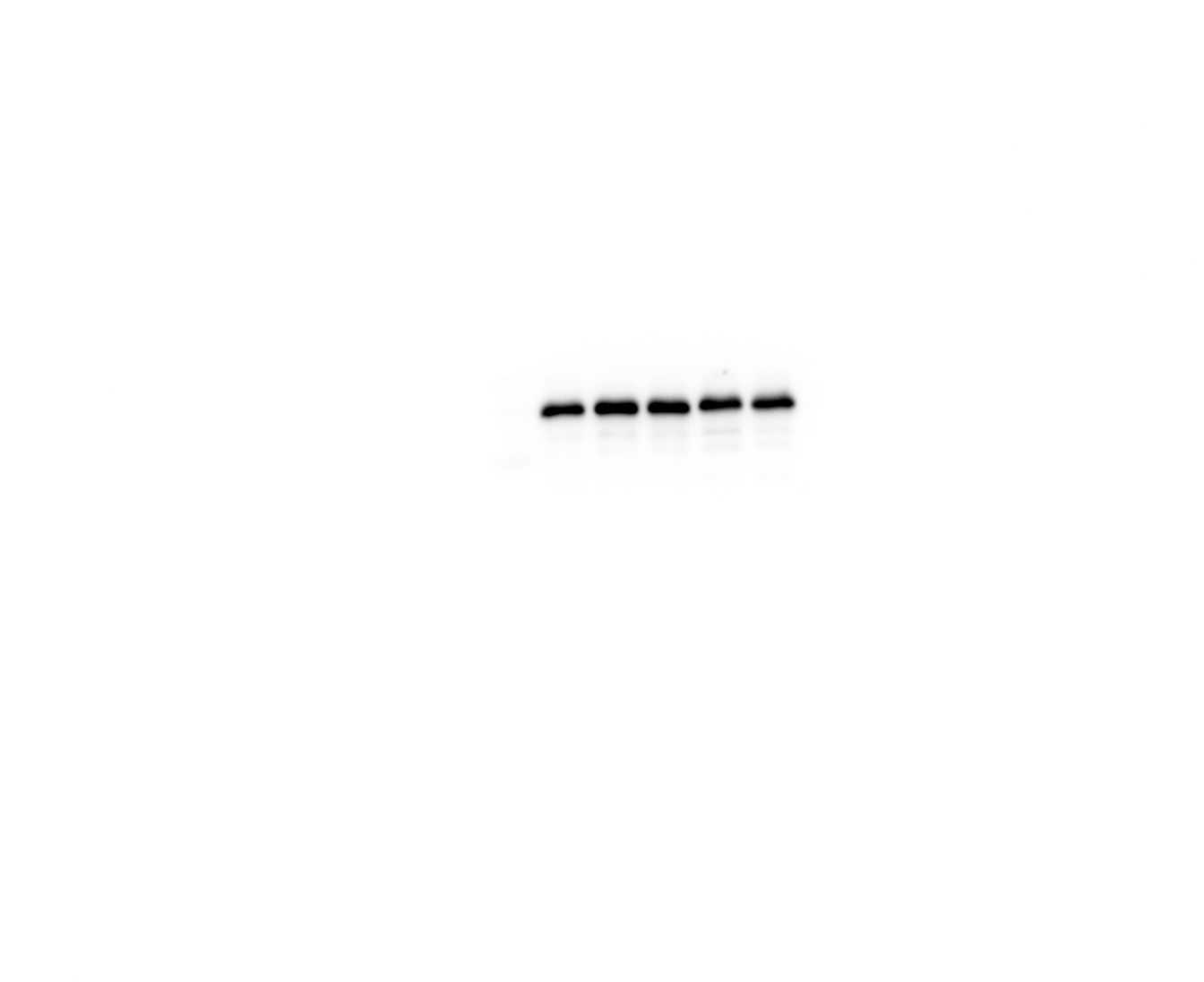

Supplement: Supplementary file 1 — Additional file 1. [file 13020_2025_1266_MOESM1_ESM.zip › Figure 7/bands/Cle-Caspase 8-1(Figure).Tif]

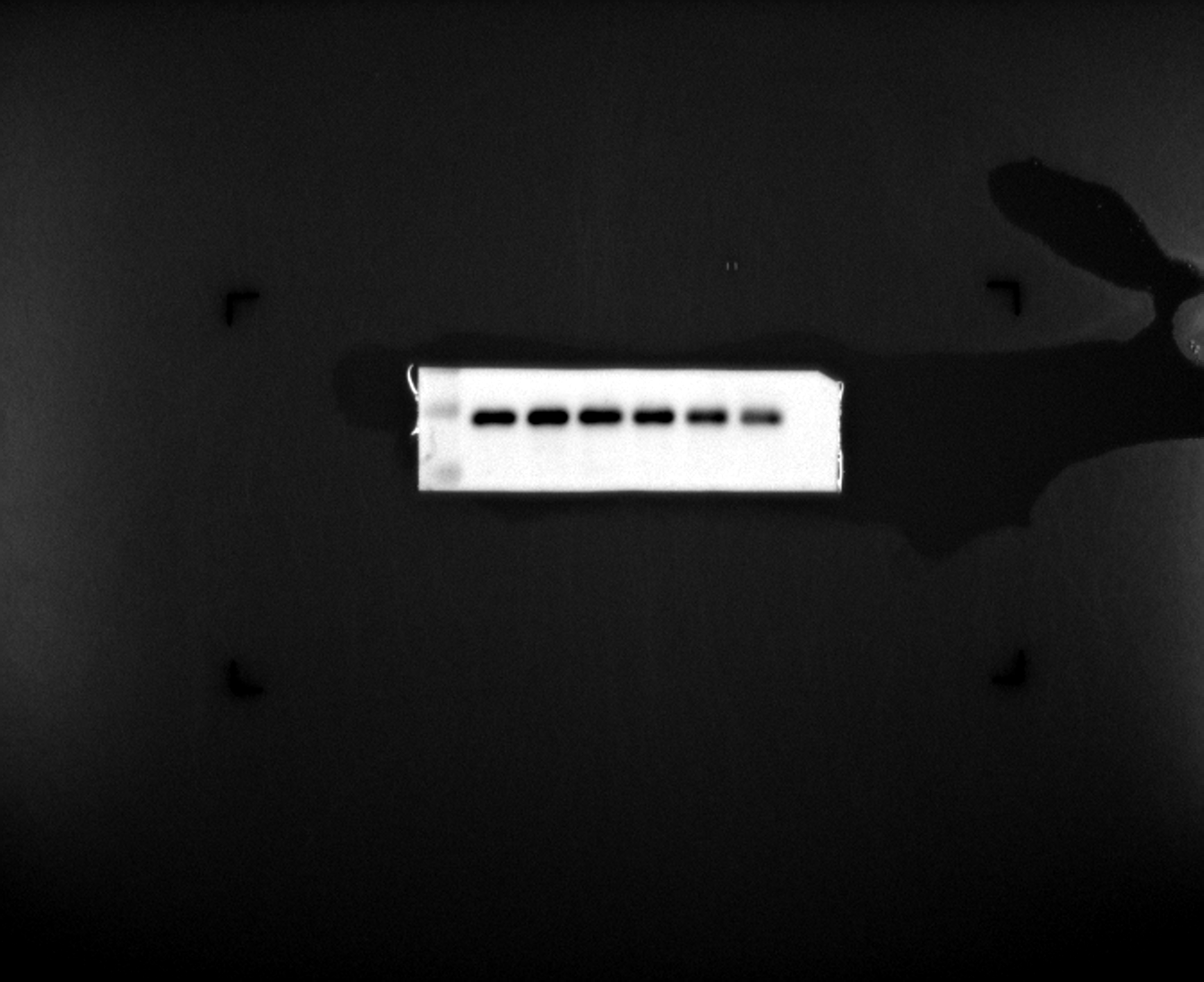

Supplement: Supplementary file 1 — Additional file 1. [file 13020_2025_1266_MOESM1_ESM.zip › Figure 7/bands/Cle-Caspase 8-2 HC.Tif]

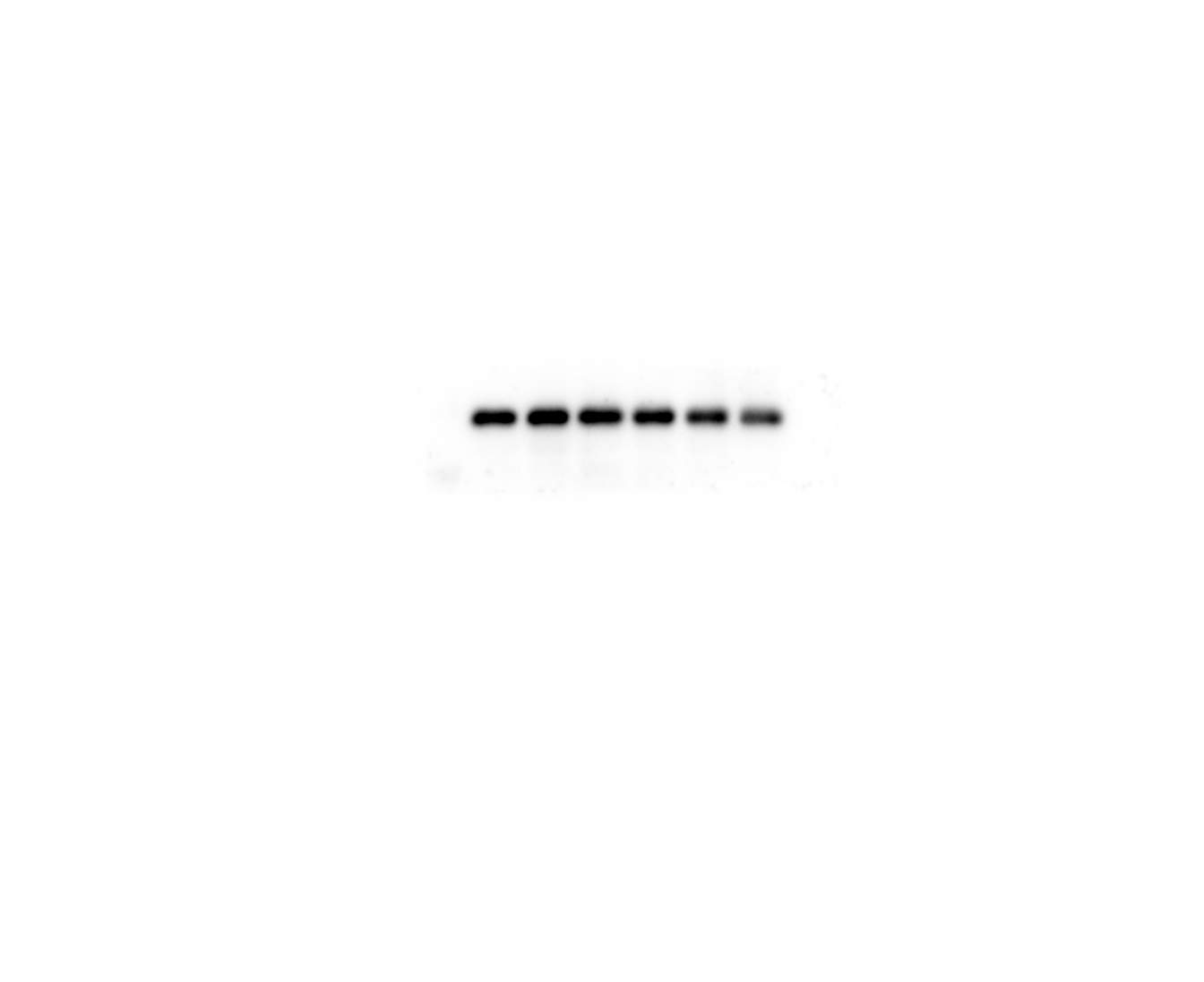

Supplement: Supplementary file 1 — Additional file 1. [file 13020_2025_1266_MOESM1_ESM.zip › Figure 7/bands/Cle-Caspase 8-2.Tif]

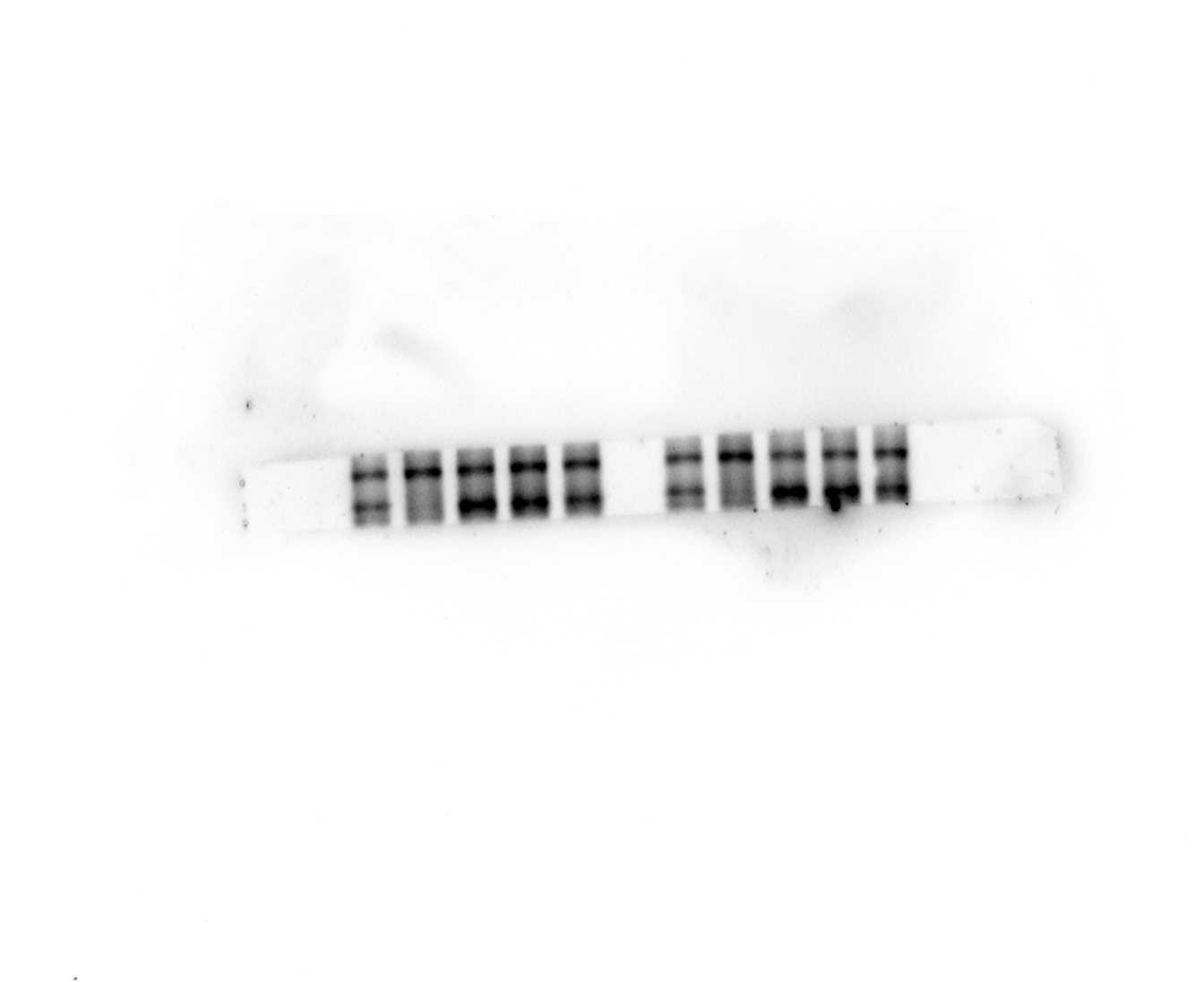

Supplement: Supplementary file 1 — Additional file 1. [file 13020_2025_1266_MOESM1_ESM.zip › Figure 7/bands/Cle-Caspase 8-3 L .Tif]

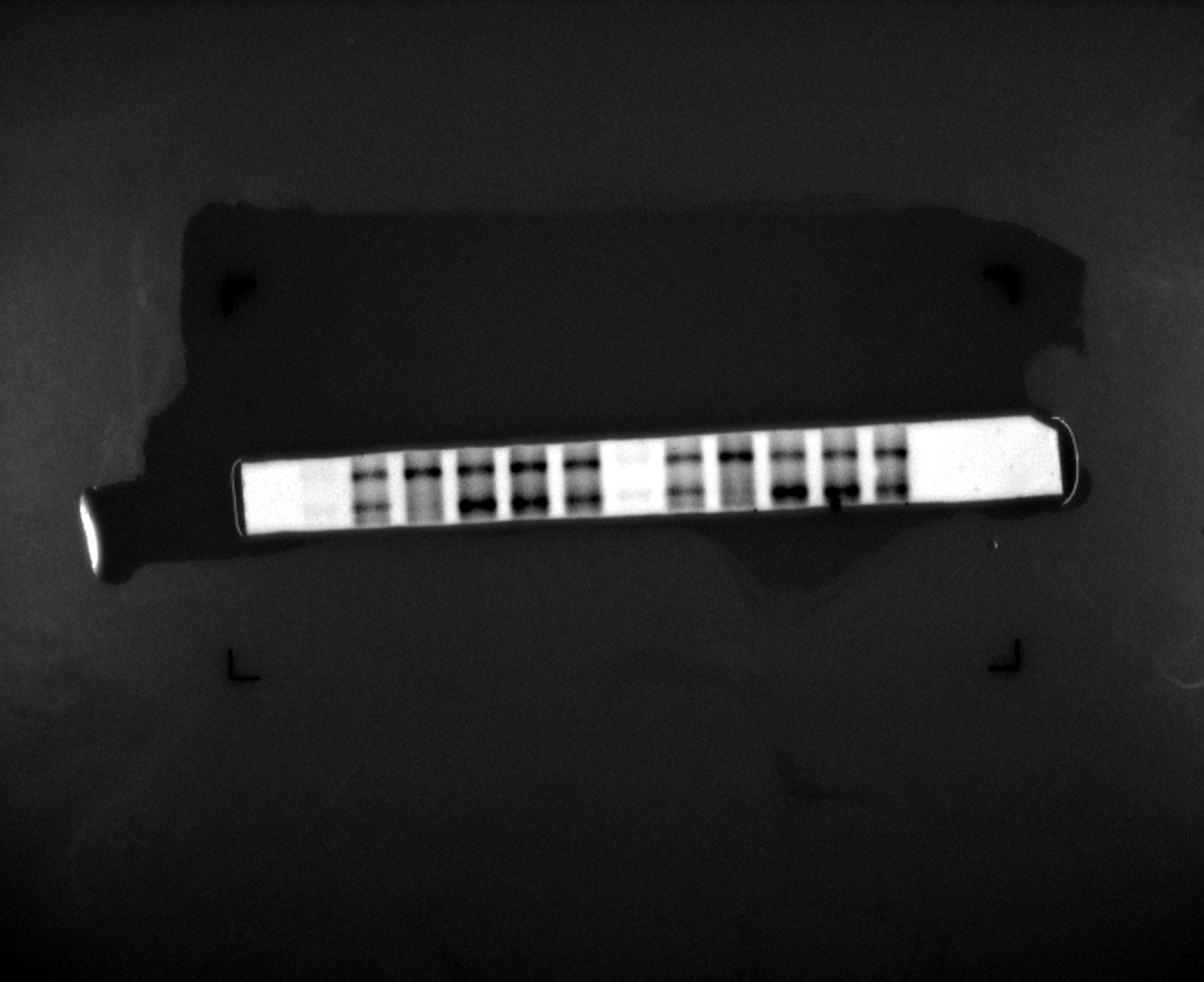

Supplement: Supplementary file 1 — Additional file 1. [file 13020_2025_1266_MOESM1_ESM.zip › Figure 7/bands/Cle-Caspase 8-3 L HC.Tif]

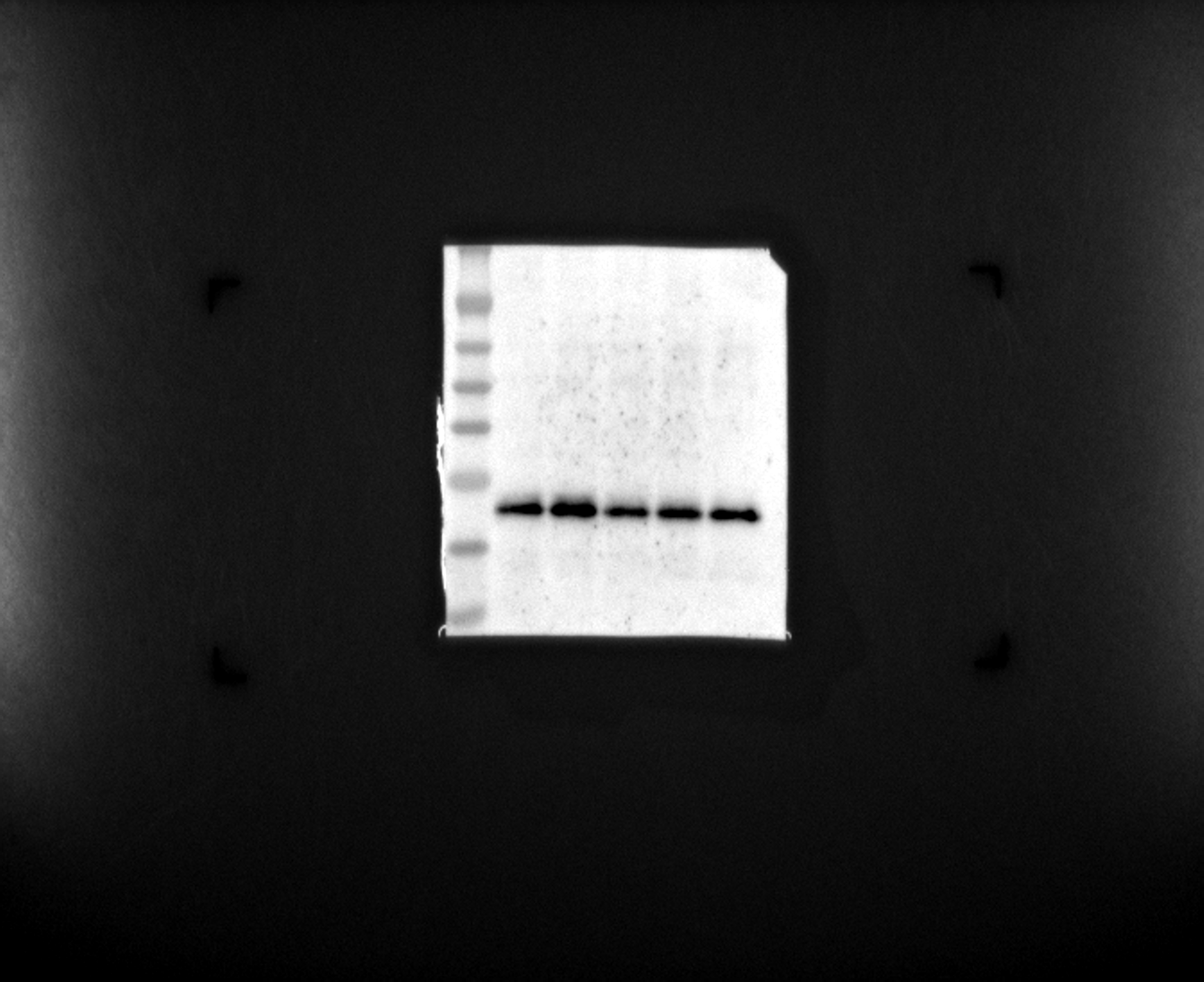

Supplement: Supplementary file 1 — Additional file 1. [file 13020_2025_1266_MOESM1_ESM.zip › Figure 7/bands/Fas-1(Figure) HC.Tif]

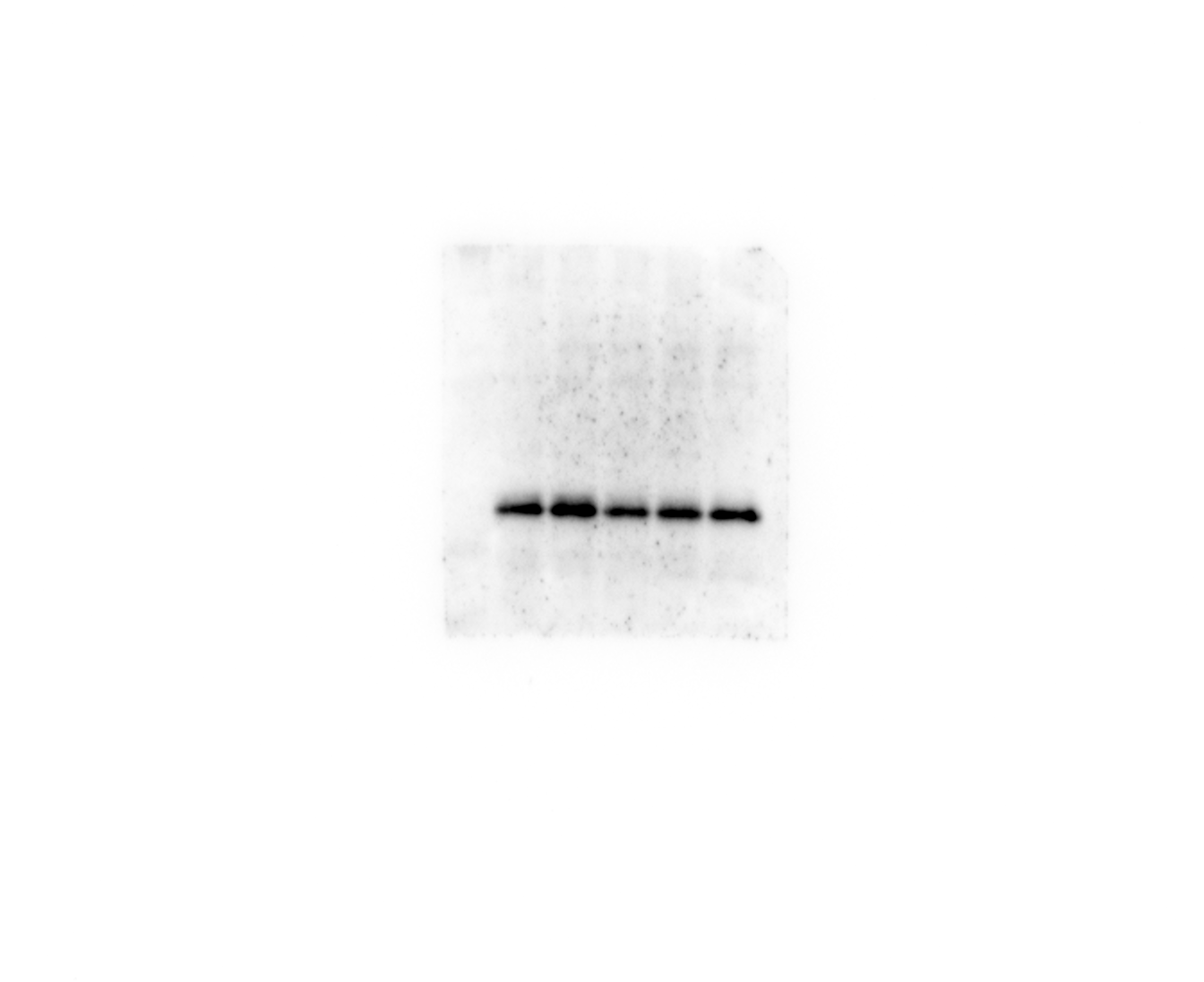

Supplement: Supplementary file 1 — Additional file 1. [file 13020_2025_1266_MOESM1_ESM.zip › Figure 7/bands/Fas-1(Figure).Tif]

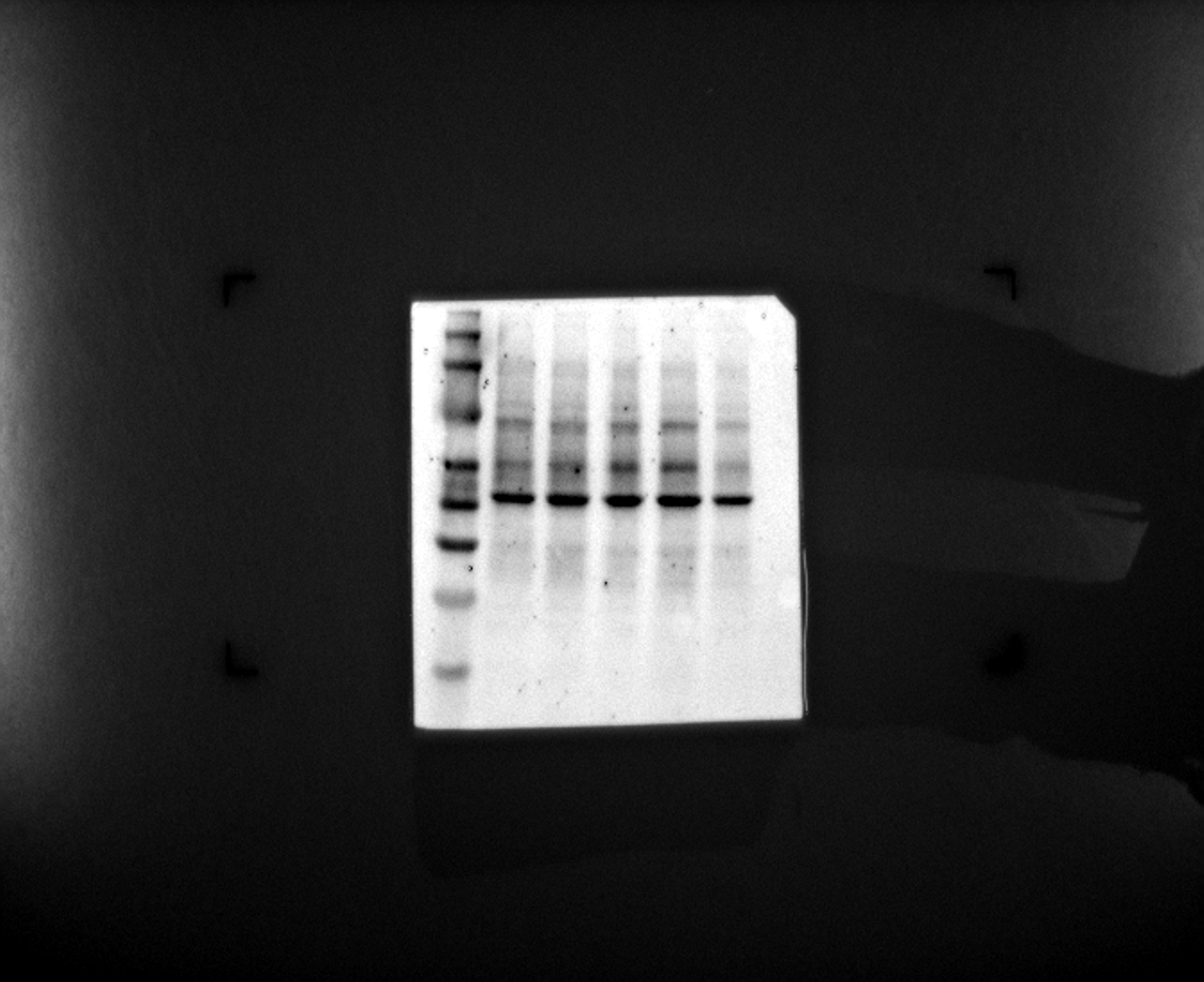

Supplement: Supplementary file 1 — Additional file 1. [file 13020_2025_1266_MOESM1_ESM.zip › Figure 7/bands/Fas-2 HC.Tif]

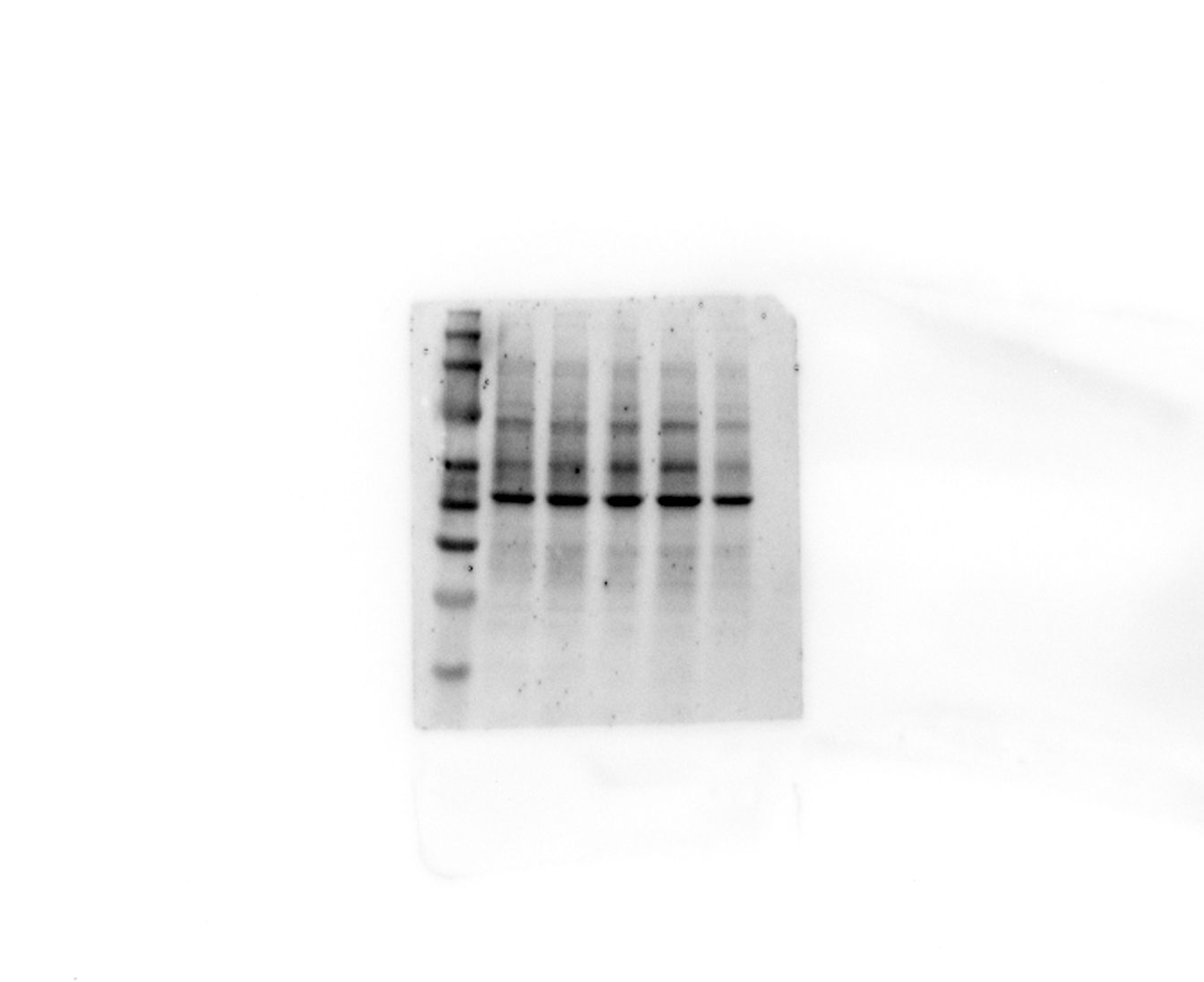

Supplement: Supplementary file 1 — Additional file 1. [file 13020_2025_1266_MOESM1_ESM.zip › Figure 7/bands/Fas-2.Tif]

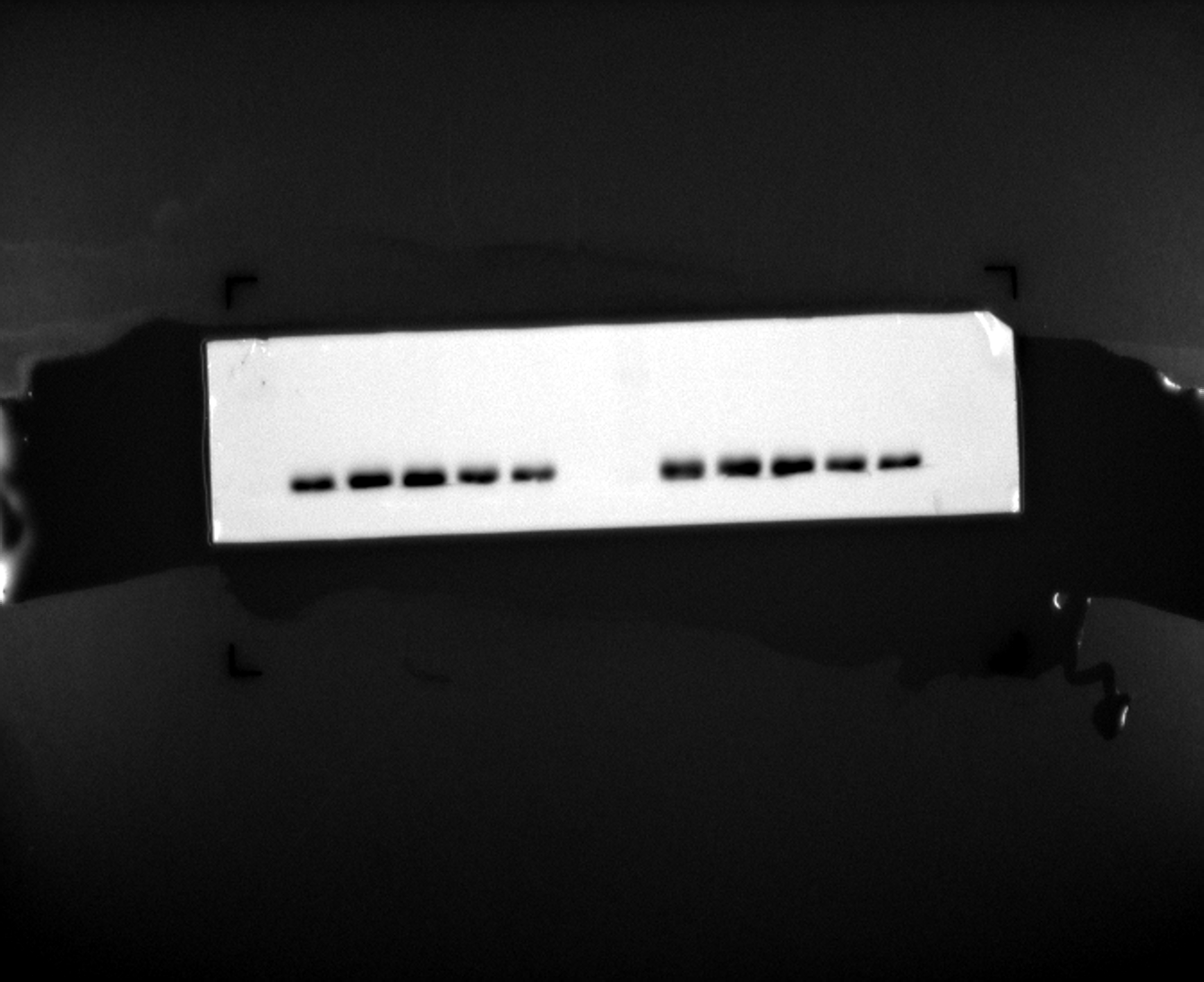

Supplement: Supplementary file 1 — Additional file 1. [file 13020_2025_1266_MOESM1_ESM.zip › Figure 7/bands/Fas-3 HC.Tif]

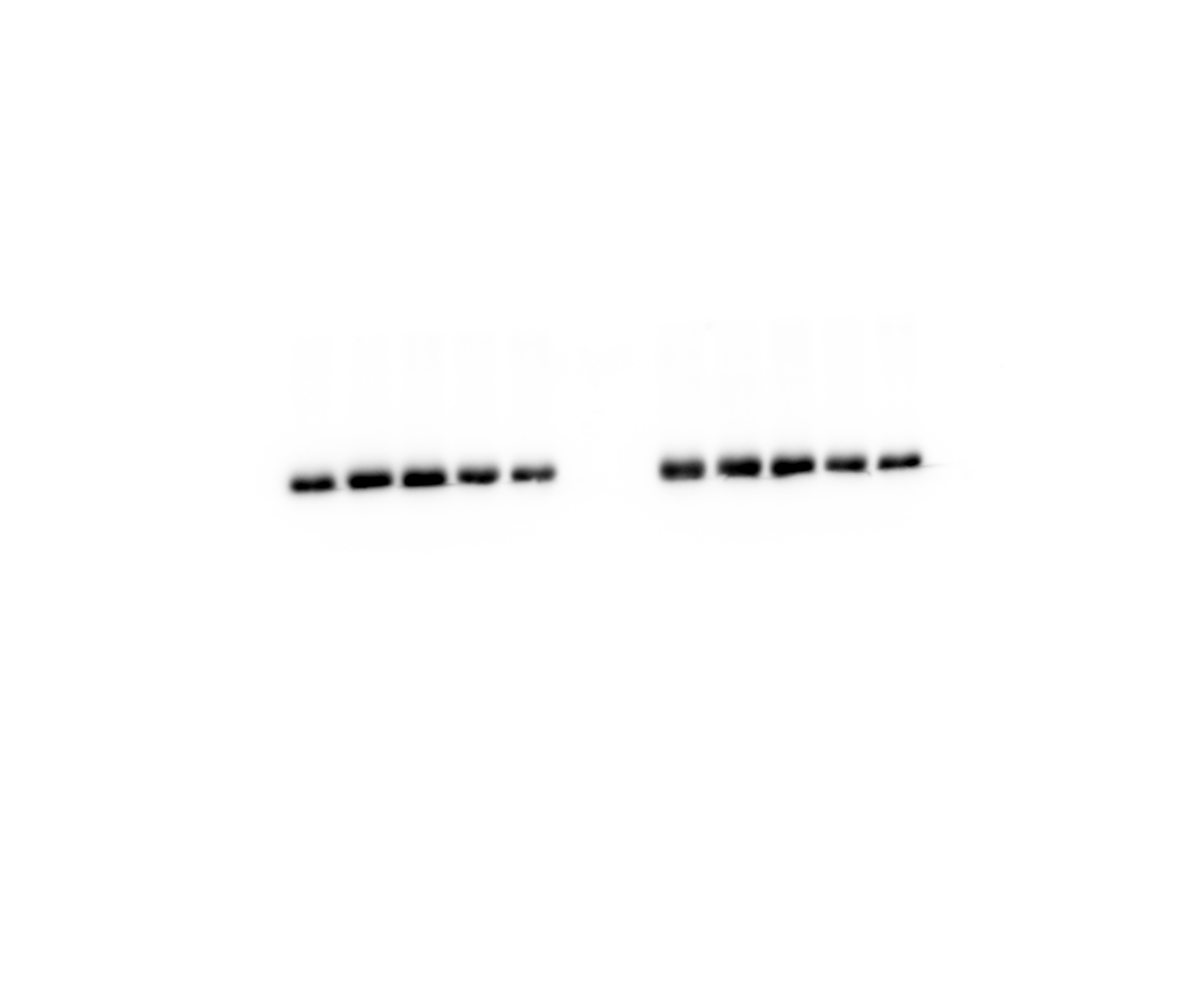

Supplement: Supplementary file 1 — Additional file 1. [file 13020_2025_1266_MOESM1_ESM.zip › Figure 7/bands/Fas-3.Tif]

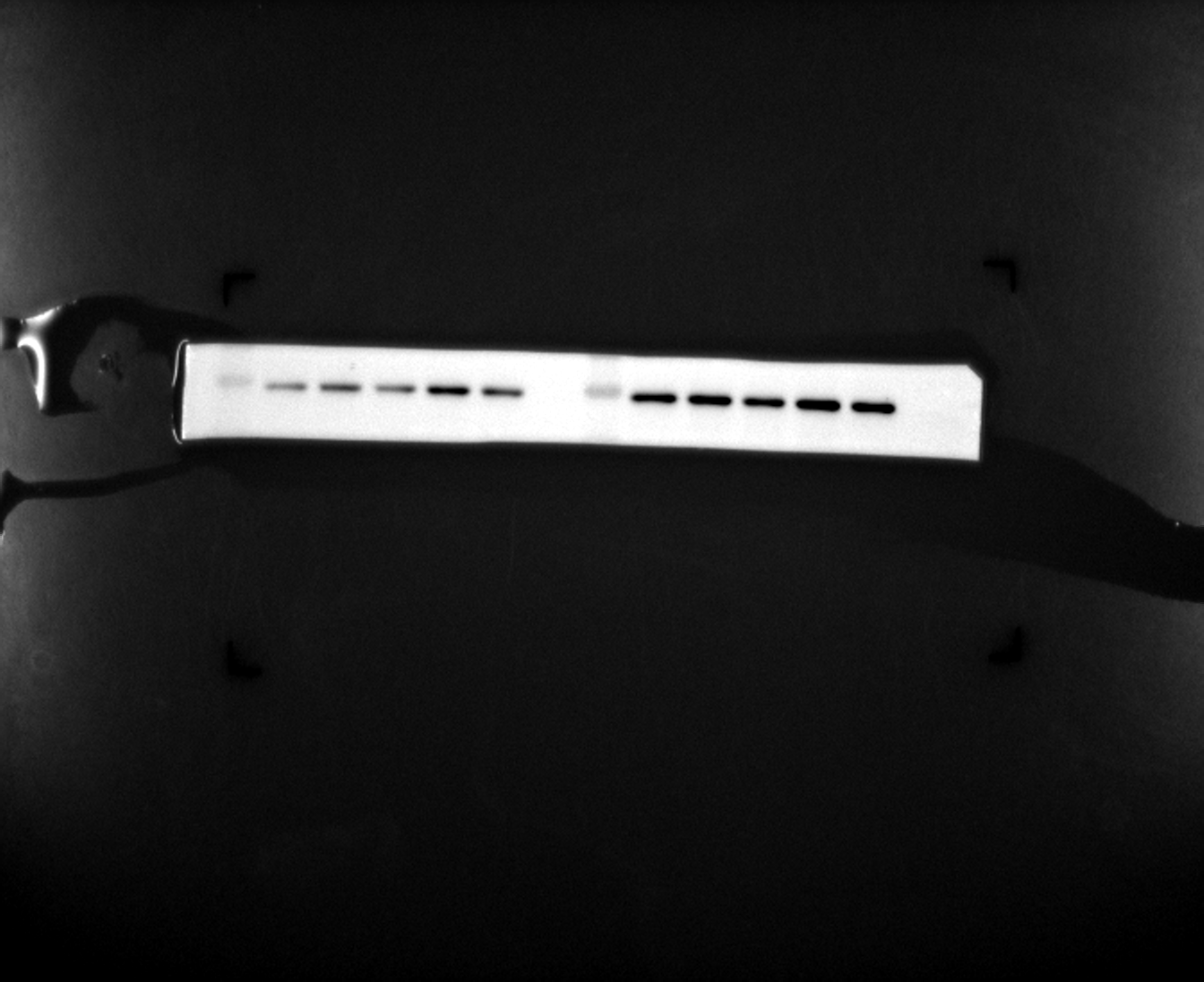

Supplement: Supplementary file 1 — Additional file 1. [file 13020_2025_1266_MOESM1_ESM.zip › Figure 7/bands/GAPDH HC-3.Tif]

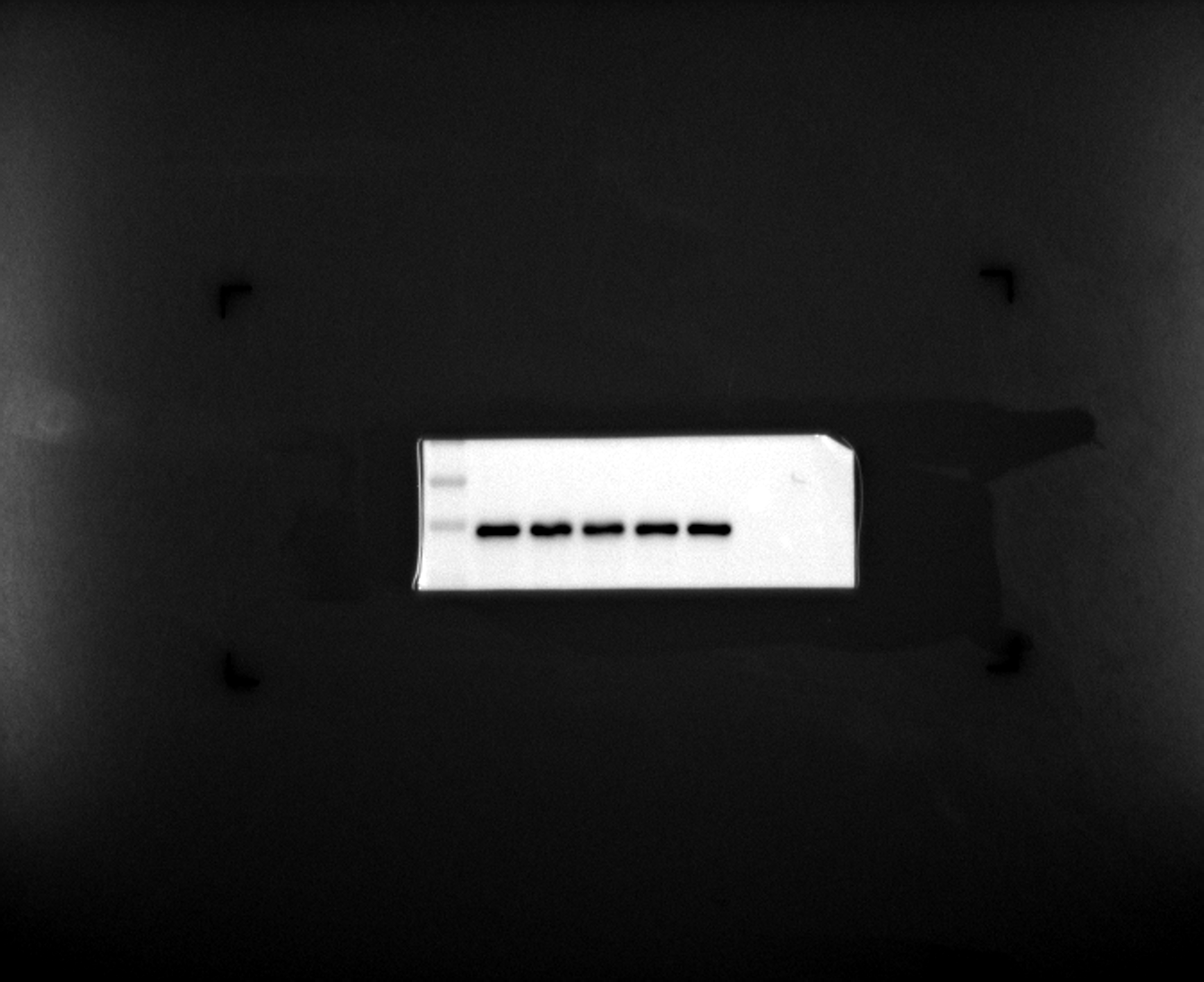

Supplement: Supplementary file 1 — Additional file 1. [file 13020_2025_1266_MOESM1_ESM.zip › Figure 7/bands/GAPDH-1 HC.Tif]

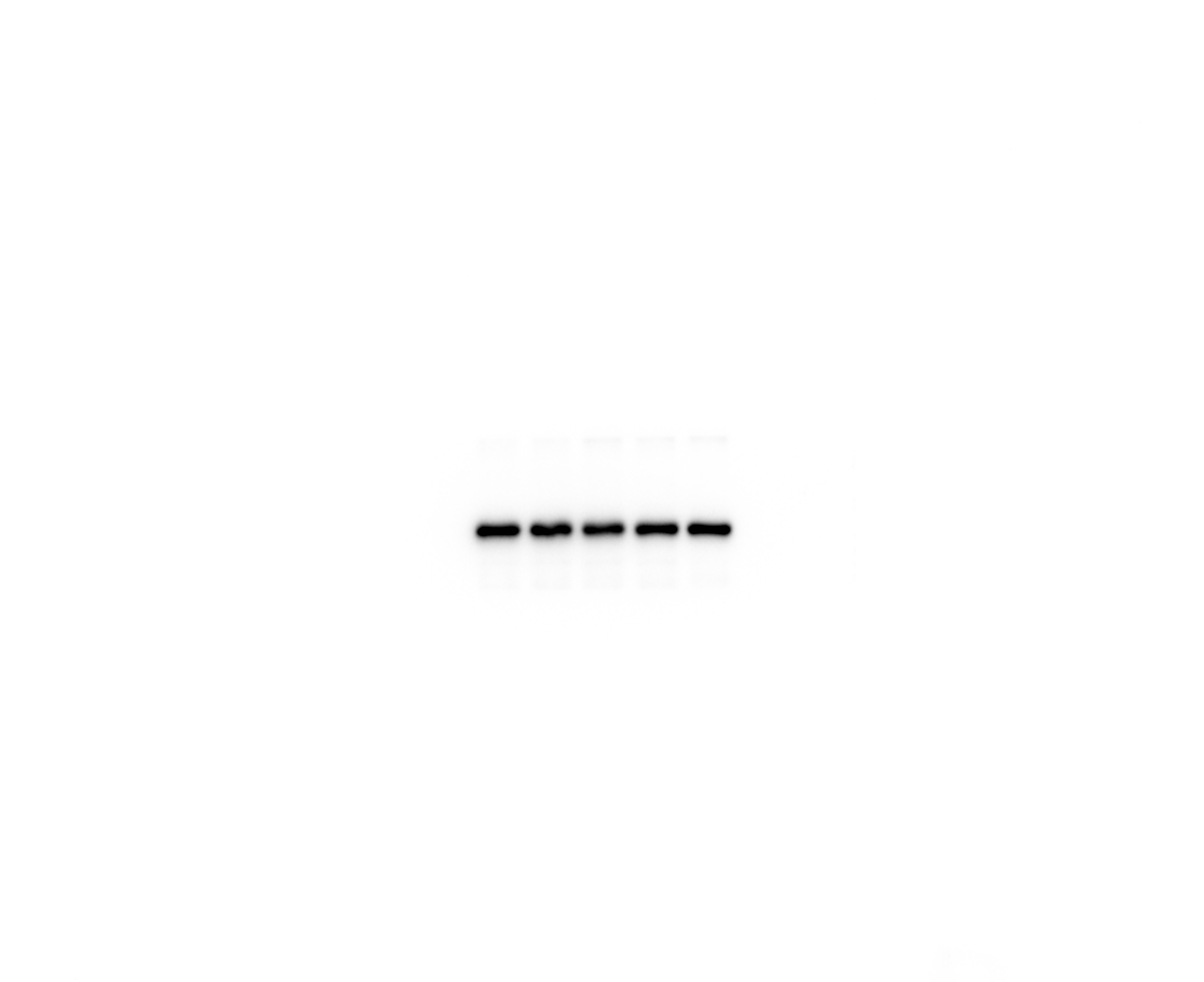

Supplement: Supplementary file 1 — Additional file 1. [file 13020_2025_1266_MOESM1_ESM.zip › Figure 7/bands/GAPDH-1.Tif]

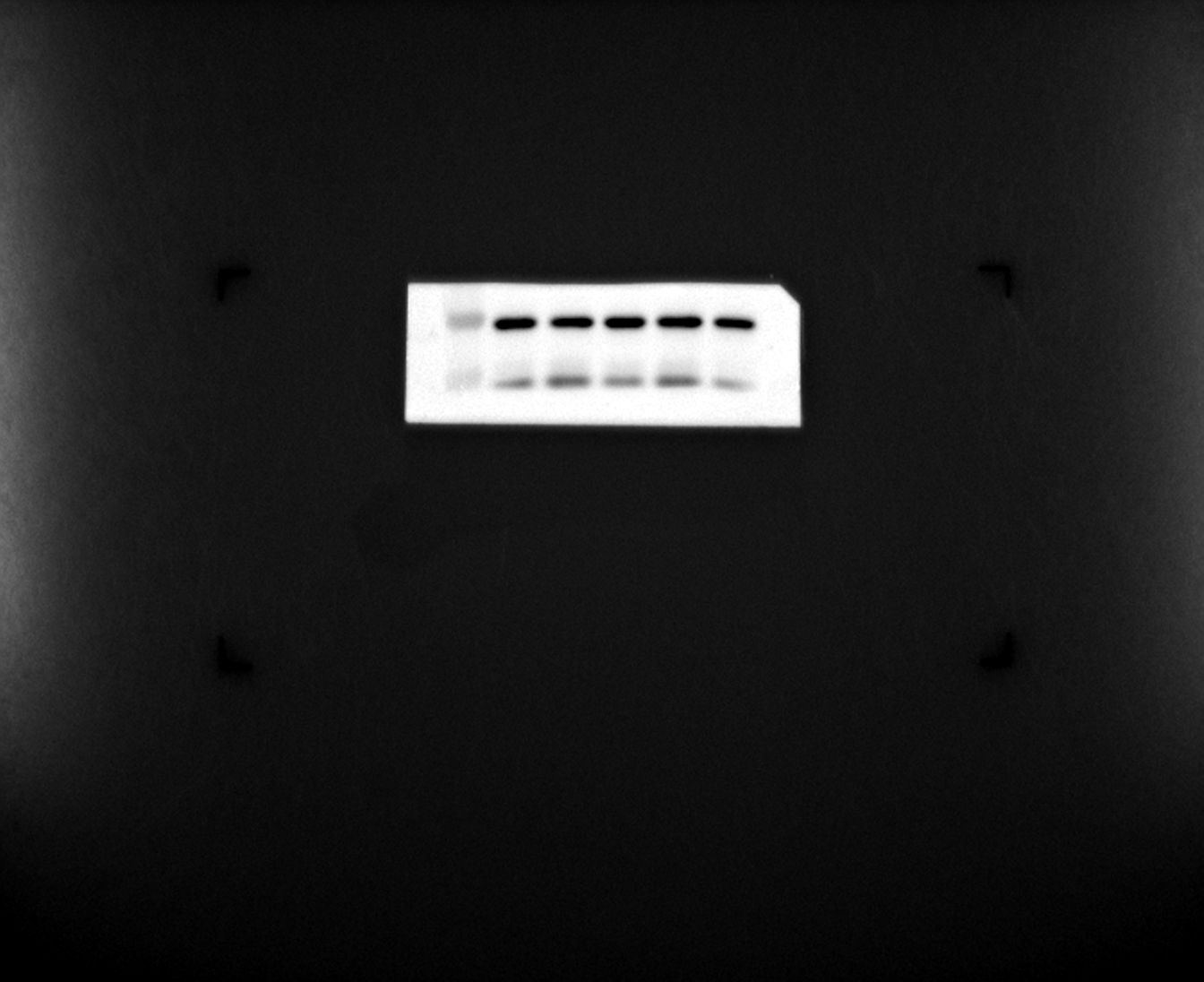

Supplement: Supplementary file 1 — Additional file 1. [file 13020_2025_1266_MOESM1_ESM.zip › Figure 7/bands/GAPDH-2 HC.Tif]

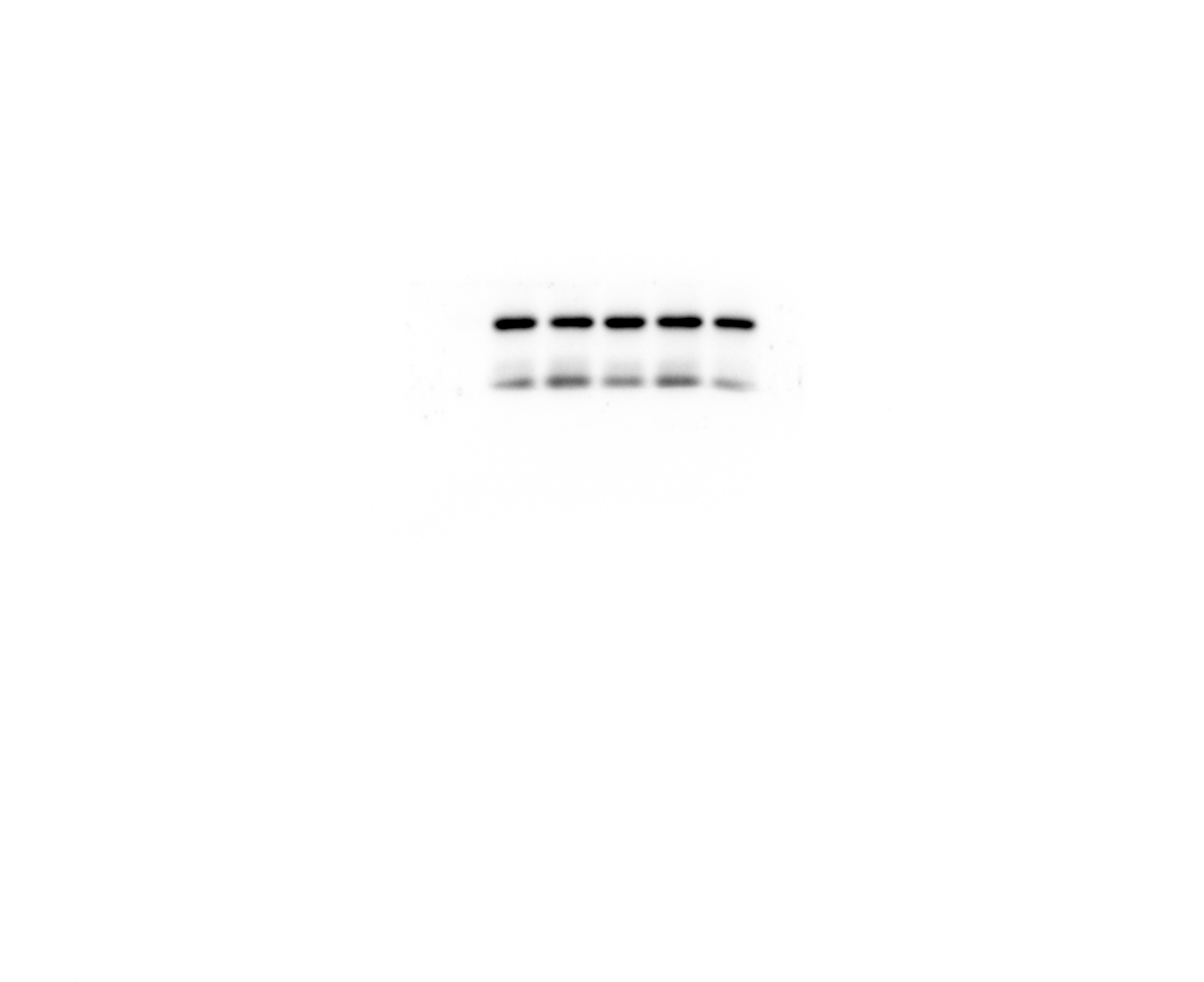

Supplement: Supplementary file 1 — Additional file 1. [file 13020_2025_1266_MOESM1_ESM.zip › Figure 7/bands/GAPDH-2.Tif]

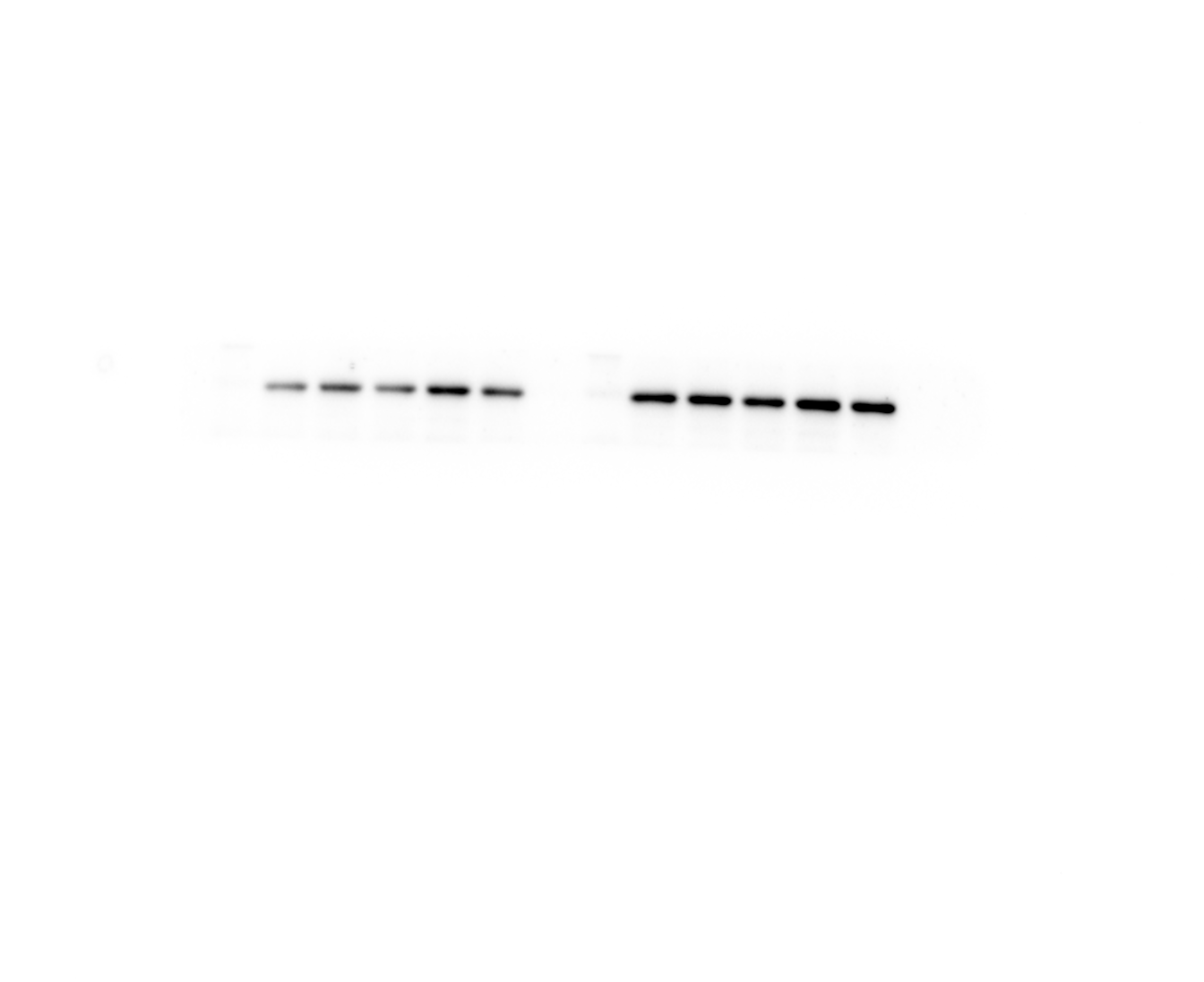

Supplement: Supplementary file 1 — Additional file 1. [file 13020_2025_1266_MOESM1_ESM.zip › Figure 7/bands/GAPDH-3.Tif]

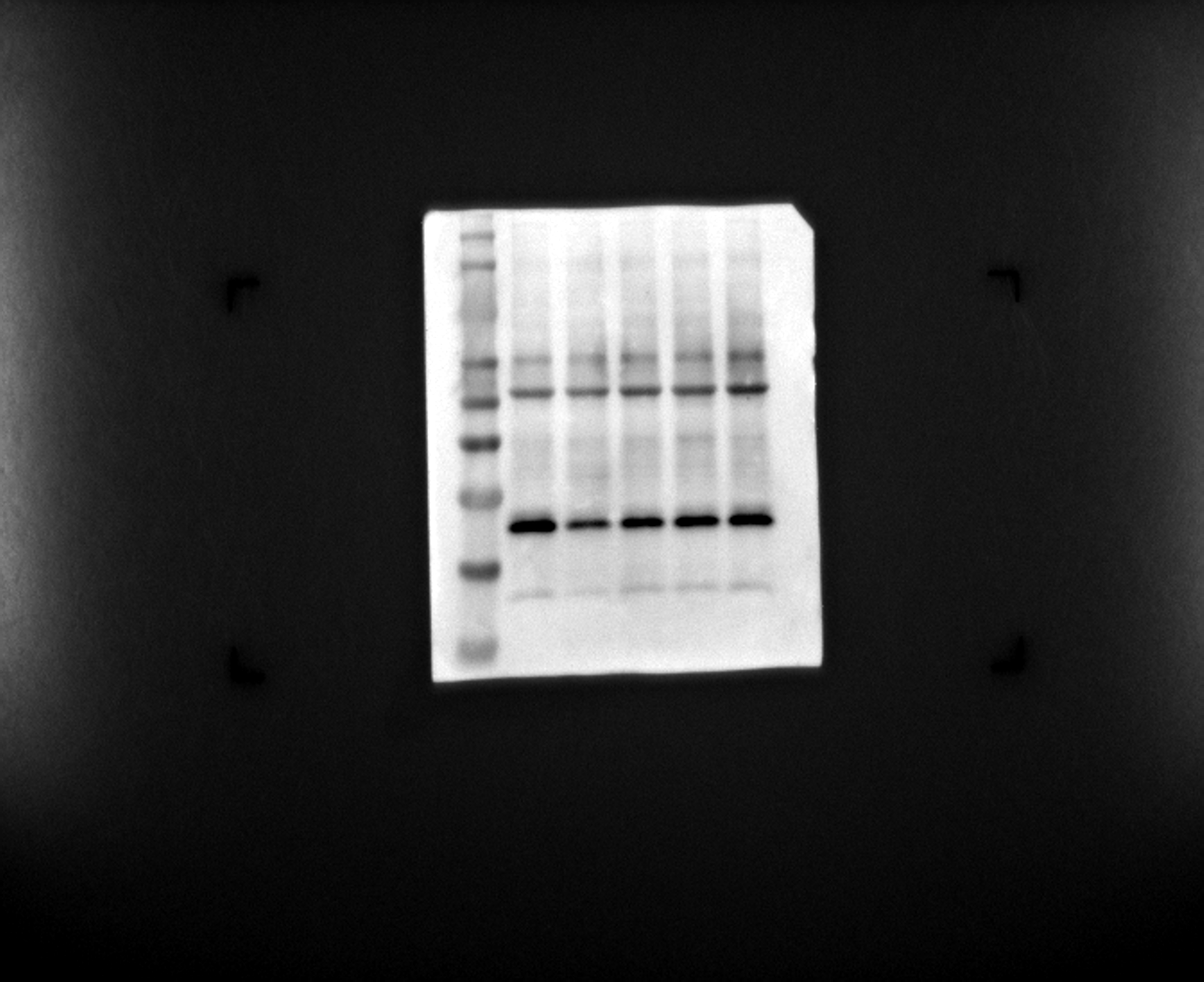

Supplement: Supplementary file 1 — Additional file 1. [file 13020_2025_1266_MOESM1_ESM.zip › Figure 7/bands/p-BCL-2 1(Figure) HC.Tif]

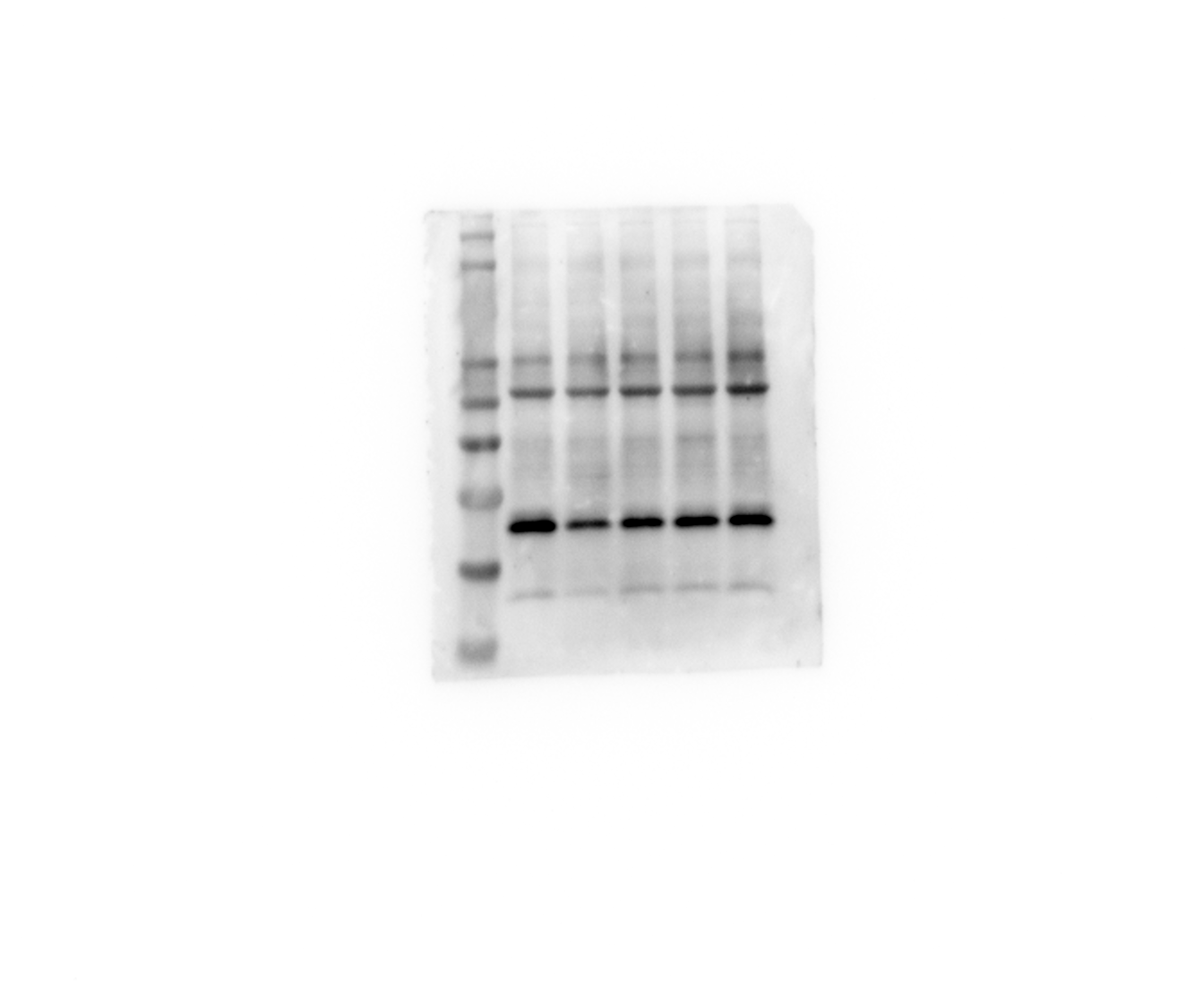

Supplement: Supplementary file 1 — Additional file 1. [file 13020_2025_1266_MOESM1_ESM.zip › Figure 7/bands/p-BCL-2 1(Figure).Tif]

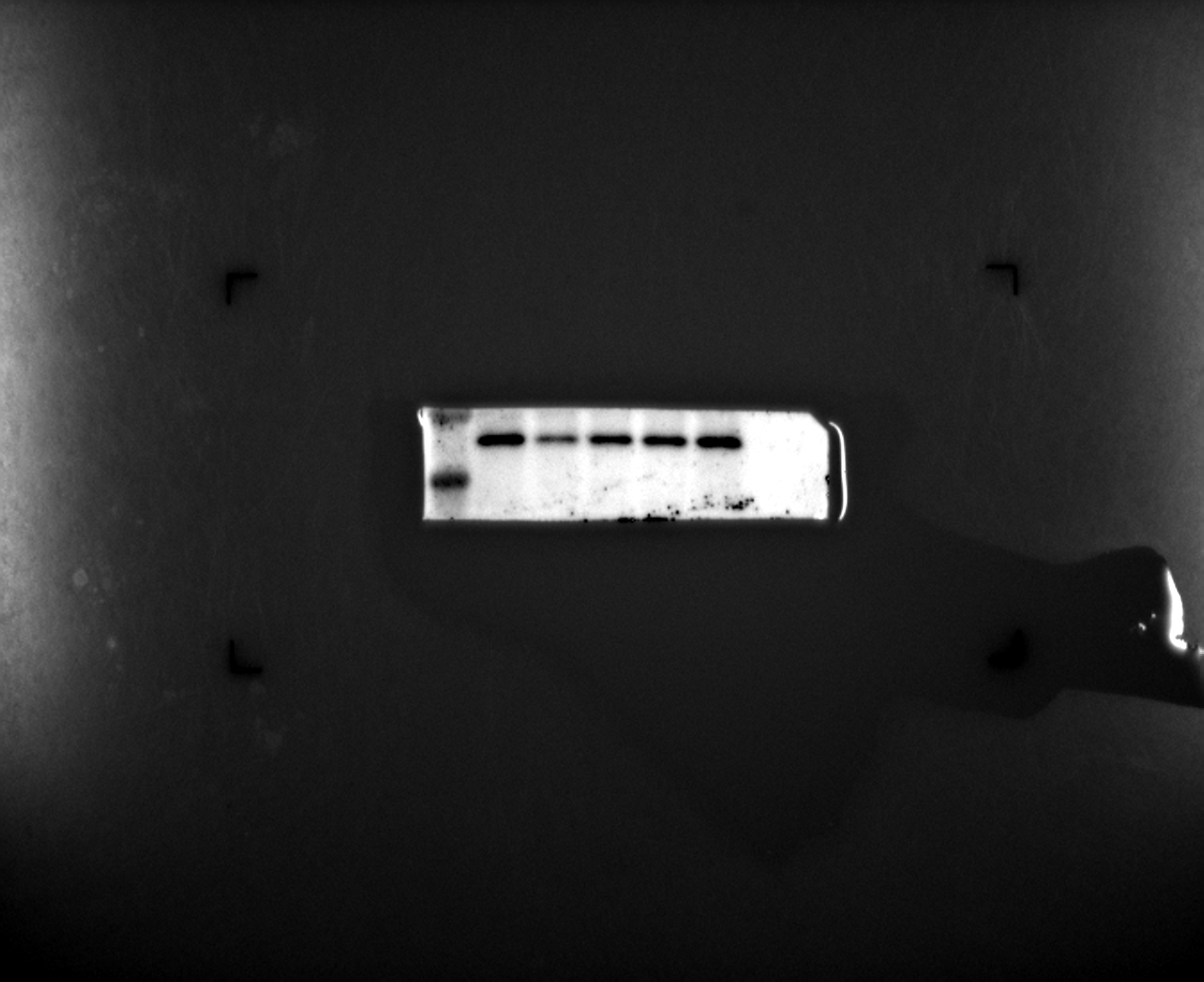

Supplement: Supplementary file 1 — Additional file 1. [file 13020_2025_1266_MOESM1_ESM.zip › Figure 7/bands/p-BCL-2 2 HC.Tif]

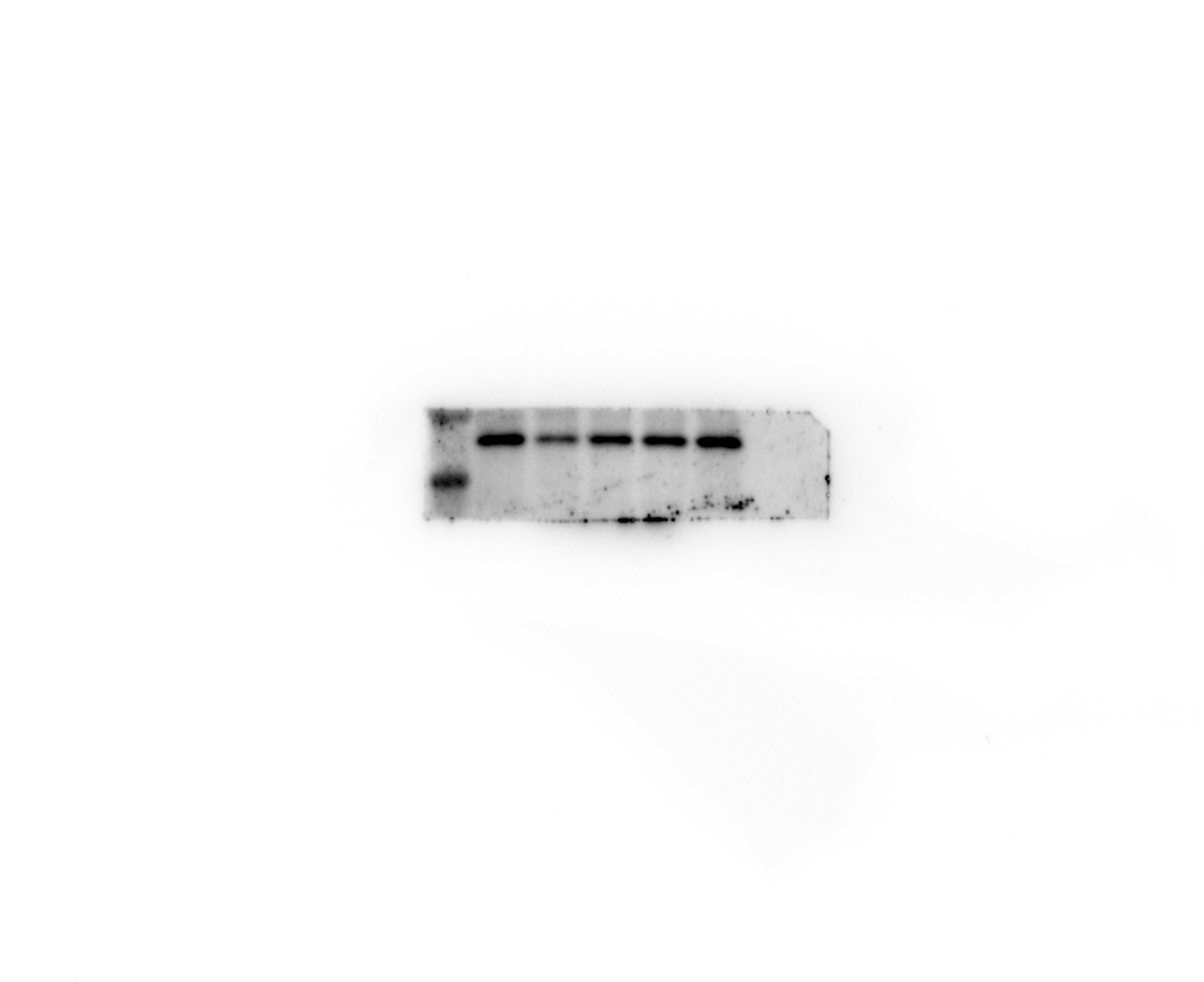

Supplement: Supplementary file 1 — Additional file 1. [file 13020_2025_1266_MOESM1_ESM.zip › Figure 7/bands/p-BCL-2 2.Tif]

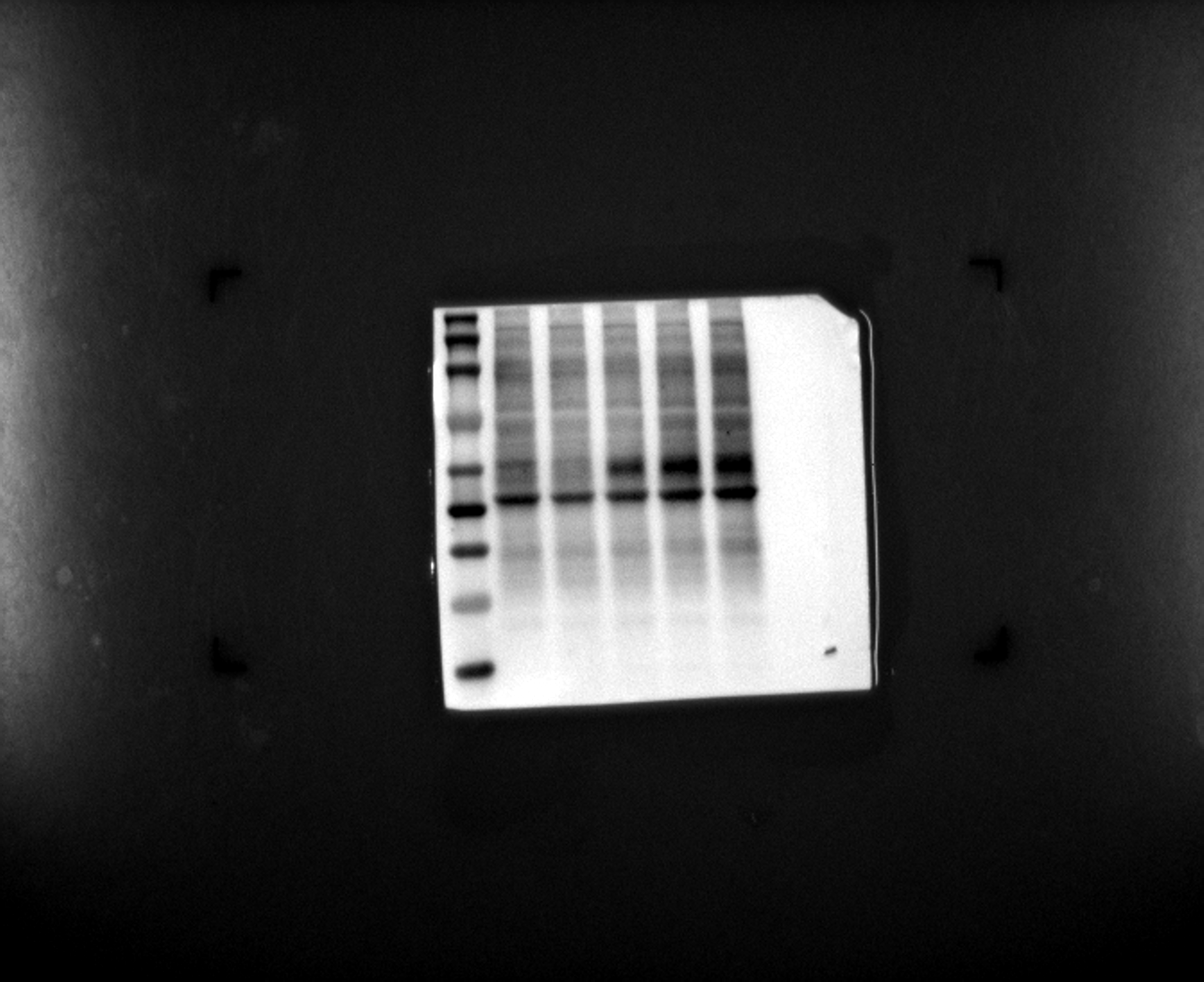

Supplement: Supplementary file 1 — Additional file 1. [file 13020_2025_1266_MOESM1_ESM.zip › Figure 7/bands/p-BCL-2 3 HC.Tif]

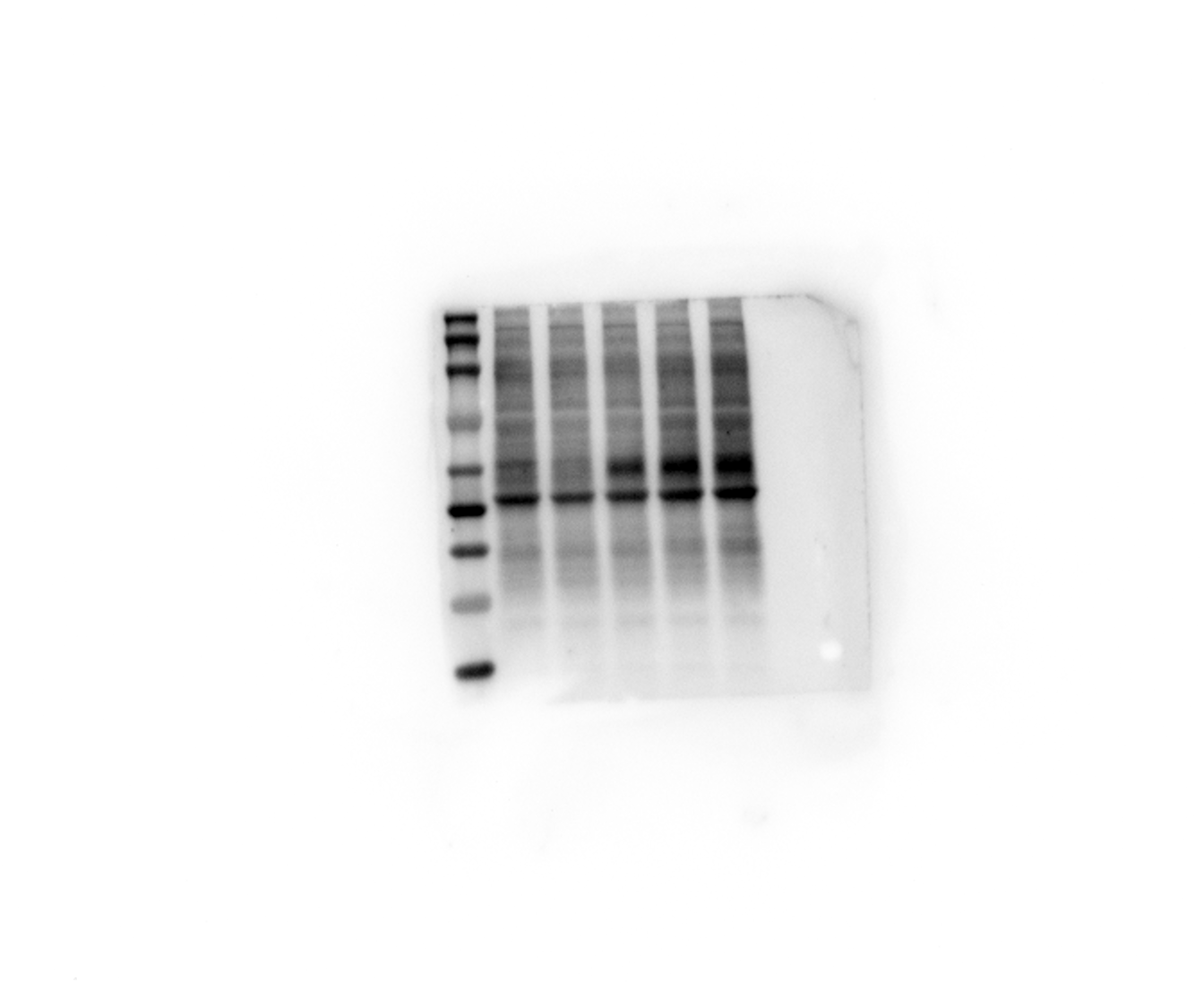

Supplement: Supplementary file 1 — Additional file 1. [file 13020_2025_1266_MOESM1_ESM.zip › Figure 7/bands/p-BCL-2 3.Tif]

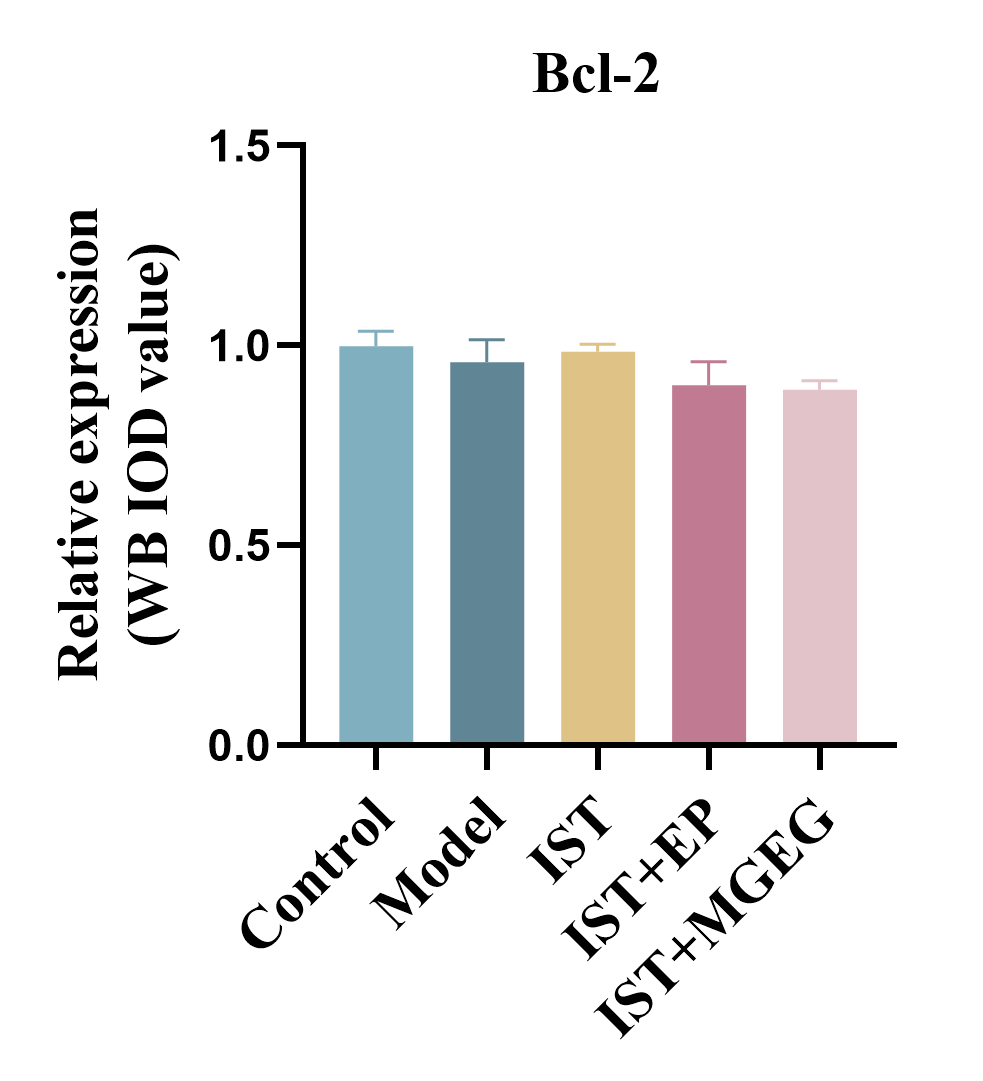

Supplement: Supplementary file 1 — Additional file 1. [file 13020_2025_1266_MOESM1_ESM.zip › Figure 7/Bcl-2.tif]

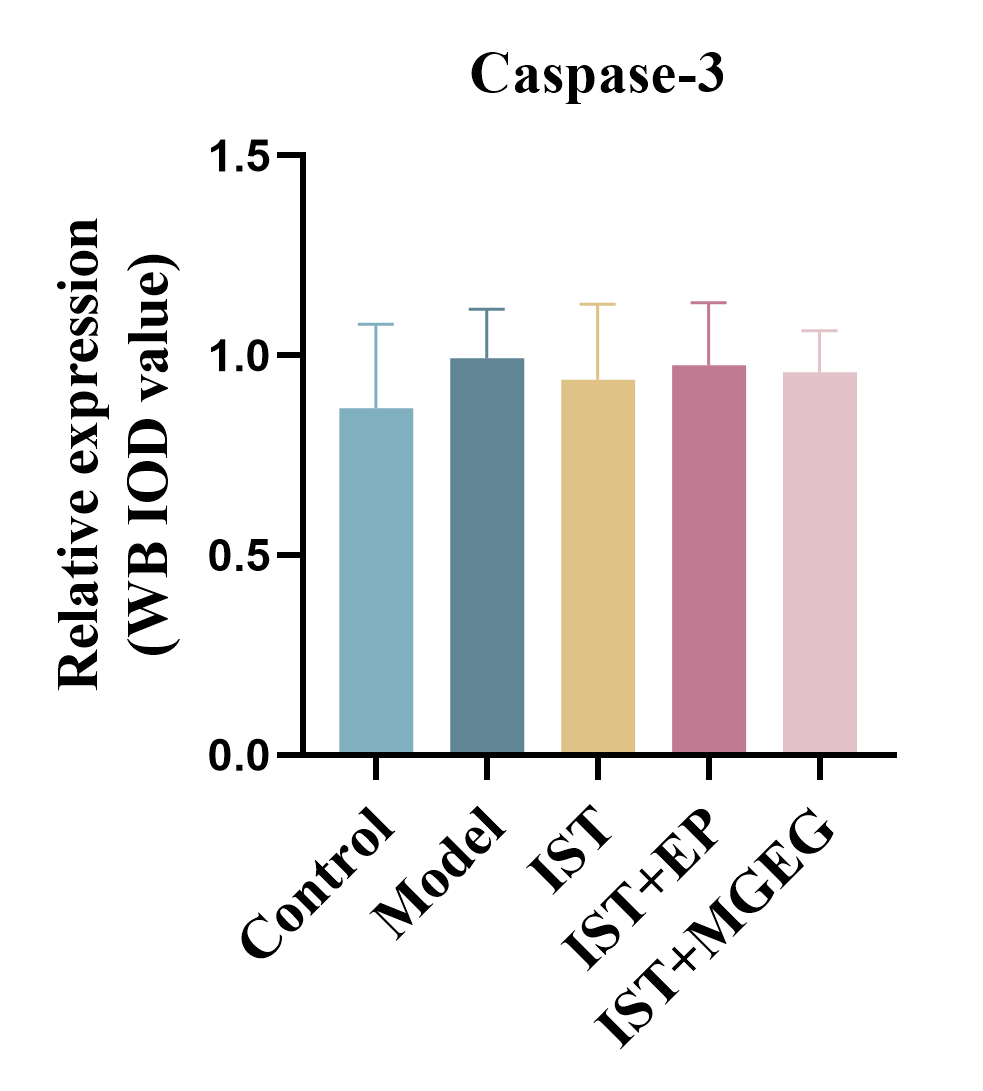

Supplement: Supplementary file 1 — Additional file 1. [file 13020_2025_1266_MOESM1_ESM.zip › Figure 7/Cas3.tif]

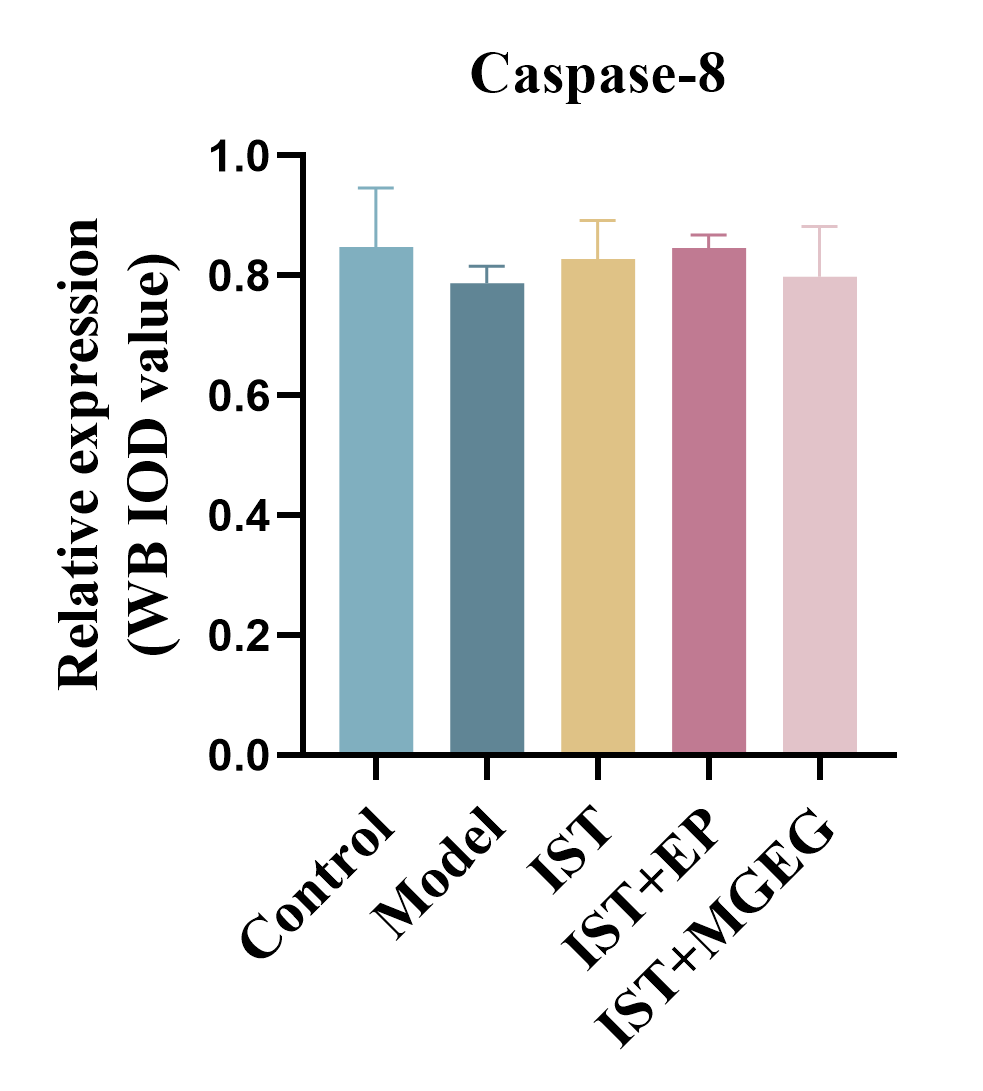

Supplement: Supplementary file 1 — Additional file 1. [file 13020_2025_1266_MOESM1_ESM.zip › Figure 7/Cas8.tif]

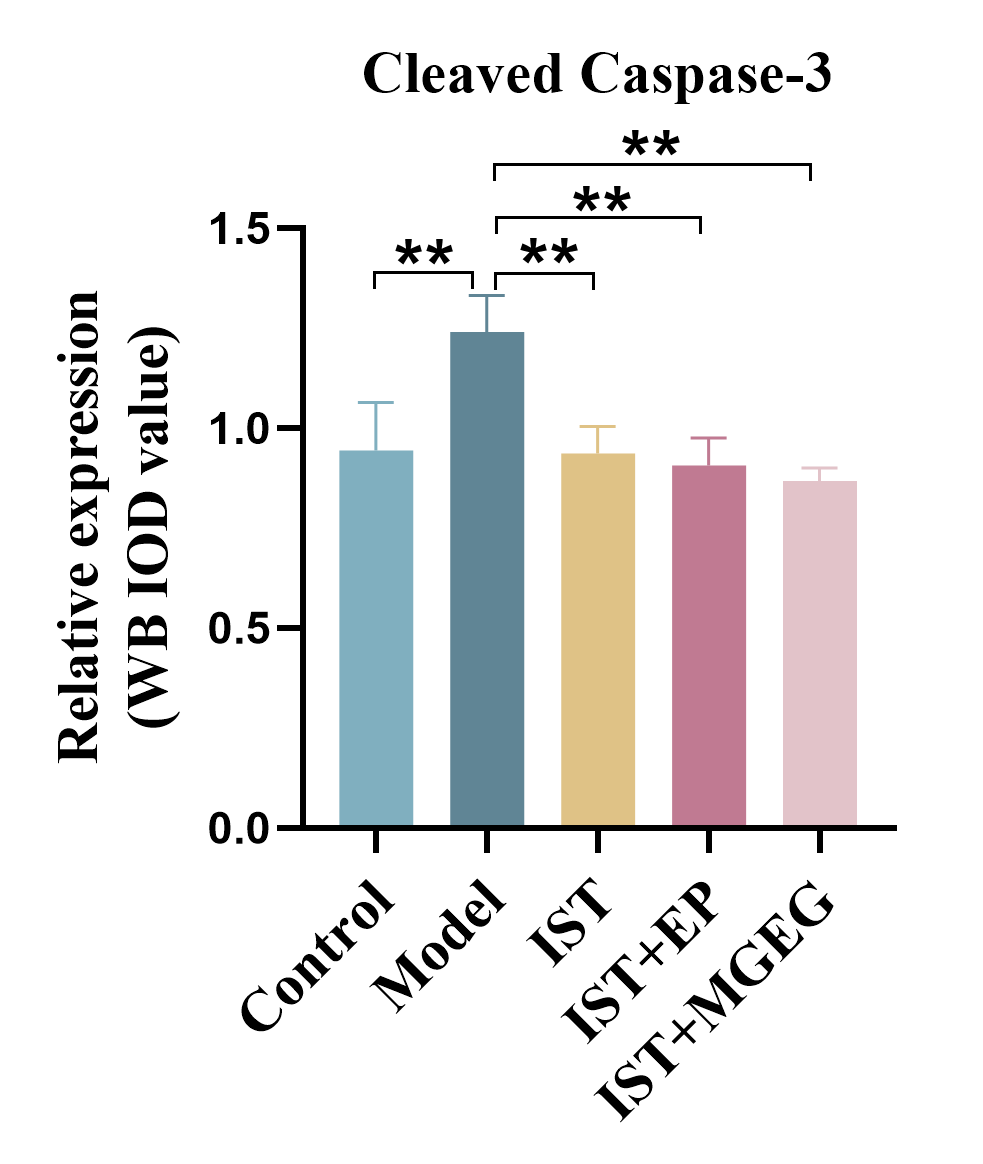

Supplement: Supplementary file 1 — Additional file 1. [file 13020_2025_1266_MOESM1_ESM.zip › Figure 7/Cle-cas3.tif]

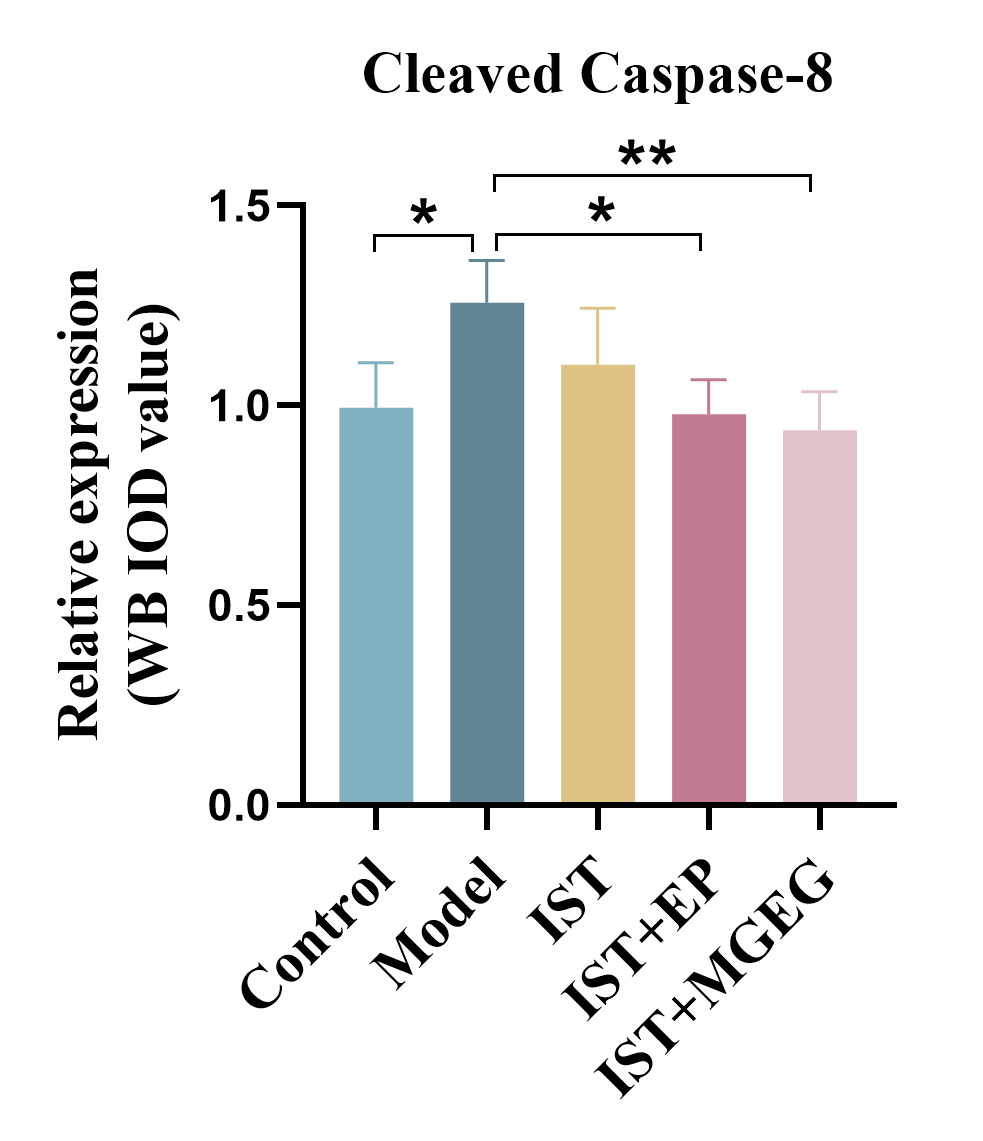

Supplement: Supplementary file 1 — Additional file 1. [file 13020_2025_1266_MOESM1_ESM.zip › Figure 7/Cle-cas8.tif]

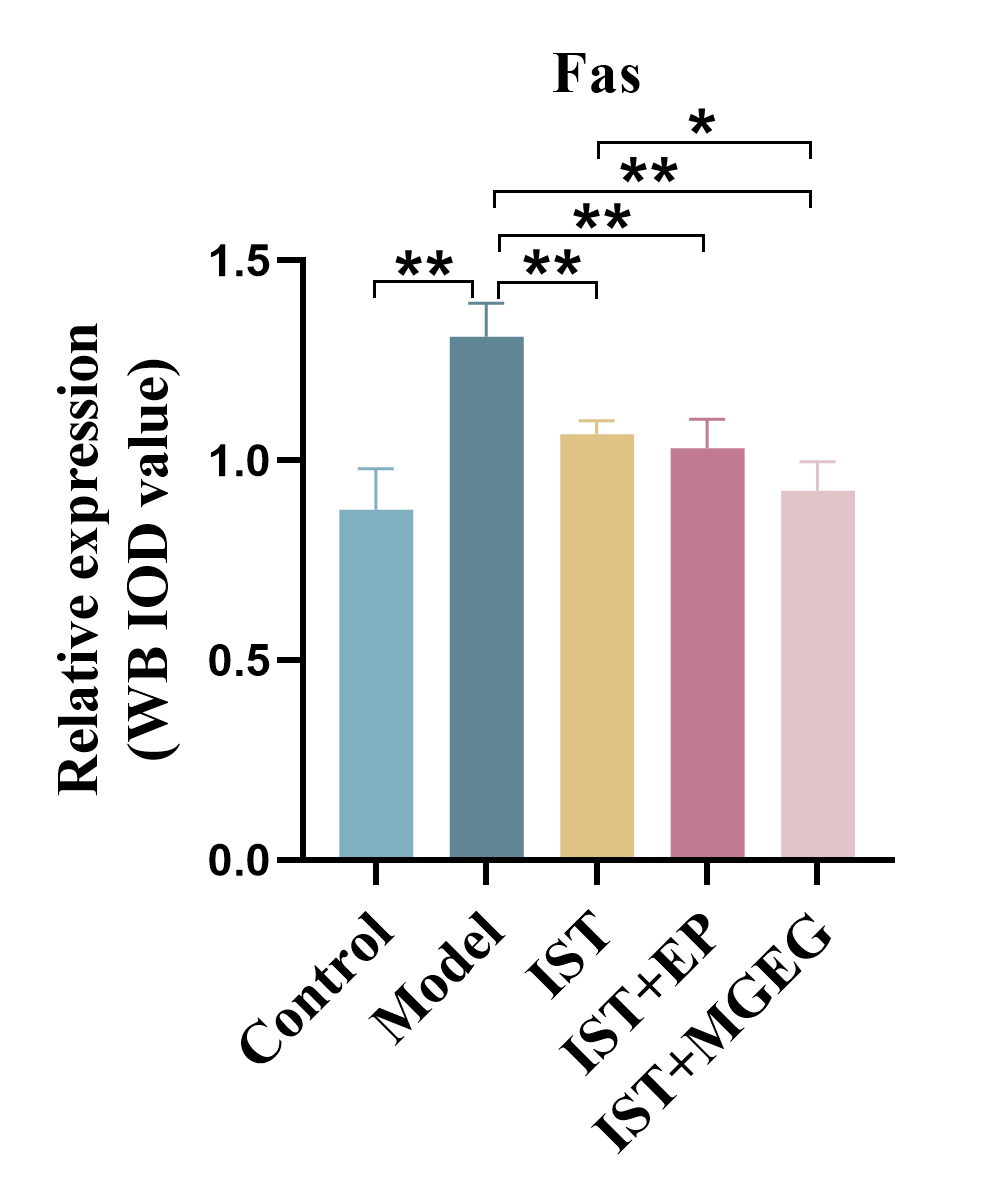

Supplement: Supplementary file 1 — Additional file 1. [file 13020_2025_1266_MOESM1_ESM.zip › Figure 7/Fas.tif]

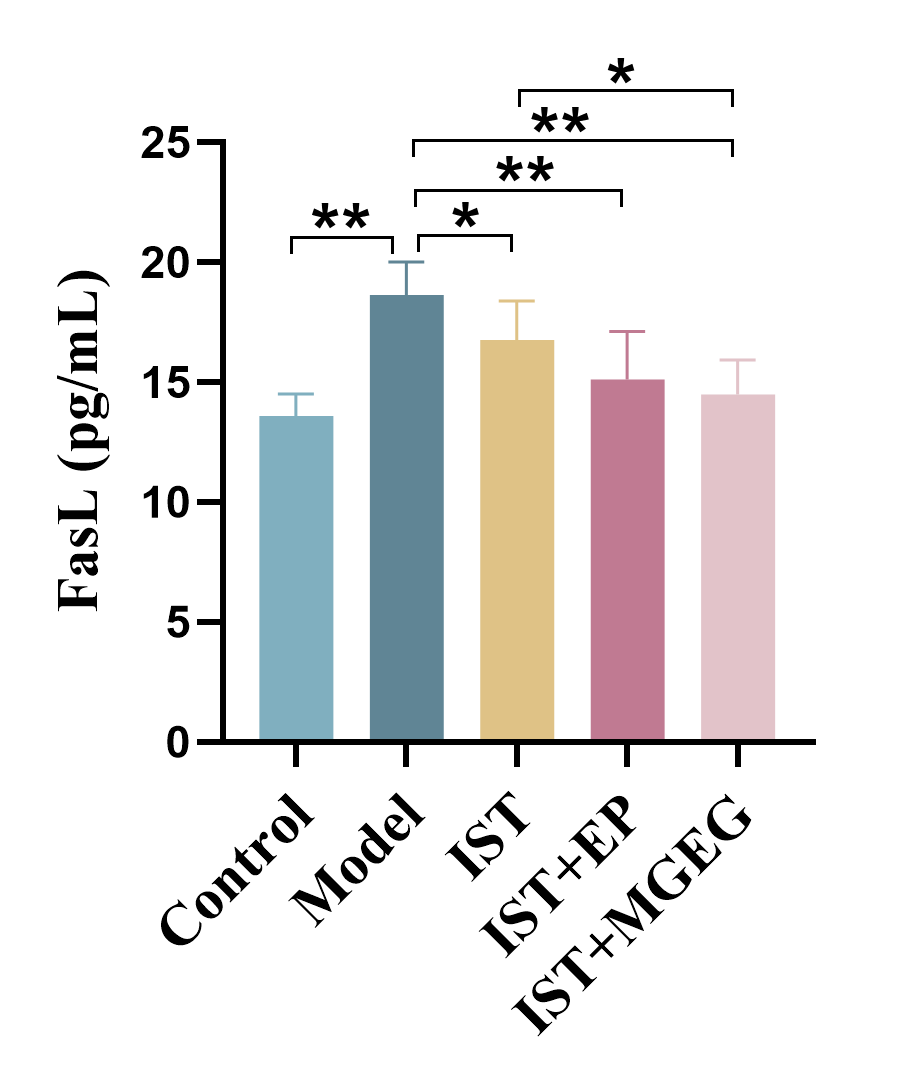

Supplement: Supplementary file 1 — Additional file 1. [file 13020_2025_1266_MOESM1_ESM.zip › Figure 7/FasL.tif]

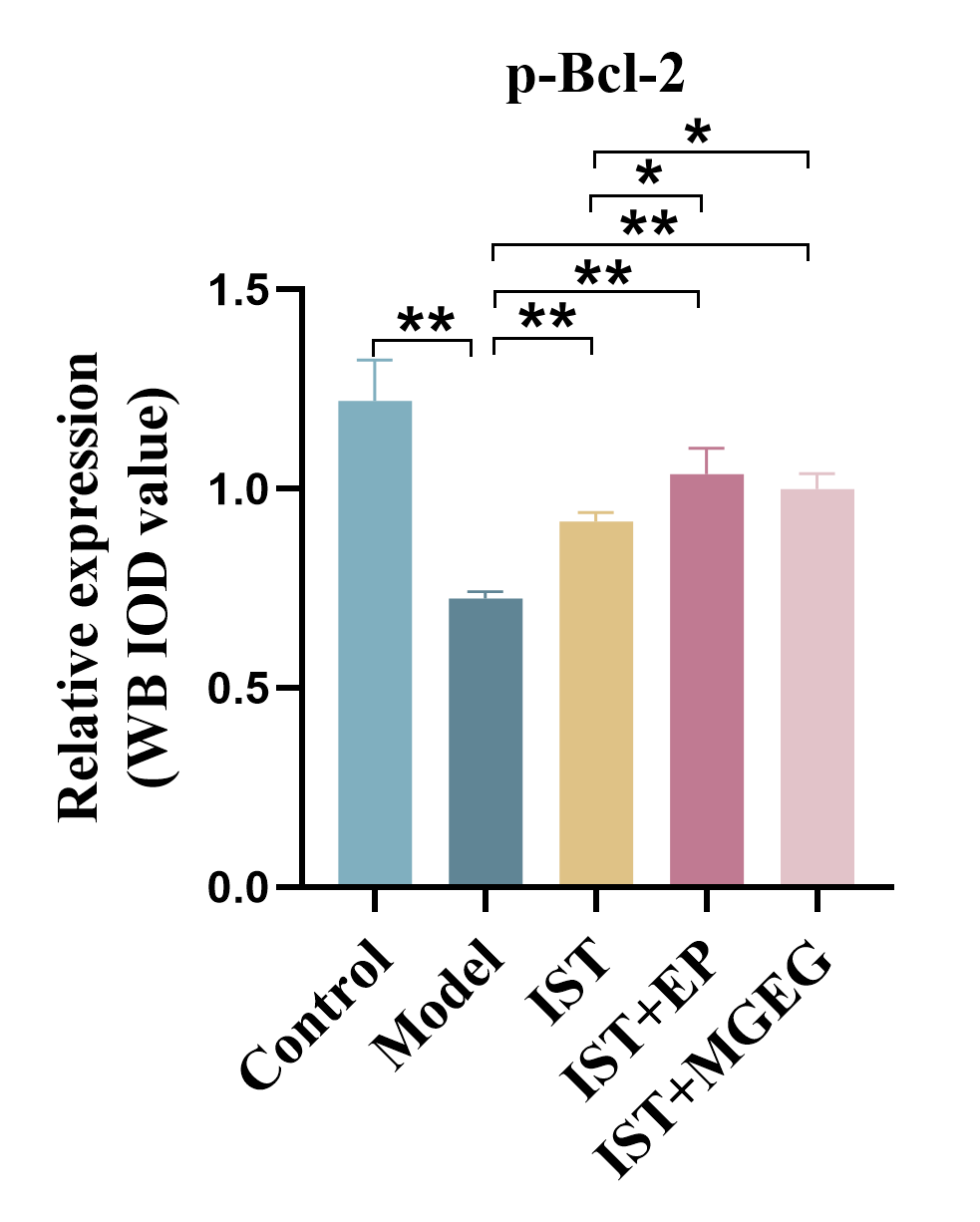

Supplement: Supplementary file 1 — Additional file 1. [file 13020_2025_1266_MOESM1_ESM.zip › Figure 7/p-Bcl2.tif]

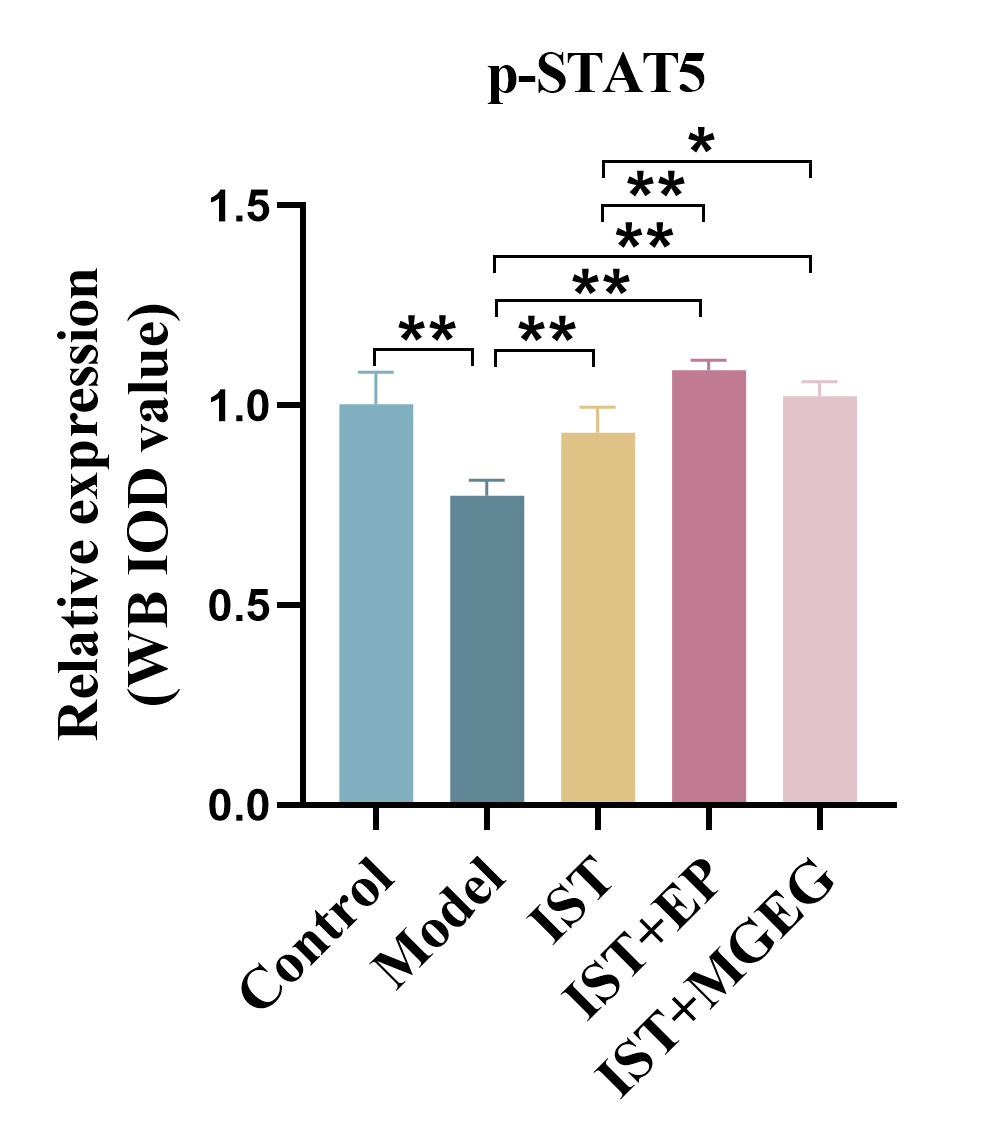

Supplement: Supplementary file 1 — Additional file 1. [file 13020_2025_1266_MOESM1_ESM.zip › Figure 7/p-STAT5.tif]

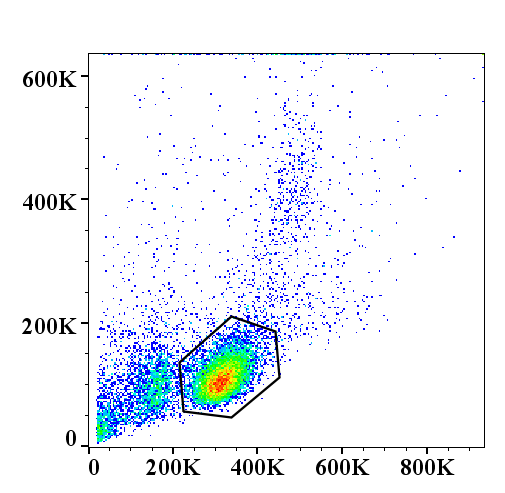

Supplement: Supplementary file 1 — Additional file 1. [file 13020_2025_1266_MOESM1_ESM.zip › Figure 8/1-FSC-SSC.tiff]

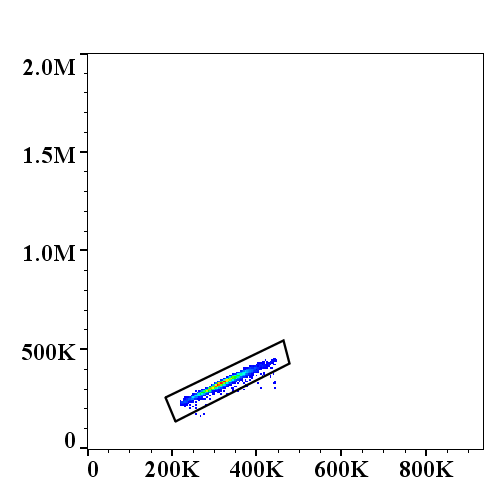

Supplement: Supplementary file 1 — Additional file 1. [file 13020_2025_1266_MOESM1_ESM.zip › Figure 8/2-FSC-FSC.tiff]

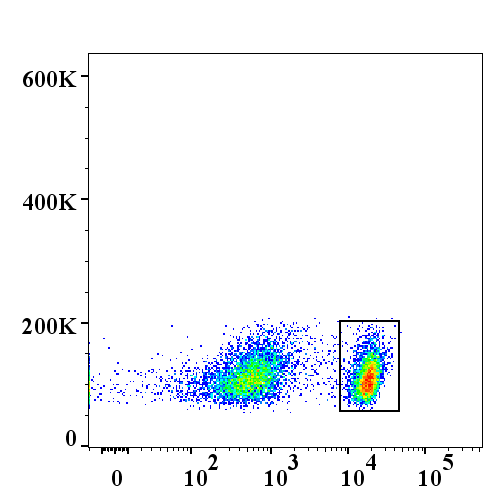

Supplement: Supplementary file 1 — Additional file 1. [file 13020_2025_1266_MOESM1_ESM.zip › Figure 8/3-CD4-FSC.tiff]

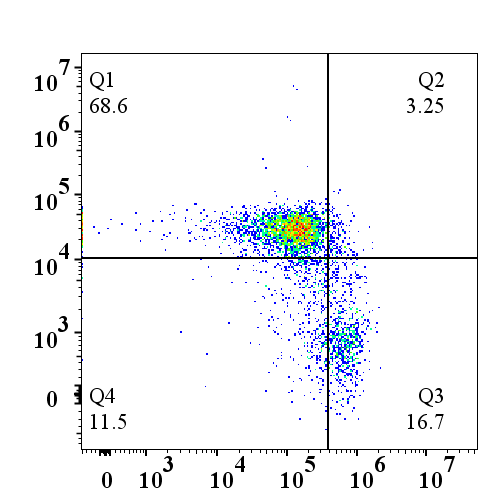

Supplement: Supplementary file 1 — Additional file 1. [file 13020_2025_1266_MOESM1_ESM.zip › Figure 8/Control.tiff]

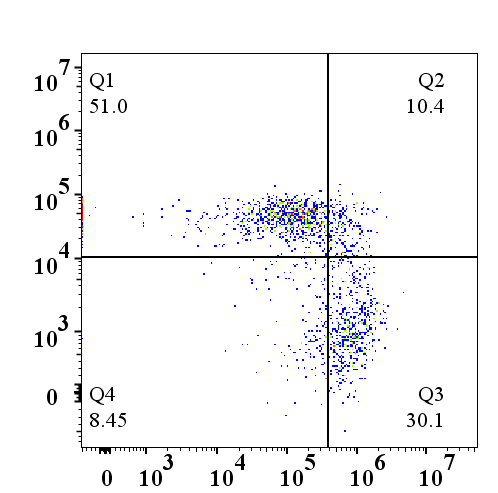

Supplement: Supplementary file 1 — Additional file 1. [file 13020_2025_1266_MOESM1_ESM.zip › Figure 8/IST+EP.tiff]

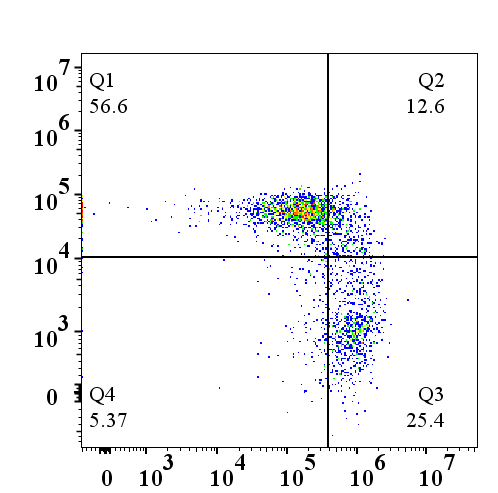

Supplement: Supplementary file 1 — Additional file 1. [file 13020_2025_1266_MOESM1_ESM.zip › Figure 8/IST+MGEG.tiff]

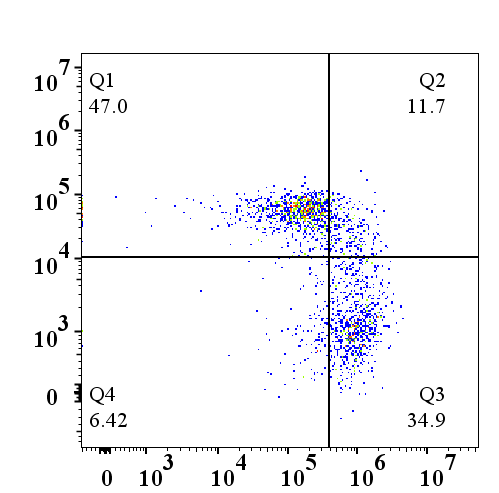

Supplement: Supplementary file 1 — Additional file 1. [file 13020_2025_1266_MOESM1_ESM.zip › Figure 8/IST.tiff]

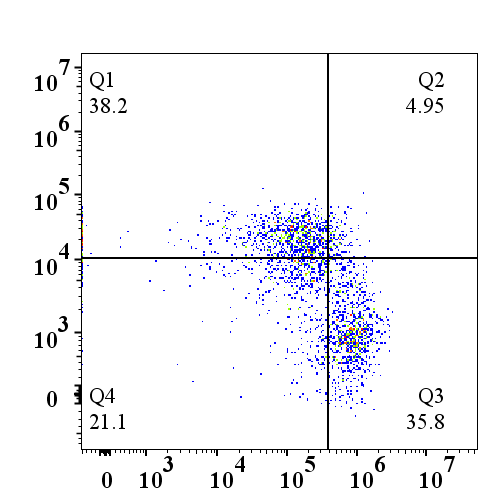

Supplement: Supplementary file 1 — Additional file 1. [file 13020_2025_1266_MOESM1_ESM.zip › Figure 8/Model.tiff]

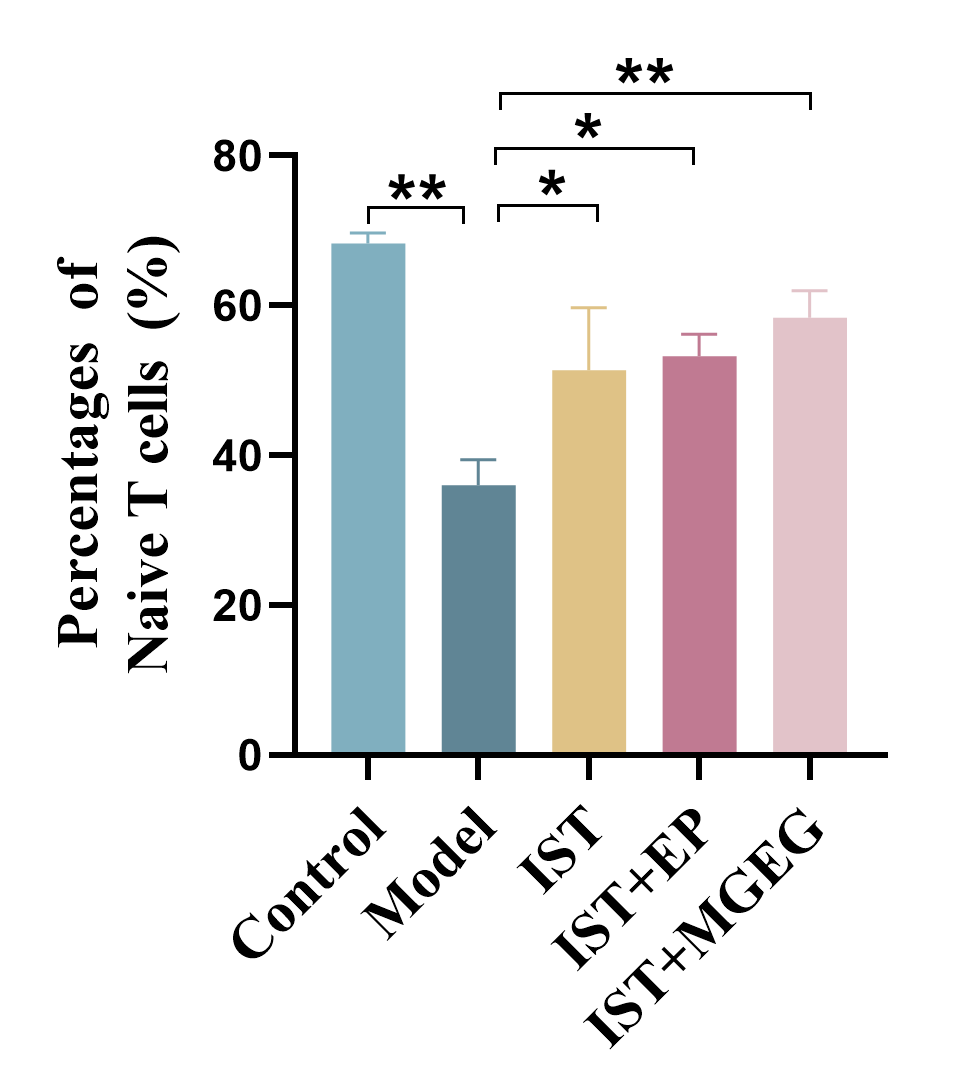

Supplement: Supplementary file 1 — Additional file 1. [file 13020_2025_1266_MOESM1_ESM.zip › Figure 8/naive T.tif]

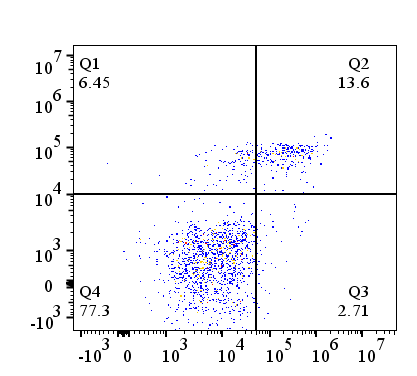

Supplement: Supplementary file 1 — Additional file 1. [file 13020_2025_1266_MOESM1_ESM.zip › Figure 8/Treg-Control.tiff]

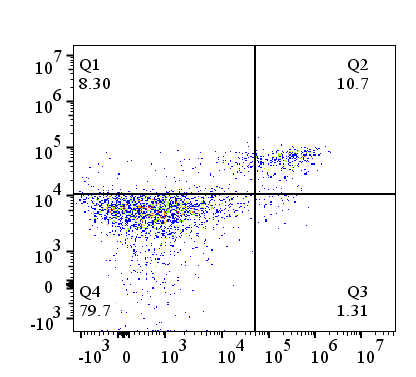

Supplement: Supplementary file 1 — Additional file 1. [file 13020_2025_1266_MOESM1_ESM.zip › Figure 8/Treg-IST+EP.tiff]

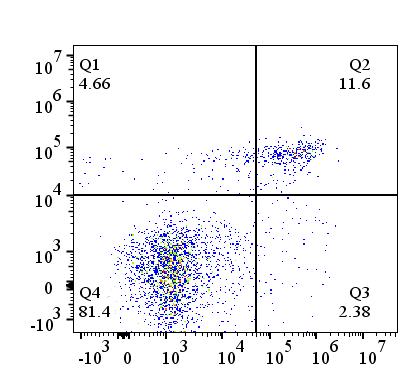

Supplement: Supplementary file 1 — Additional file 1. [file 13020_2025_1266_MOESM1_ESM.zip › Figure 8/Treg-IST+MGEG.tiff]

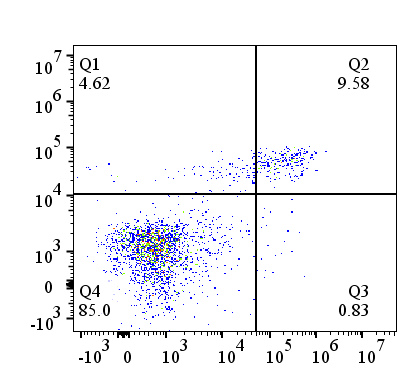

Supplement: Supplementary file 1 — Additional file 1. [file 13020_2025_1266_MOESM1_ESM.zip › Figure 8/Treg-IST.tiff]

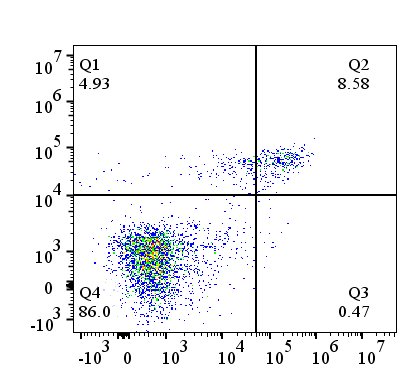

Supplement: Supplementary file 1 — Additional file 1. [file 13020_2025_1266_MOESM1_ESM.zip › Figure 8/Treg-Model.tiff]

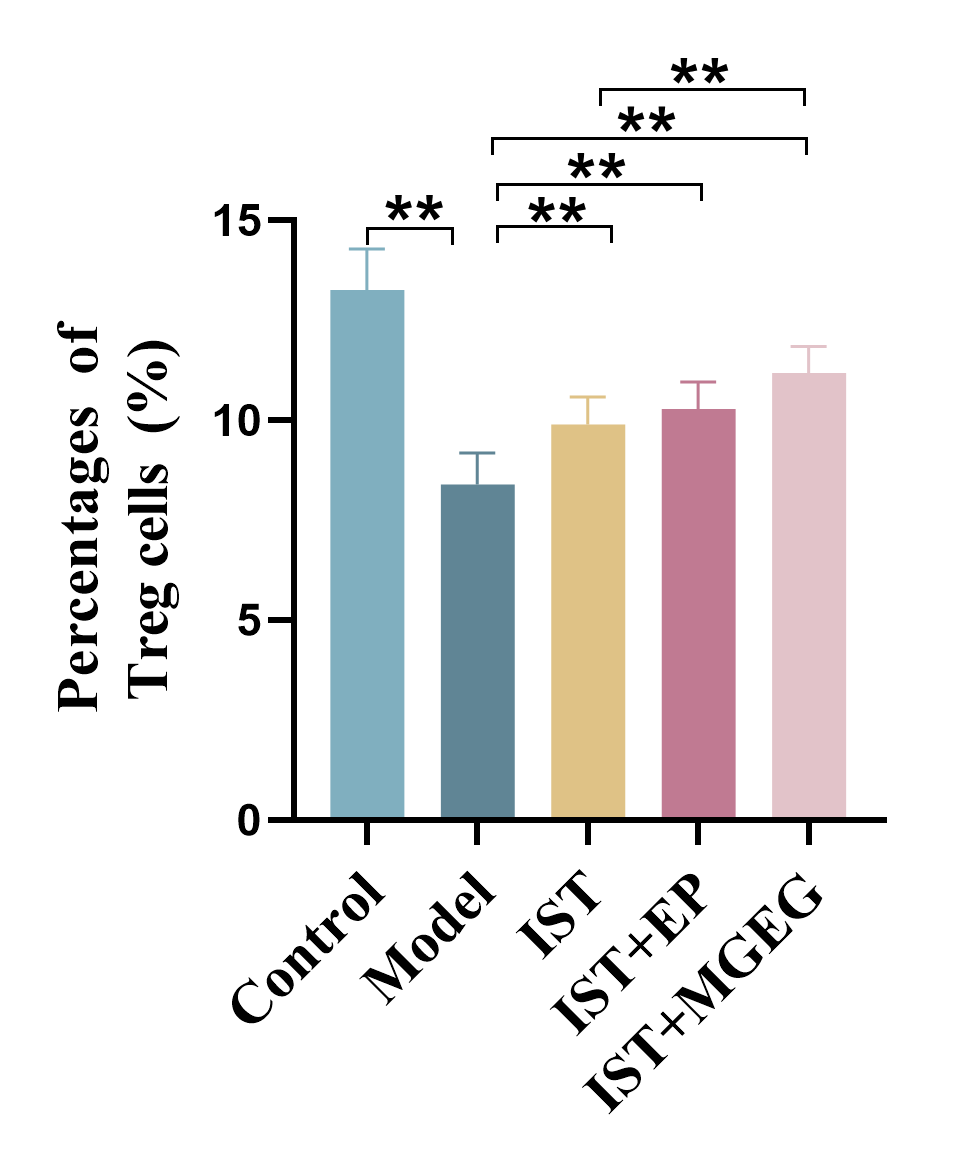

Supplement: Supplementary file 1 — Additional file 1. [file 13020_2025_1266_MOESM1_ESM.zip › Figure 8/Treg.tif]

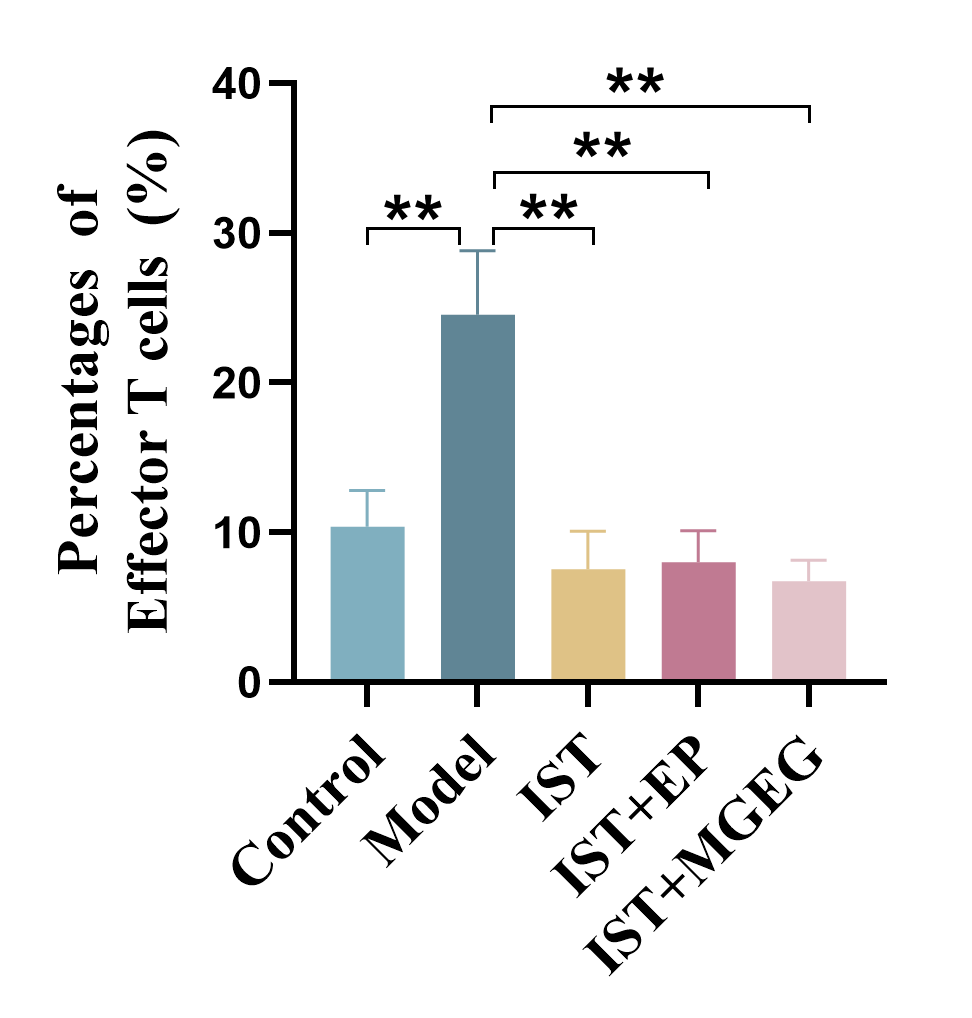

Supplement: Supplementary file 1 — Additional file 1. [file 13020_2025_1266_MOESM1_ESM.zip › Figure 8/效应T.tif]

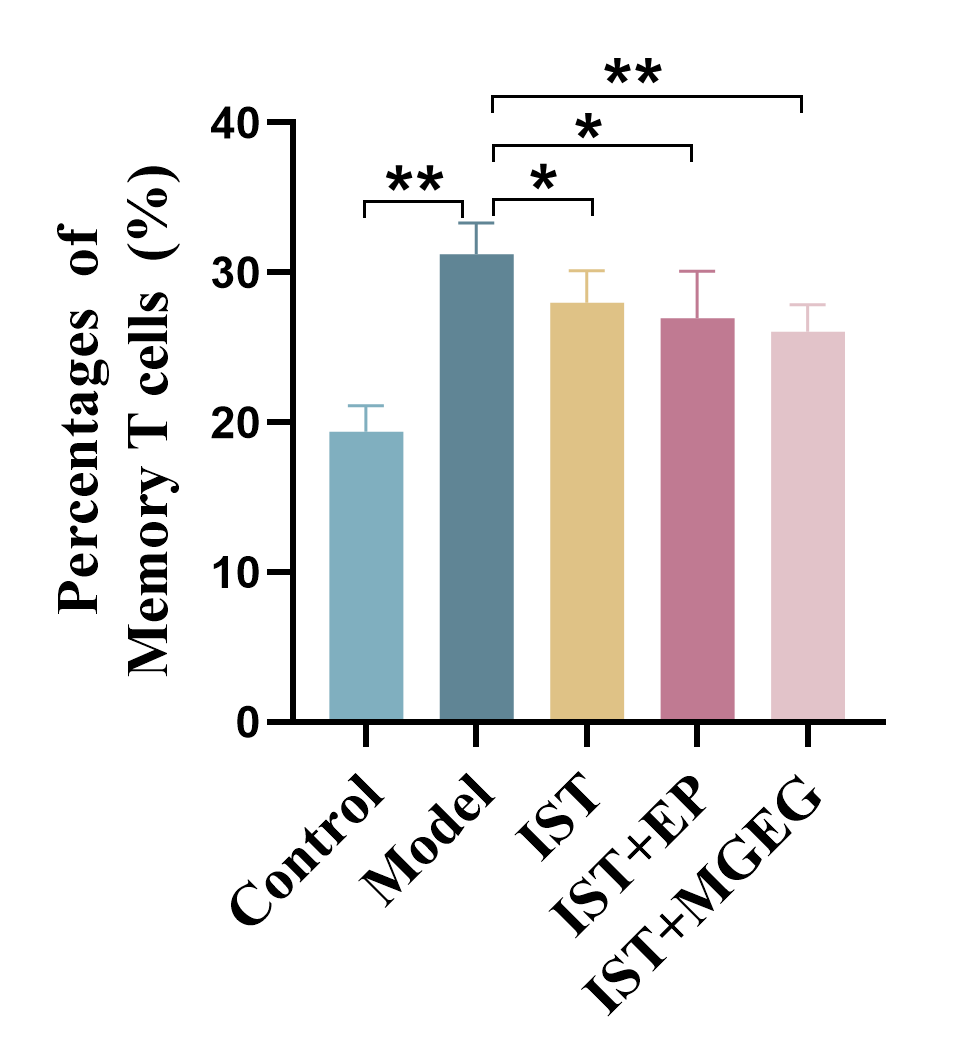

Supplement: Supplementary file 1 — Additional file 1. [file 13020_2025_1266_MOESM1_ESM.zip › Figure 8/记忆T.tif]

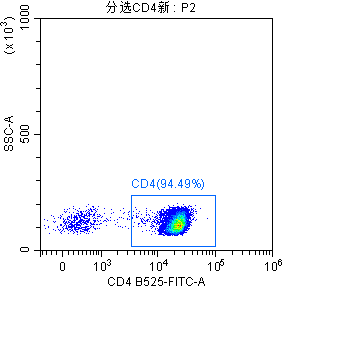

Supplement: Supplementary file 1 — Additional file 1. [file 13020_2025_1266_MOESM1_ESM.zip › Figure 9/After sorting CD4.tif]

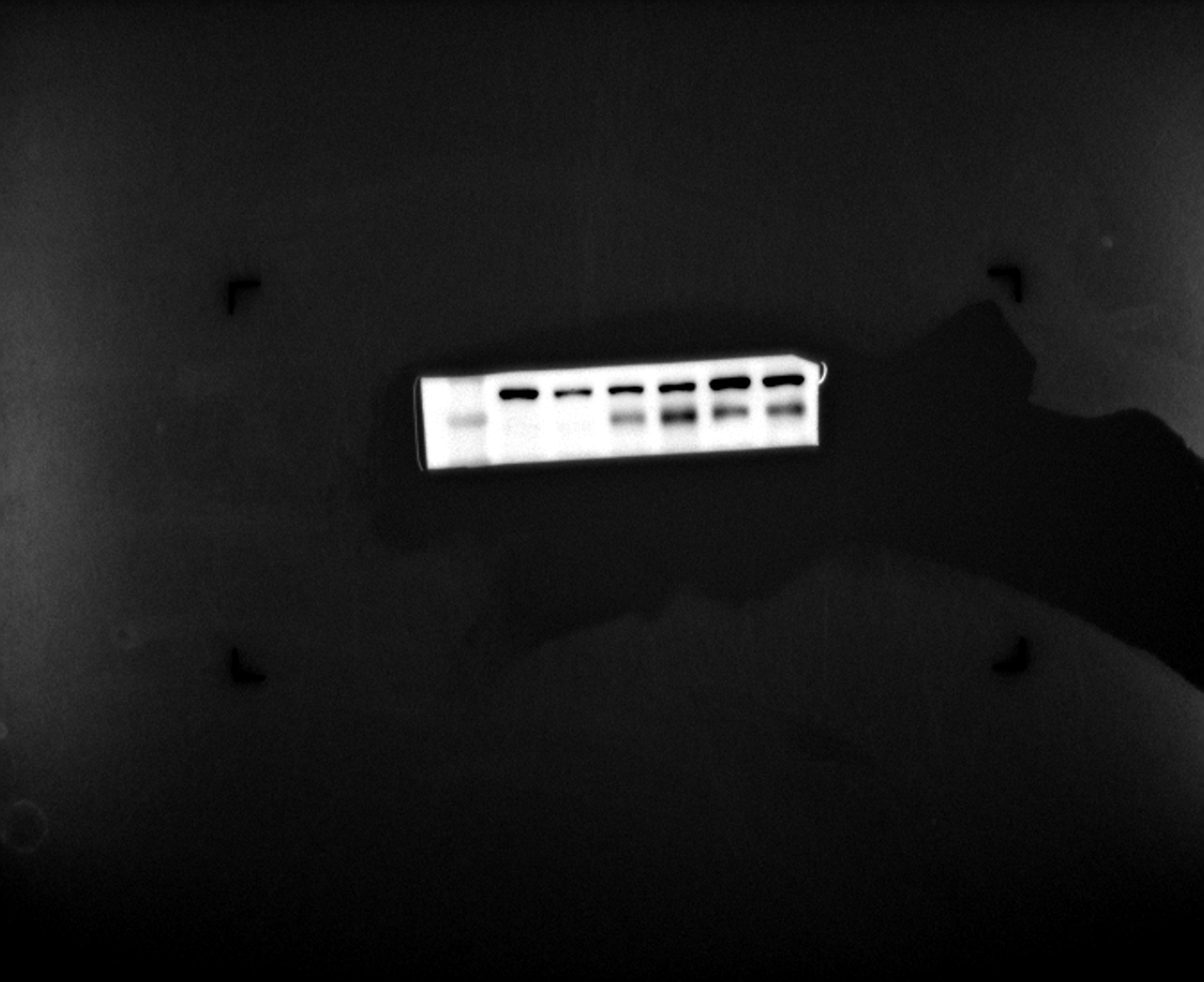

Supplement: Supplementary file 1 — Additional file 1. [file 13020_2025_1266_MOESM1_ESM.zip › Figure 9/bands/EOS-1(Figure) HC.Tif]

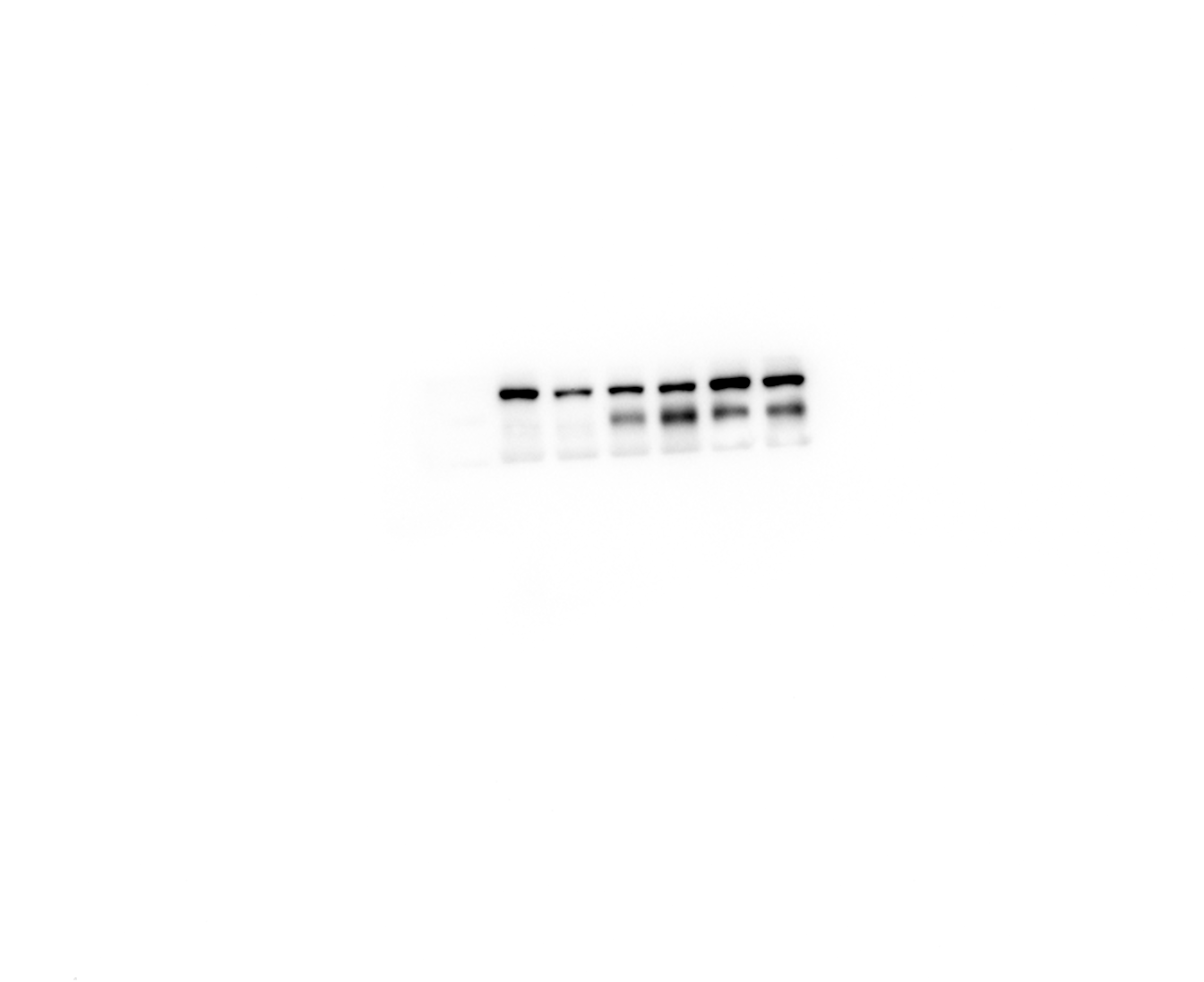

Supplement: Supplementary file 1 — Additional file 1. [file 13020_2025_1266_MOESM1_ESM.zip › Figure 9/bands/EOS-1(Figure).Tif]

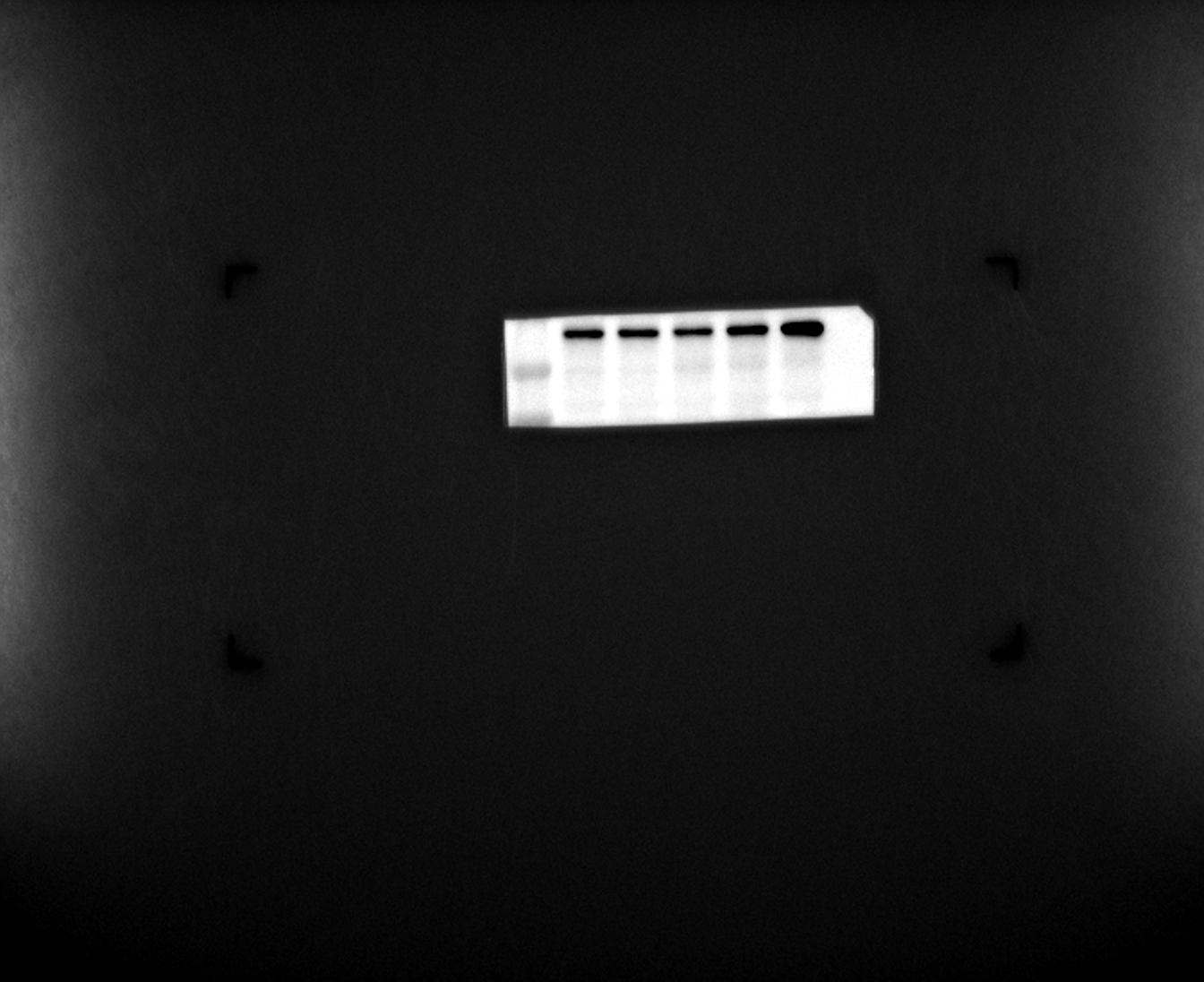

Supplement: Supplementary file 1 — Additional file 1. [file 13020_2025_1266_MOESM1_ESM.zip › Figure 9/bands/EOS-2 HC.Tif]

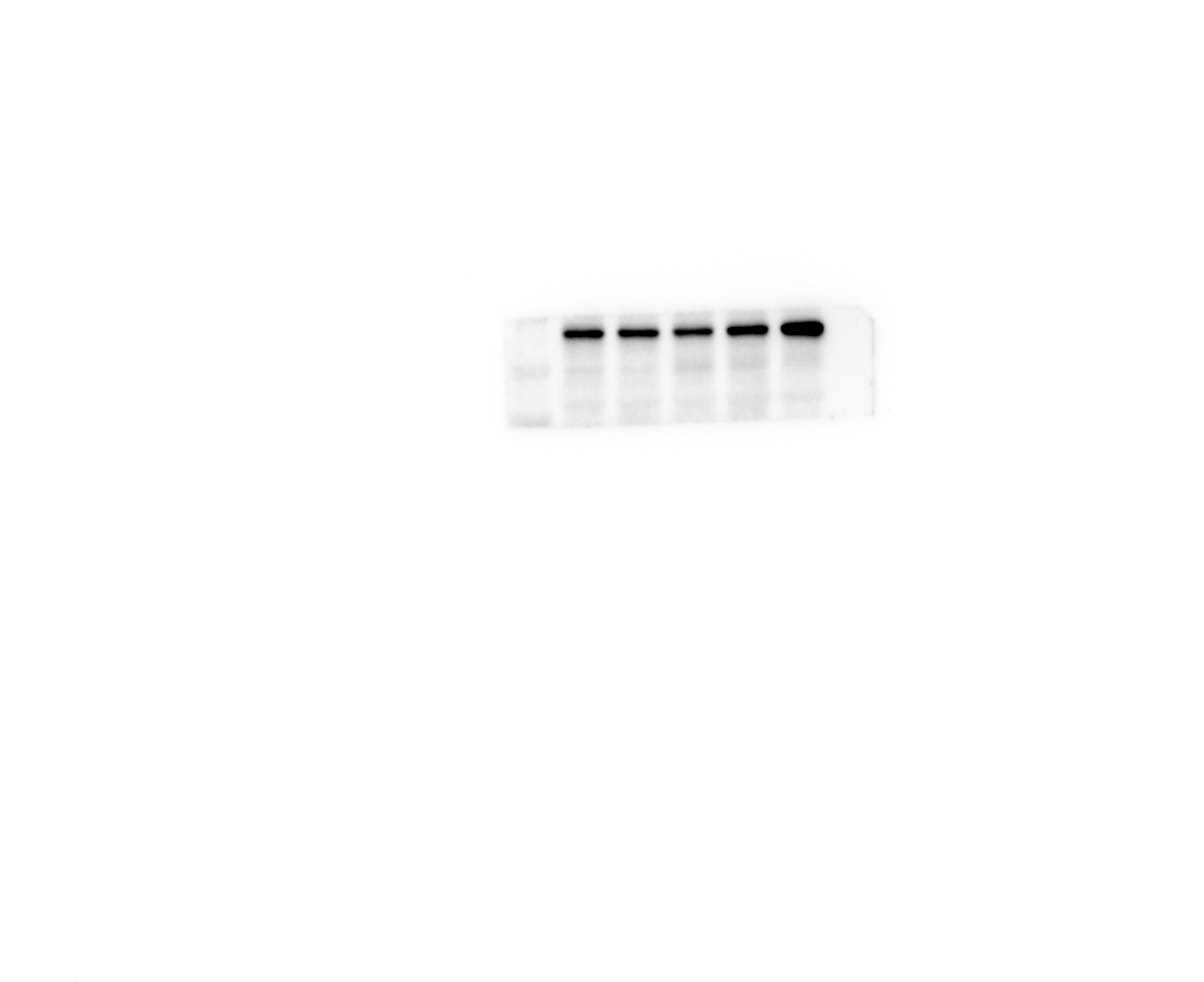

Supplement: Supplementary file 1 — Additional file 1. [file 13020_2025_1266_MOESM1_ESM.zip › Figure 9/bands/EOS-2.Tif]

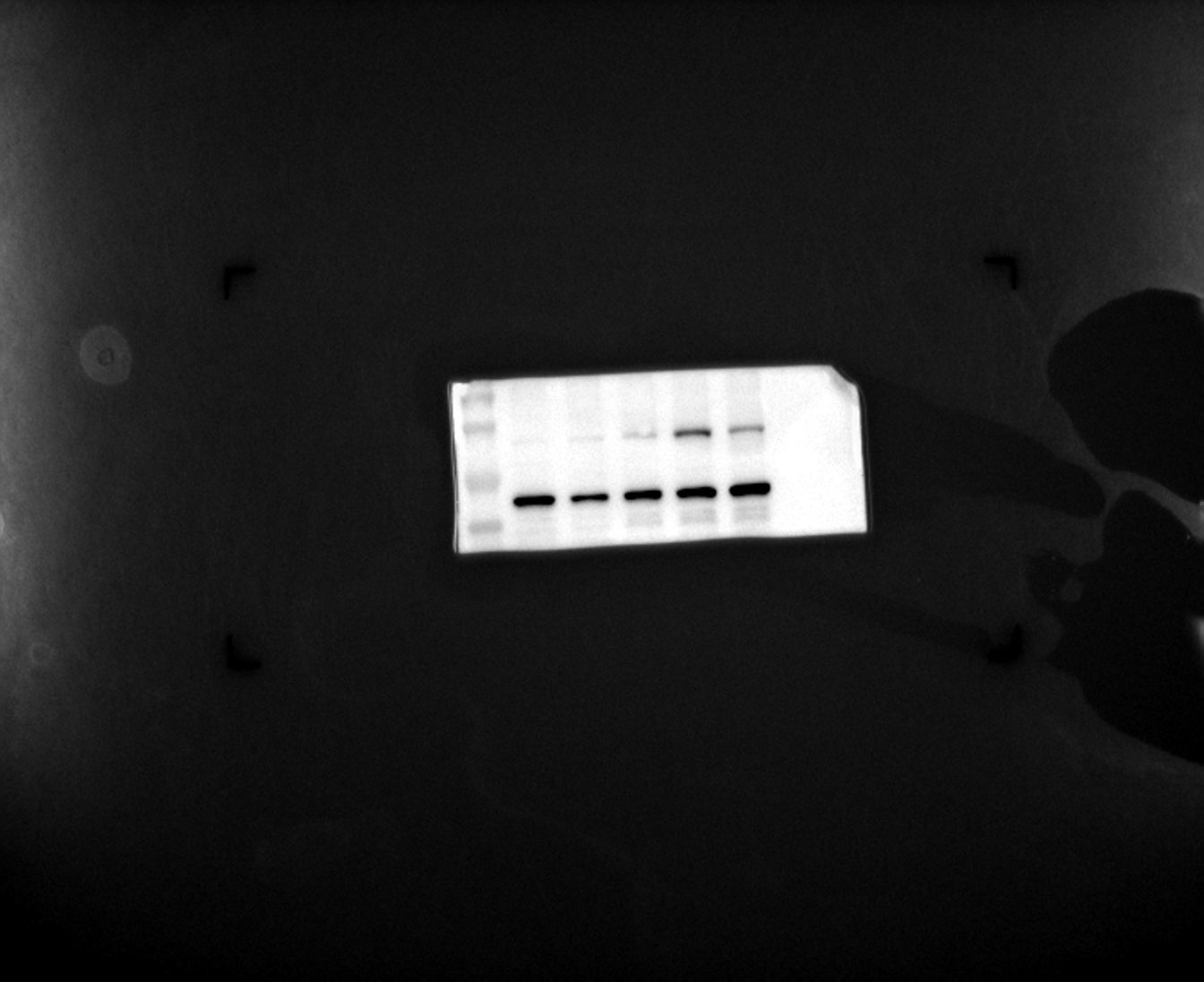

Supplement: Supplementary file 1 — Additional file 1. [file 13020_2025_1266_MOESM1_ESM.zip › Figure 9/bands/EOS-3 HC.Tif]

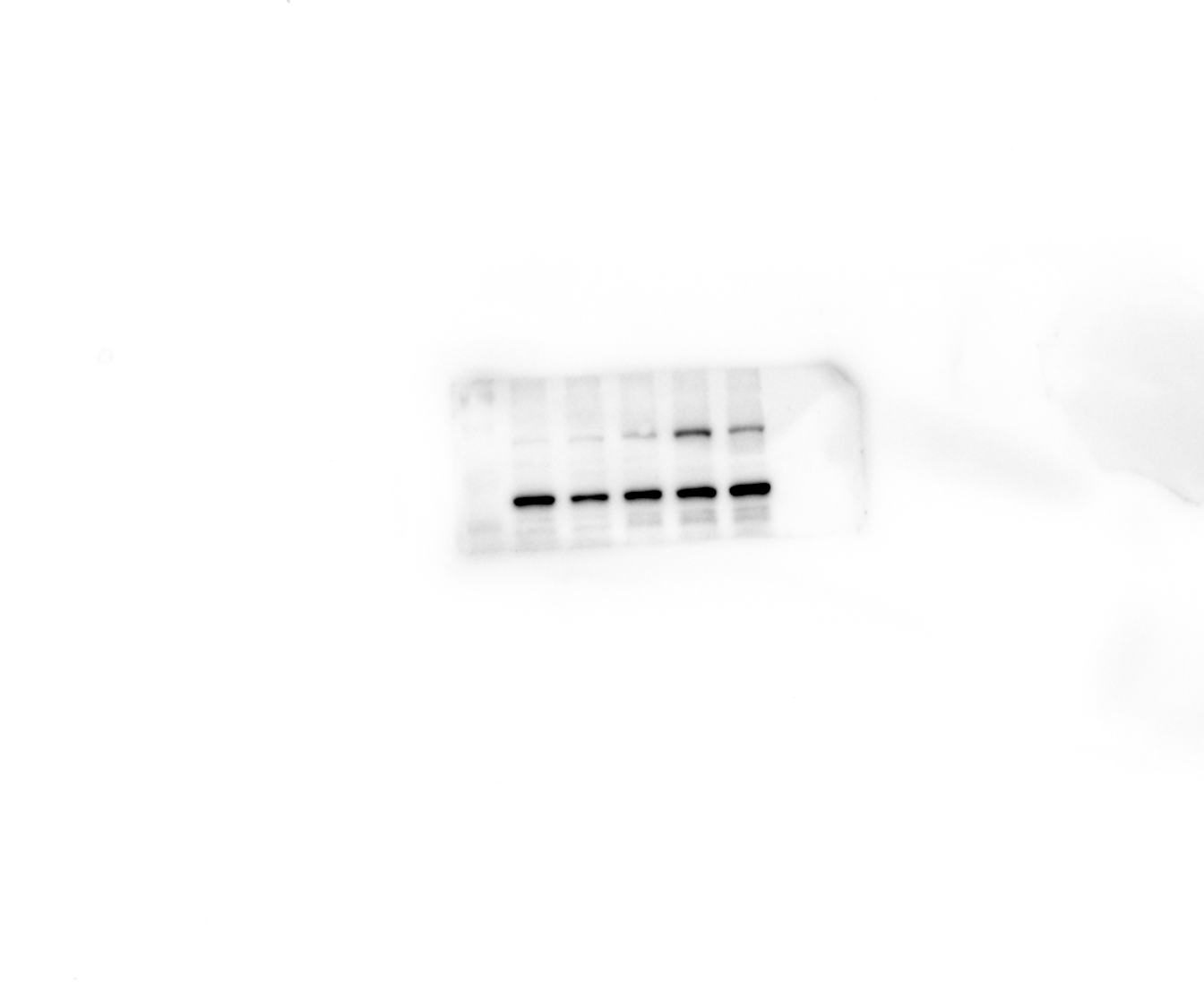

Supplement: Supplementary file 1 — Additional file 1. [file 13020_2025_1266_MOESM1_ESM.zip › Figure 9/bands/EOS-3.Tif]

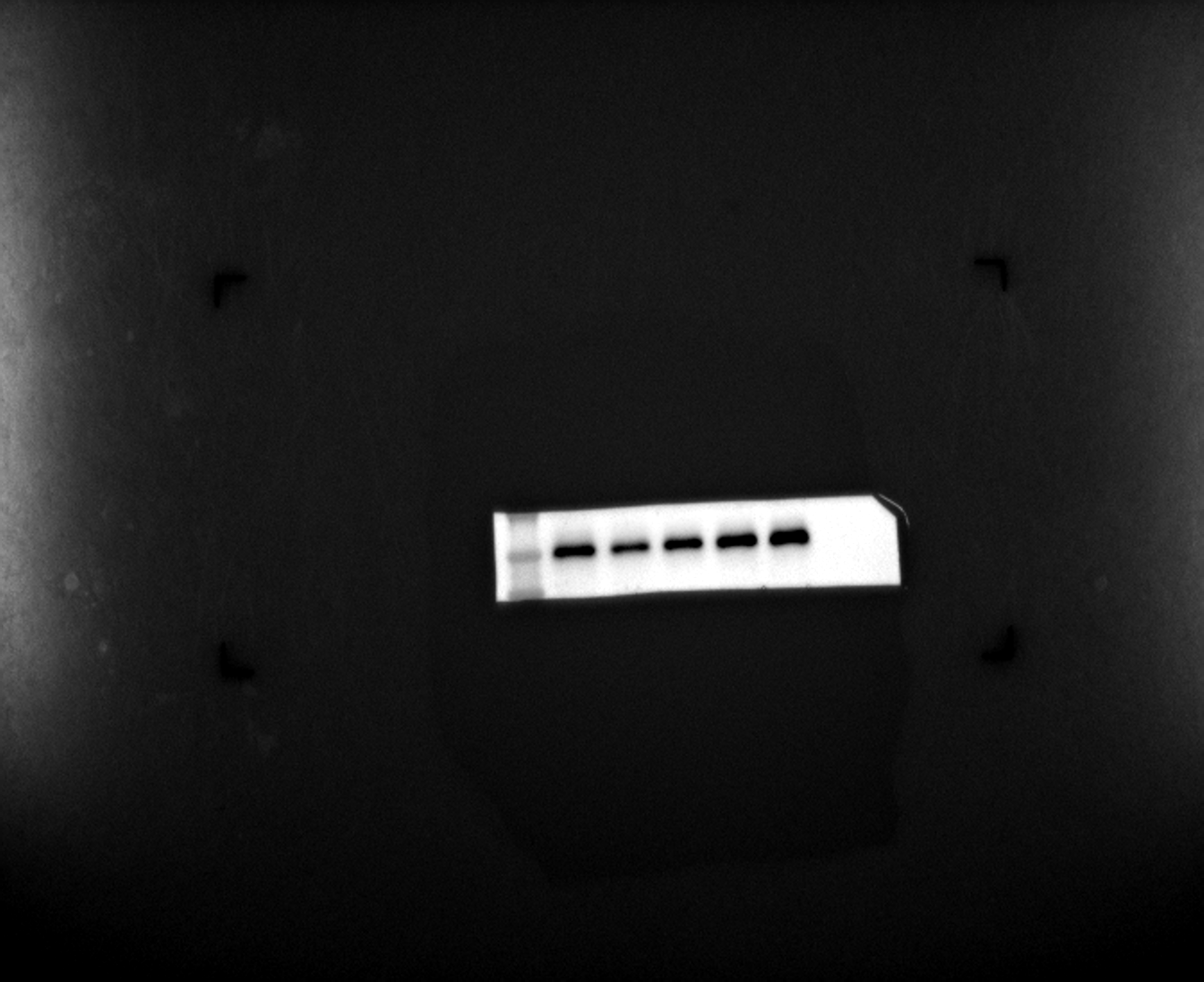

Supplement: Supplementary file 1 — Additional file 1. [file 13020_2025_1266_MOESM1_ESM.zip › Figure 9/bands/FOXP3-1(Figure) HC.Tif]

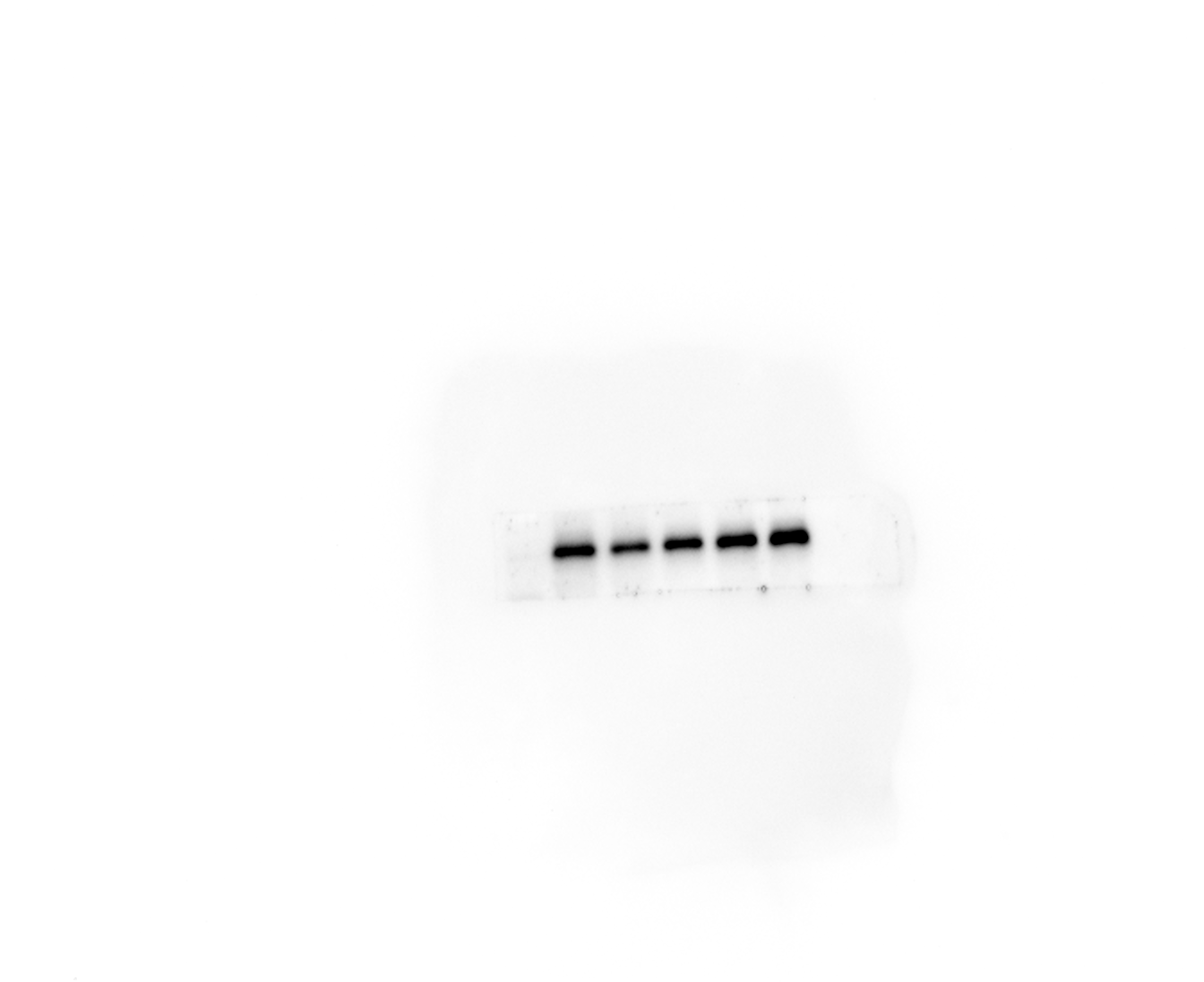

Supplement: Supplementary file 1 — Additional file 1. [file 13020_2025_1266_MOESM1_ESM.zip › Figure 9/bands/FOXP3-1(Figure).Tif]

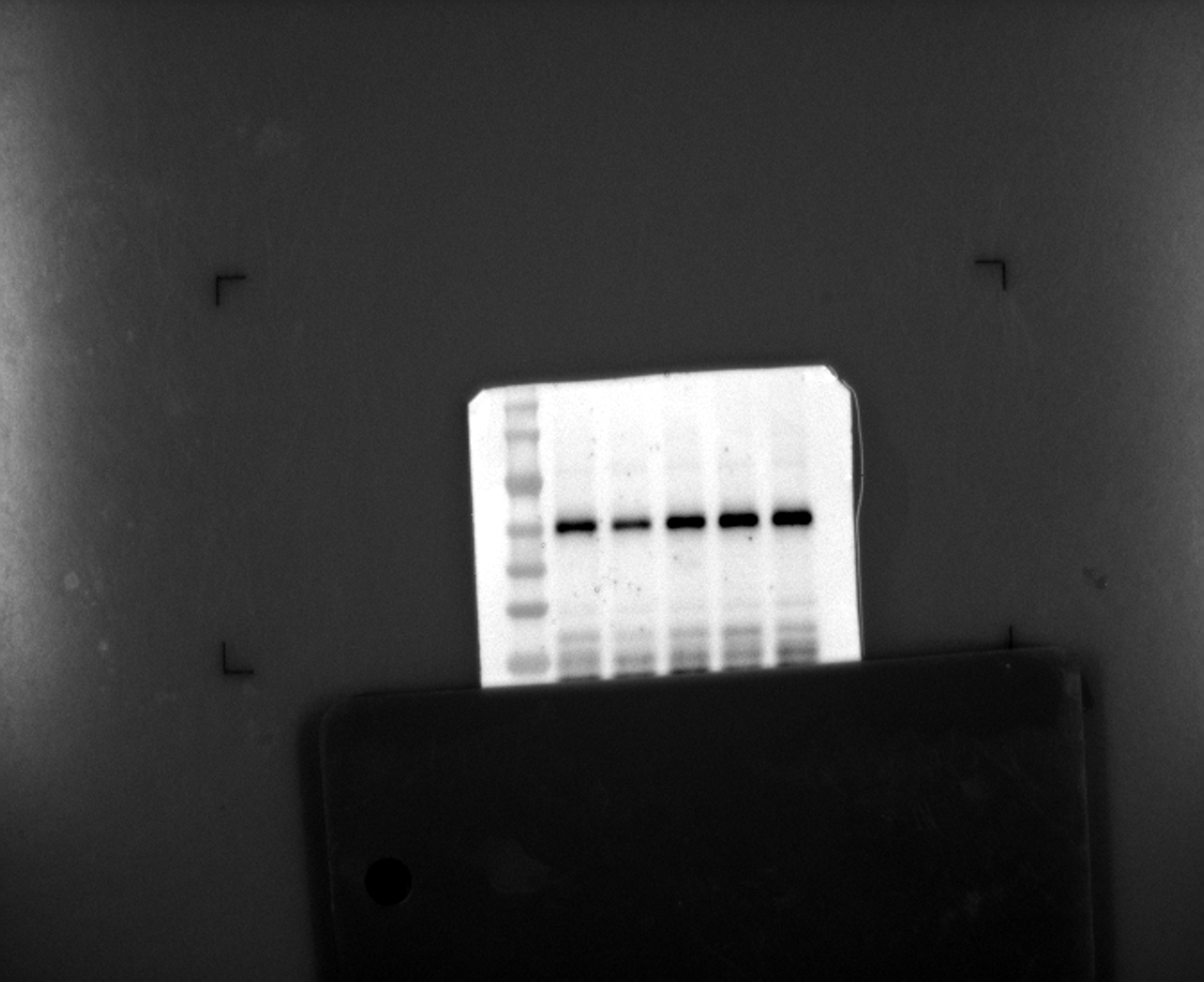

Supplement: Supplementary file 1 — Additional file 1. [file 13020_2025_1266_MOESM1_ESM.zip › Figure 9/bands/FOXP3-2 HC.Tif]

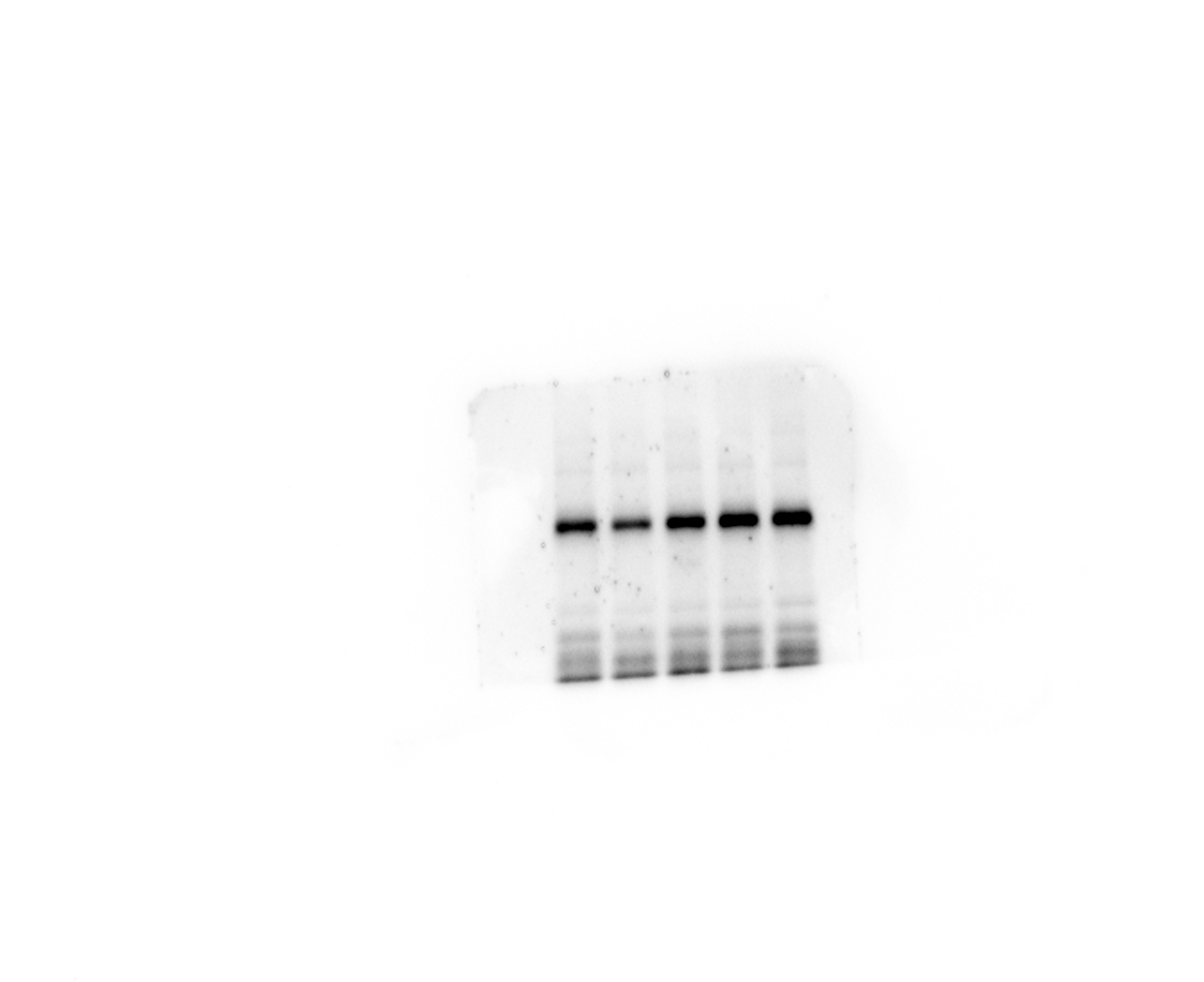

Supplement: Supplementary file 1 — Additional file 1. [file 13020_2025_1266_MOESM1_ESM.zip › Figure 9/bands/FOXP3-2.Tif]

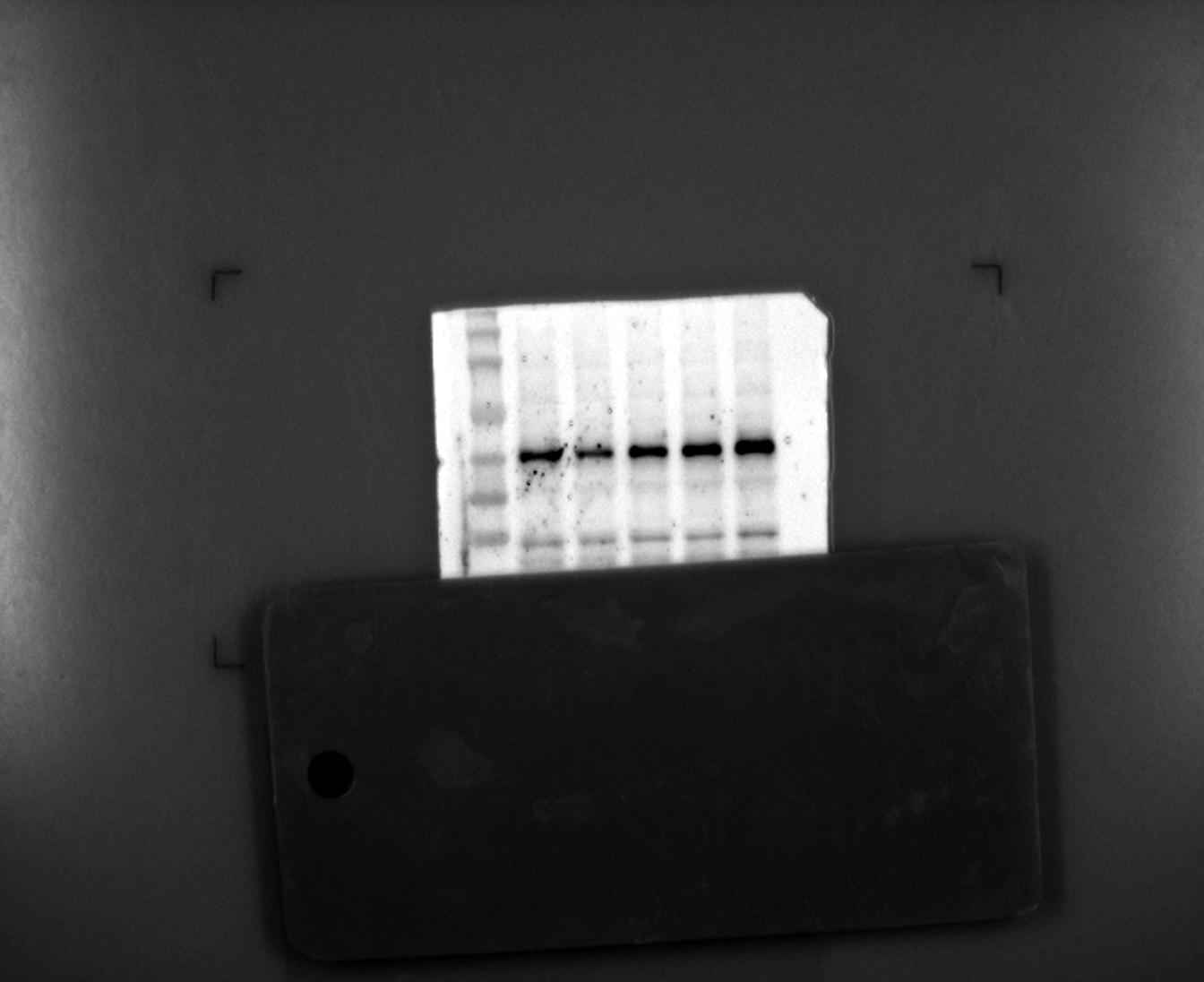

Supplement: Supplementary file 1 — Additional file 1. [file 13020_2025_1266_MOESM1_ESM.zip › Figure 9/bands/FOXP3-3 HC.Tif]

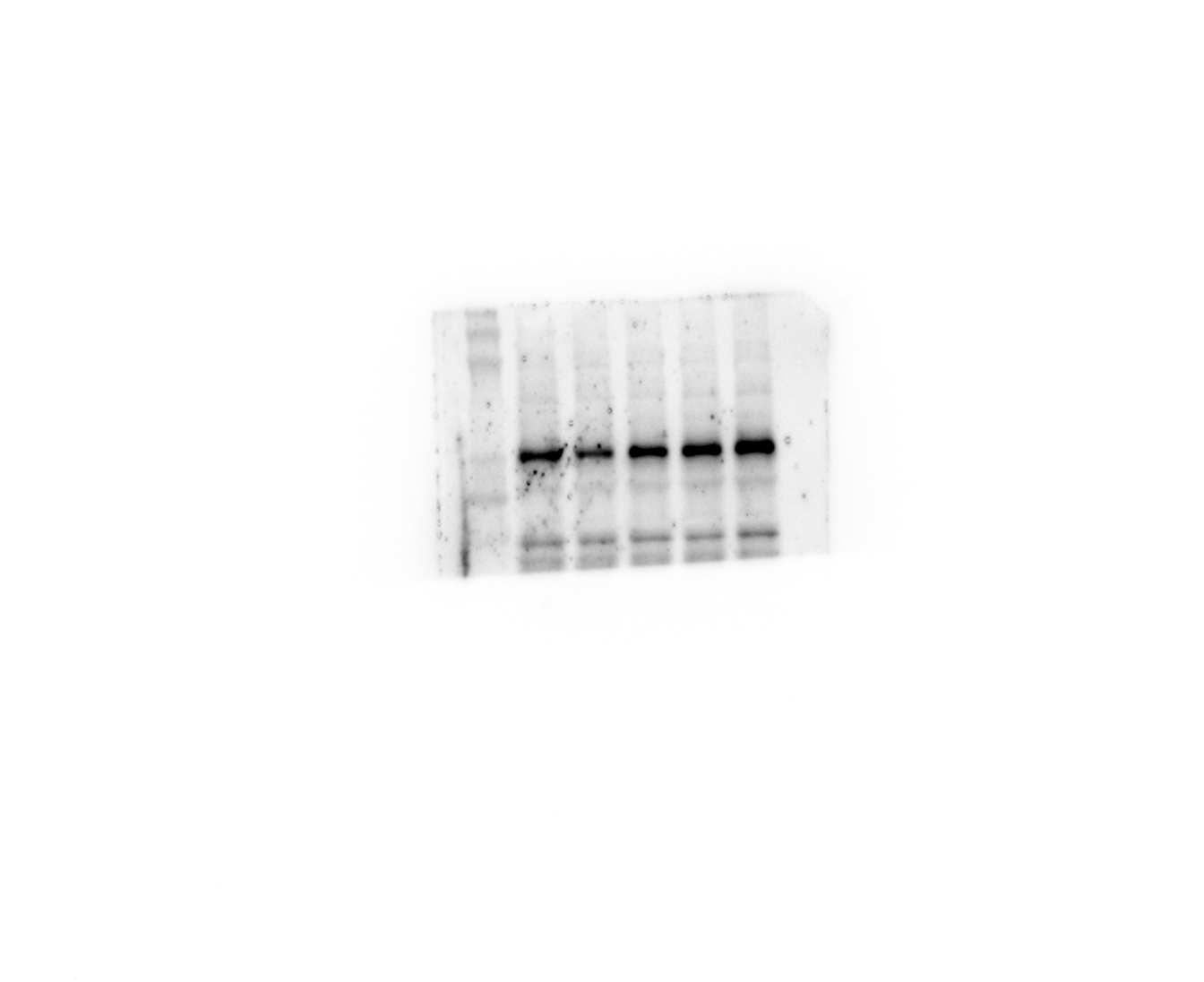

Supplement: Supplementary file 1 — Additional file 1. [file 13020_2025_1266_MOESM1_ESM.zip › Figure 9/bands/FOXP3-3.Tif]

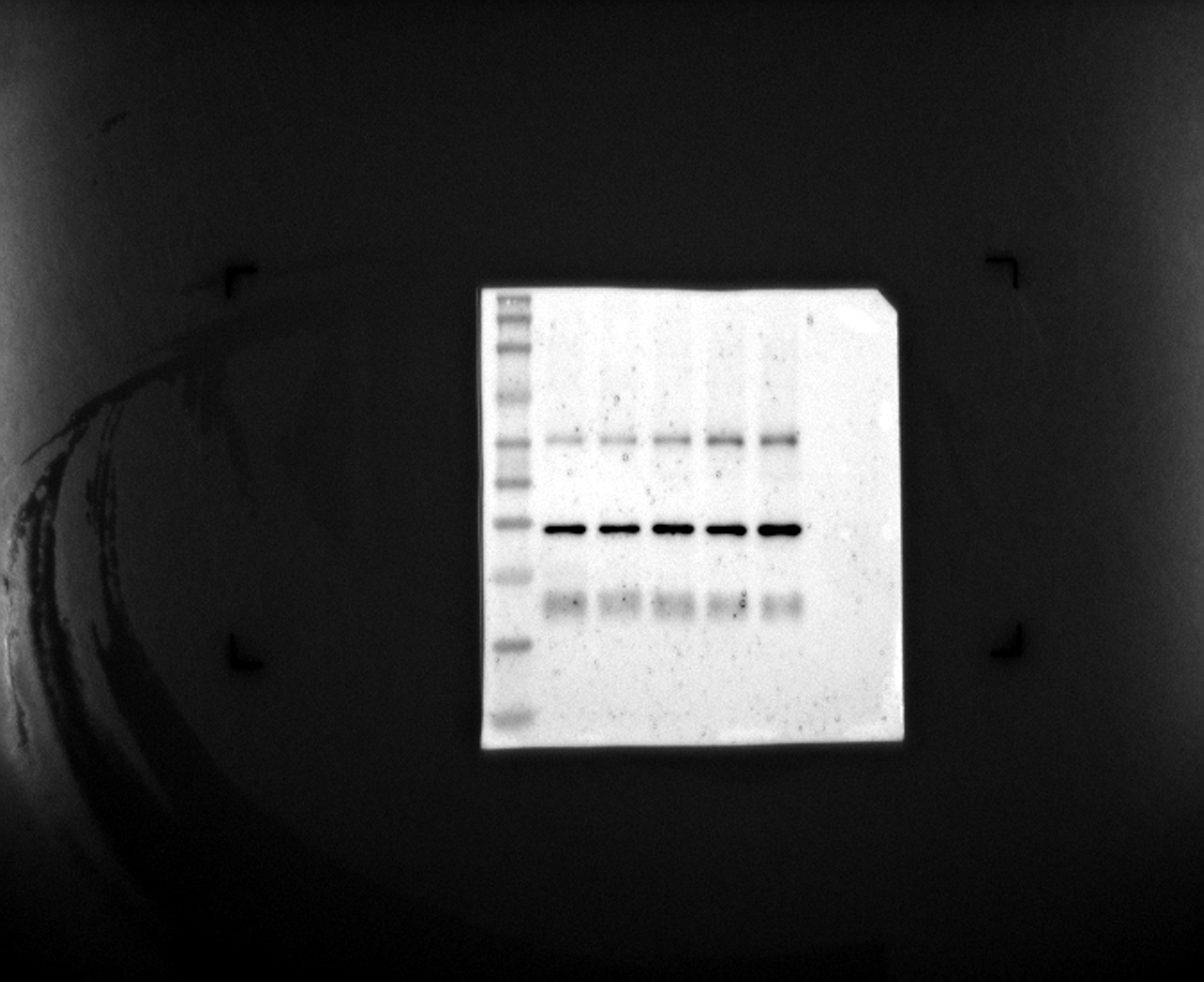

Supplement: Supplementary file 1 — Additional file 1. [file 13020_2025_1266_MOESM1_ESM.zip › Figure 9/bands/GAPDH-1 HC.Tif]

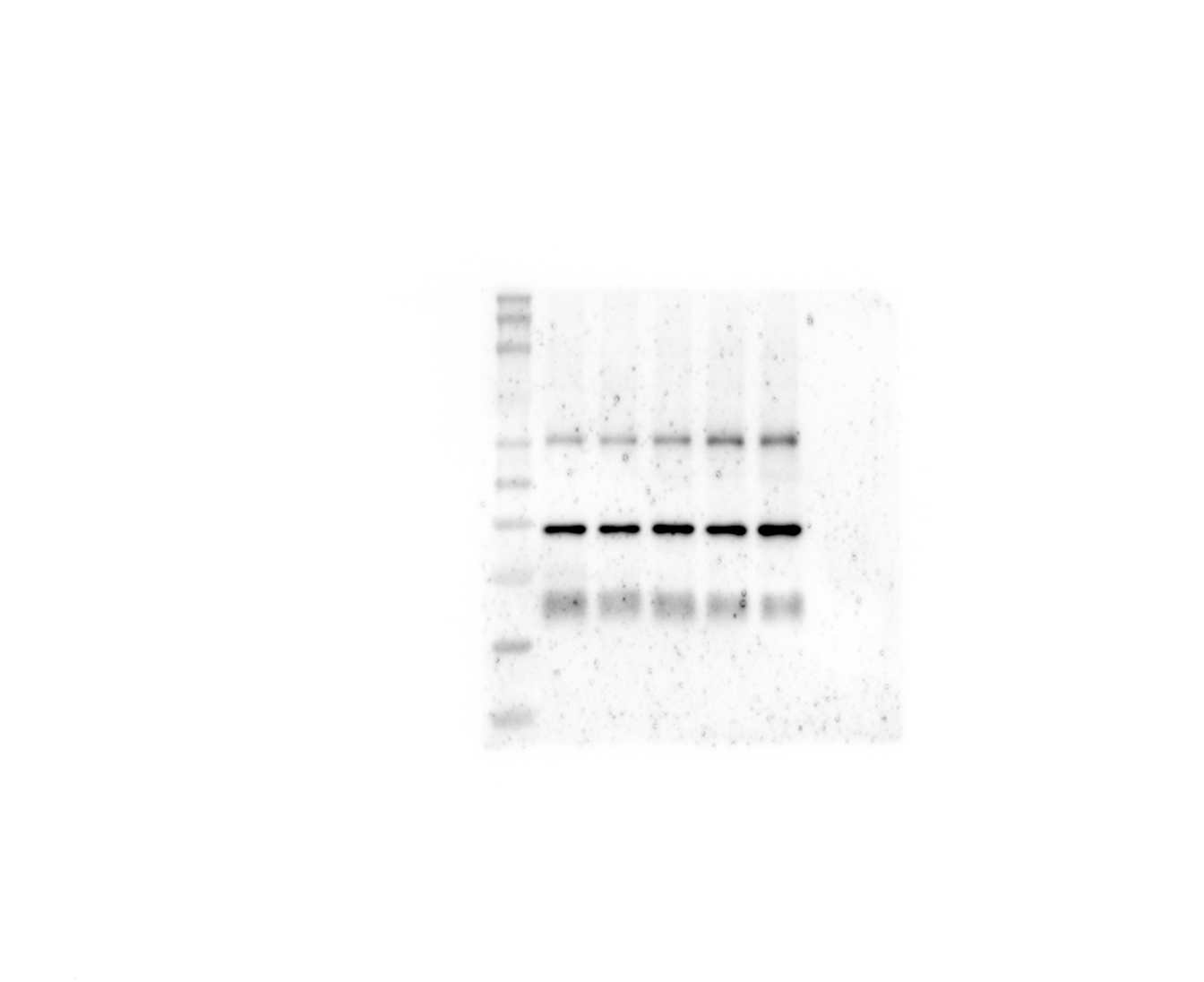

Supplement: Supplementary file 1 — Additional file 1. [file 13020_2025_1266_MOESM1_ESM.zip › Figure 9/bands/GAPDH-1.Tif]

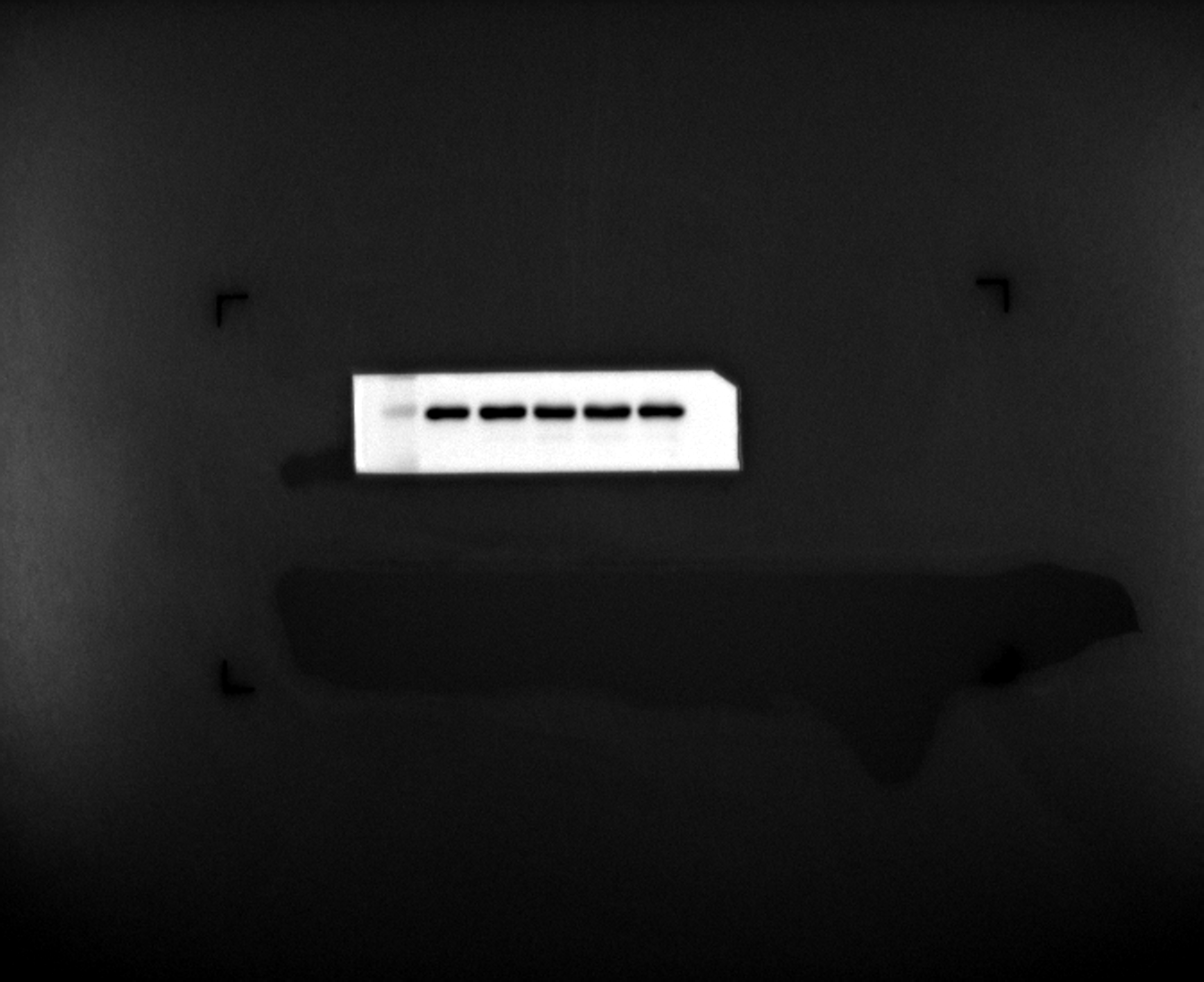

Supplement: Supplementary file 1 — Additional file 1. [file 13020_2025_1266_MOESM1_ESM.zip › Figure 9/bands/GAPDH-2 HC.Tif]

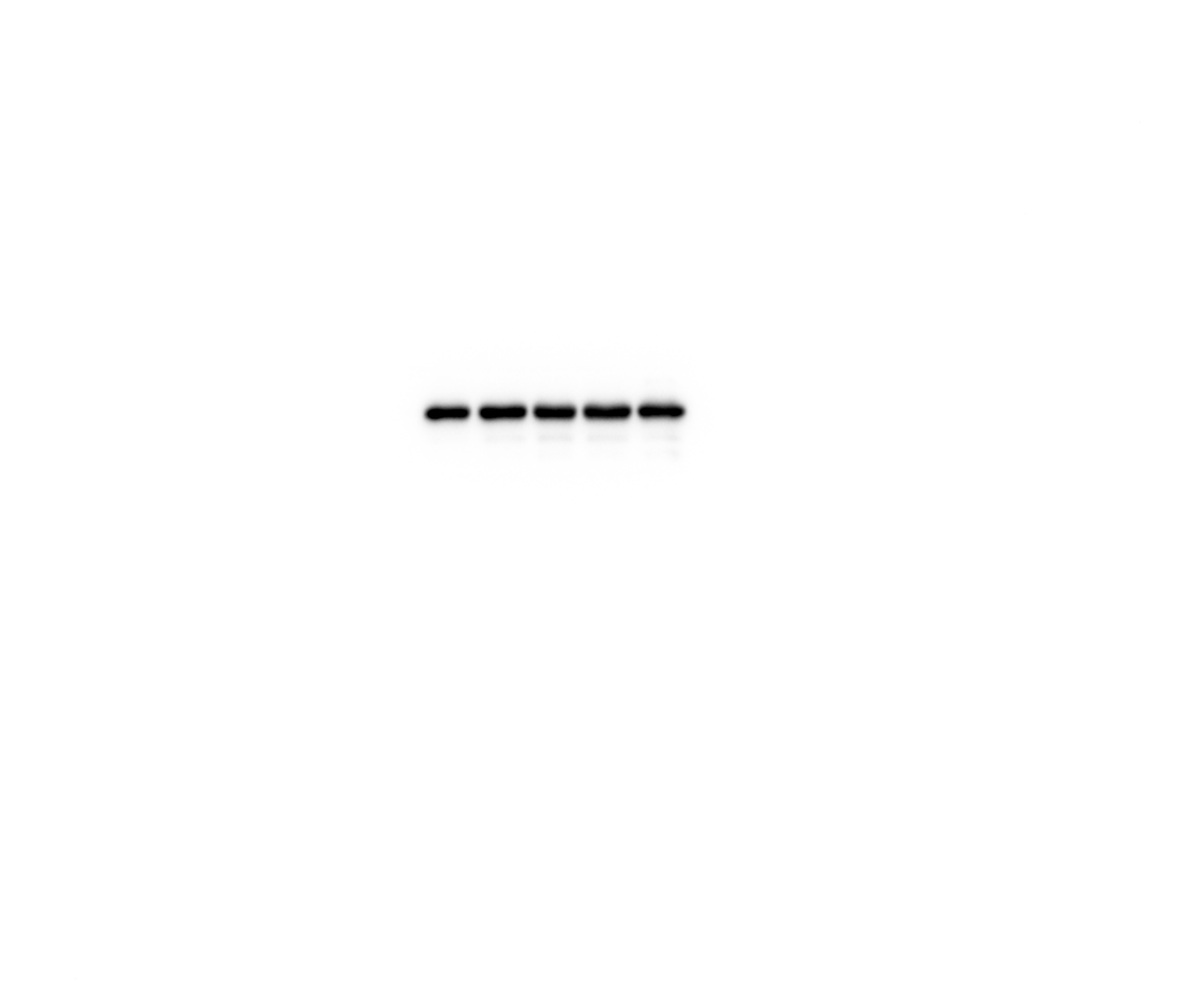

Supplement: Supplementary file 1 — Additional file 1. [file 13020_2025_1266_MOESM1_ESM.zip › Figure 9/bands/GAPDH-2.Tif]

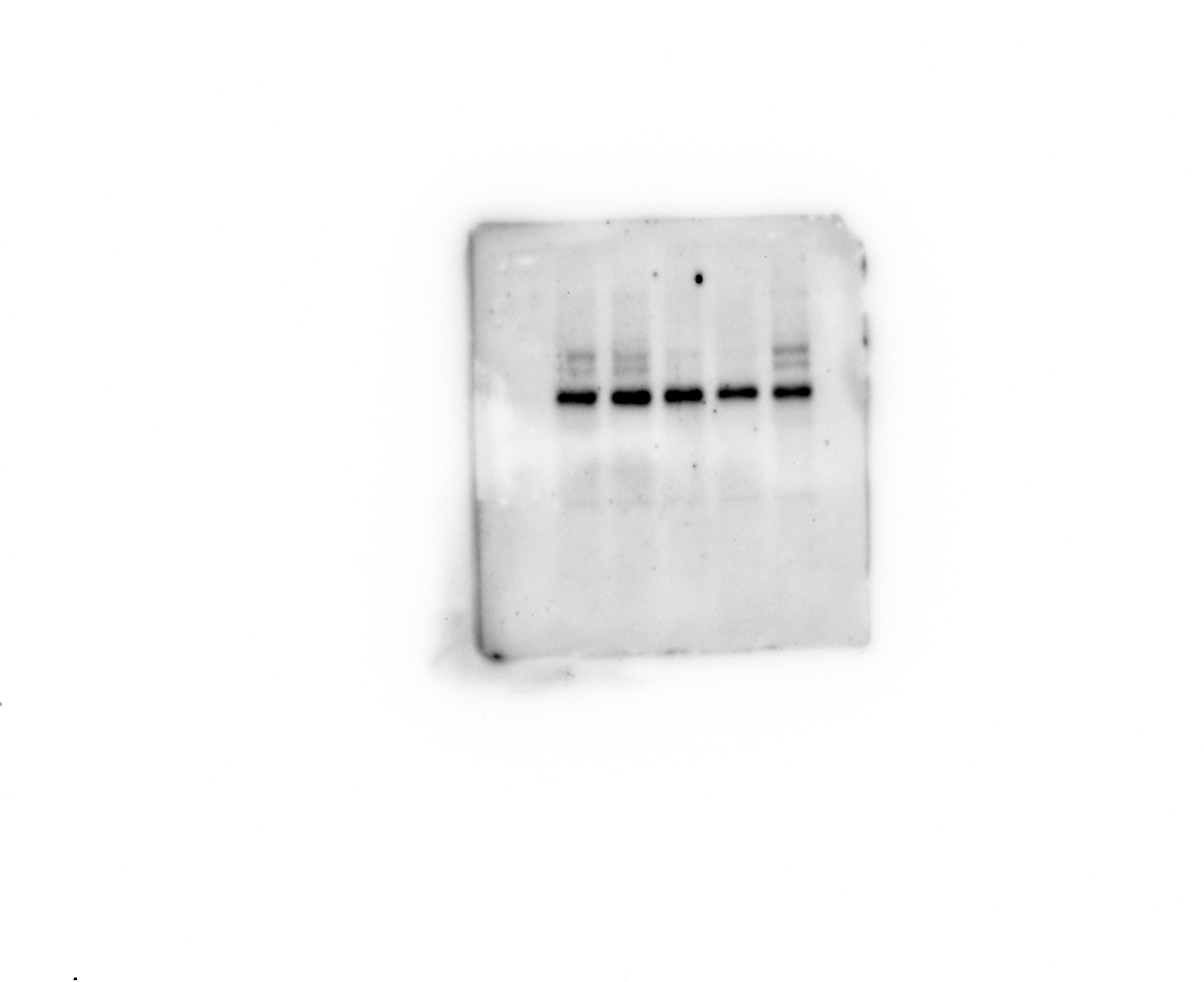

Supplement: Supplementary file 1 — Additional file 1. [file 13020_2025_1266_MOESM1_ESM.zip › Figure 9/bands/HIF-1 (Figure).Tif]

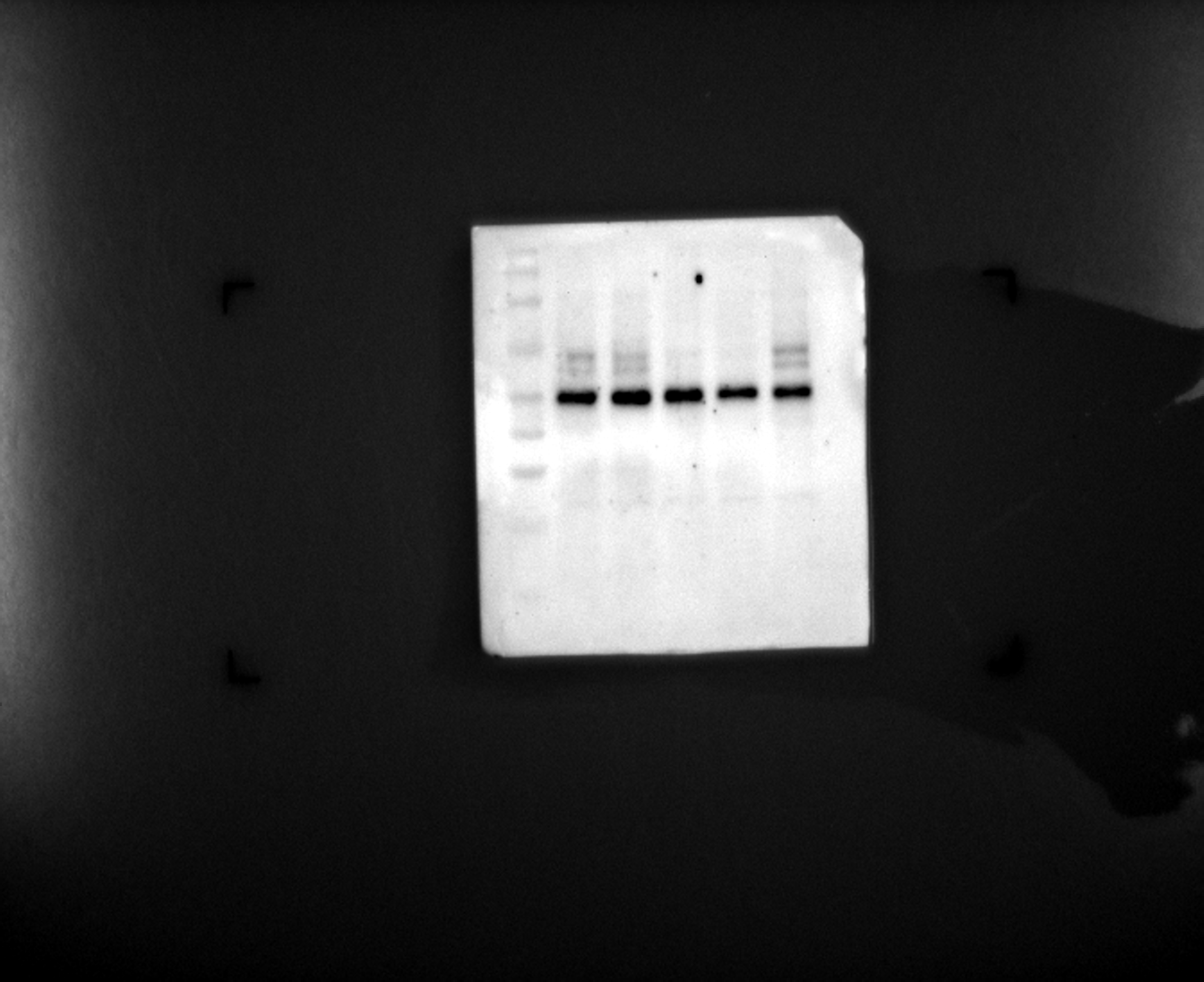

Supplement: Supplementary file 1 — Additional file 1. [file 13020_2025_1266_MOESM1_ESM.zip › Figure 9/bands/HIF-1(Figure) HC.Tif]

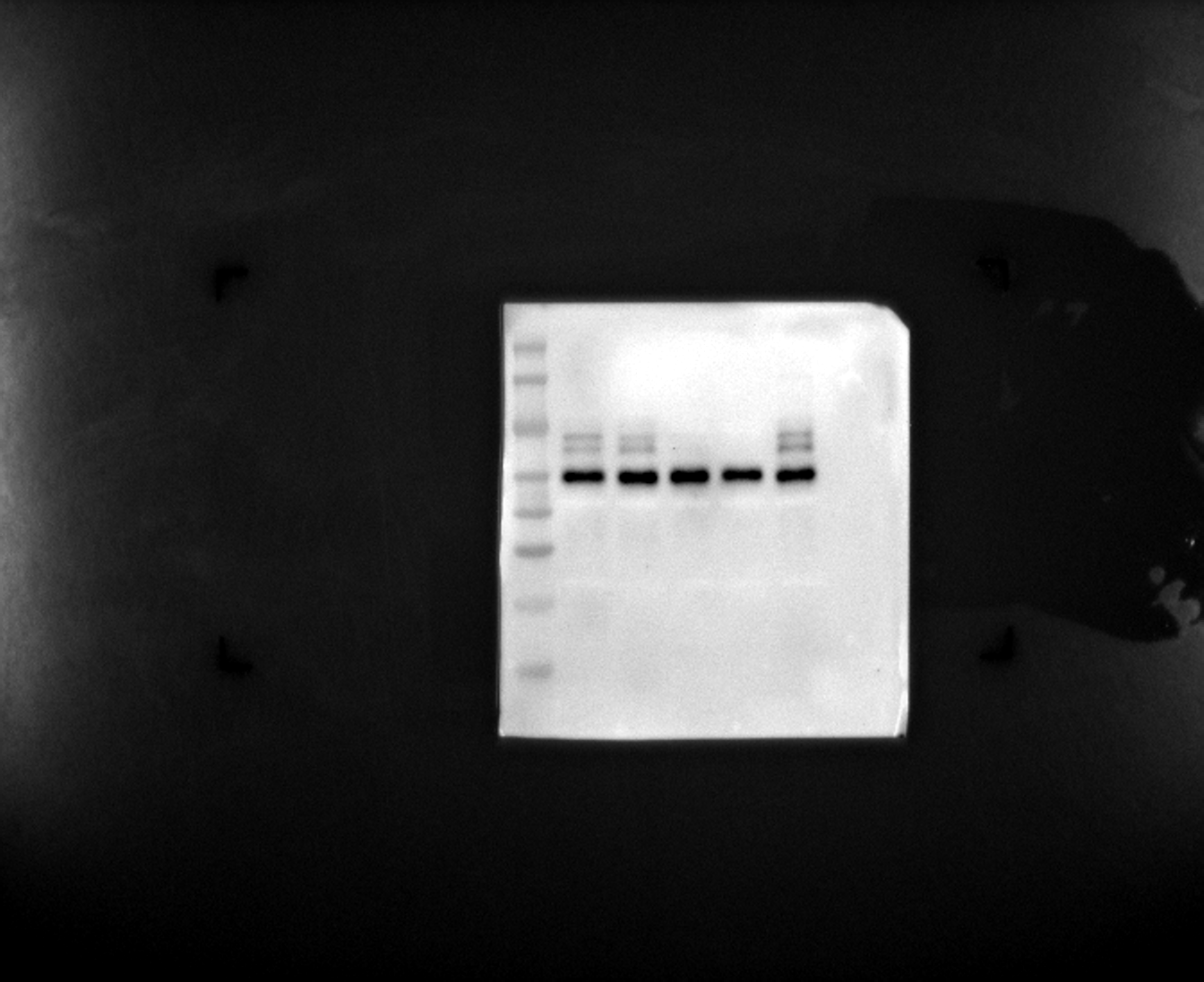

Supplement: Supplementary file 1 — Additional file 1. [file 13020_2025_1266_MOESM1_ESM.zip › Figure 9/bands/HIF-2 HC.Tif]

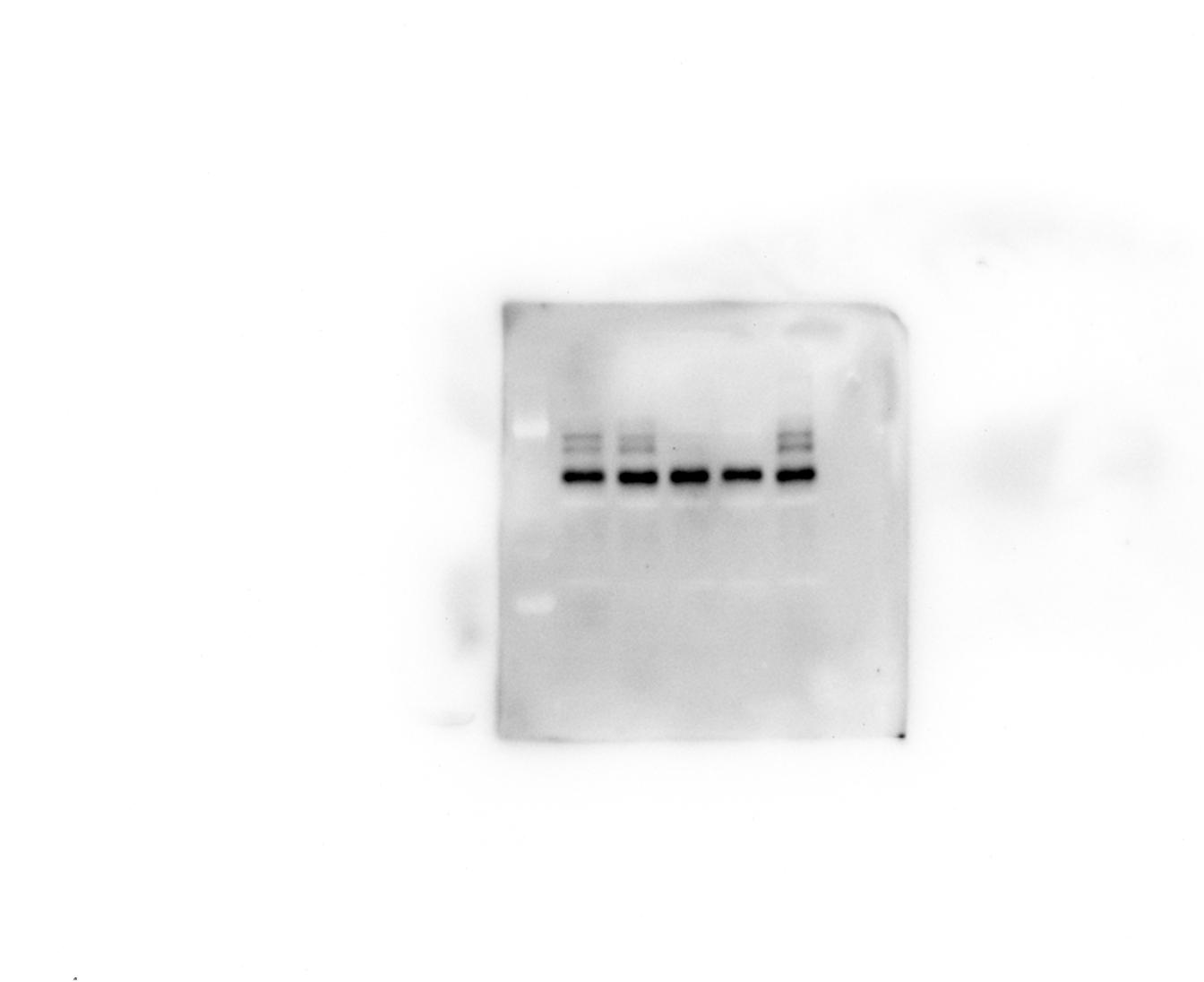

Supplement: Supplementary file 1 — Additional file 1. [file 13020_2025_1266_MOESM1_ESM.zip › Figure 9/bands/HIF-2.Tif]

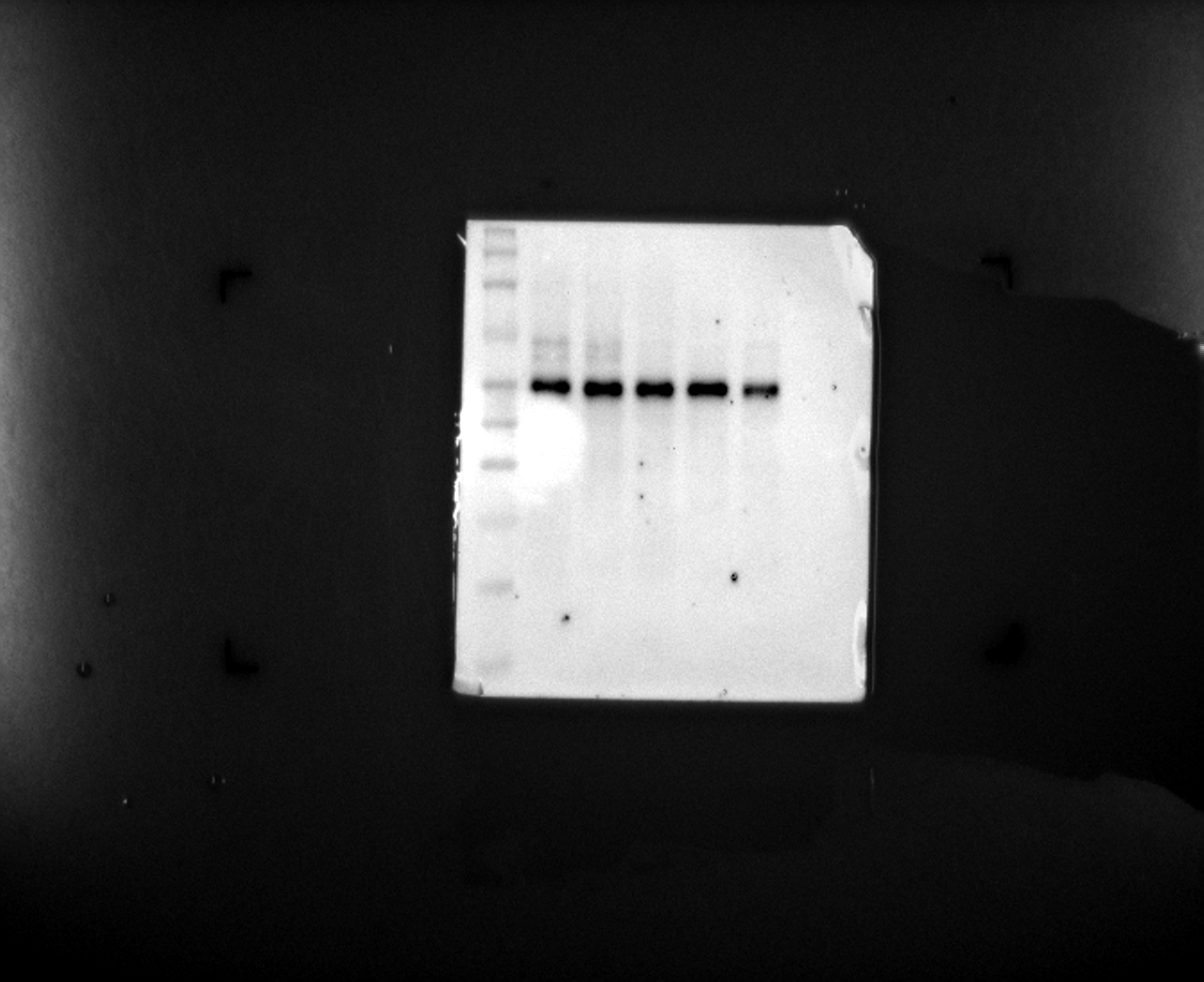

Supplement: Supplementary file 1 — Additional file 1. [file 13020_2025_1266_MOESM1_ESM.zip › Figure 9/bands/HIF-3 HC.Tif]

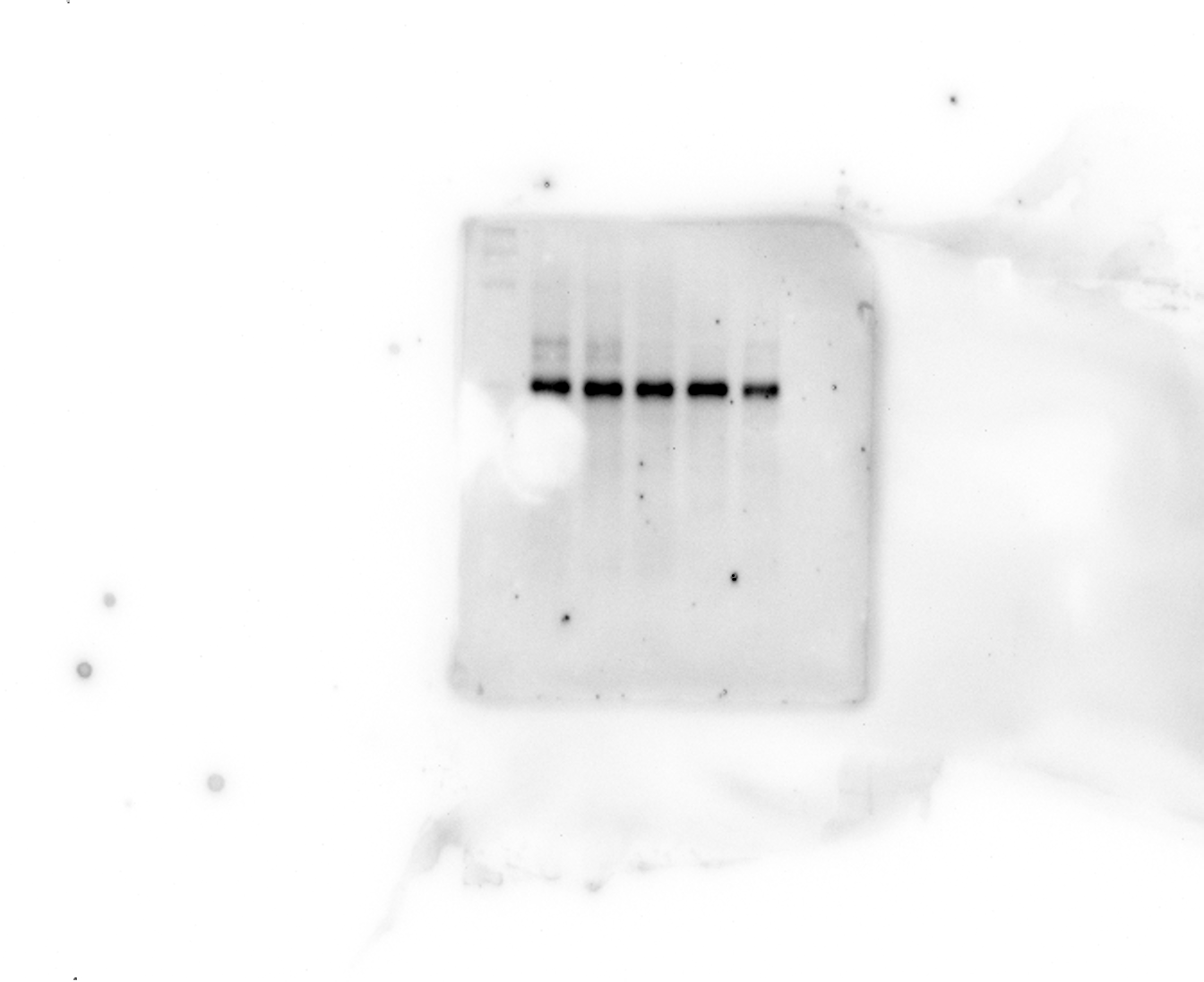

Supplement: Supplementary file 1 — Additional file 1. [file 13020_2025_1266_MOESM1_ESM.zip › Figure 9/bands/HIF-3.Tif]

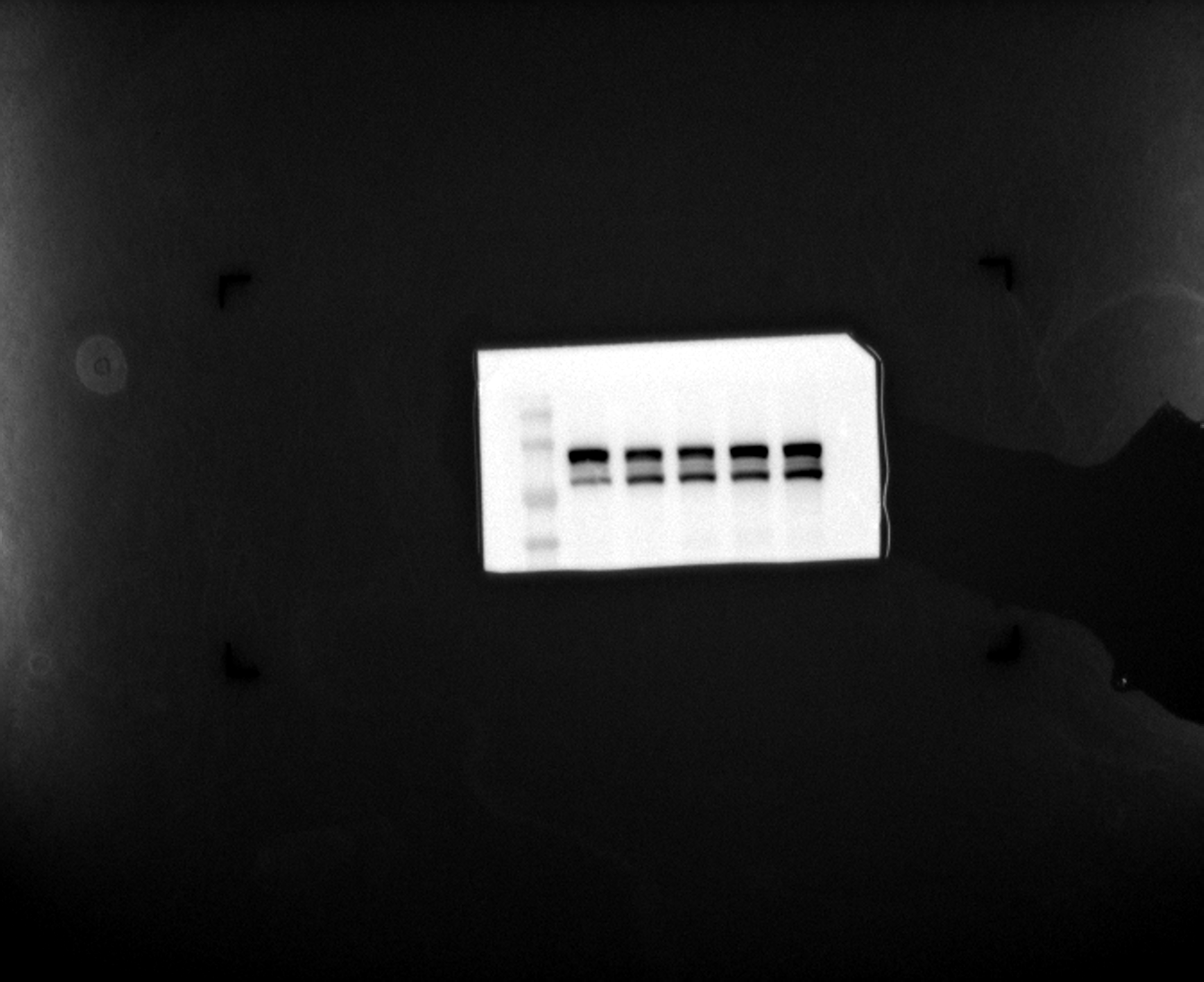

Supplement: Supplementary file 1 — Additional file 1. [file 13020_2025_1266_MOESM1_ESM.zip › Figure 9/bands/p-STAT5 1(Figure) HC.Tif]

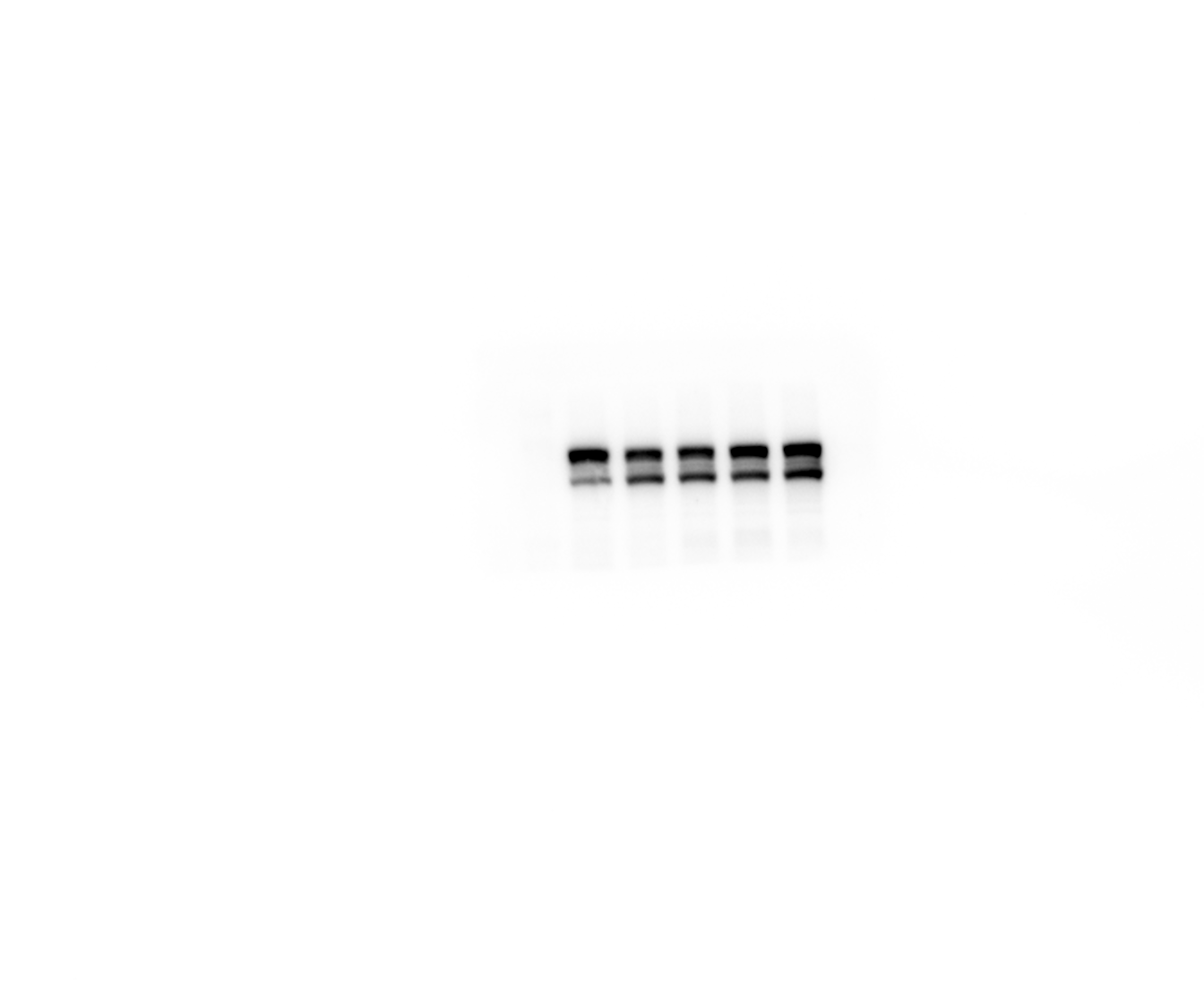

Supplement: Supplementary file 1 — Additional file 1. [file 13020_2025_1266_MOESM1_ESM.zip › Figure 9/bands/p-STAT5 1(Figure).Tif]

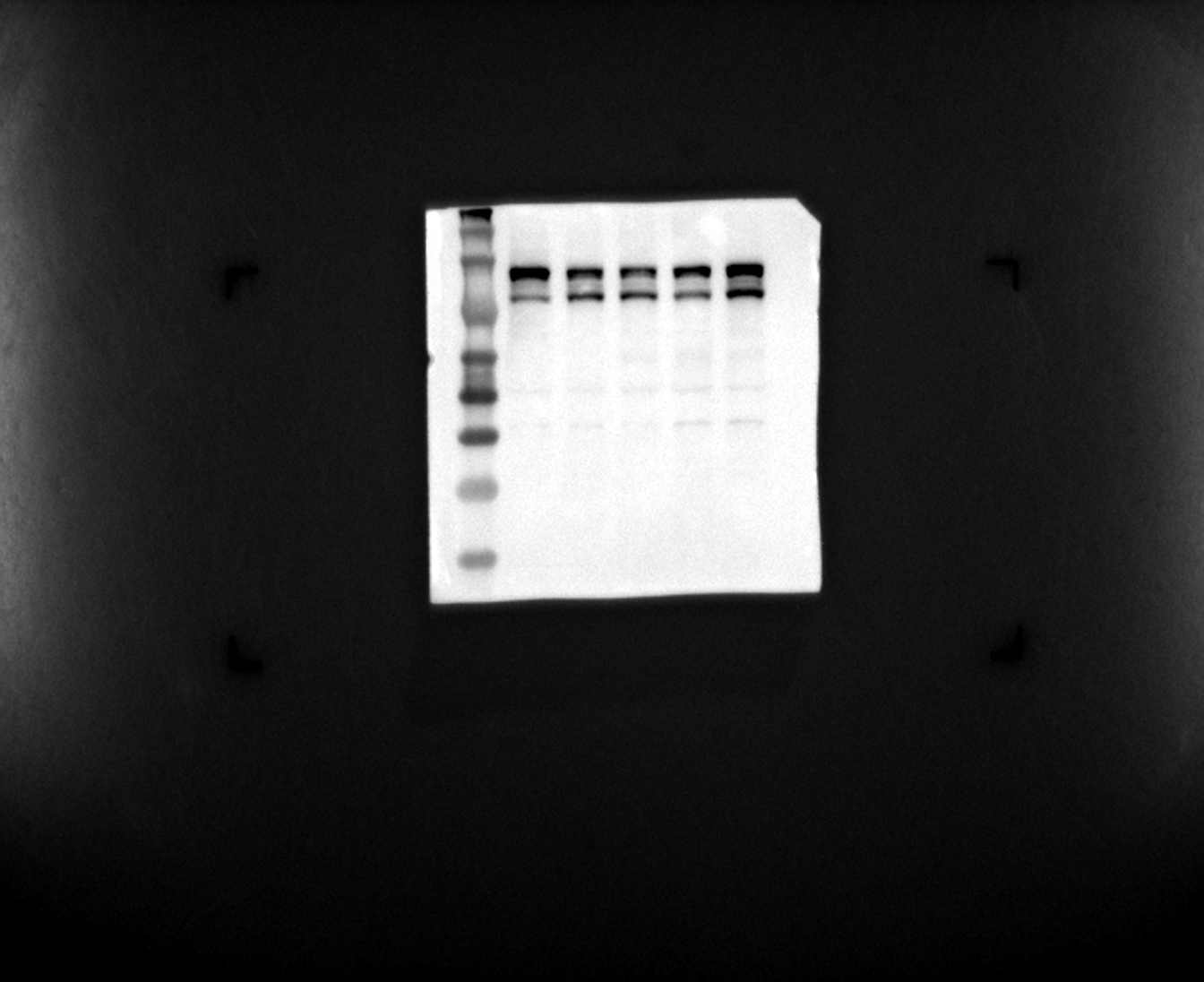

Supplement: Supplementary file 1 — Additional file 1. [file 13020_2025_1266_MOESM1_ESM.zip › Figure 9/bands/p-STAT5 2 HC.Tif]

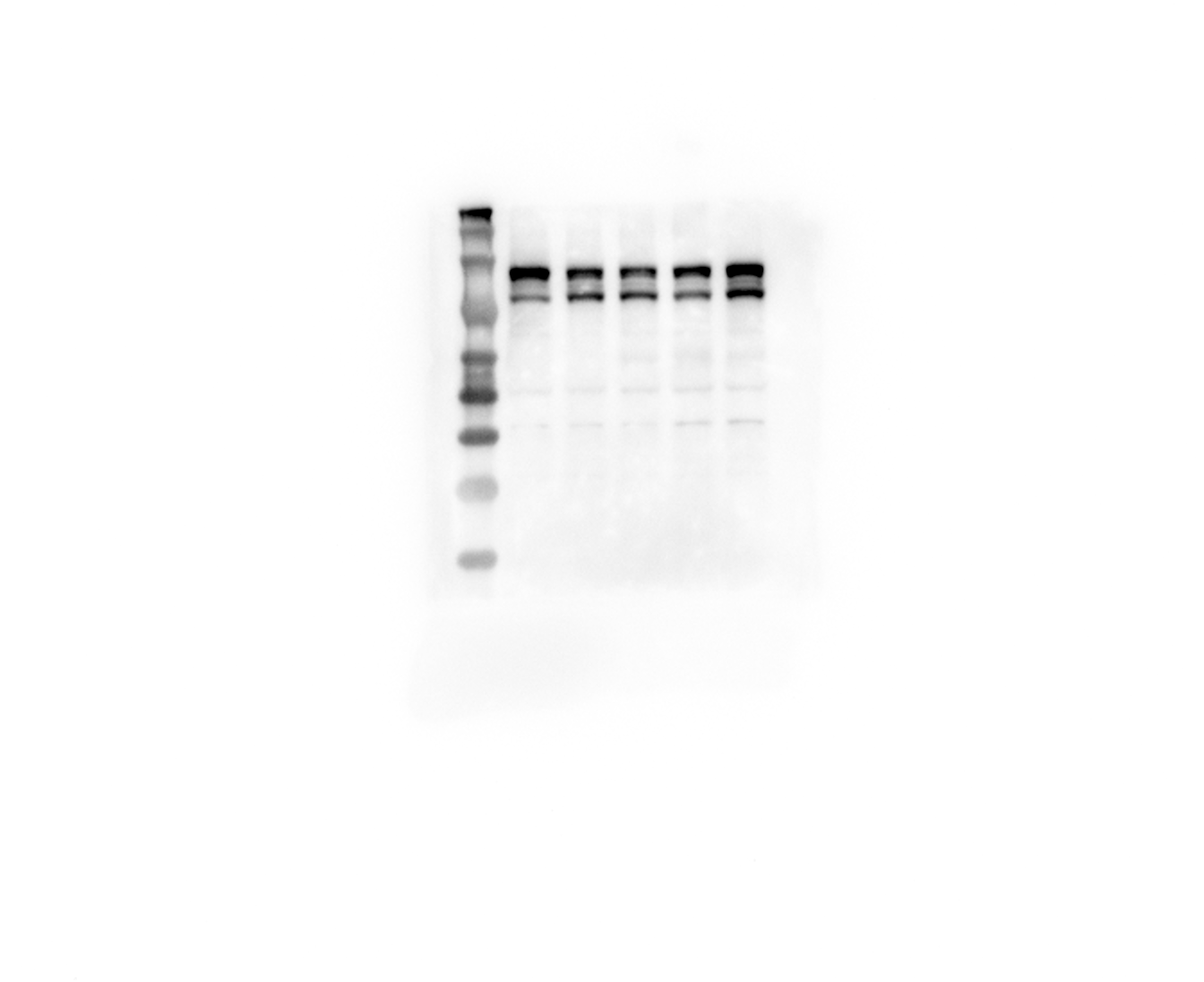

Supplement: Supplementary file 1 — Additional file 1. [file 13020_2025_1266_MOESM1_ESM.zip › Figure 9/bands/p-STAT5 2.Tif]

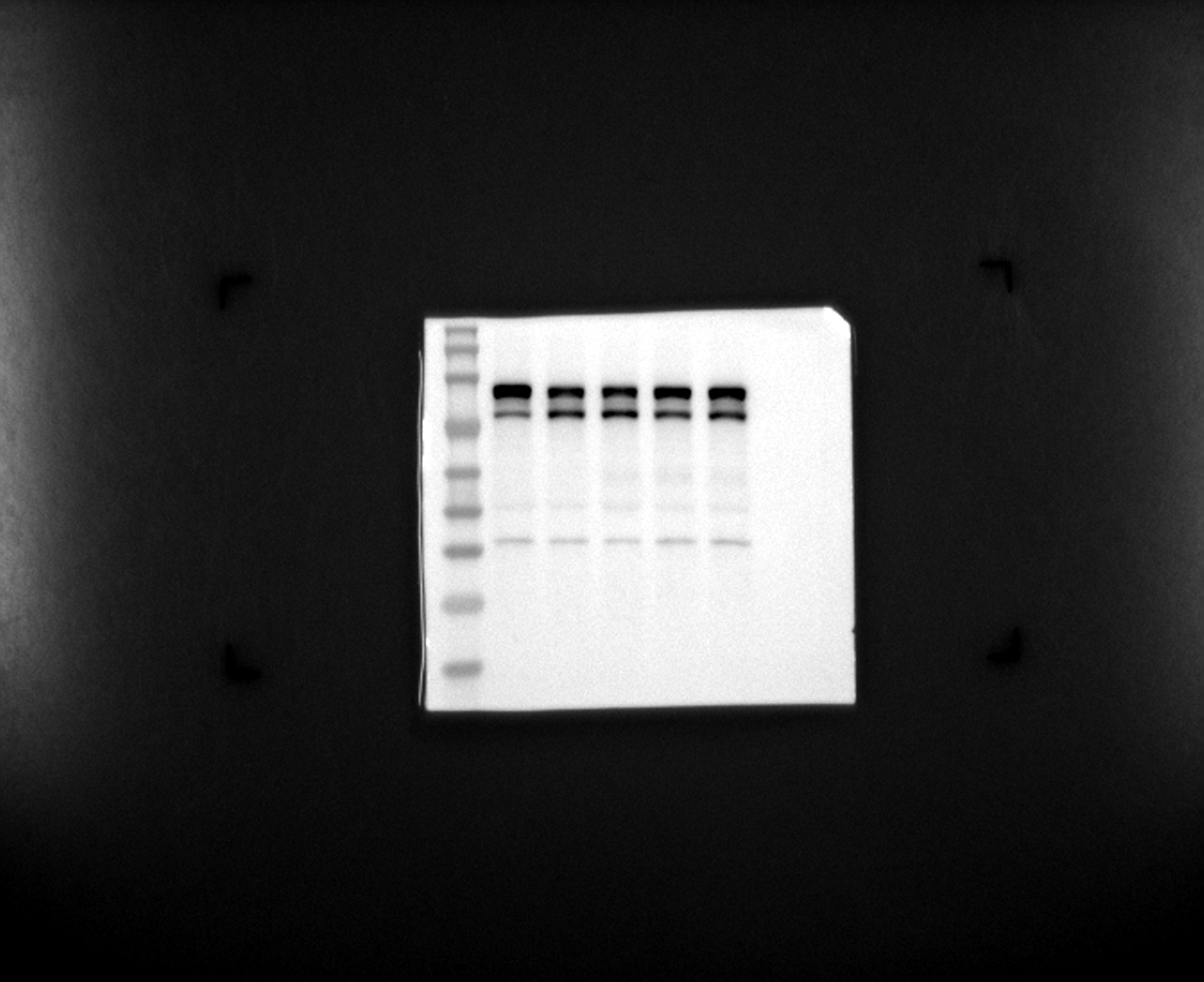

Supplement: Supplementary file 1 — Additional file 1. [file 13020_2025_1266_MOESM1_ESM.zip › Figure 9/bands/p-STAT5 3 HC.Tif]
