# Supplementary figures and images for: Modified Guilu Erxian Glue regulates Treg immune function to suppress bone marrow failure in aplastic anemia mice (part 1 of 2)
Source: Chin Med. 2025 Nov 20;20:197. doi: 10.1186/s13020-025-01266-z (PMC12632029; doi:10.1186/s13020-025-01266-z)

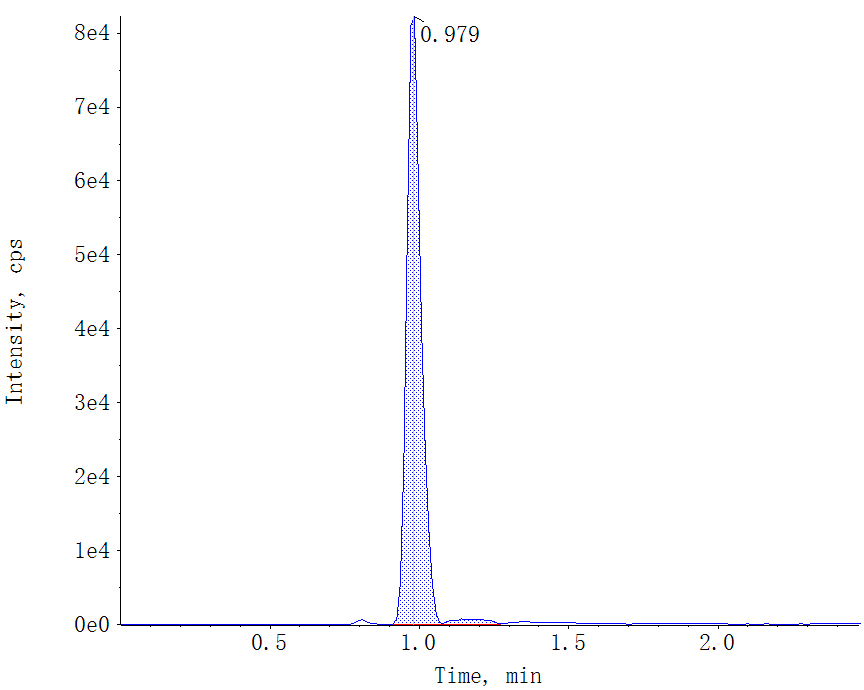

Supplement: Supplementary file 1 — Additional file 1. [file 13020_2025_1266_MOESM1_ESM.zip › Figure 1/Amentoflavone-1.png]

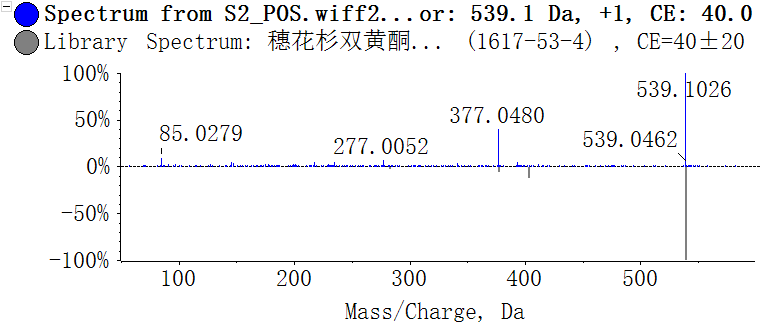

Supplement: Supplementary file 1 — Additional file 1. [file 13020_2025_1266_MOESM1_ESM.zip › Figure 1/Amentoflavone-2.png]

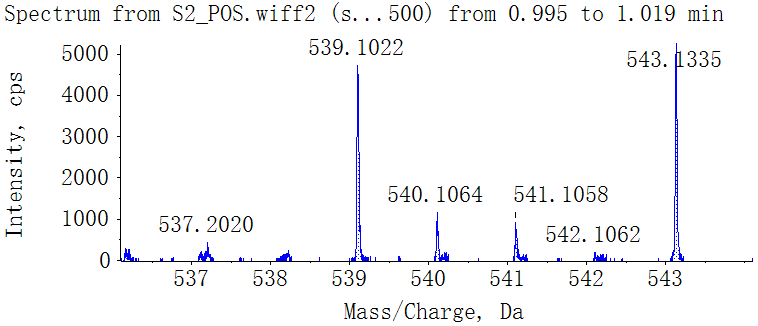

Supplement: Supplementary file 1 — Additional file 1. [file 13020_2025_1266_MOESM1_ESM.zip › Figure 1/Amentoflavone-3.png]

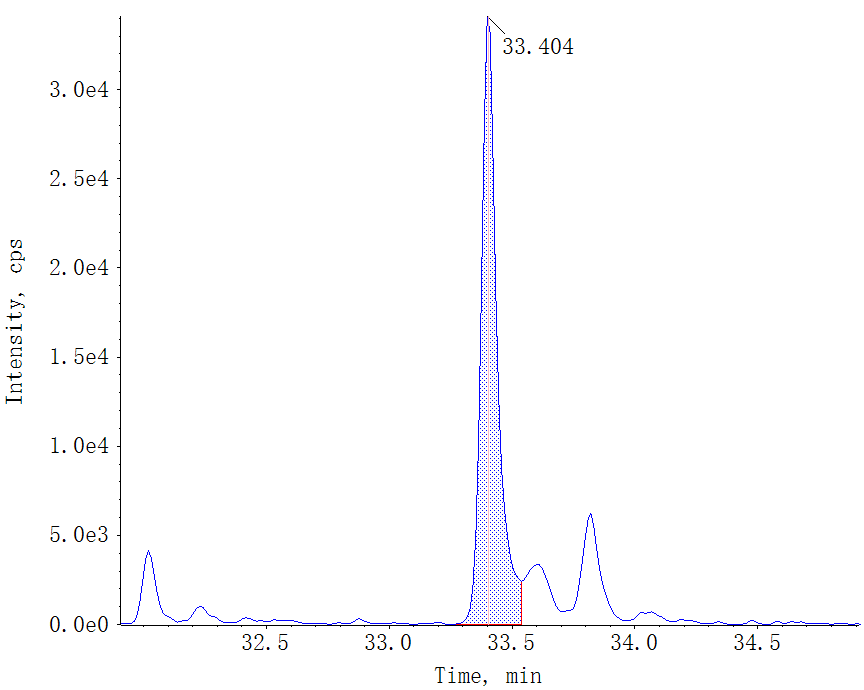

Supplement: Supplementary file 1 — Additional file 1. [file 13020_2025_1266_MOESM1_ESM.zip › Figure 1/Berberine-1.png]

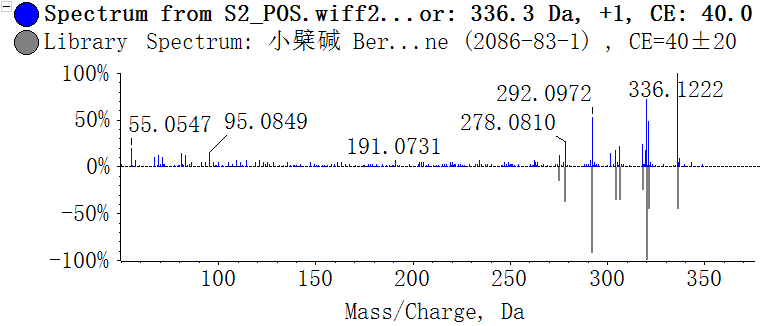

Supplement: Supplementary file 1 — Additional file 1. [file 13020_2025_1266_MOESM1_ESM.zip › Figure 1/Berberine-2.png]

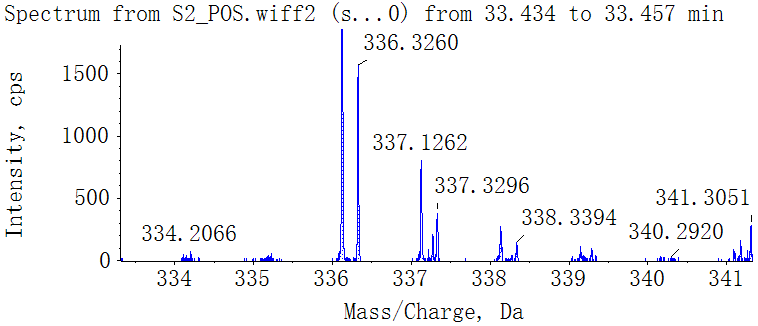

Supplement: Supplementary file 1 — Additional file 1. [file 13020_2025_1266_MOESM1_ESM.zip › Figure 1/Berberine-3.png]

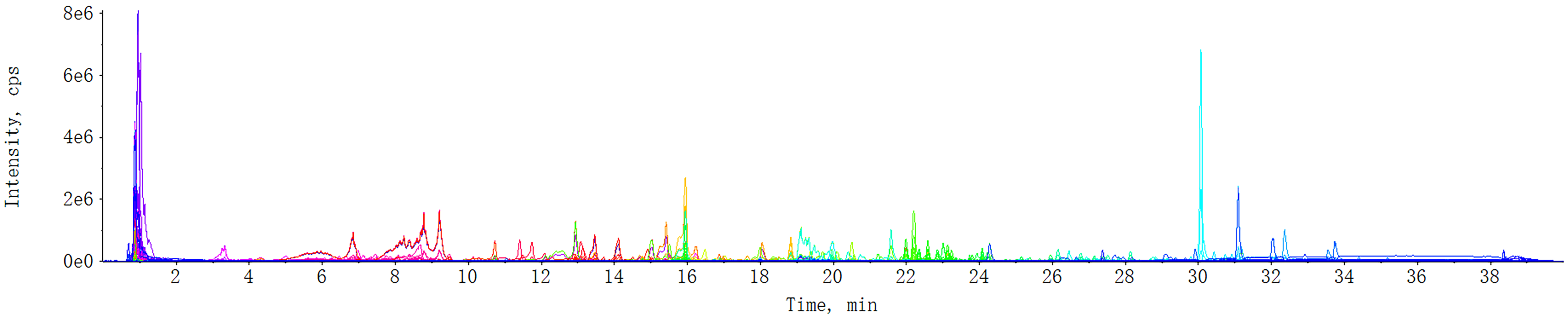

Supplement: Supplementary file 1 — Additional file 1. [file 13020_2025_1266_MOESM1_ESM.zip › Figure 1/Negative extract .tif]

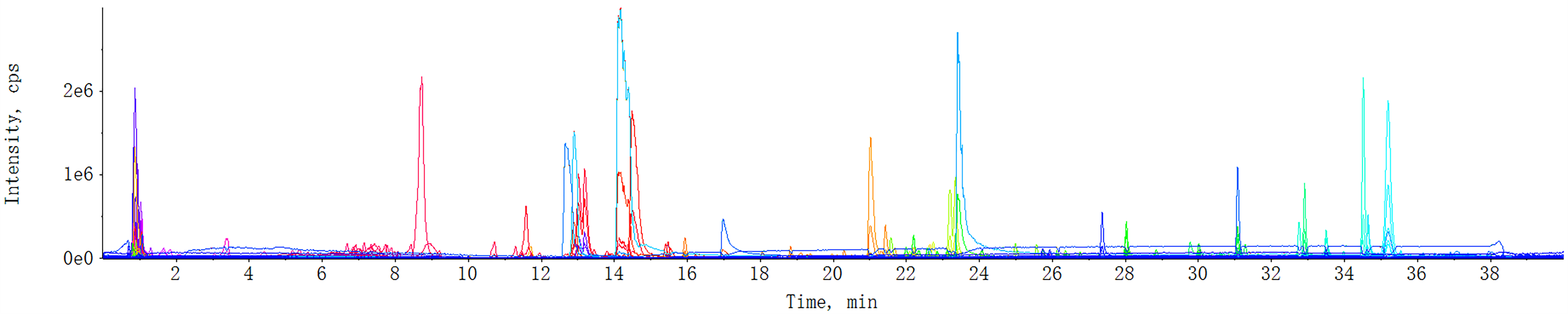

Supplement: Supplementary file 1 — Additional file 1. [file 13020_2025_1266_MOESM1_ESM.zip › Figure 1/Positive extract .tiff]

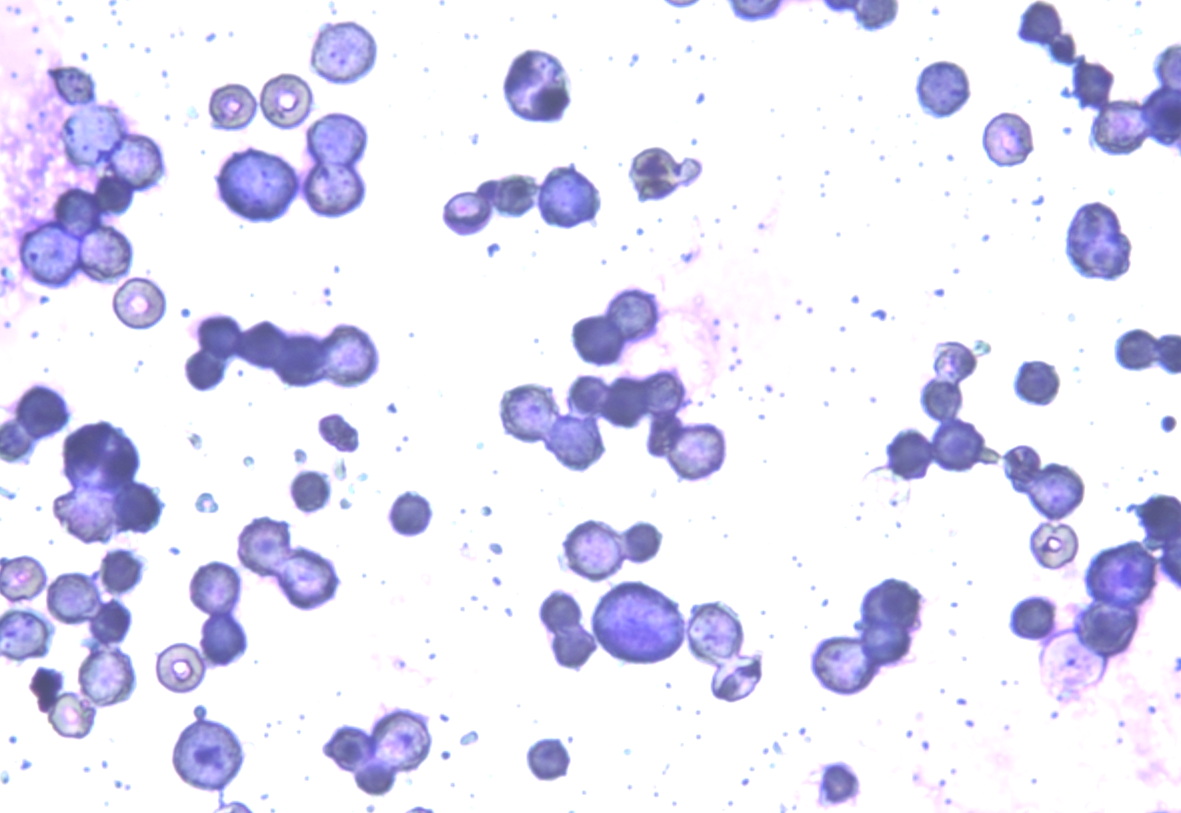

Supplement: Supplementary file 1 — Additional file 1. [file 13020_2025_1266_MOESM1_ESM.zip › Figure 2/Control A20-2.tif]

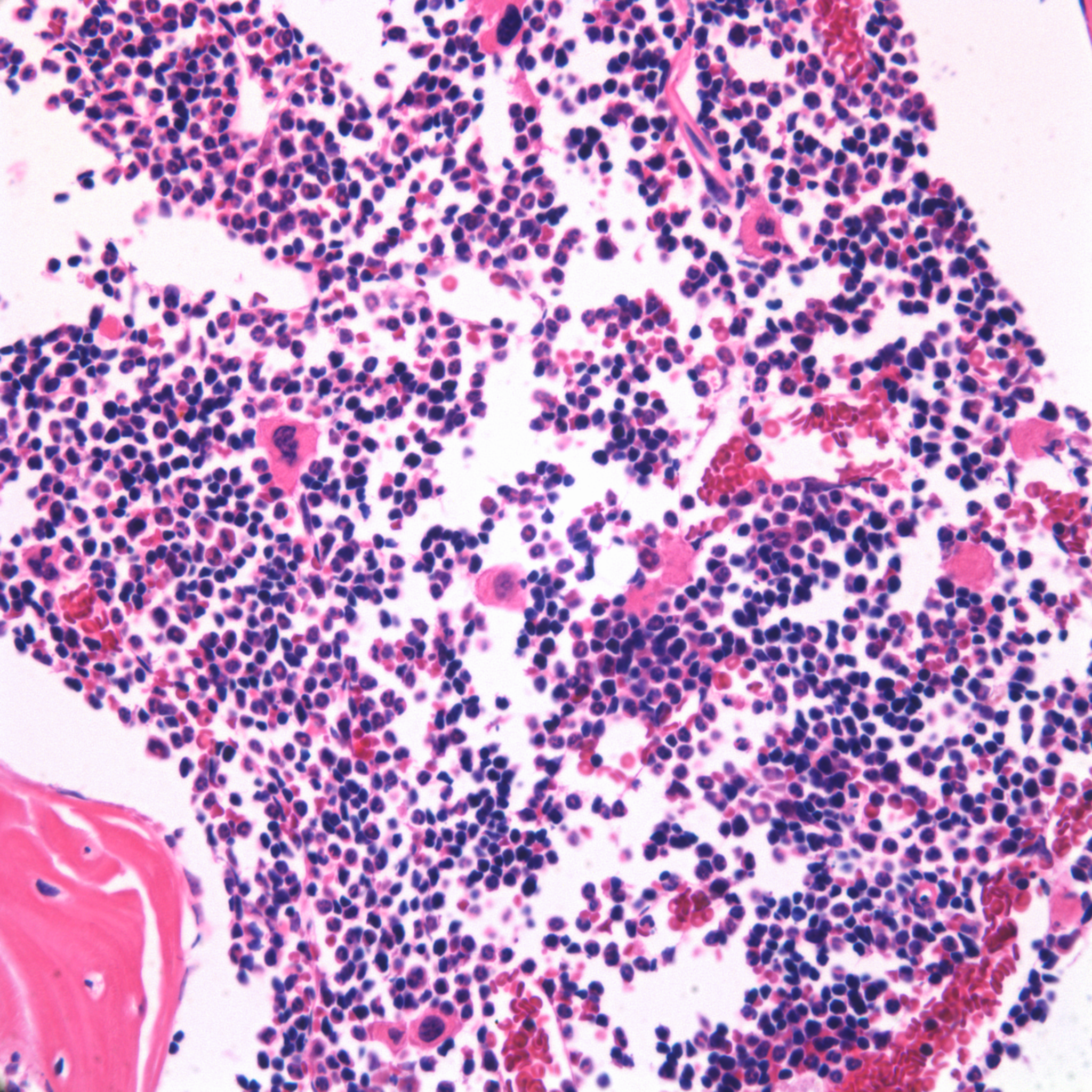

Supplement: Supplementary file 1 — Additional file 1. [file 13020_2025_1266_MOESM1_ESM.zip › Figure 2/Control-5 40X.tif]

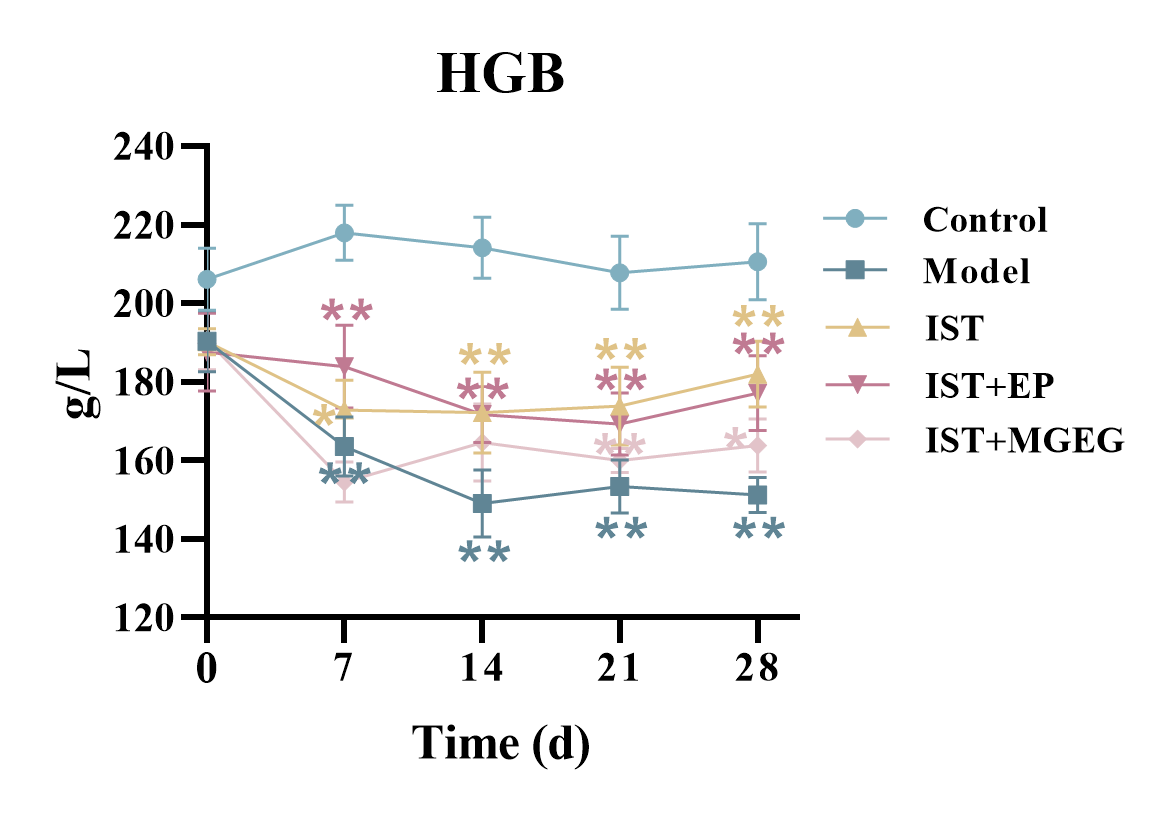

Supplement: Supplementary file 1 — Additional file 1. [file 13020_2025_1266_MOESM1_ESM.zip › Figure 2/HGB.tif]

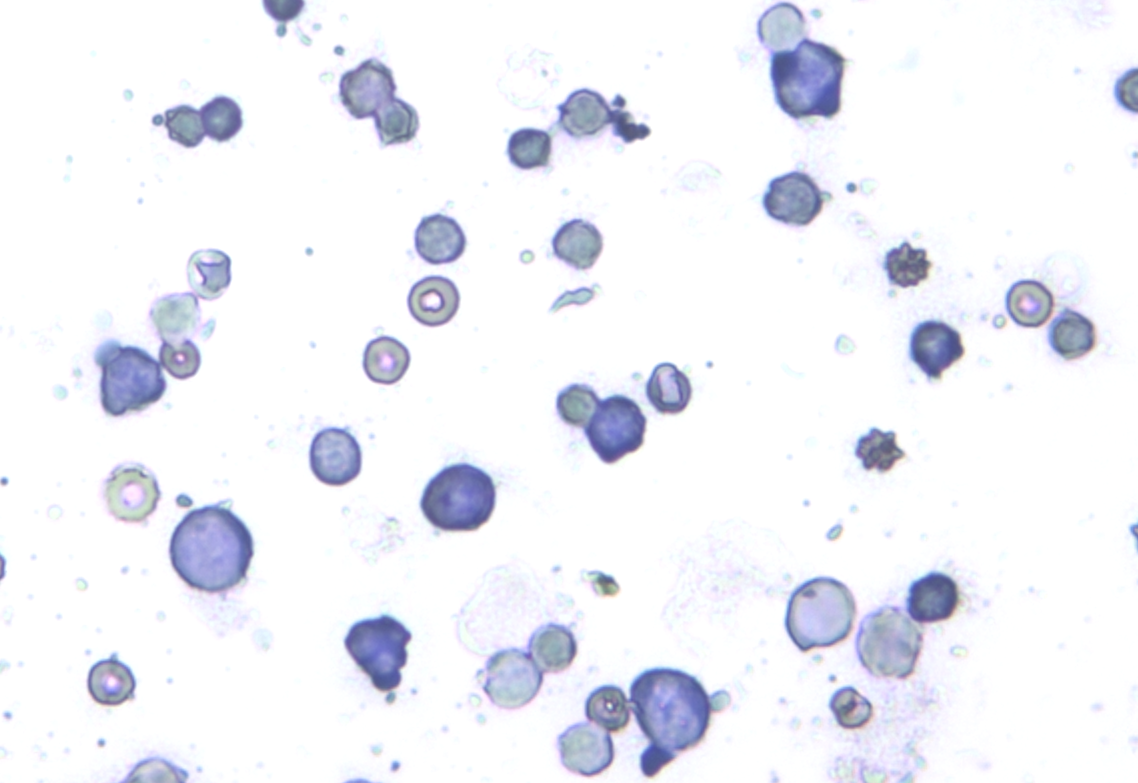

Supplement: Supplementary file 1 — Additional file 1. [file 13020_2025_1266_MOESM1_ESM.zip › Figure 2/IST A54-1.tif]

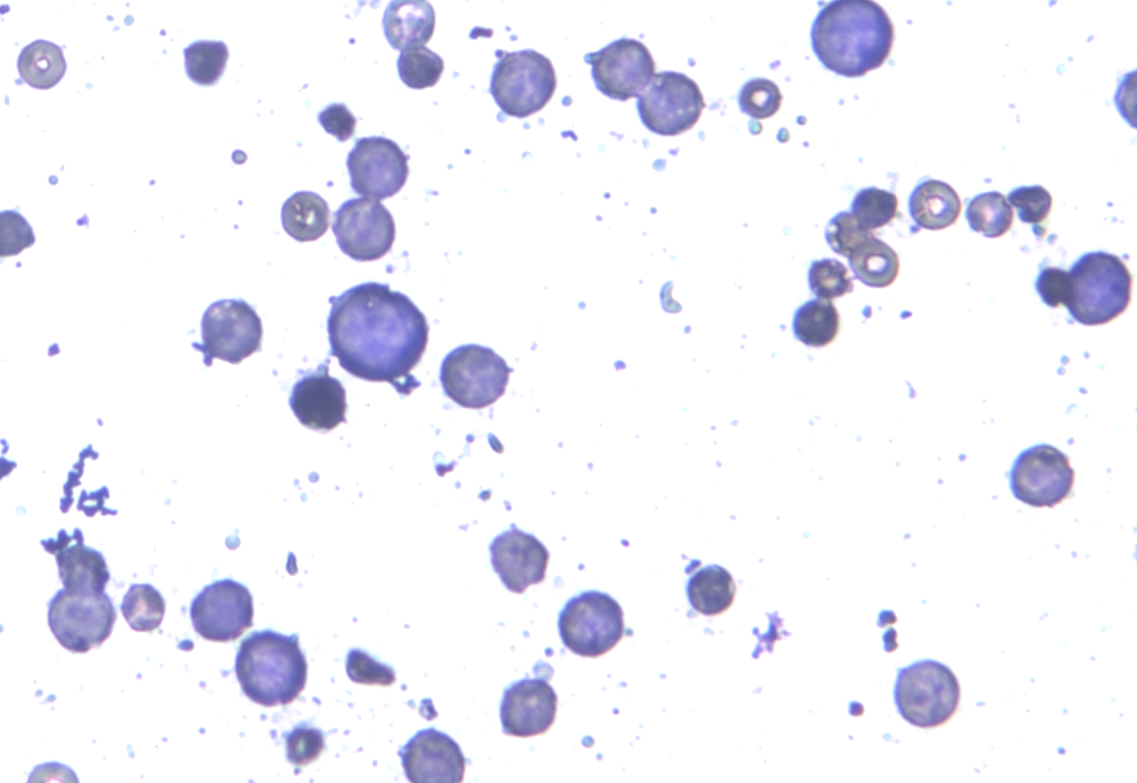

Supplement: Supplementary file 1 — Additional file 1. [file 13020_2025_1266_MOESM1_ESM.zip › Figure 2/IST+EP A75-1 .tif]

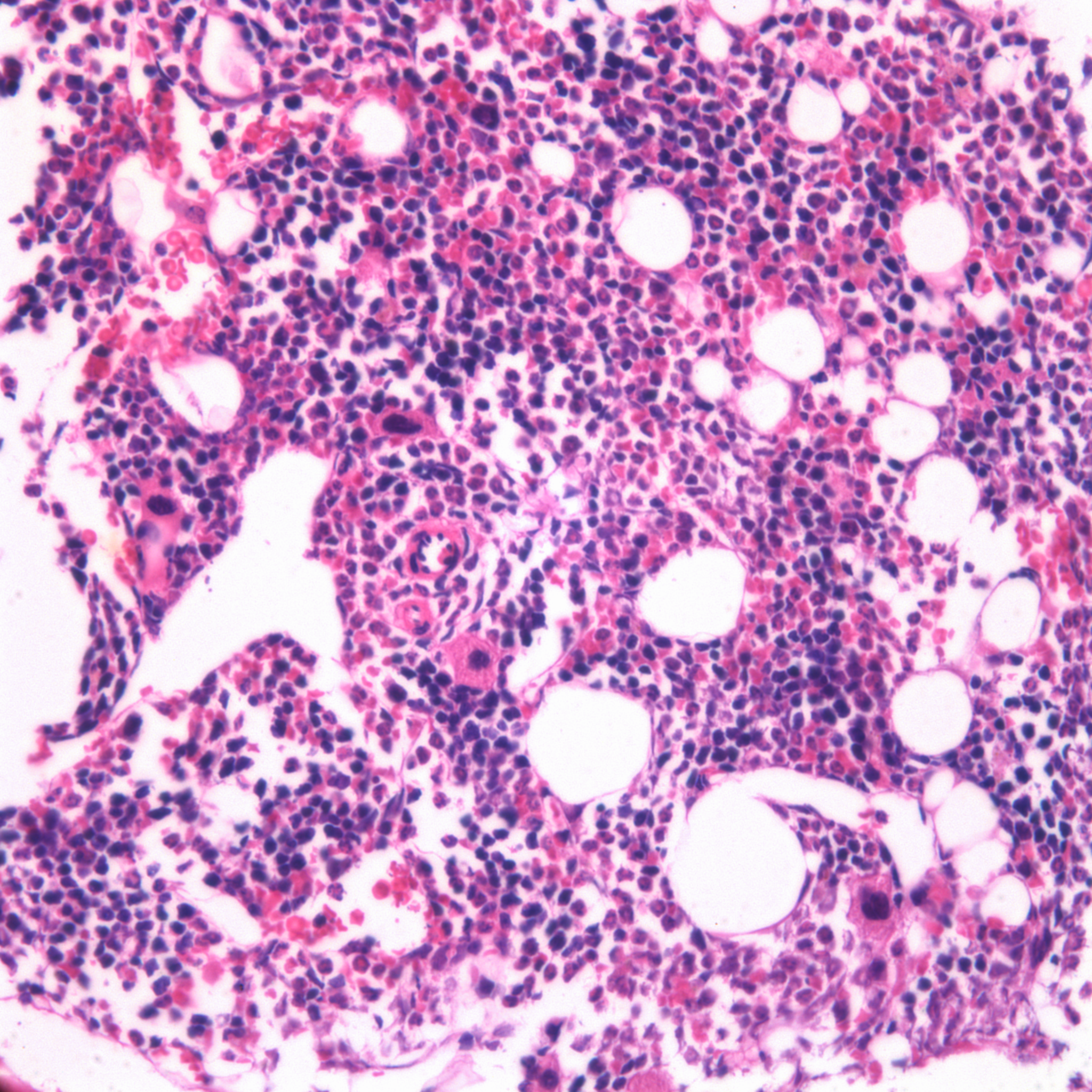

Supplement: Supplementary file 1 — Additional file 1. [file 13020_2025_1266_MOESM1_ESM.zip › Figure 2/IST+EP-1 40X.tif]

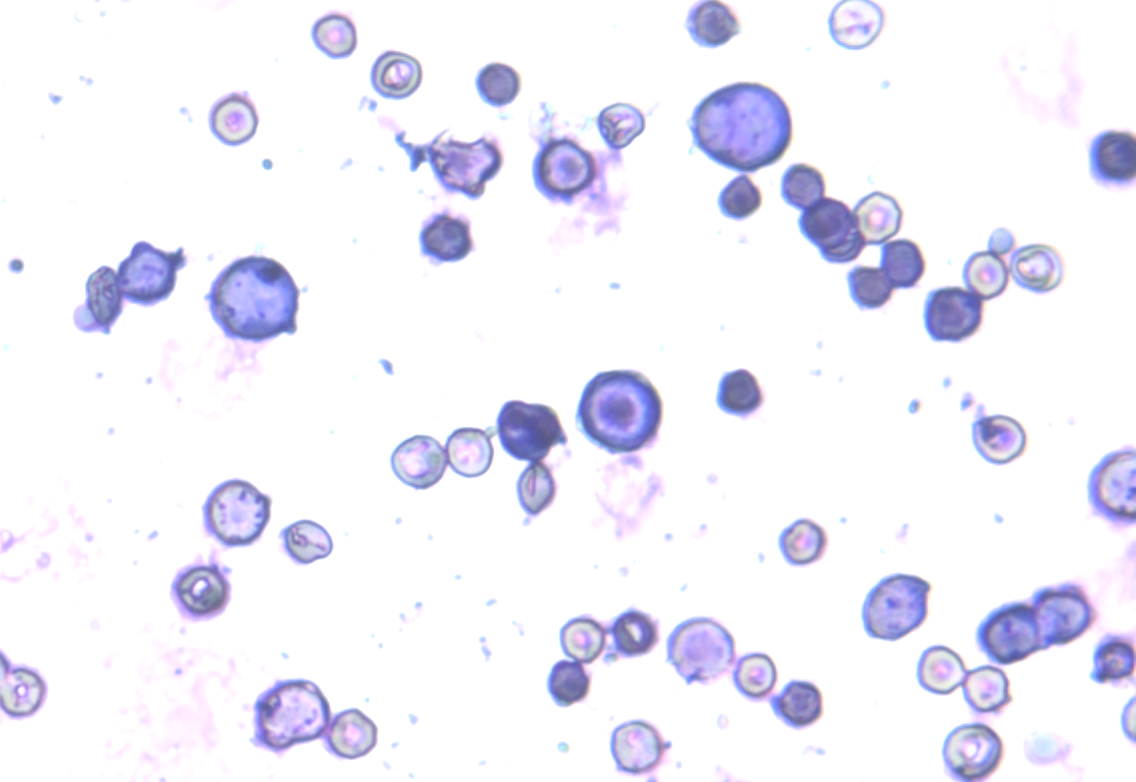

Supplement: Supplementary file 1 — Additional file 1. [file 13020_2025_1266_MOESM1_ESM.zip › Figure 2/IST+MGEG A98.tif]

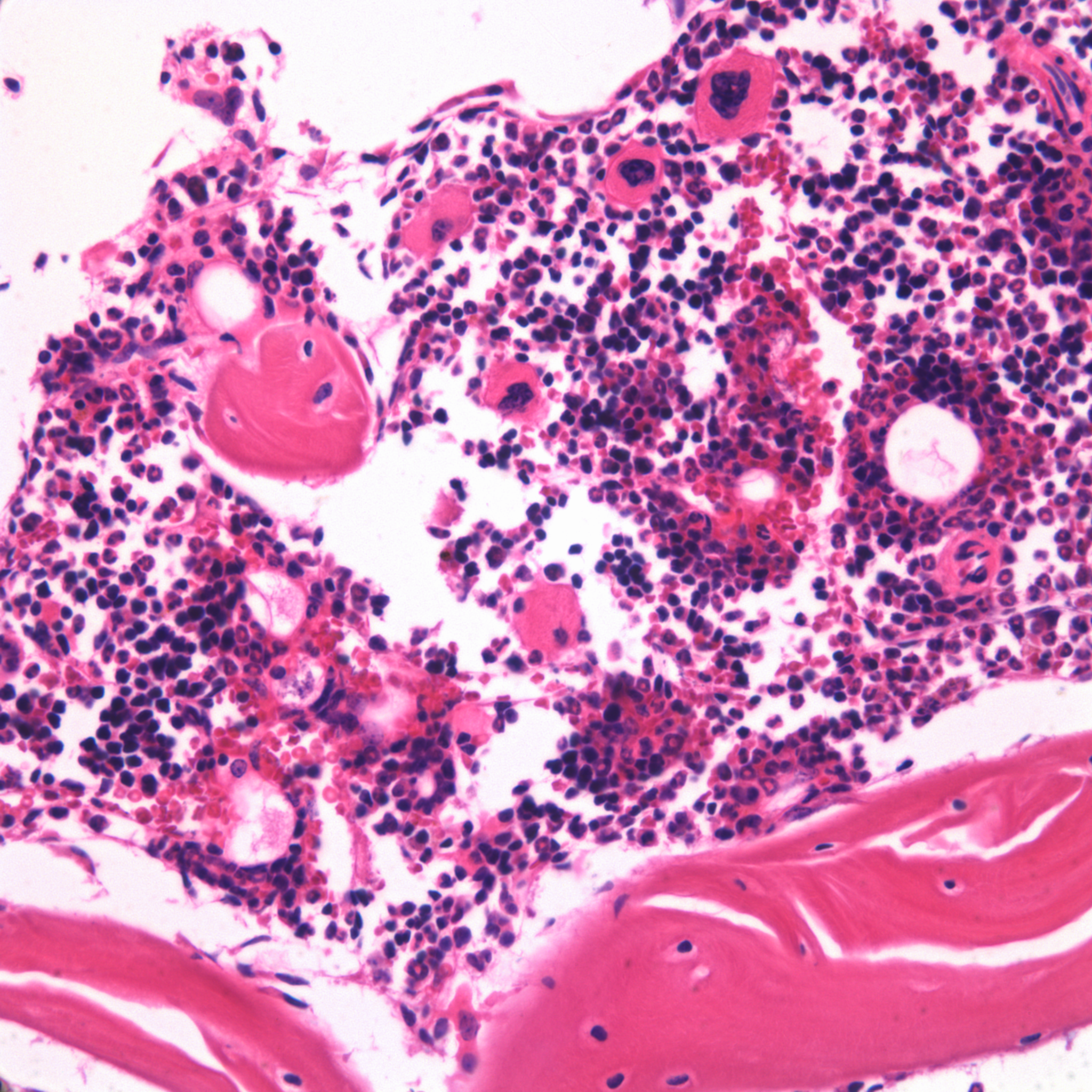

Supplement: Supplementary file 1 — Additional file 1. [file 13020_2025_1266_MOESM1_ESM.zip › Figure 2/IST+MGEG-4 40X.tif]

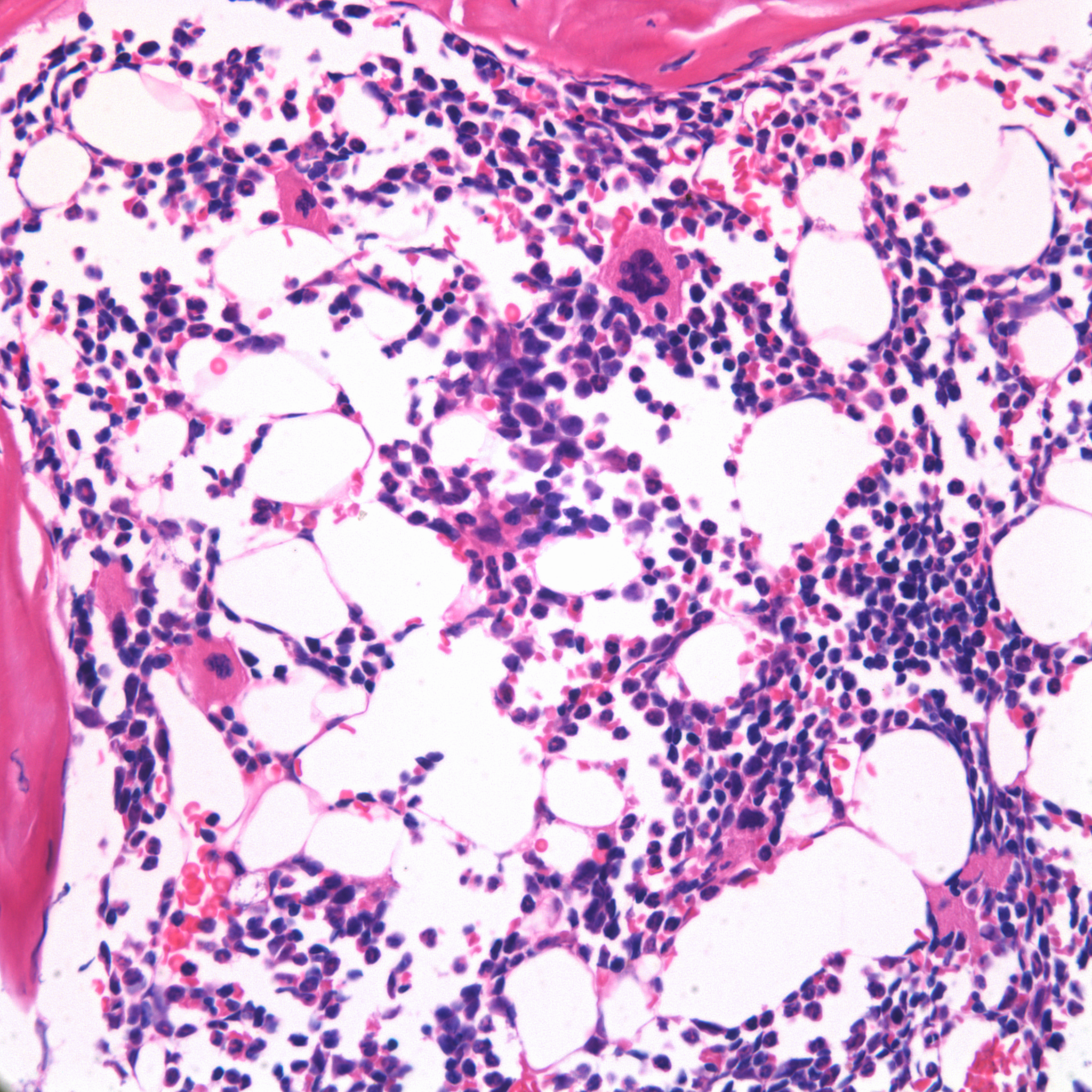

Supplement: Supplementary file 1 — Additional file 1. [file 13020_2025_1266_MOESM1_ESM.zip › Figure 2/IST-1 40X.tif]

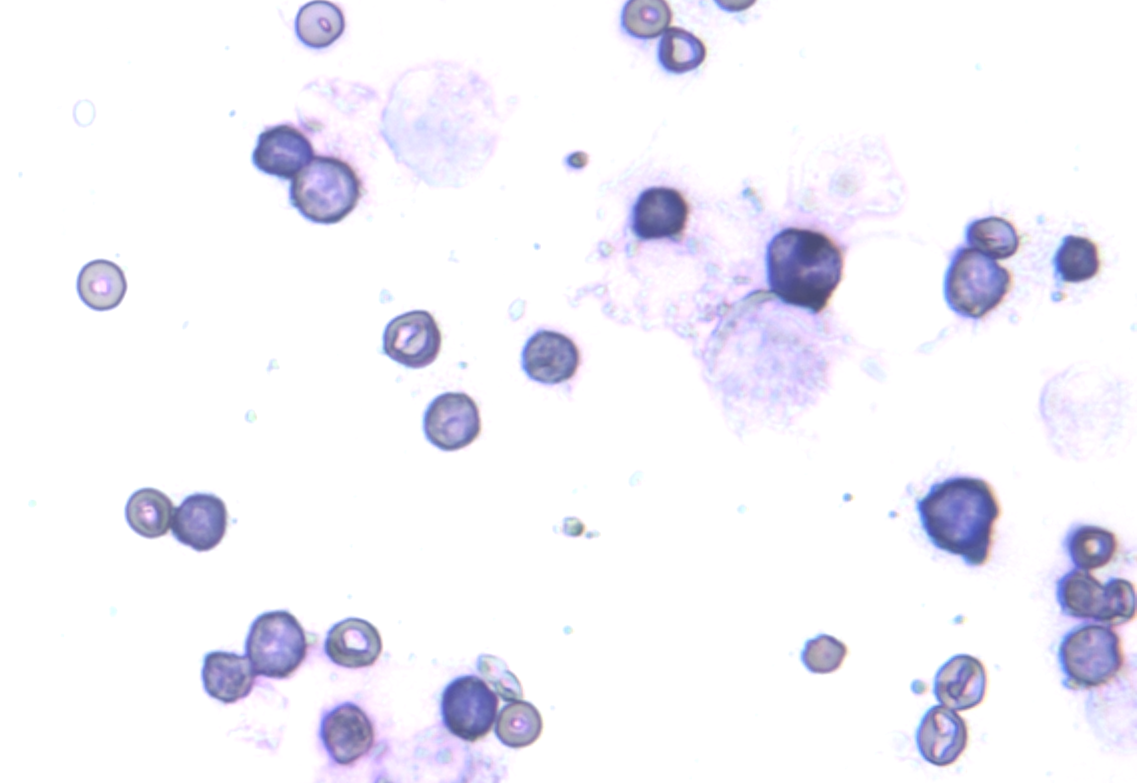

Supplement: Supplementary file 1 — Additional file 1. [file 13020_2025_1266_MOESM1_ESM.zip › Figure 2/Model A32-3 .tif]

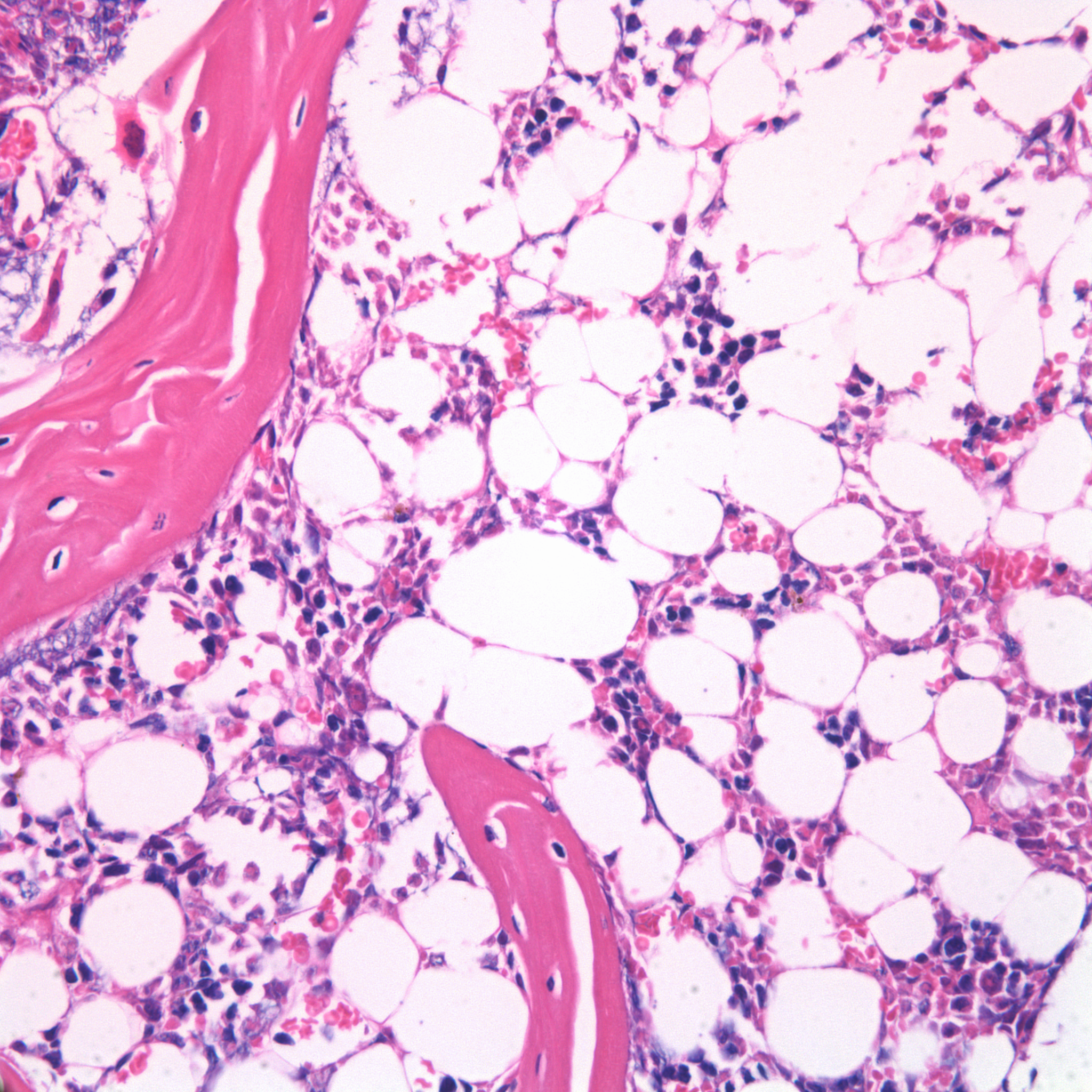

Supplement: Supplementary file 1 — Additional file 1. [file 13020_2025_1266_MOESM1_ESM.zip › Figure 2/Model-1 40X.tif]

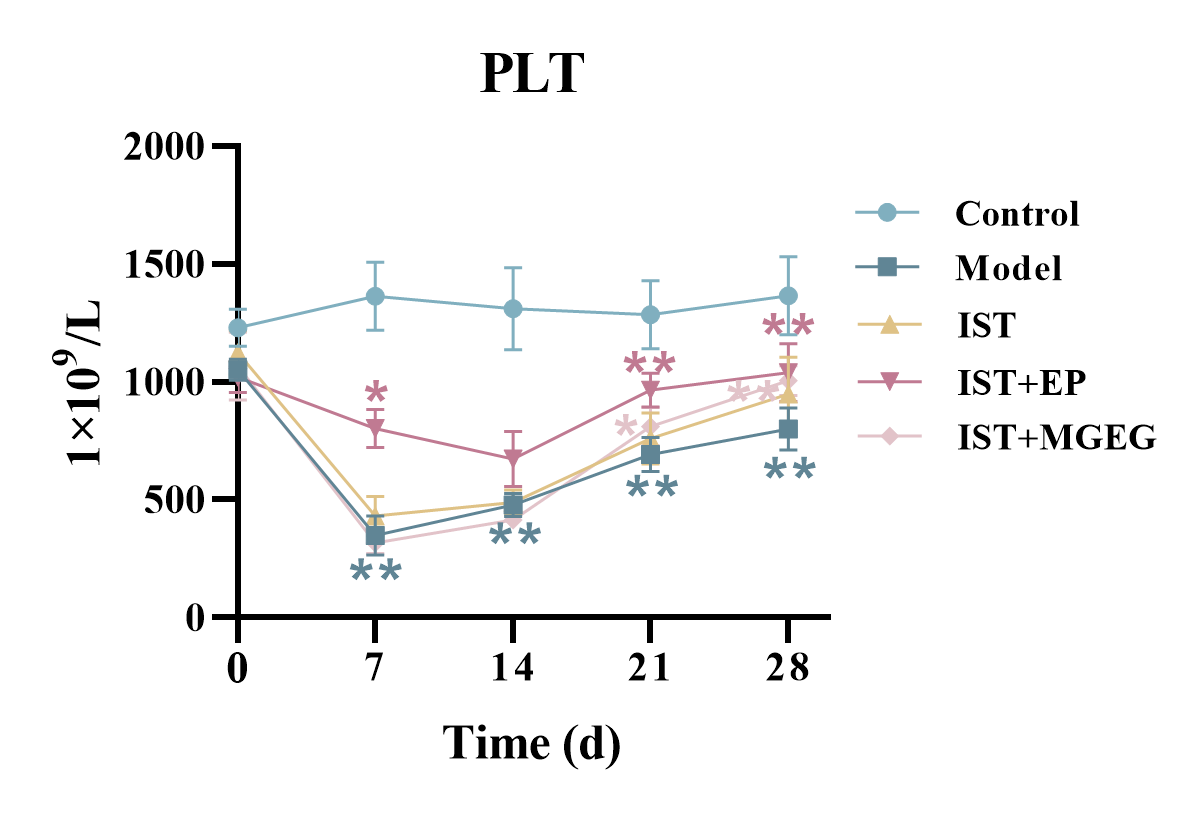

Supplement: Supplementary file 1 — Additional file 1. [file 13020_2025_1266_MOESM1_ESM.zip › Figure 2/PLT.tif]

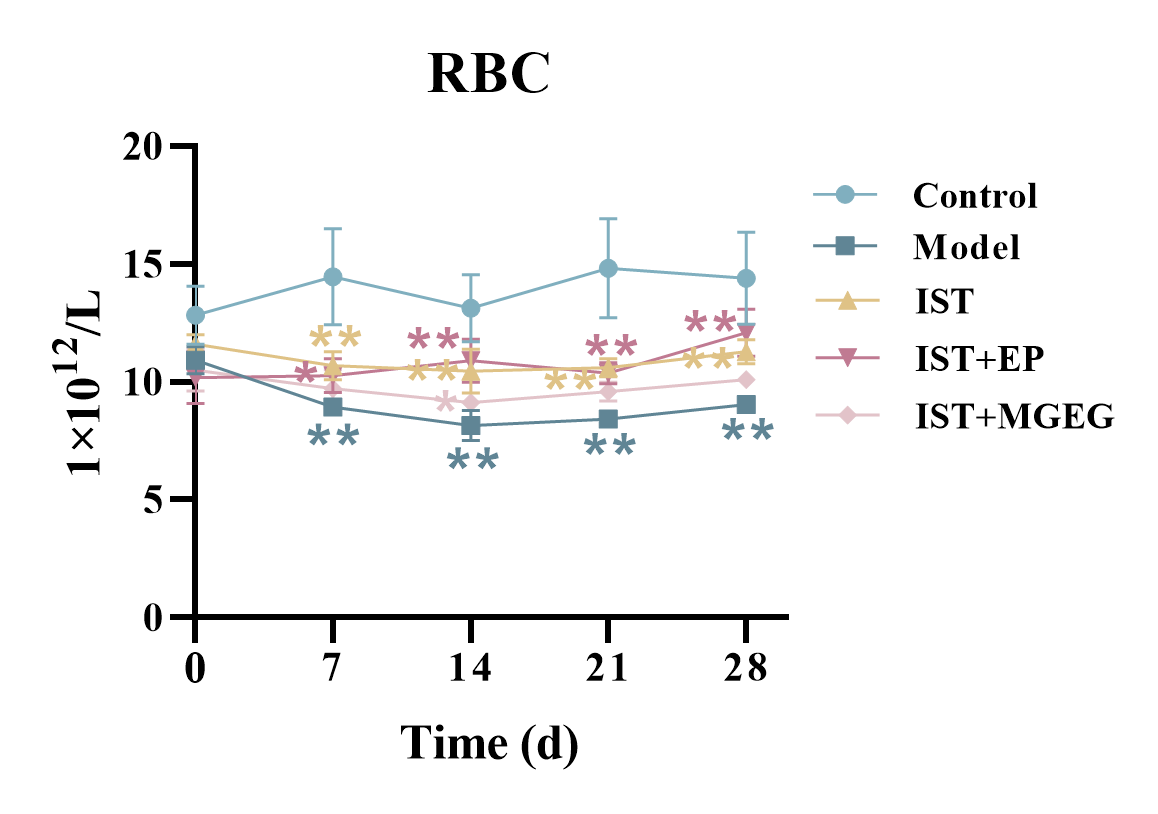

Supplement: Supplementary file 1 — Additional file 1. [file 13020_2025_1266_MOESM1_ESM.zip › Figure 2/RBC.tif]

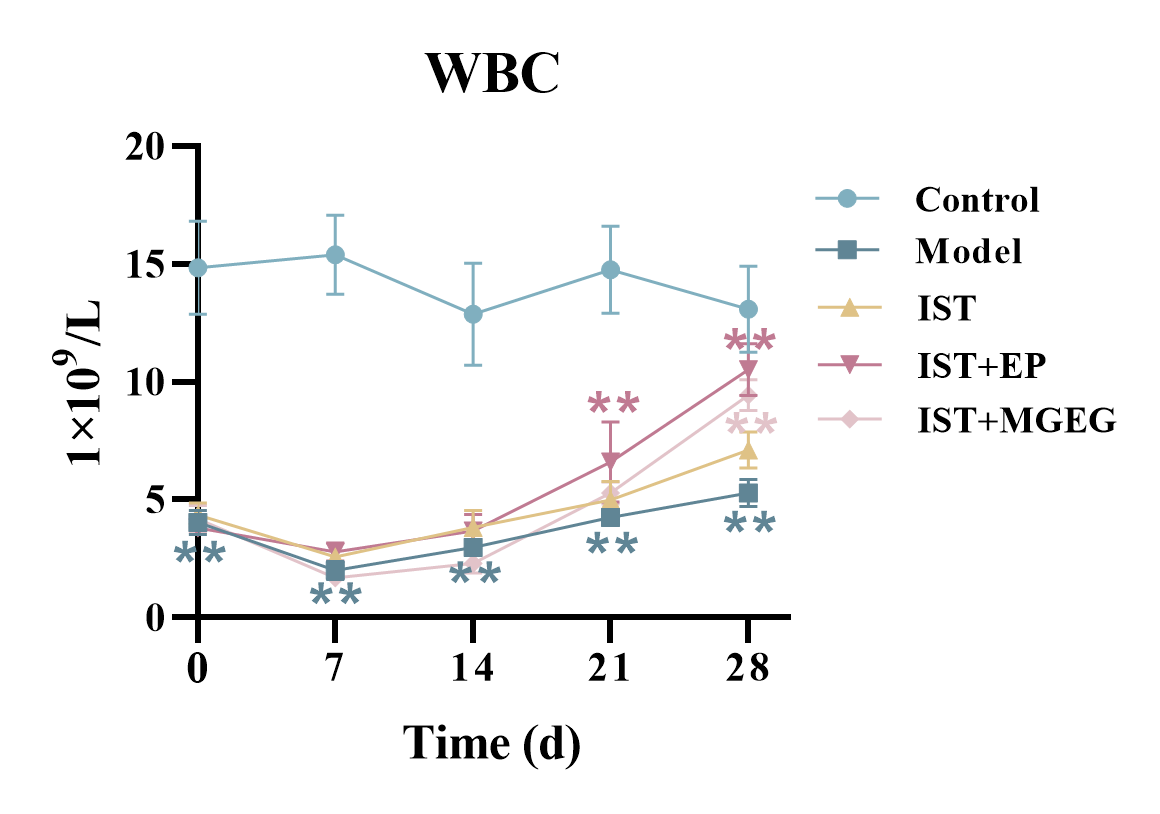

Supplement: Supplementary file 1 — Additional file 1. [file 13020_2025_1266_MOESM1_ESM.zip › Figure 2/WBC.tif]

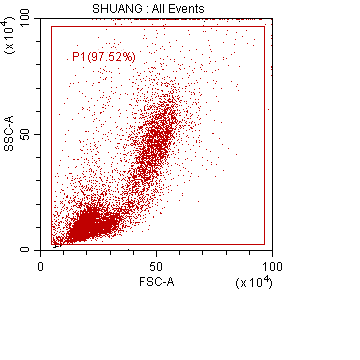

Supplement: Supplementary file 1 — Additional file 1. [file 13020_2025_1266_MOESM1_ESM.zip › Figure 3/BMCs-1.tif]

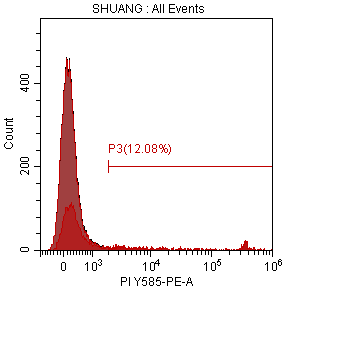

Supplement: Supplementary file 1 — Additional file 1. [file 13020_2025_1266_MOESM1_ESM.zip › Figure 3/BMCs-2.tif]

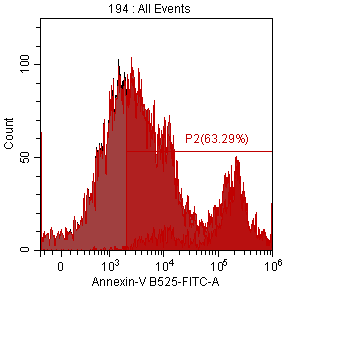

Supplement: Supplementary file 1 — Additional file 1. [file 13020_2025_1266_MOESM1_ESM.zip › Figure 3/BMCs-3.tif]

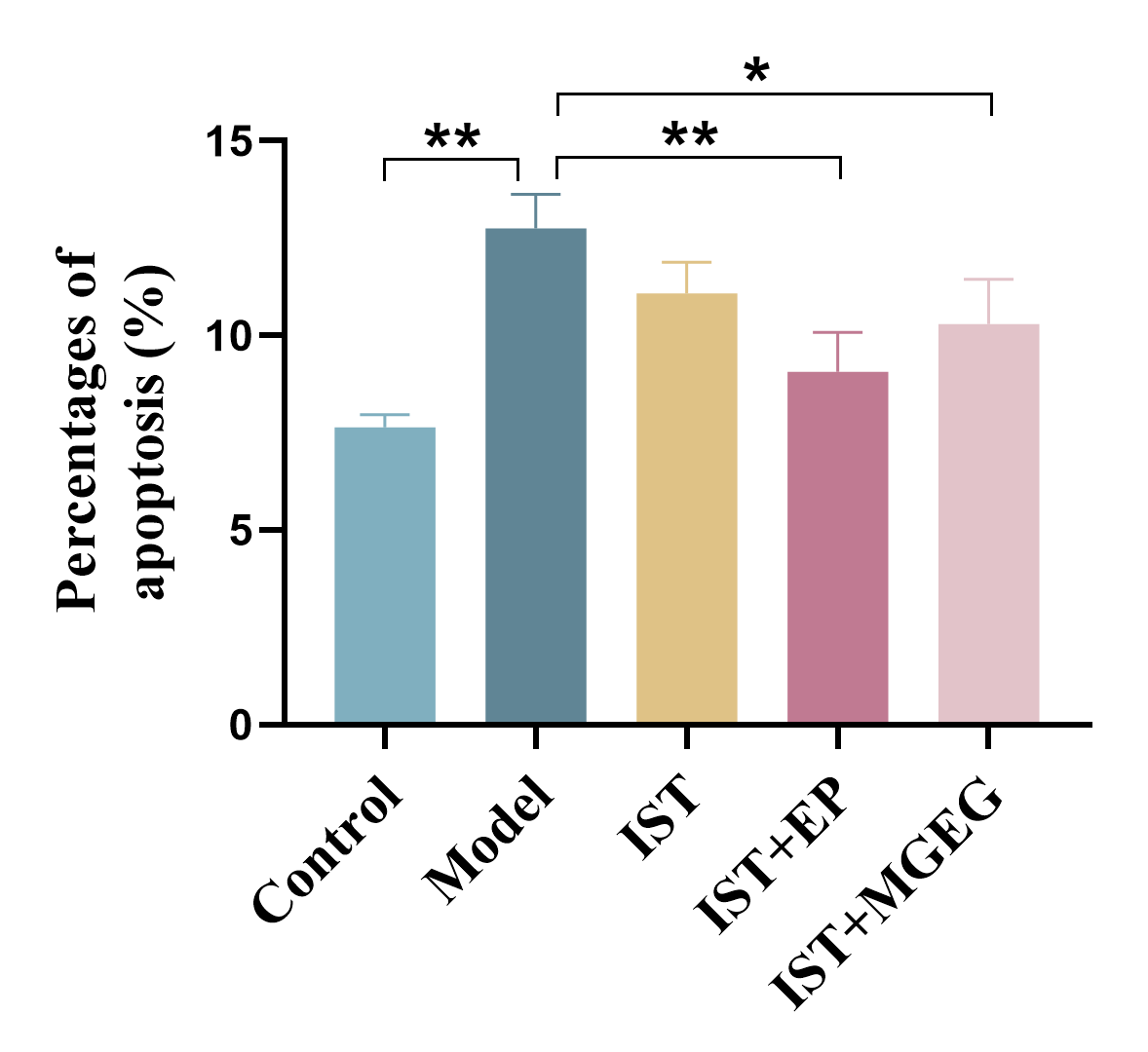

Supplement: Supplementary file 1 — Additional file 1. [file 13020_2025_1266_MOESM1_ESM.zip › Figure 3/BMCs.tif]

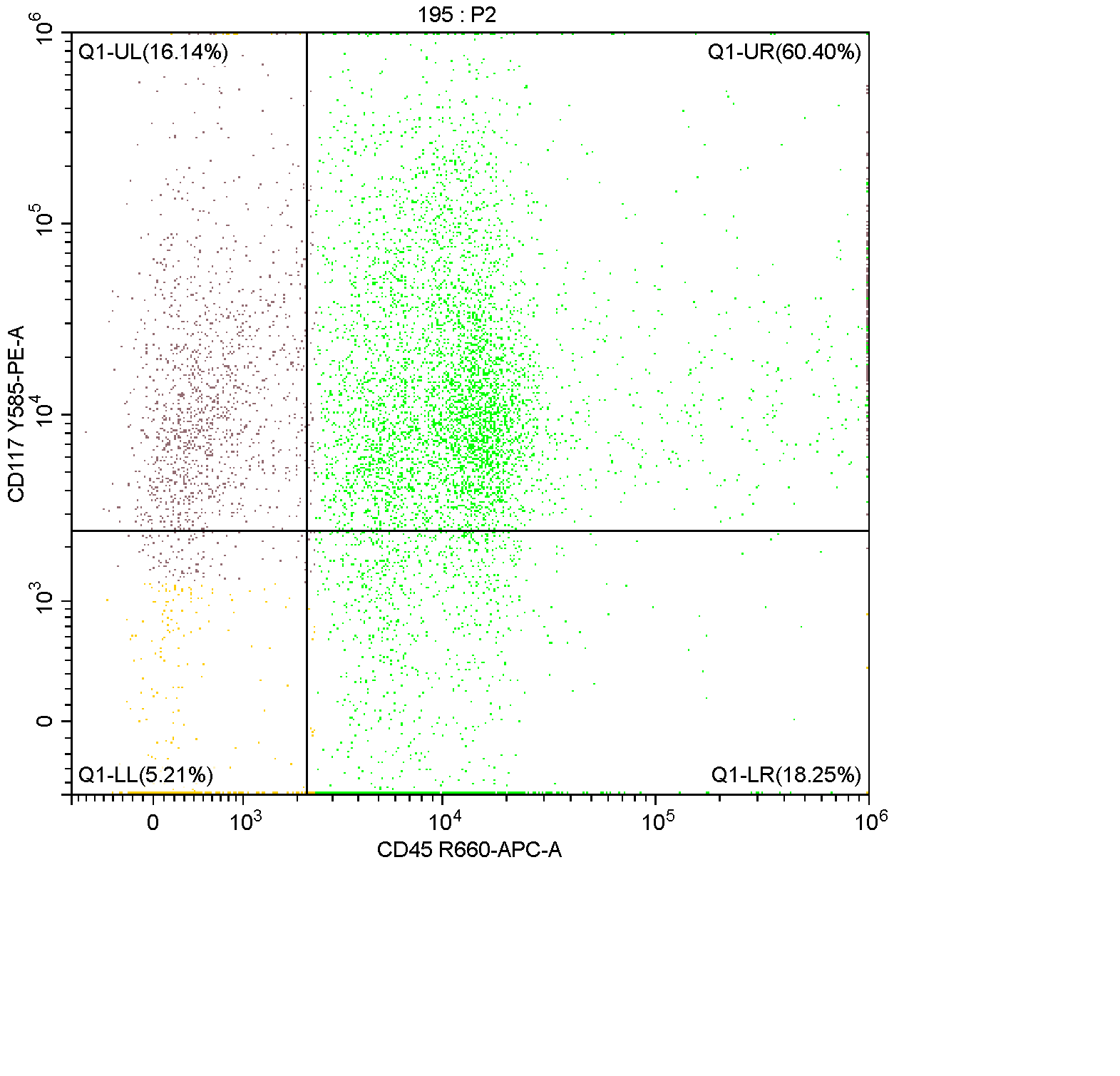

Supplement: Supplementary file 1 — Additional file 1. [file 13020_2025_1266_MOESM1_ESM.zip › Figure 3/Control-195_Plot1.bmp]

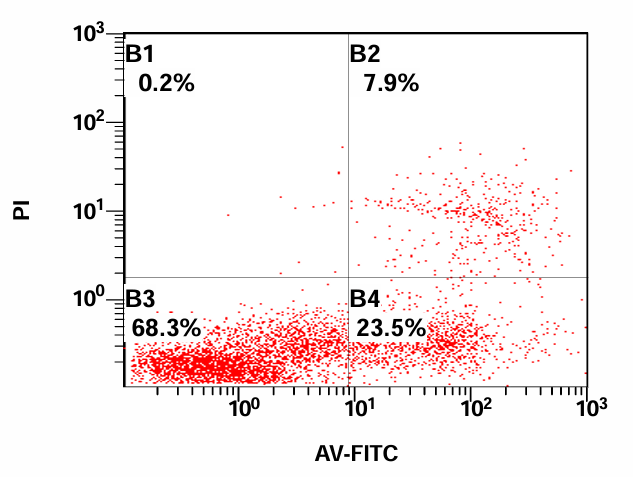

Supplement: Supplementary file 1 — Additional file 1. [file 13020_2025_1266_MOESM1_ESM.zip › Figure 3/Control(7.9).png]

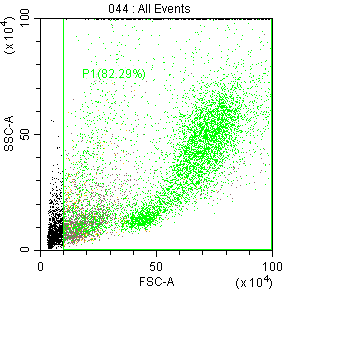

Supplement: Supplementary file 1 — Additional file 1. [file 13020_2025_1266_MOESM1_ESM.zip › Figure 3/HSCs-1.tif]

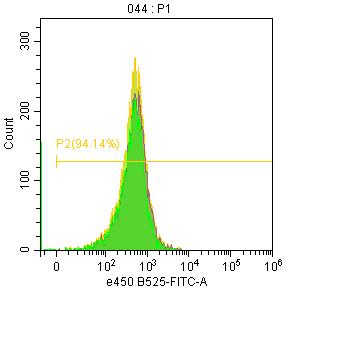

Supplement: Supplementary file 1 — Additional file 1. [file 13020_2025_1266_MOESM1_ESM.zip › Figure 3/HSCs-2.tif]

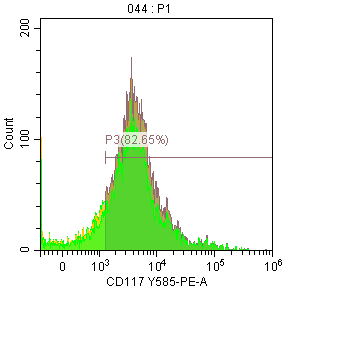

Supplement: Supplementary file 1 — Additional file 1. [file 13020_2025_1266_MOESM1_ESM.zip › Figure 3/HSCs-3.tif]

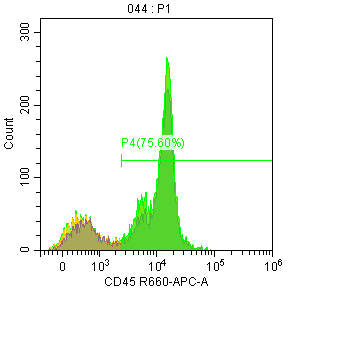

Supplement: Supplementary file 1 — Additional file 1. [file 13020_2025_1266_MOESM1_ESM.zip › Figure 3/HSCs-4.tif]

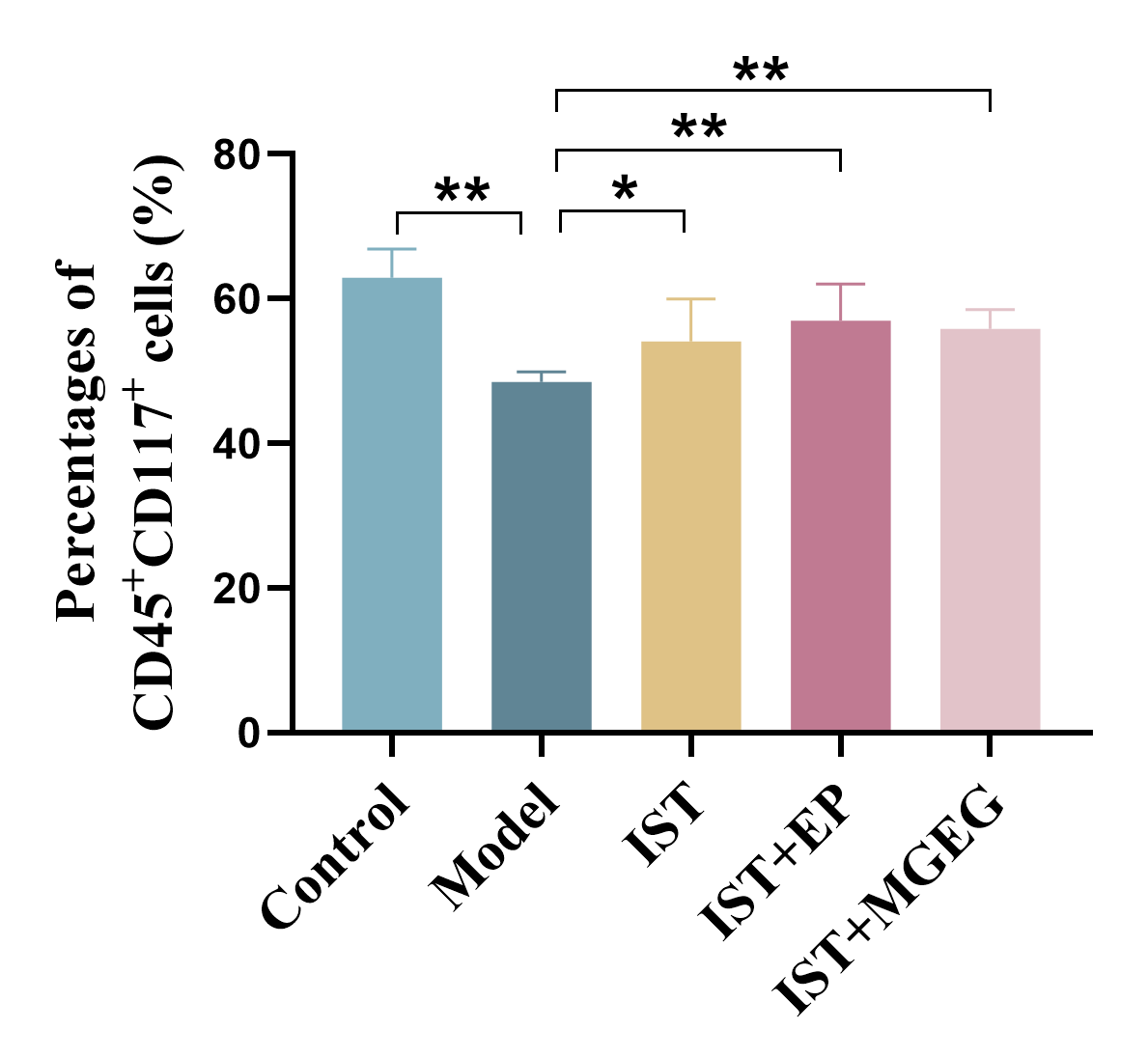

Supplement: Supplementary file 1 — Additional file 1. [file 13020_2025_1266_MOESM1_ESM.zip › Figure 3/HSCs.tif]

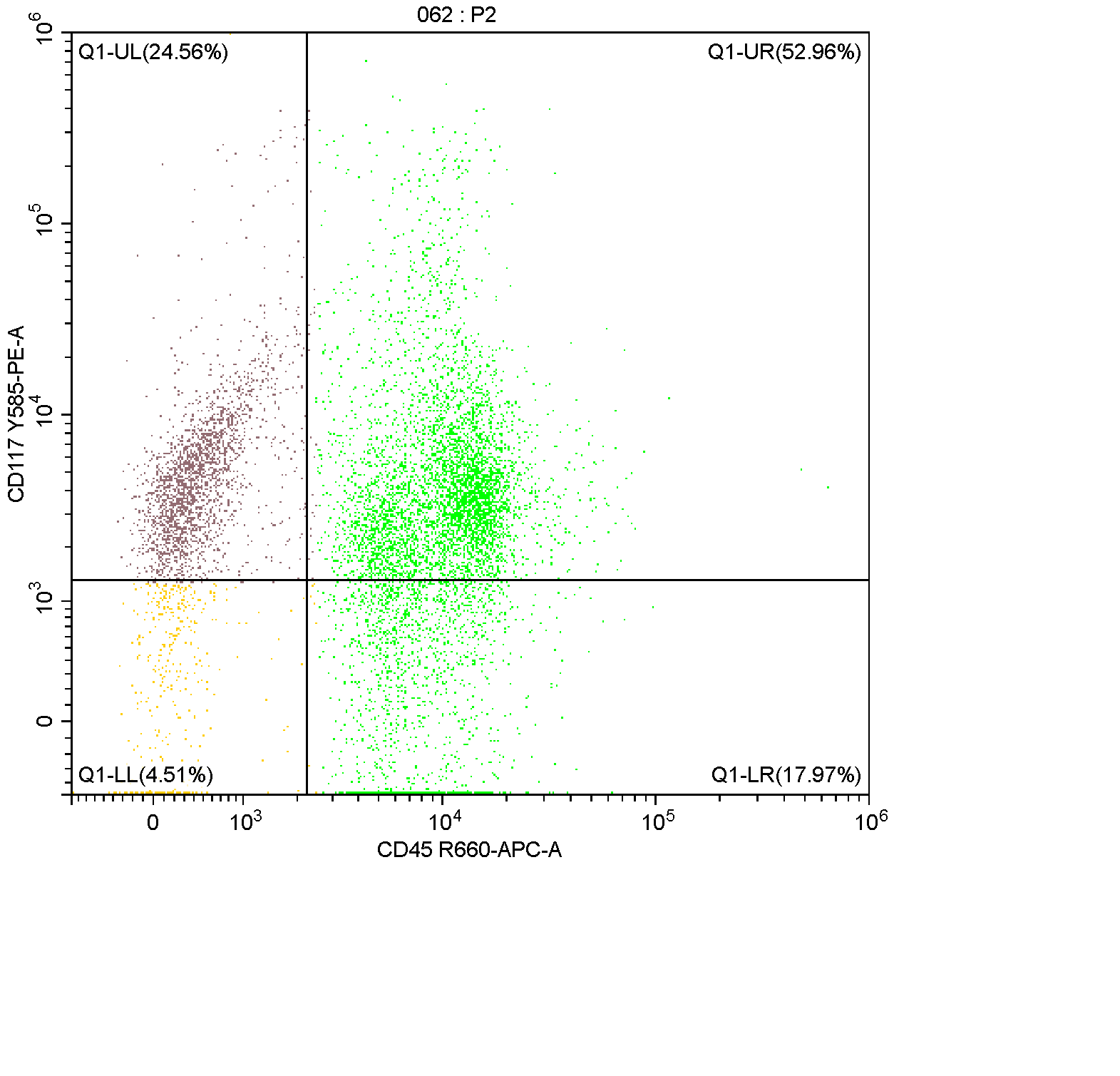

Supplement: Supplementary file 1 — Additional file 1. [file 13020_2025_1266_MOESM1_ESM.zip › Figure 3/IST 062_Plot1.bmp]

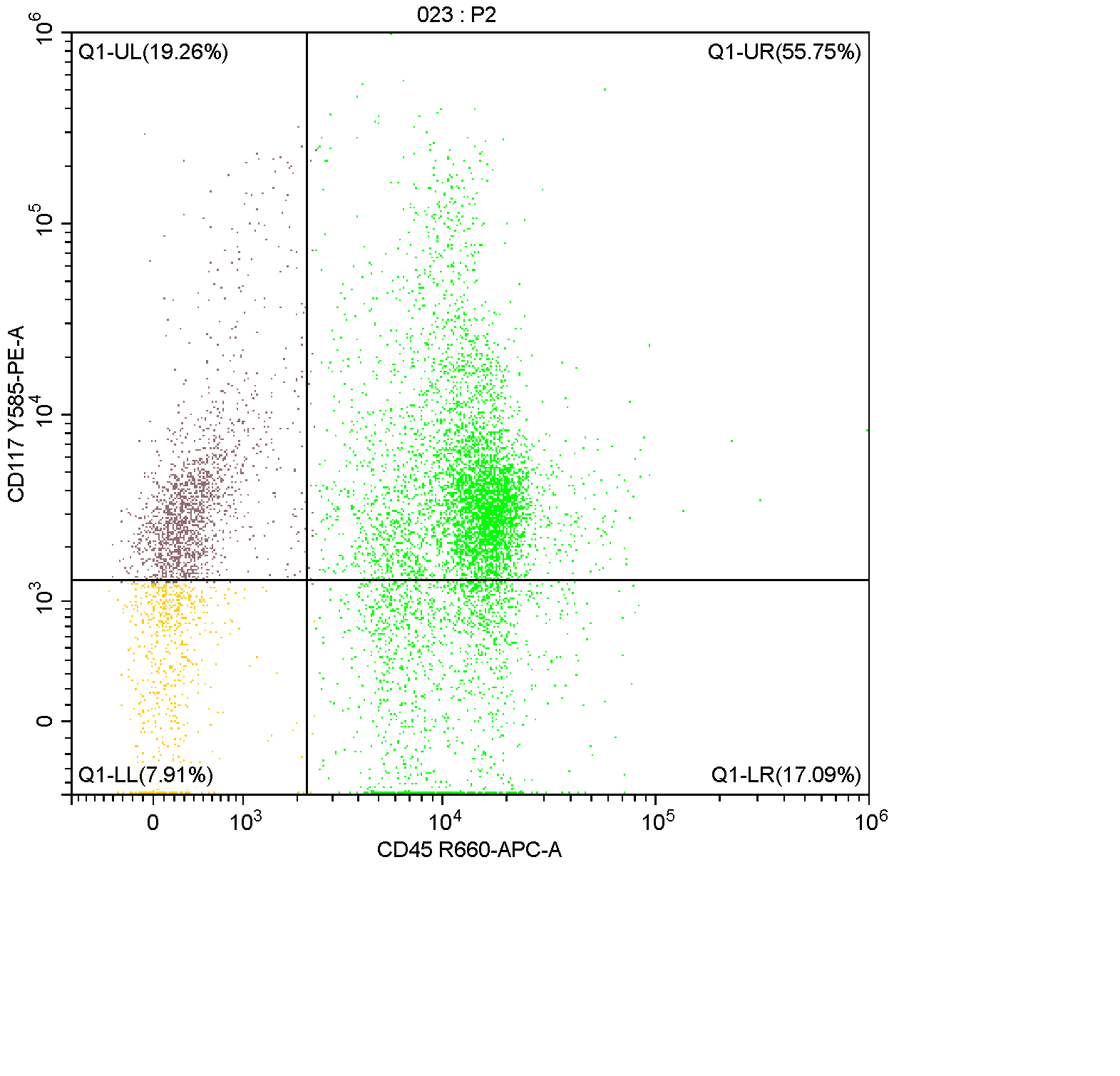

Supplement: Supplementary file 1 — Additional file 1. [file 13020_2025_1266_MOESM1_ESM.zip › Figure 3/IST+EP-023_Plot1.bmp]

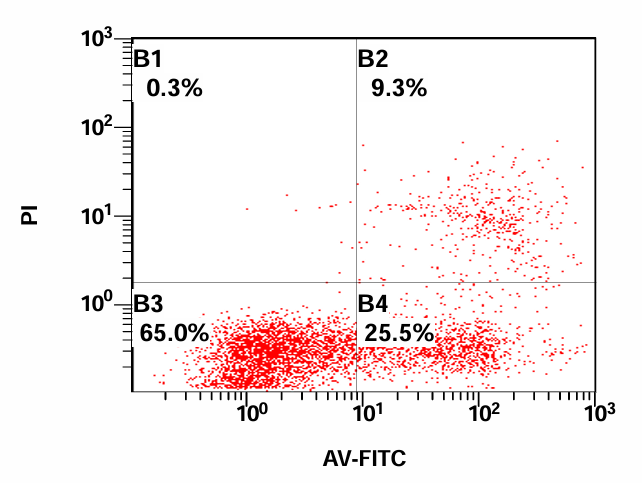

Supplement: Supplementary file 1 — Additional file 1. [file 13020_2025_1266_MOESM1_ESM.zip › Figure 3/IST+EP(9.3).png]

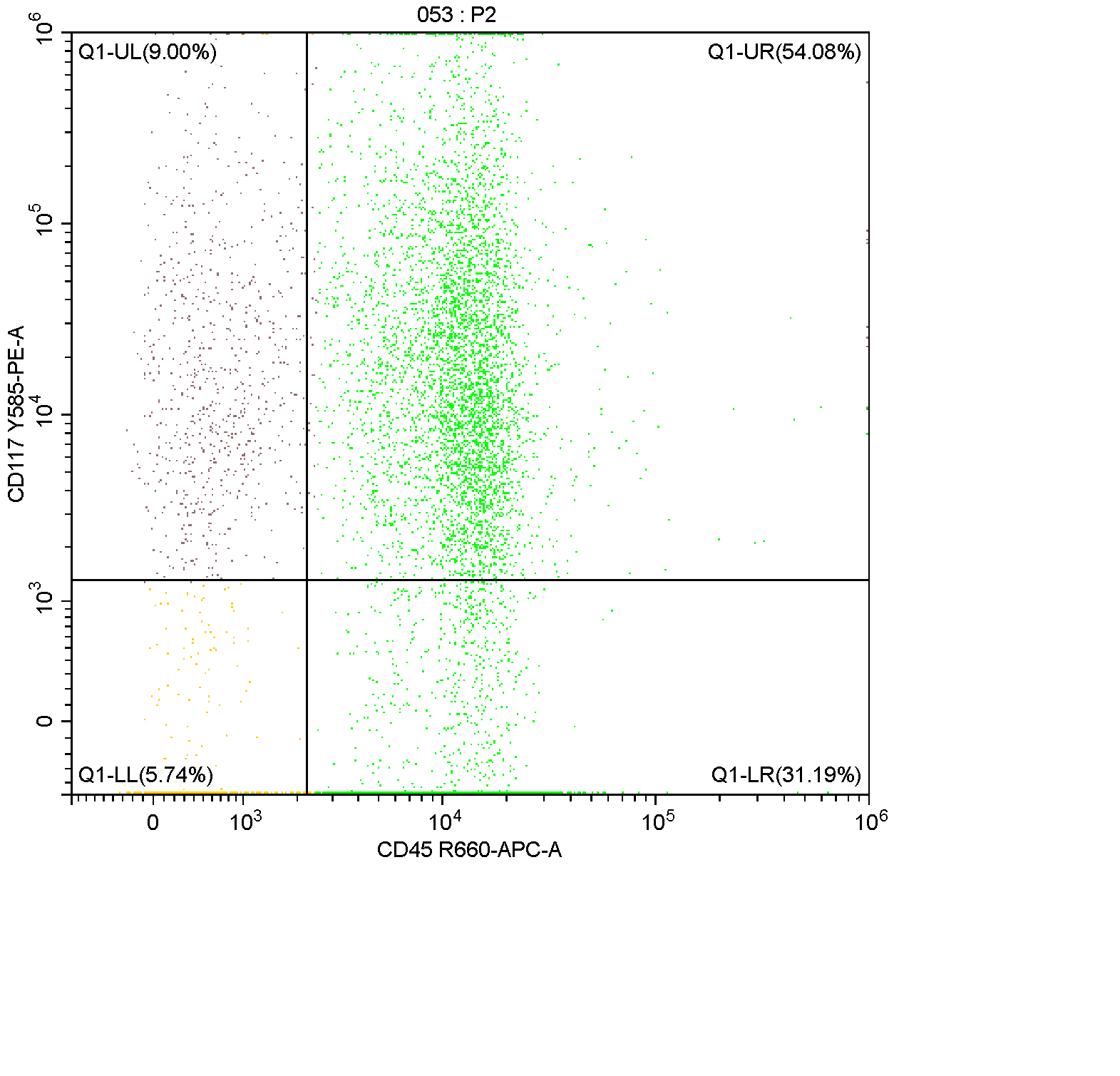

Supplement: Supplementary file 1 — Additional file 1. [file 13020_2025_1266_MOESM1_ESM.zip › Figure 3/IST+MGEG 053_Plot1.bmp]

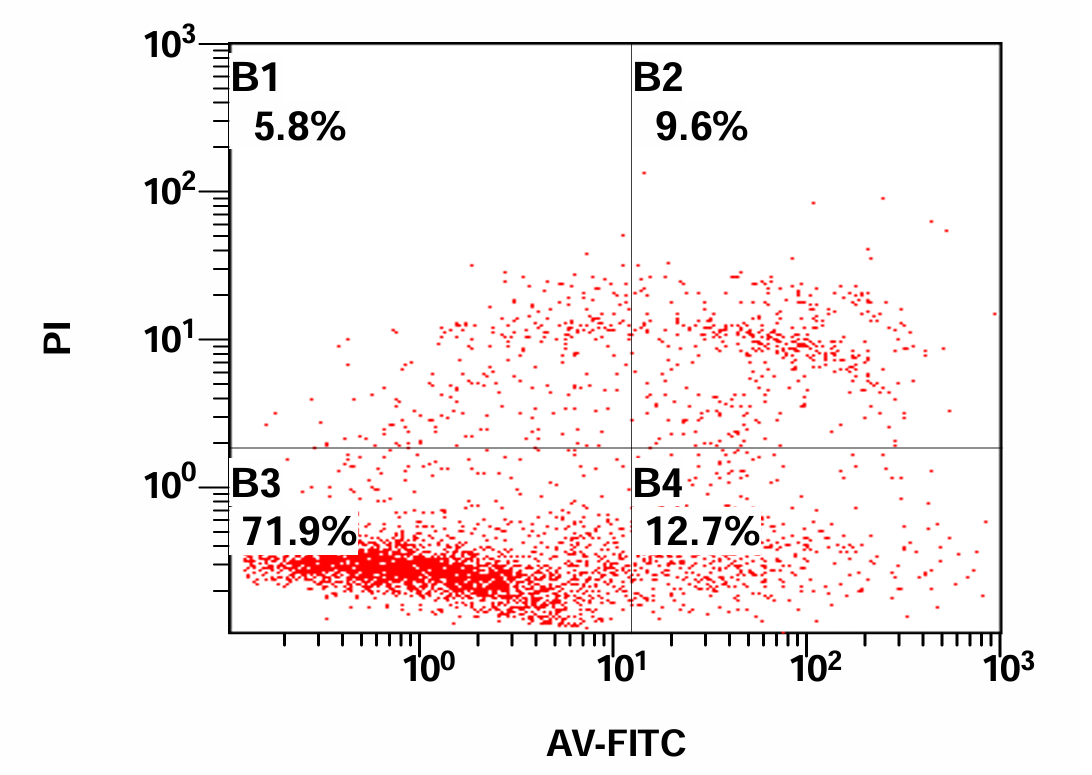

Supplement: Supplementary file 1 — Additional file 1. [file 13020_2025_1266_MOESM1_ESM.zip › Figure 3/IST+MGEG(9.6).png]

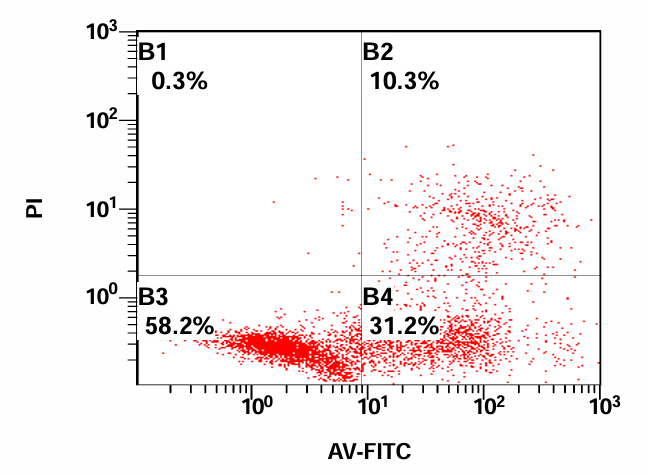

Supplement: Supplementary file 1 — Additional file 1. [file 13020_2025_1266_MOESM1_ESM.zip › Figure 3/IST(10.3).png]

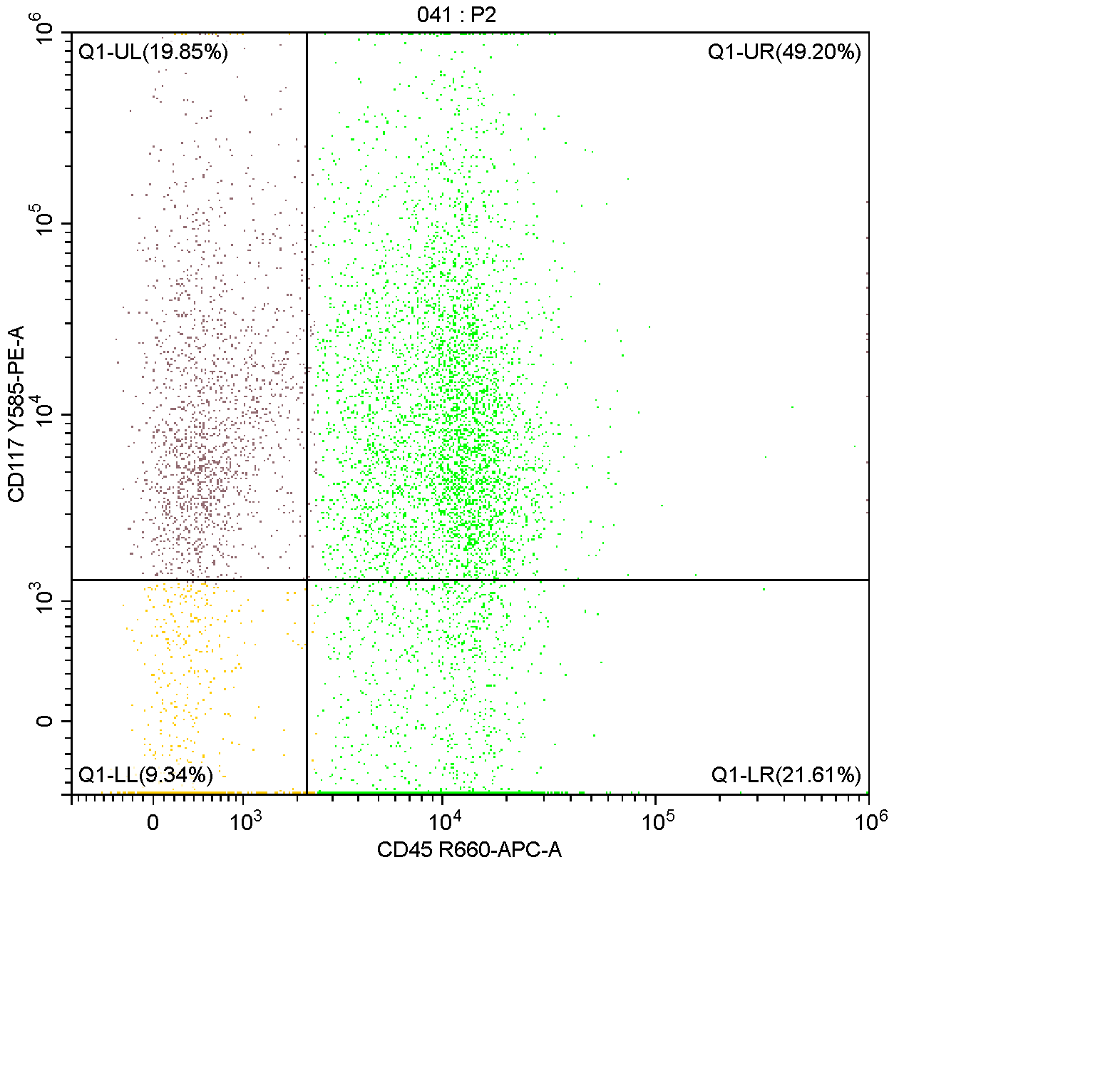

Supplement: Supplementary file 1 — Additional file 1. [file 13020_2025_1266_MOESM1_ESM.zip › Figure 3/Model-041_Plot1.bmp]

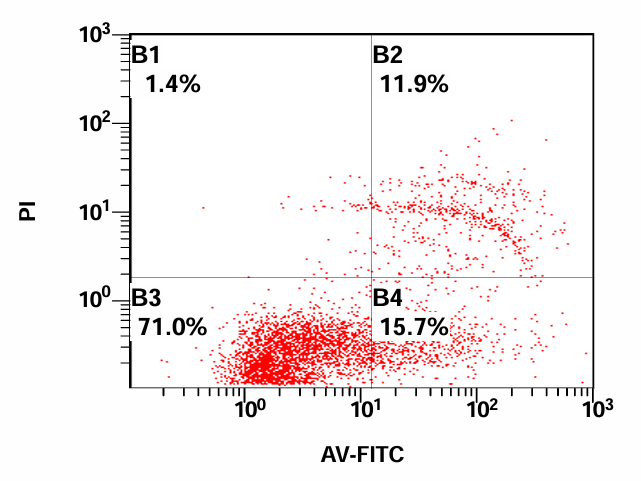

Supplement: Supplementary file 1 — Additional file 1. [file 13020_2025_1266_MOESM1_ESM.zip › Figure 3/Model(11.9).png]

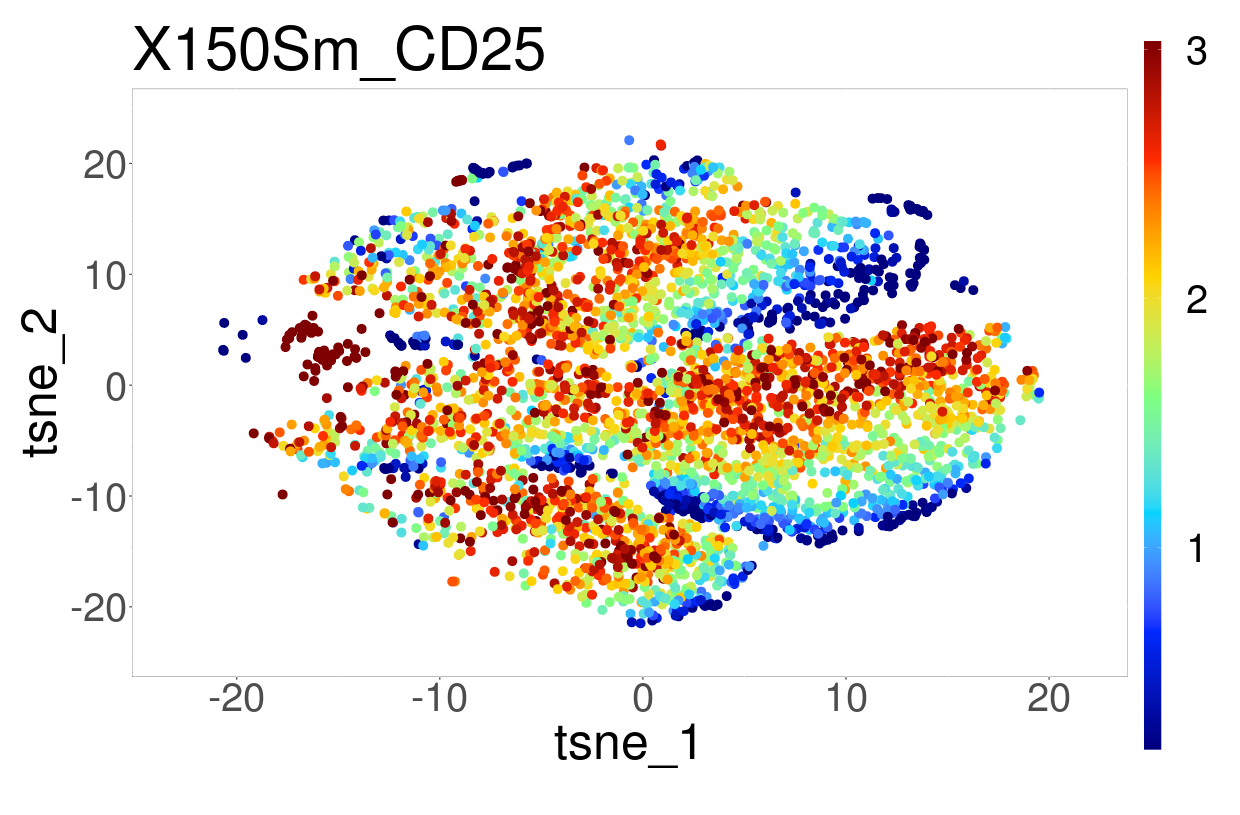

Supplement: Supplementary file 1 — Additional file 1. [file 13020_2025_1266_MOESM1_ESM.zip › Figure 4/CD25.png]

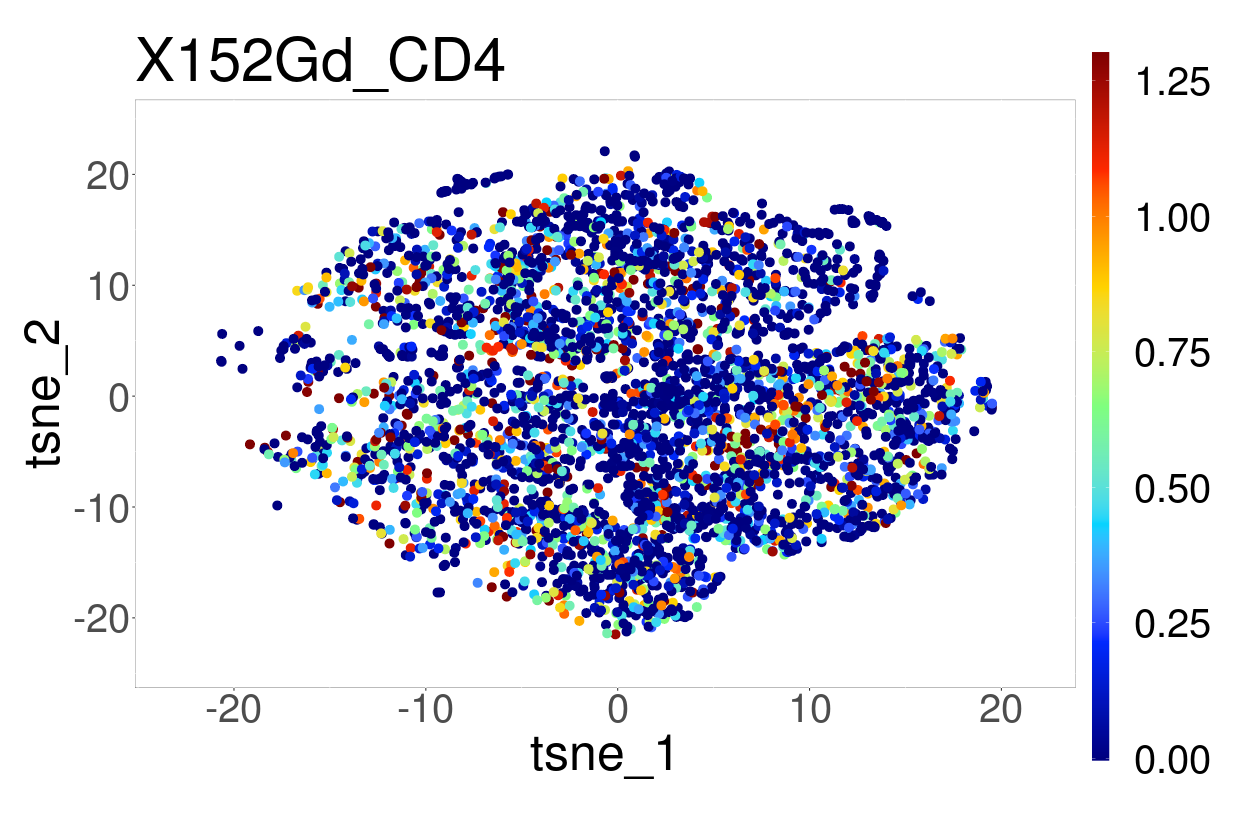

Supplement: Supplementary file 1 — Additional file 1. [file 13020_2025_1266_MOESM1_ESM.zip › Figure 4/CD4.png]

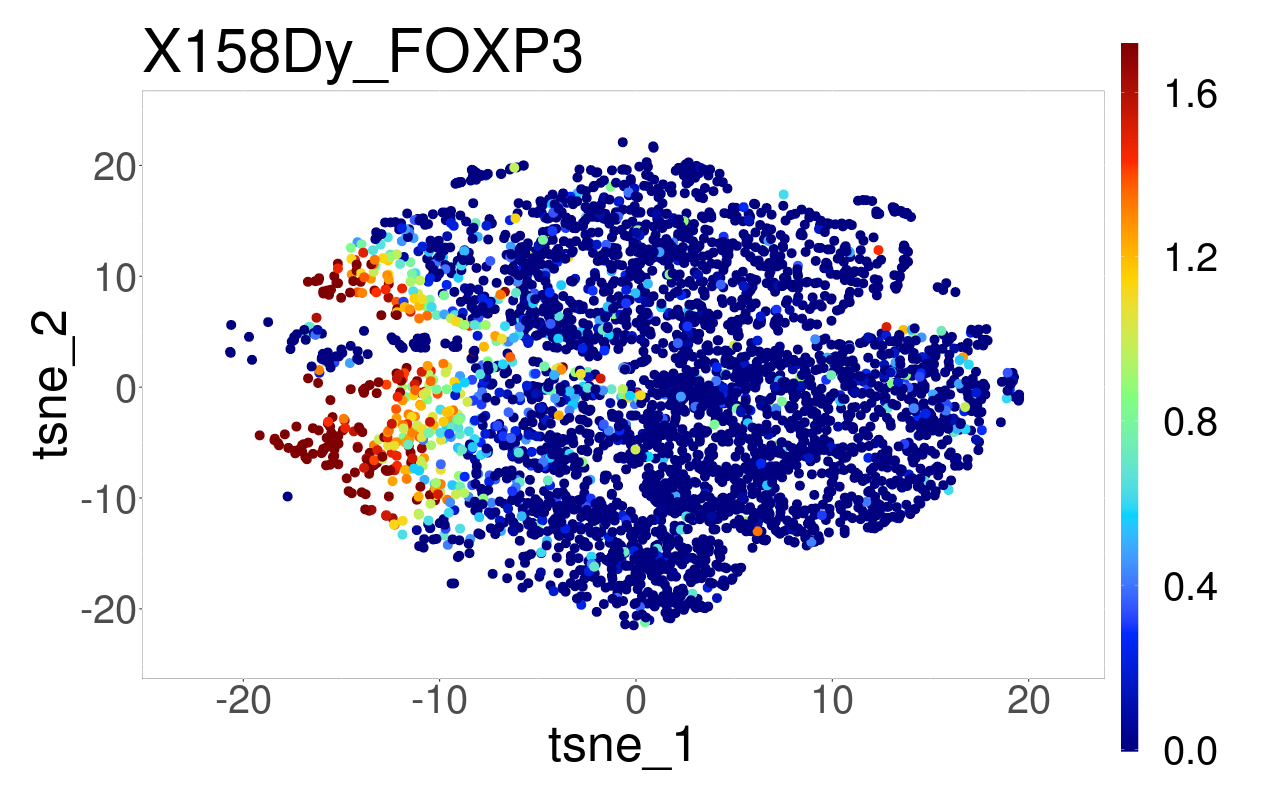

Supplement: Supplementary file 1 — Additional file 1. [file 13020_2025_1266_MOESM1_ESM.zip › Figure 4/FOXP3.png]

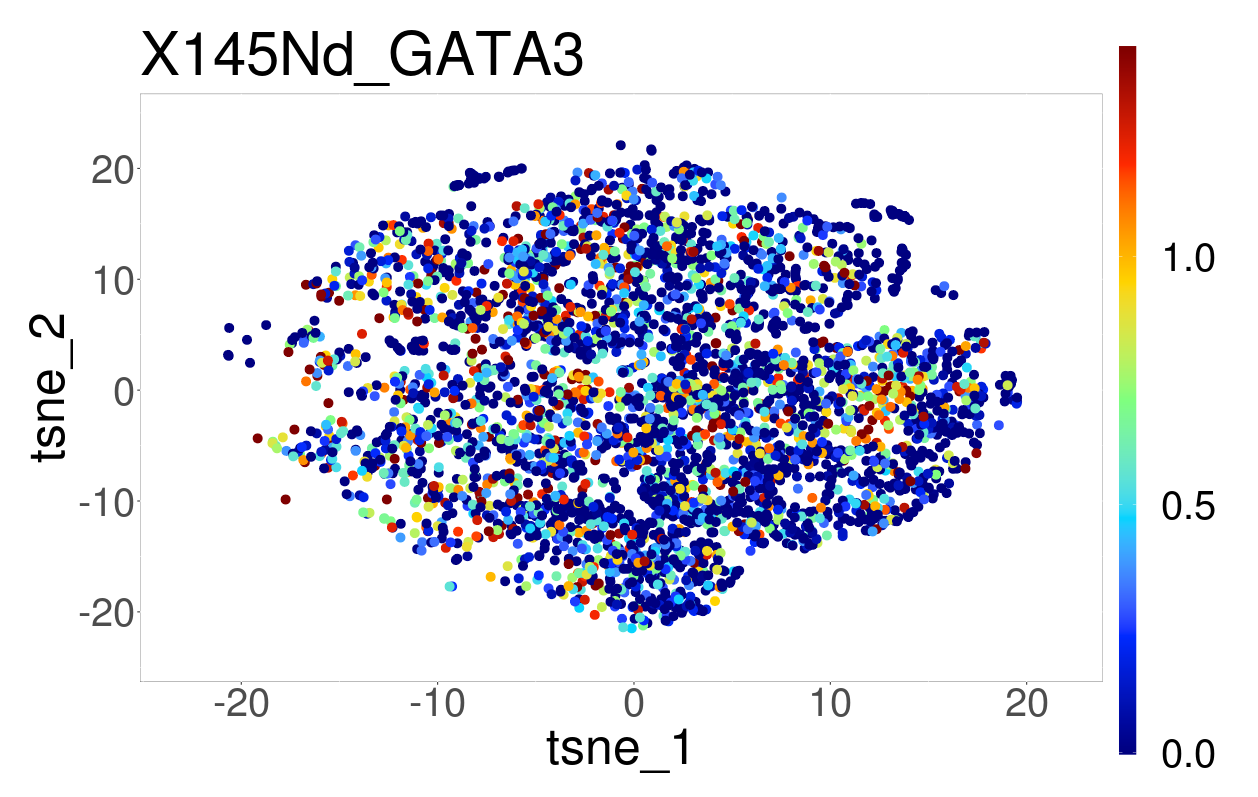

Supplement: Supplementary file 1 — Additional file 1. [file 13020_2025_1266_MOESM1_ESM.zip › Figure 4/GATA3.png]

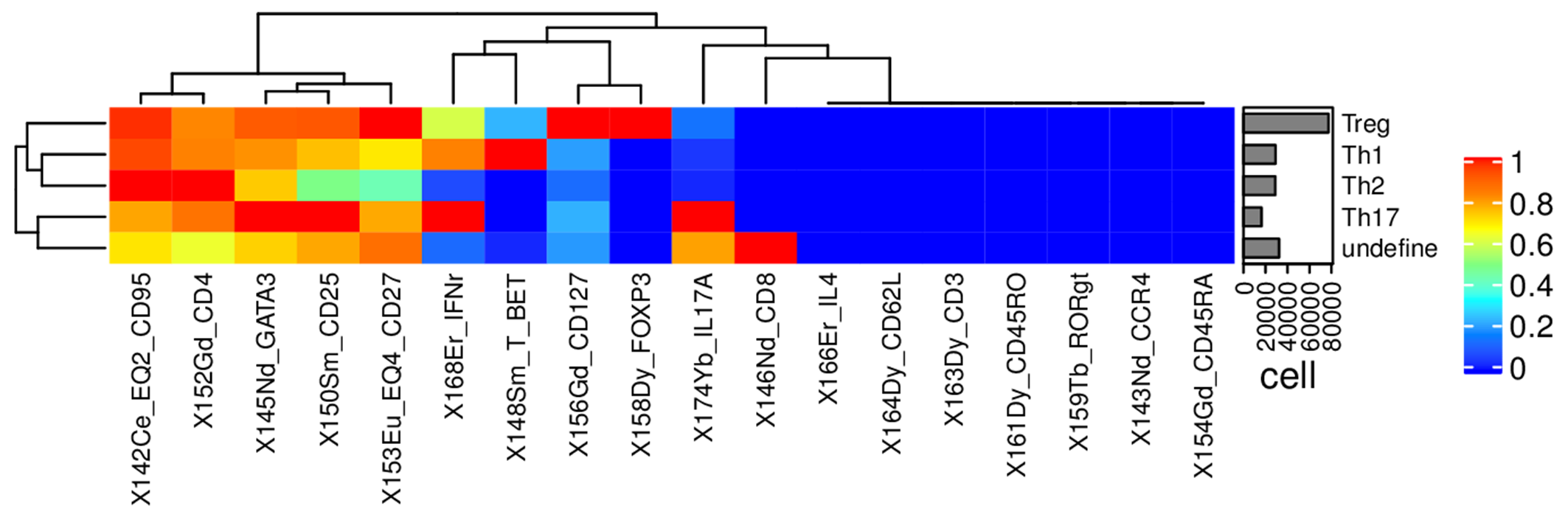

Supplement: Supplementary file 1 — Additional file 1. [file 13020_2025_1266_MOESM1_ESM.zip › Figure 4/heatmap.tif]

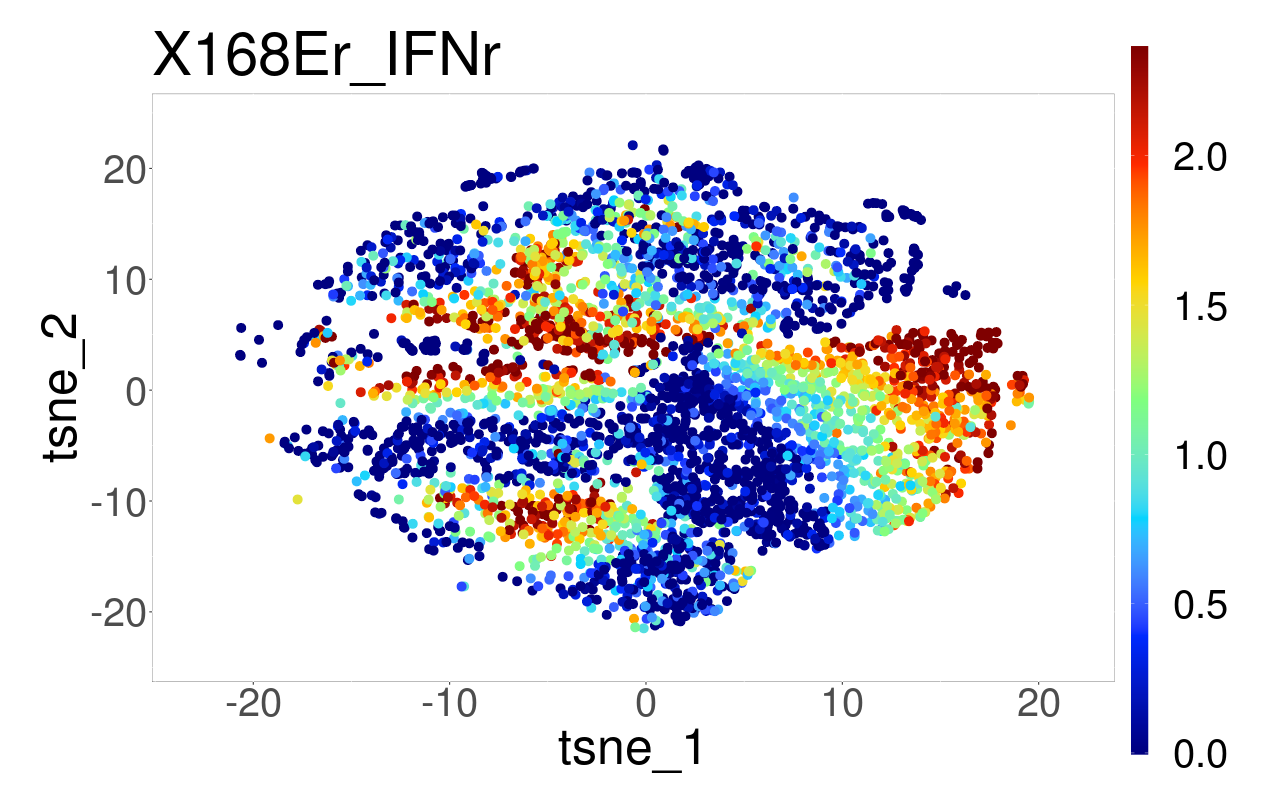

Supplement: Supplementary file 1 — Additional file 1. [file 13020_2025_1266_MOESM1_ESM.zip › Figure 4/IFN-γ.png]

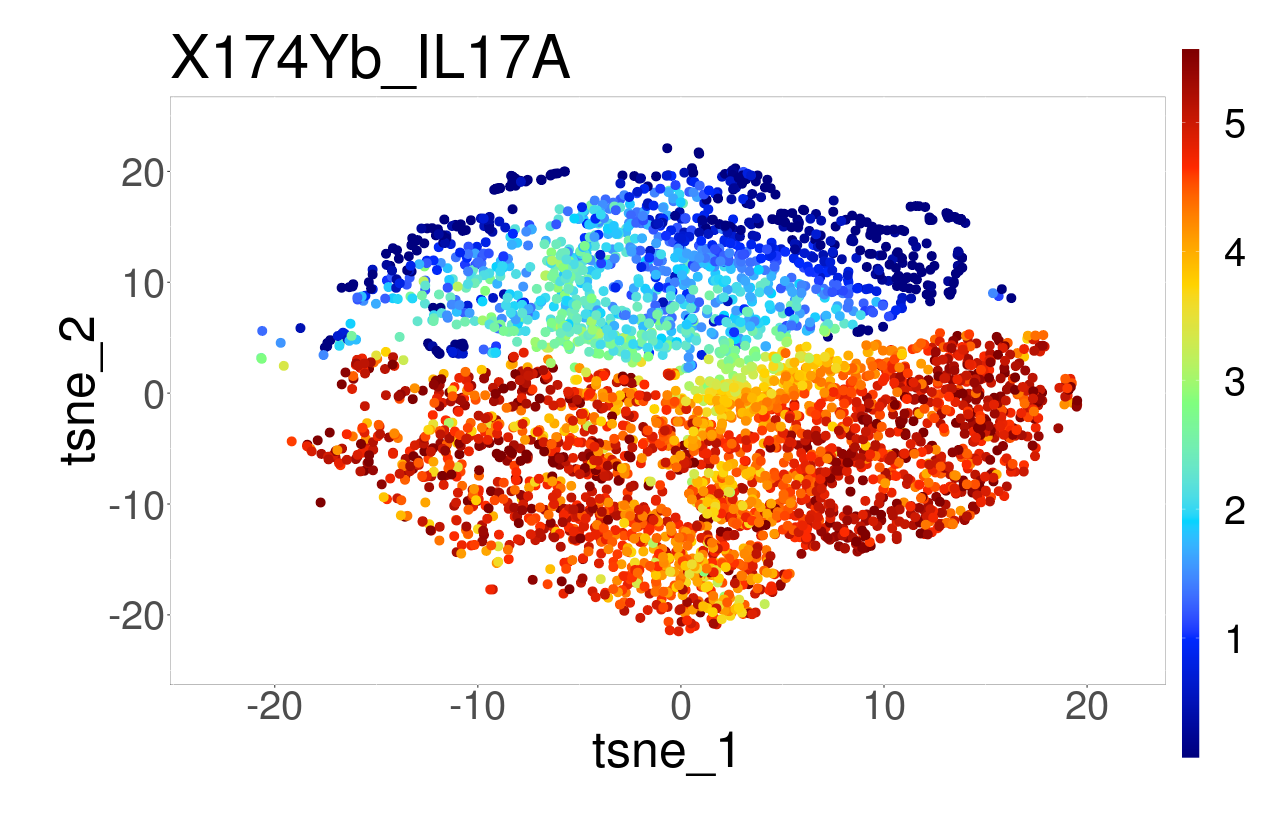

Supplement: Supplementary file 1 — Additional file 1. [file 13020_2025_1266_MOESM1_ESM.zip › Figure 4/IL-17A.png]

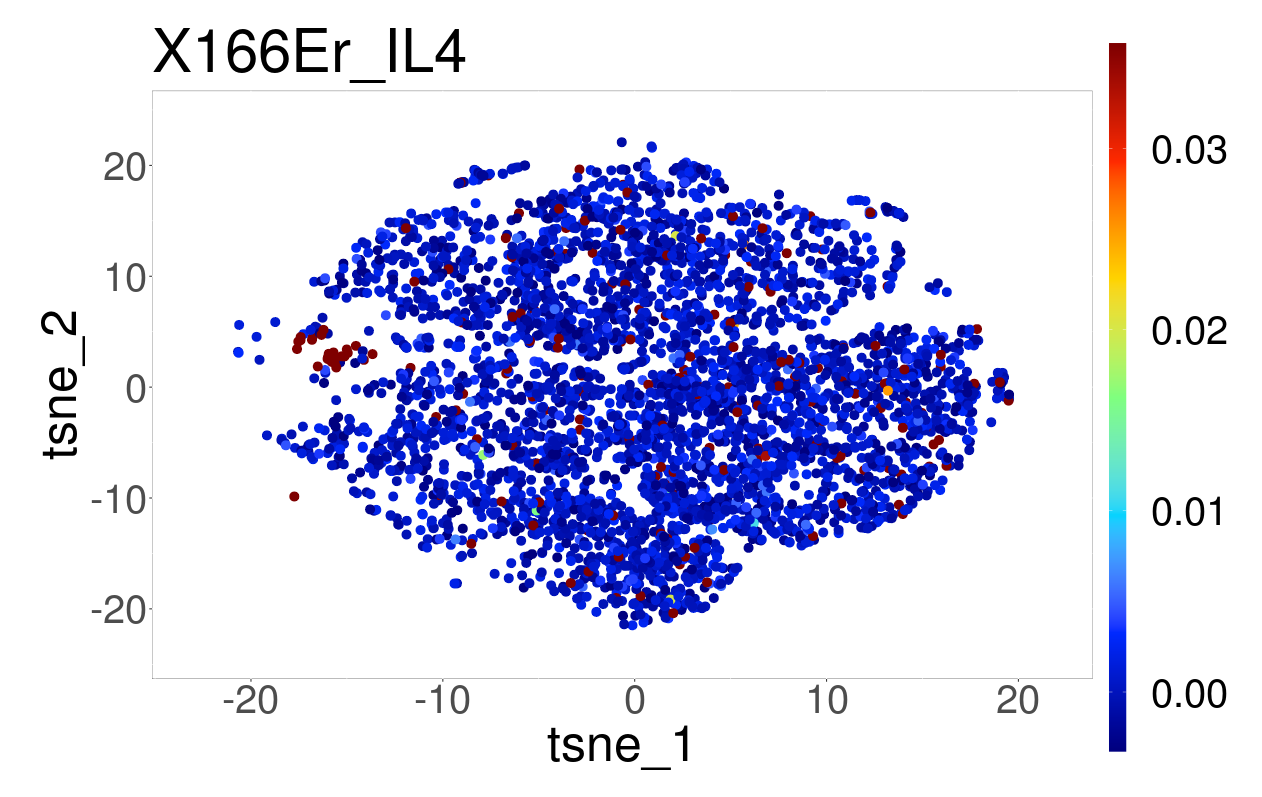

Supplement: Supplementary file 1 — Additional file 1. [file 13020_2025_1266_MOESM1_ESM.zip › Figure 4/IL-4.png]

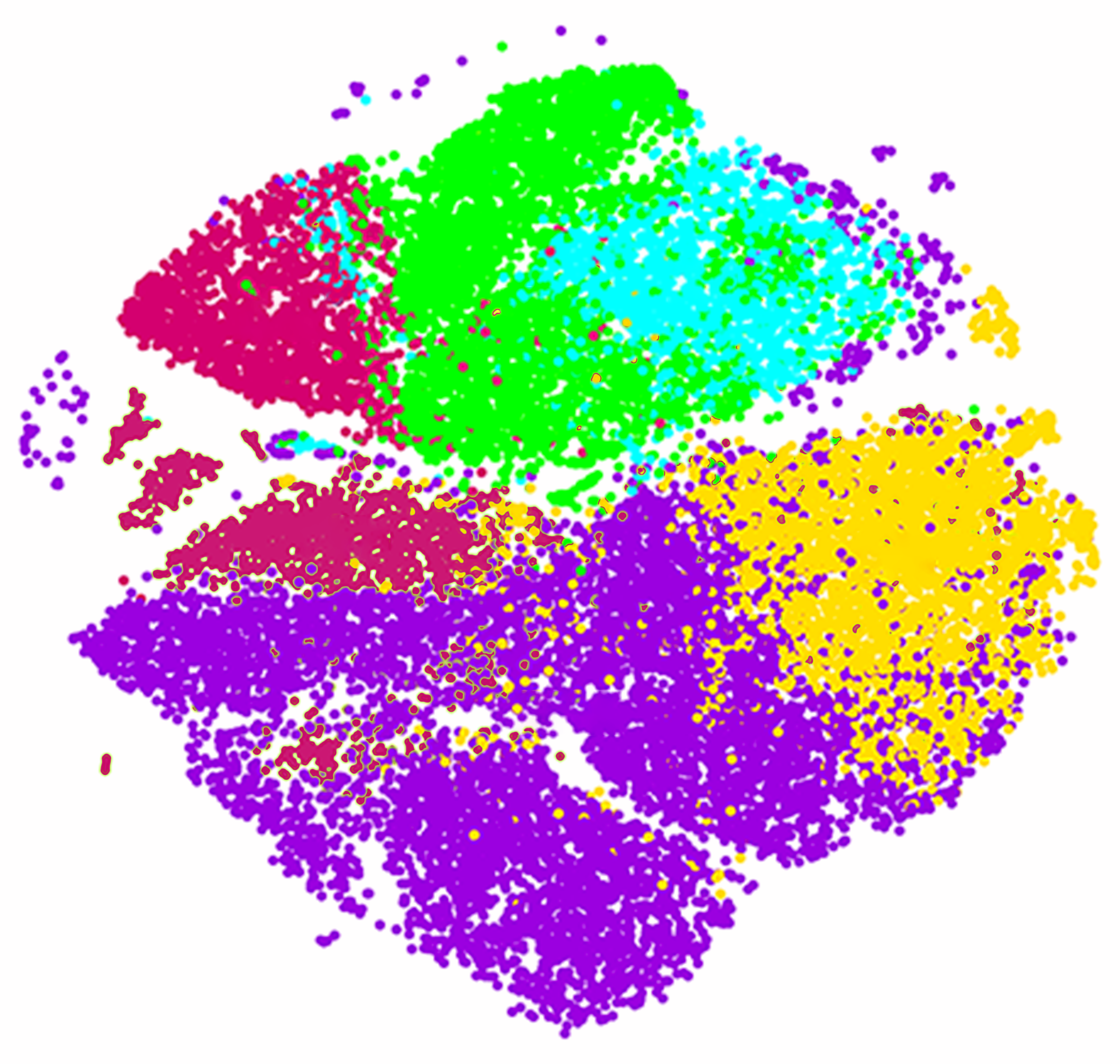

Supplement: Supplementary file 1 — Additional file 1. [file 13020_2025_1266_MOESM1_ESM.zip › Figure 4/IST+EP-A28-300bpi.png]

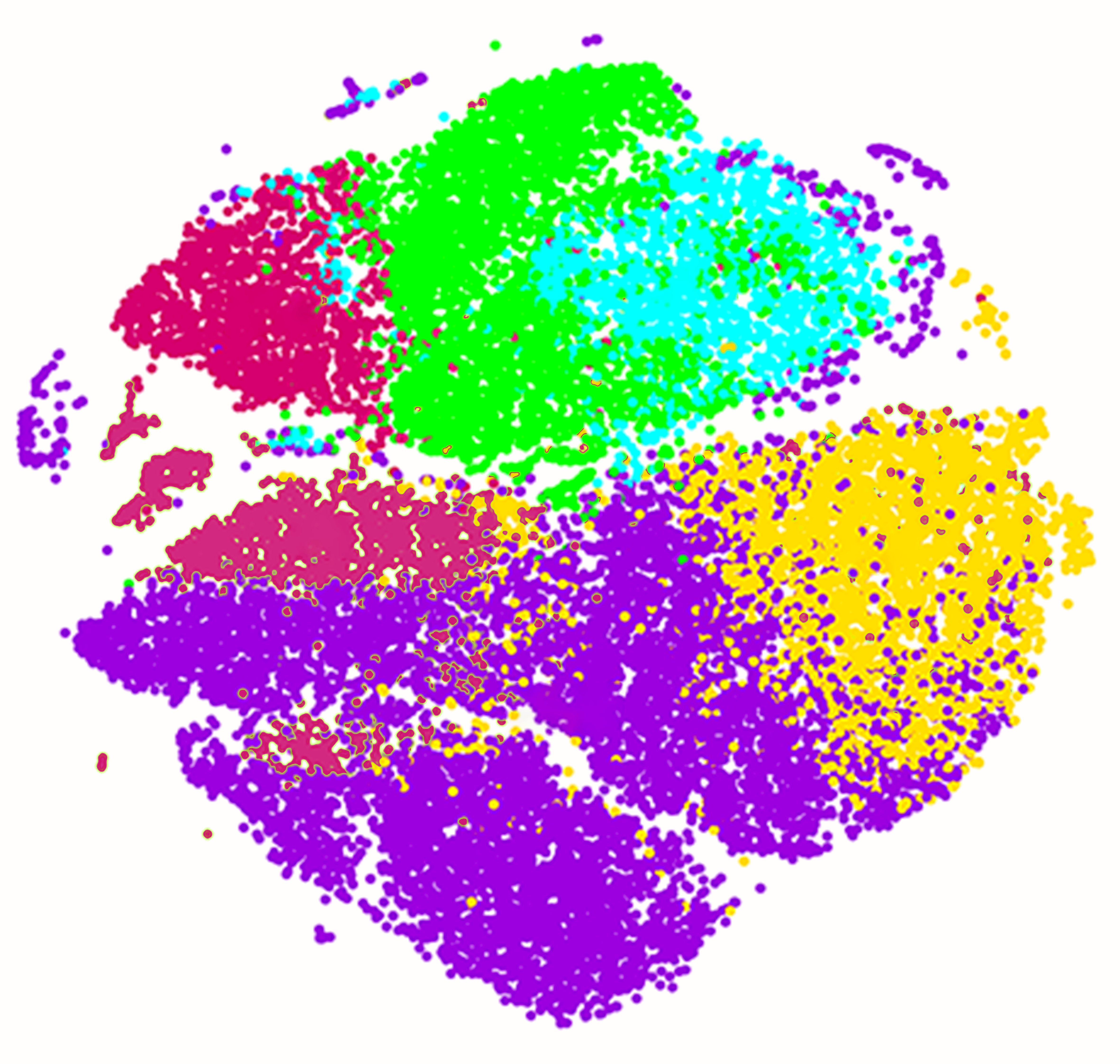

Supplement: Supplementary file 1 — Additional file 1. [file 13020_2025_1266_MOESM1_ESM.zip › Figure 4/IST+MGEG-A51-300bpi.png]

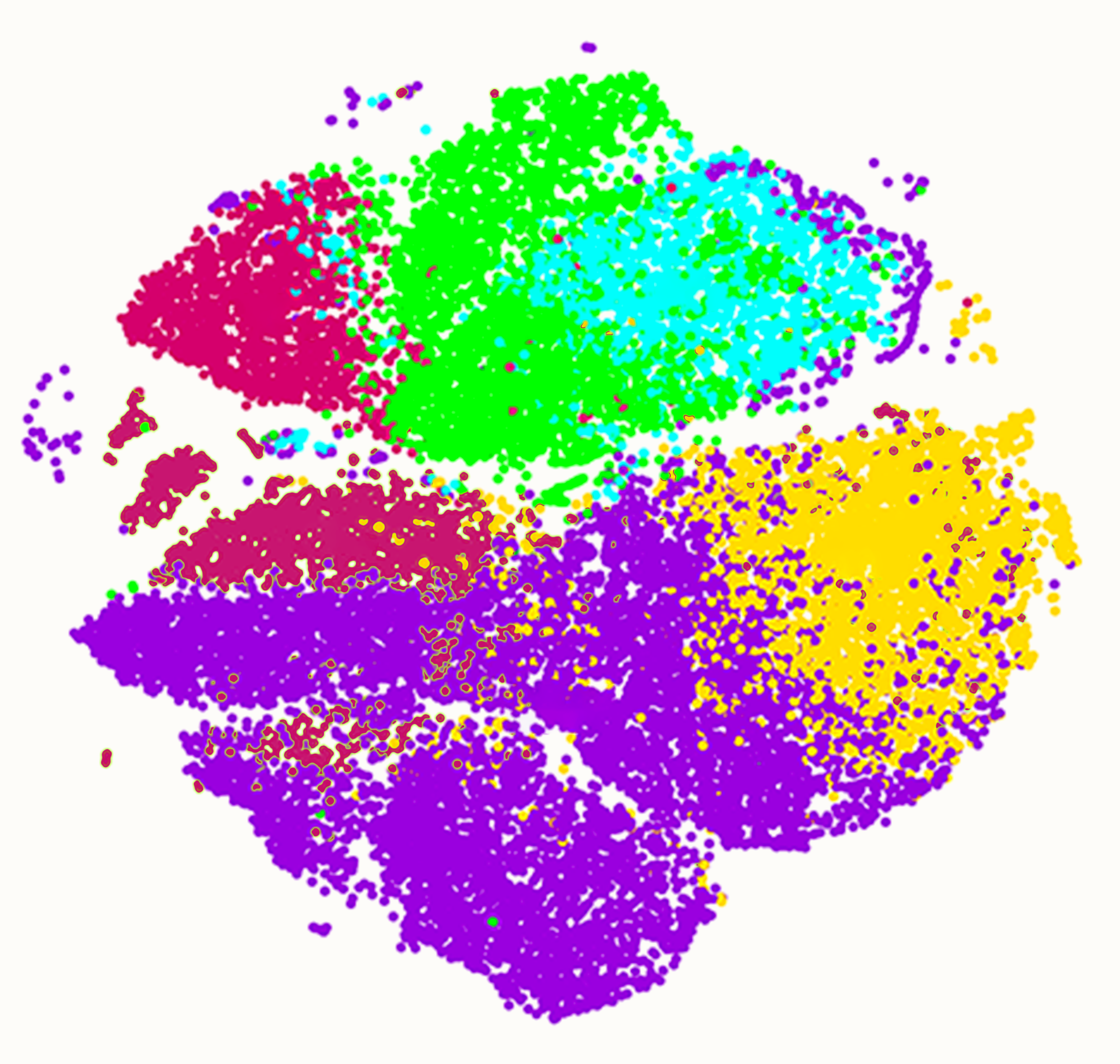

Supplement: Supplementary file 1 — Additional file 1. [file 13020_2025_1266_MOESM1_ESM.zip › Figure 4/IST-A18-300bpi.png]

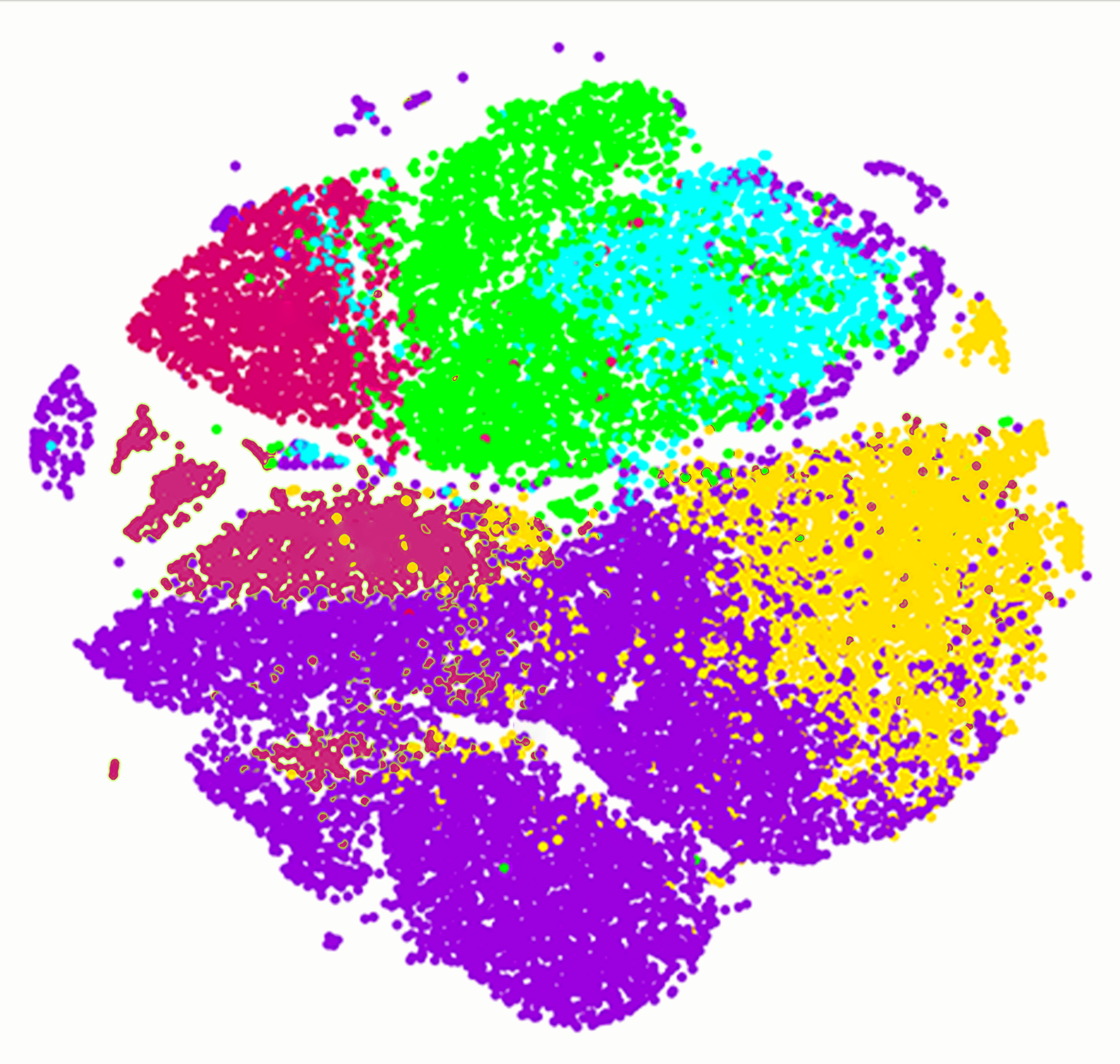

Supplement: Supplementary file 1 — Additional file 1. [file 13020_2025_1266_MOESM1_ESM.zip › Figure 4/M-A66-300bpi.png]

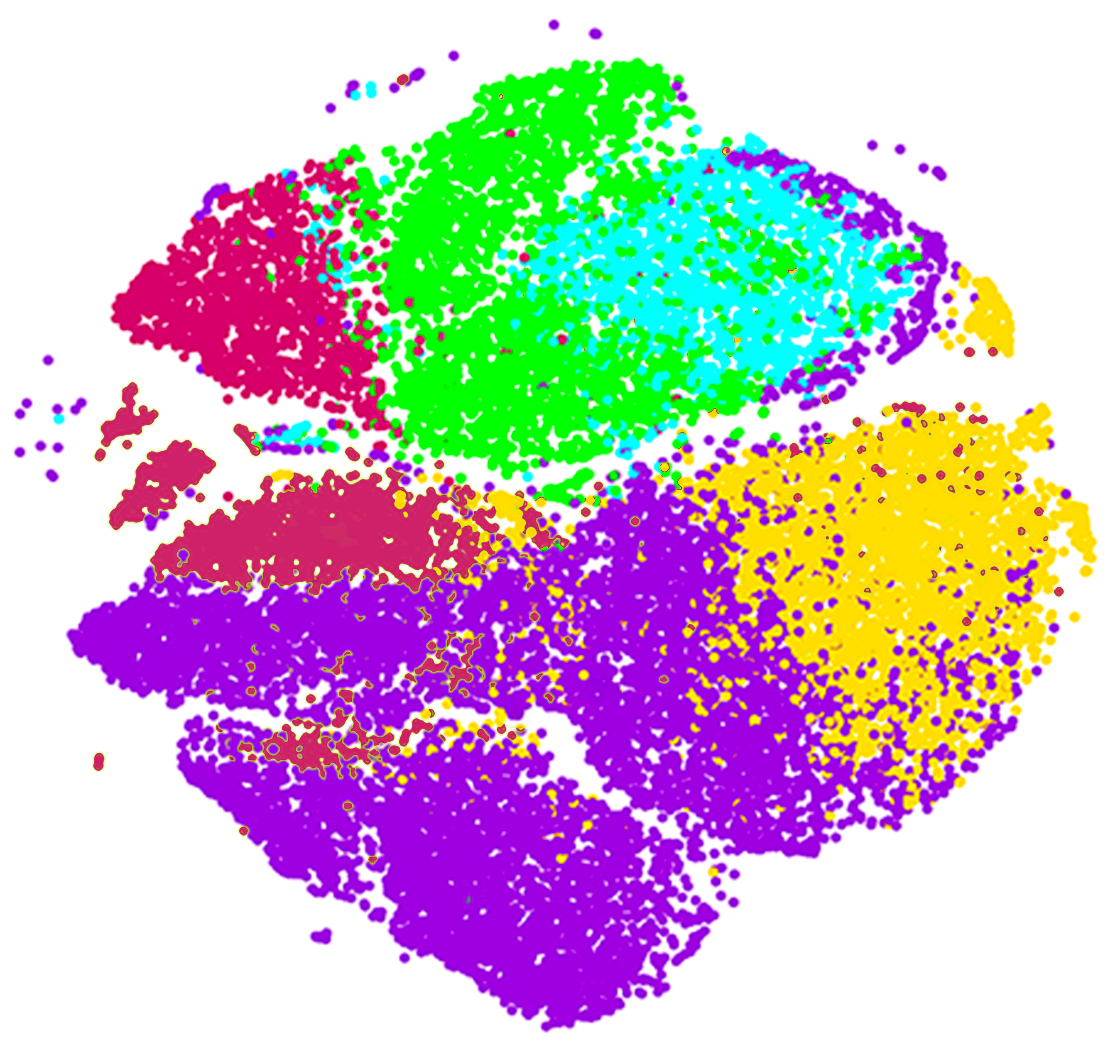

Supplement: Supplementary file 1 — Additional file 1. [file 13020_2025_1266_MOESM1_ESM.zip › Figure 4/N-A165-300bpi.png]

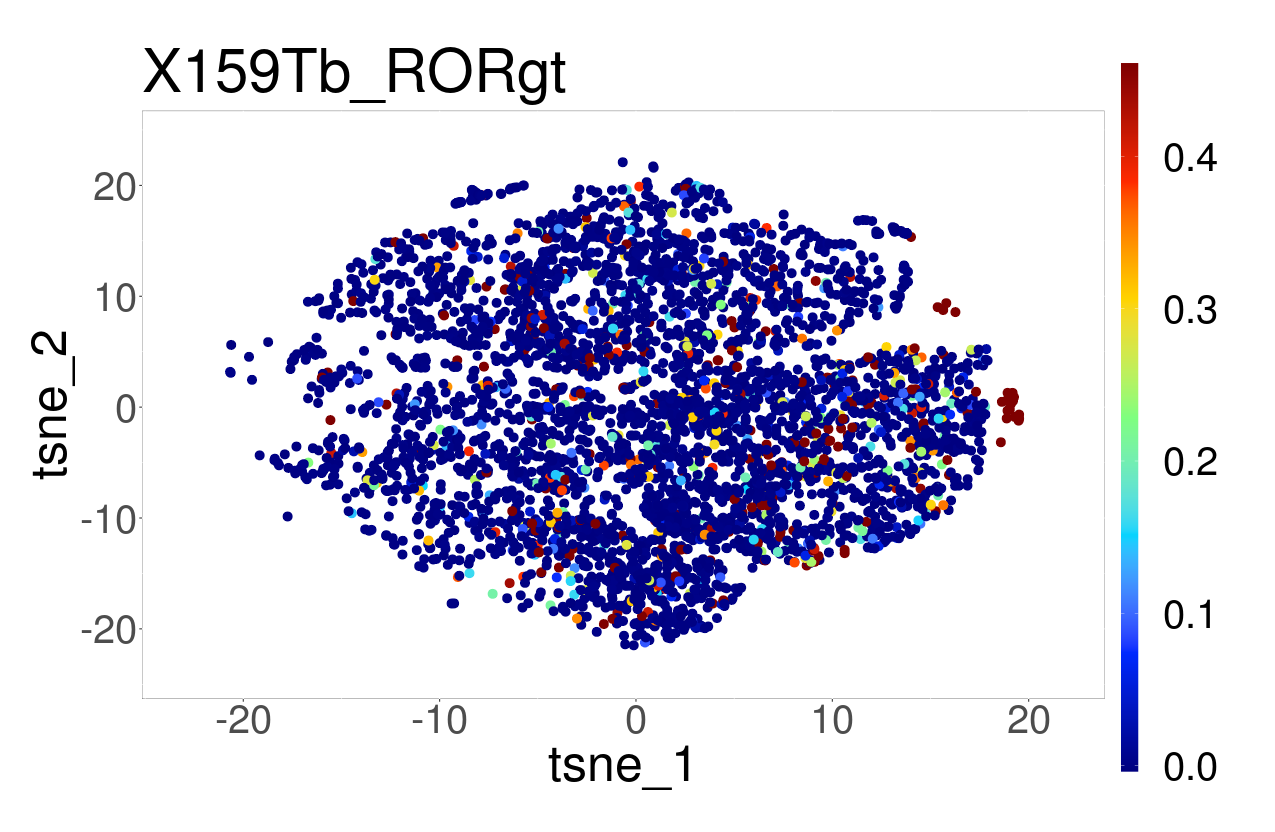

Supplement: Supplementary file 1 — Additional file 1. [file 13020_2025_1266_MOESM1_ESM.zip › Figure 4/RORγt.png]

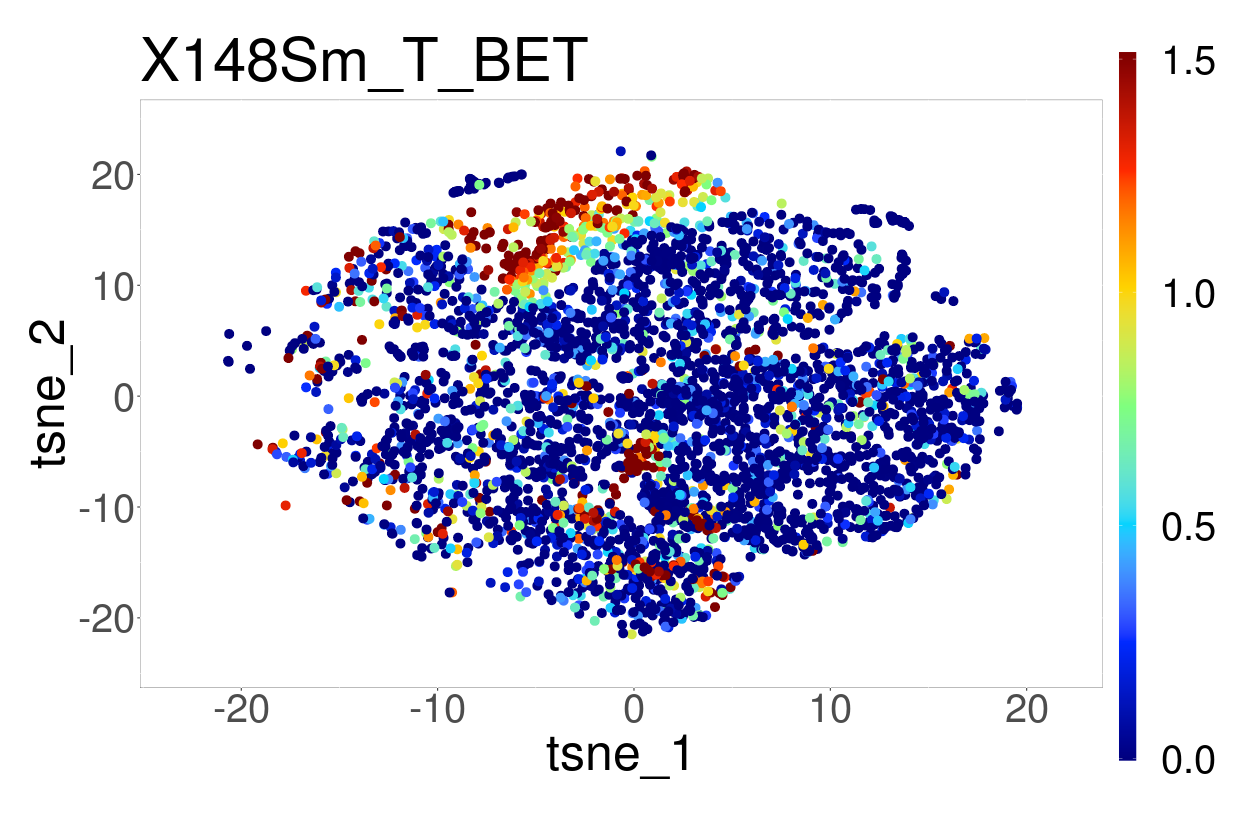

Supplement: Supplementary file 1 — Additional file 1. [file 13020_2025_1266_MOESM1_ESM.zip › Figure 4/T-bet.png]

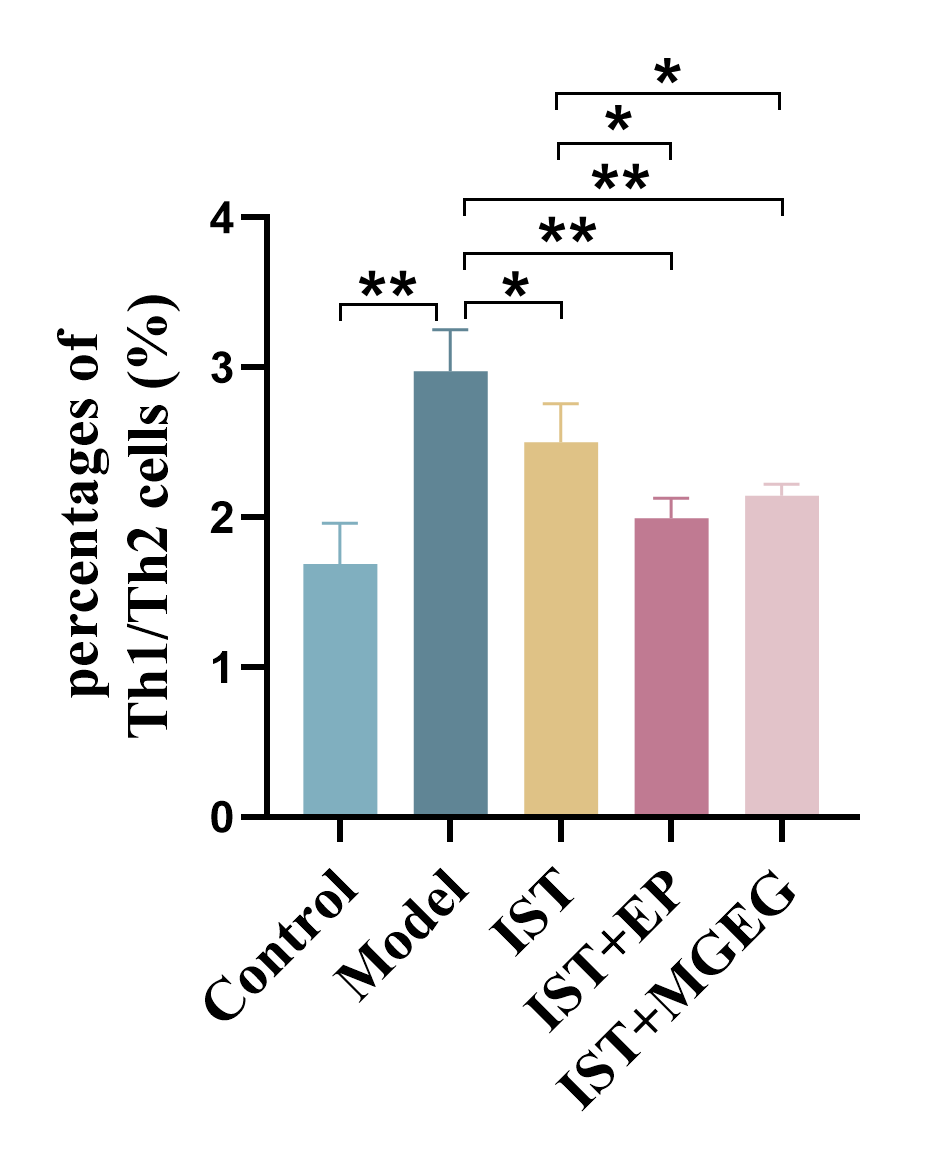

Supplement: Supplementary file 1 — Additional file 1. [file 13020_2025_1266_MOESM1_ESM.zip › Figure 4/Th1-Th2.tif]

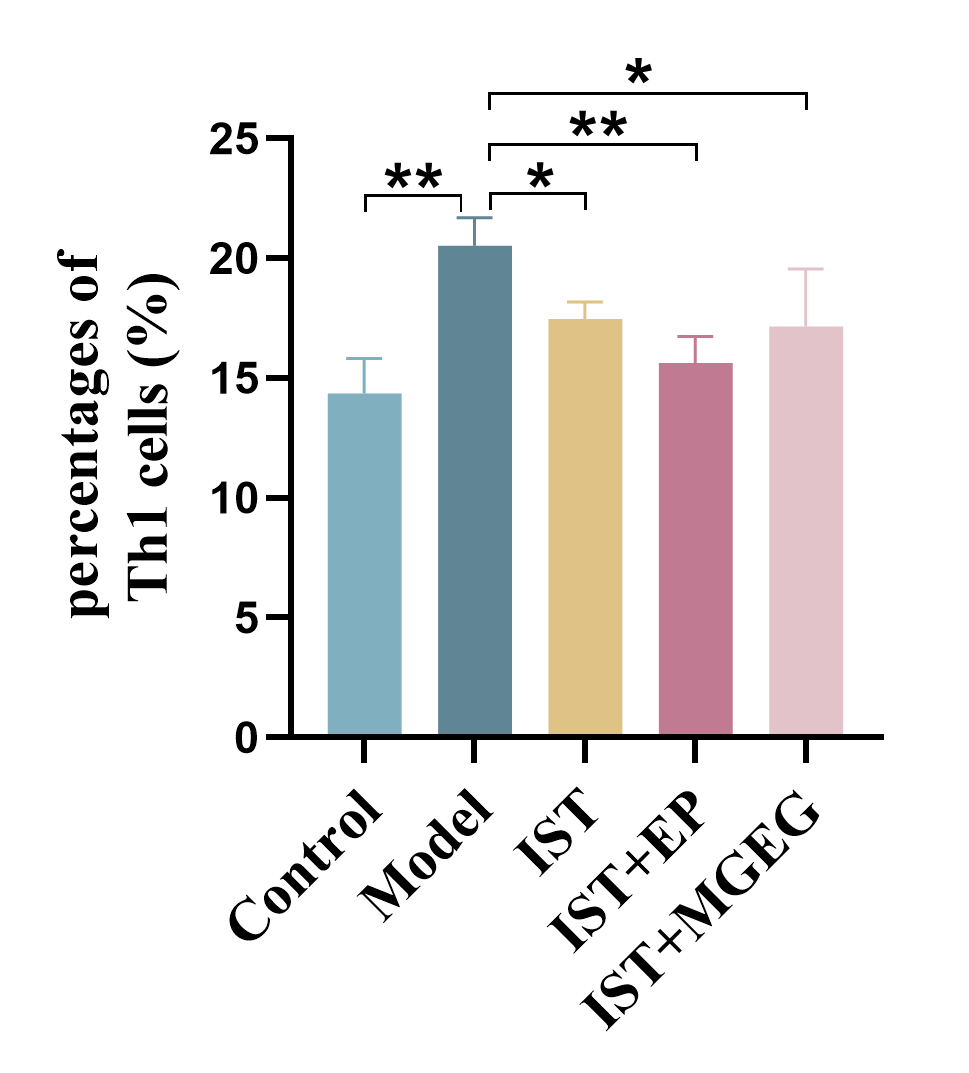

Supplement: Supplementary file 1 — Additional file 1. [file 13020_2025_1266_MOESM1_ESM.zip › Figure 4/Th1.tif]

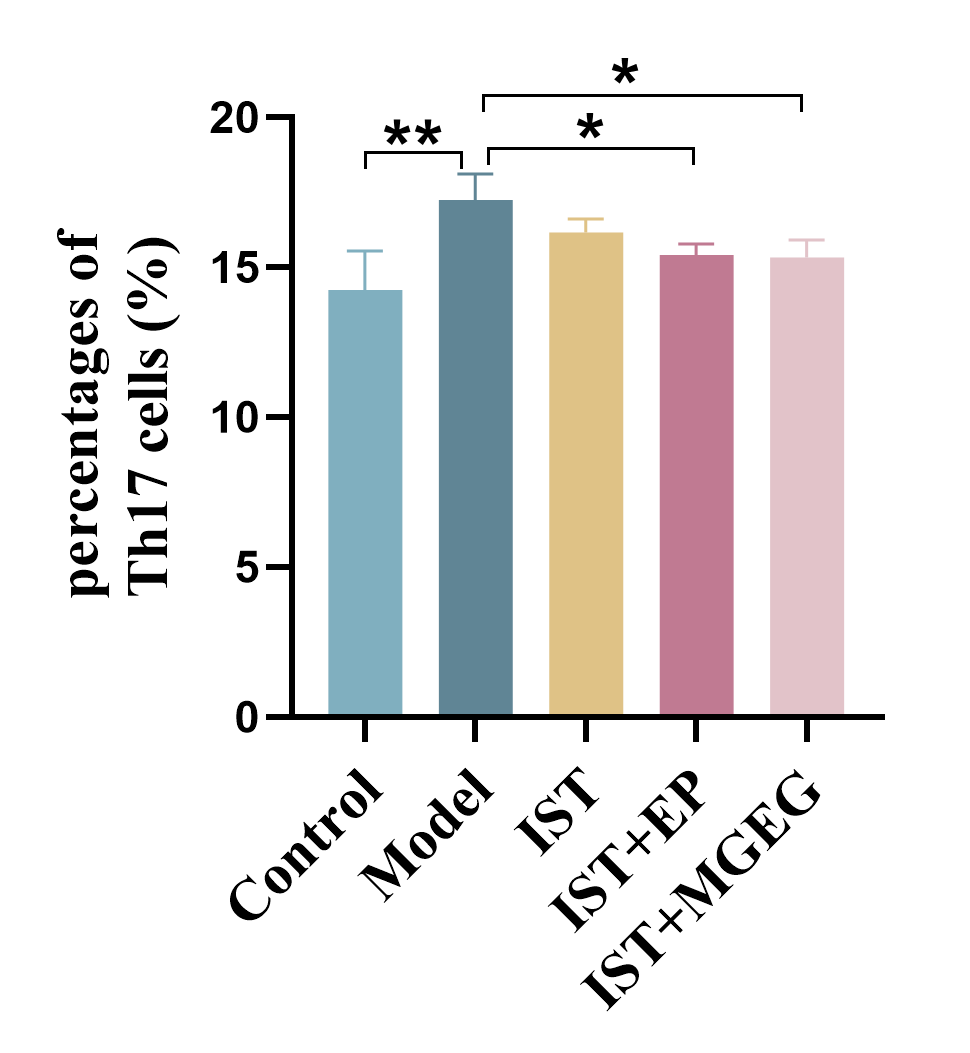

Supplement: Supplementary file 1 — Additional file 1. [file 13020_2025_1266_MOESM1_ESM.zip › Figure 4/Th17.tif]

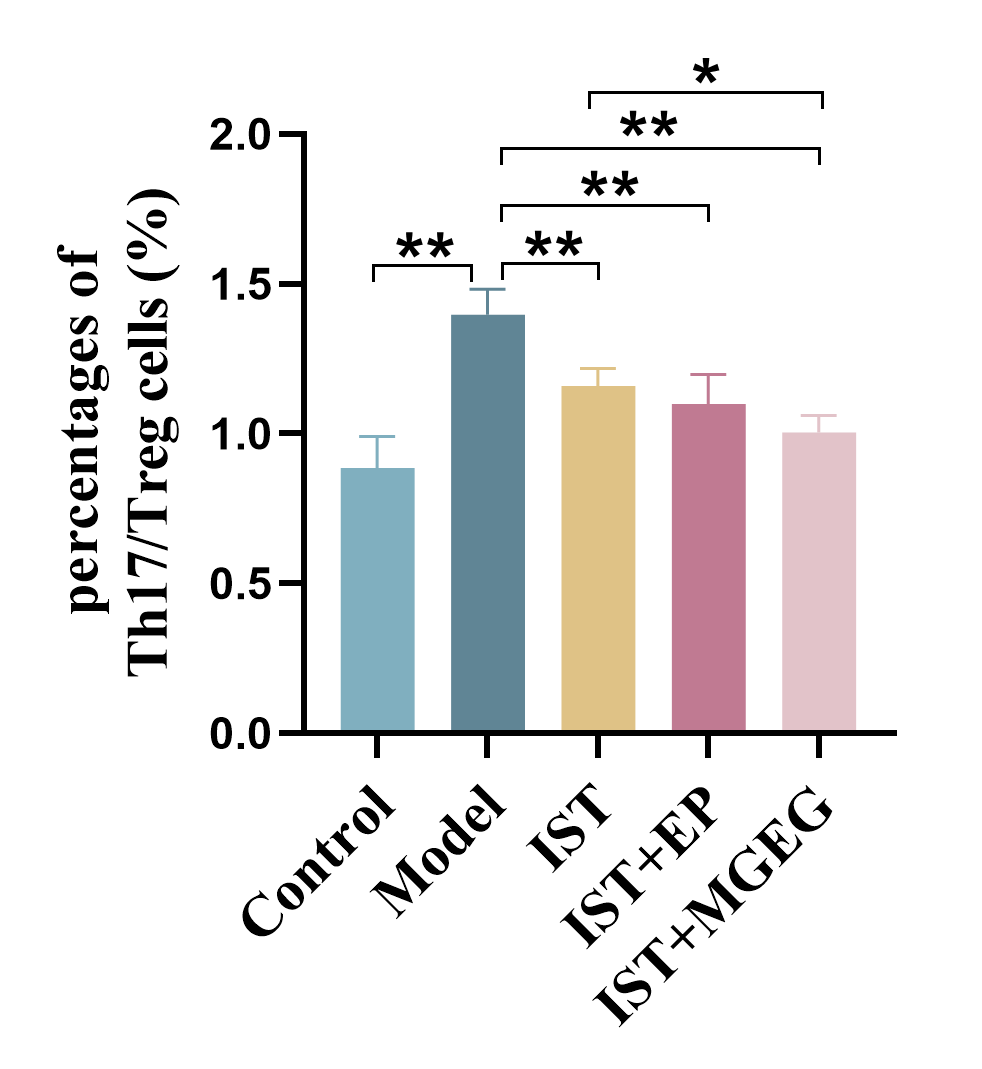

Supplement: Supplementary file 1 — Additional file 1. [file 13020_2025_1266_MOESM1_ESM.zip › Figure 4/Th17_Treg.tif]

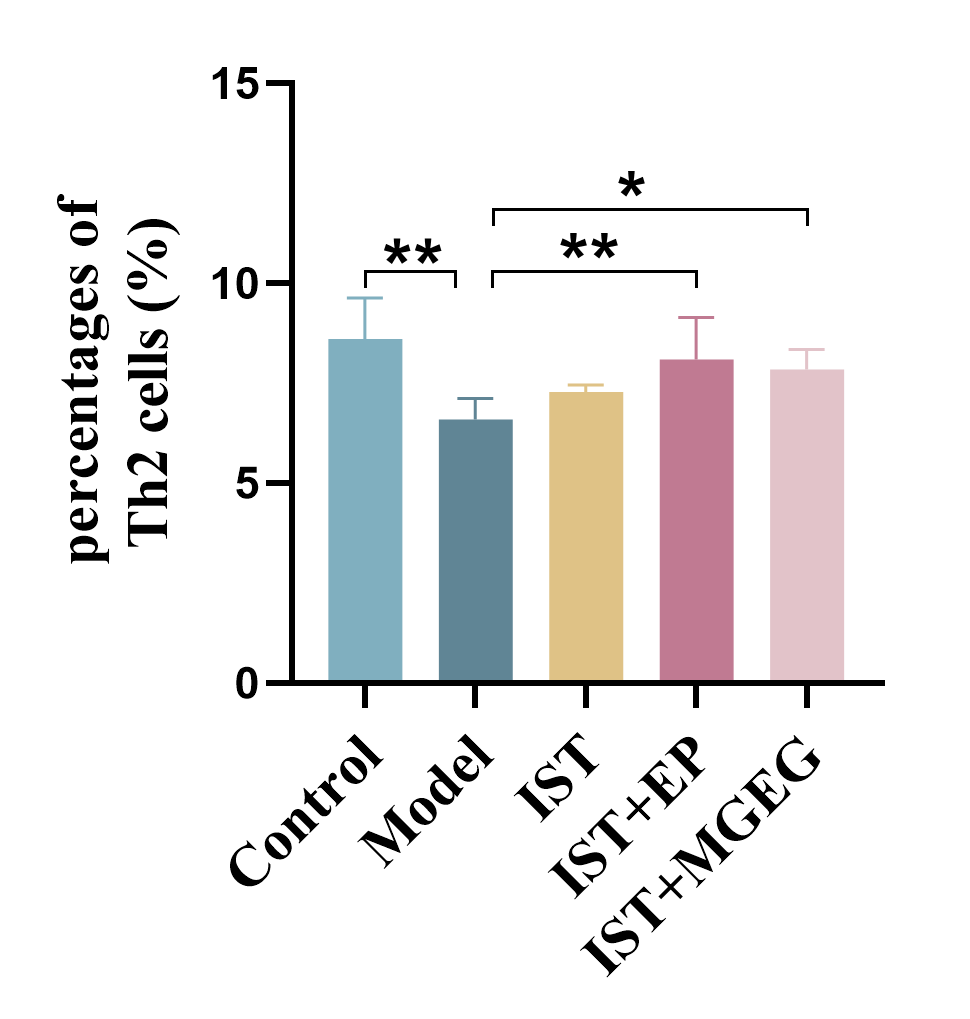

Supplement: Supplementary file 1 — Additional file 1. [file 13020_2025_1266_MOESM1_ESM.zip › Figure 4/Th2.tif]

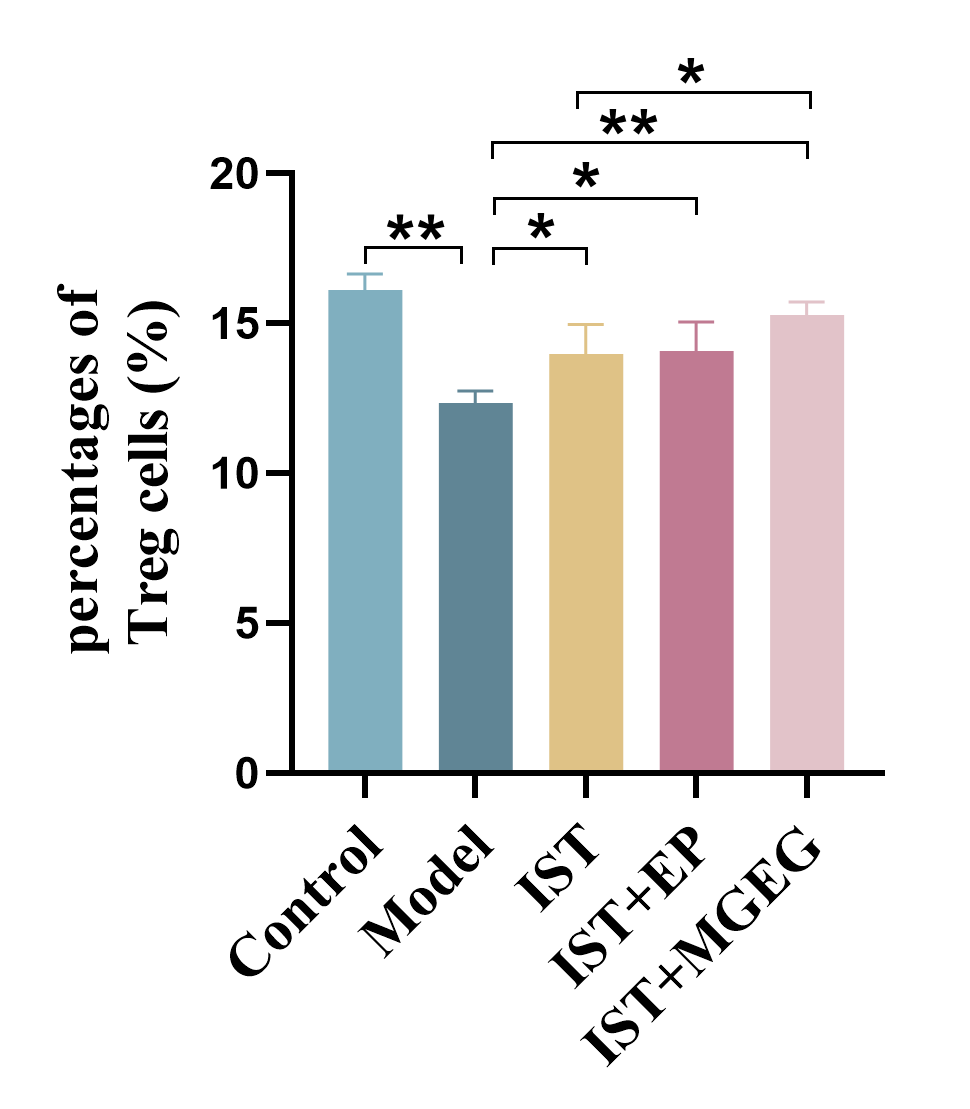

Supplement: Supplementary file 1 — Additional file 1. [file 13020_2025_1266_MOESM1_ESM.zip › Figure 4/Treg.tif]

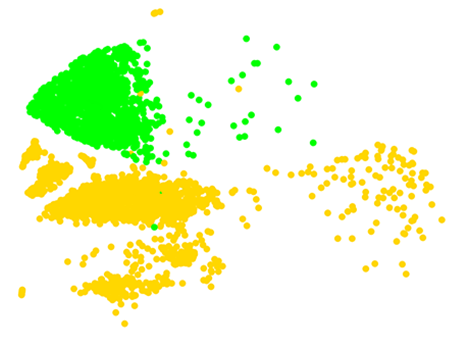

Supplement: Supplementary file 1 — Additional file 1. [file 13020_2025_1266_MOESM1_ESM.zip › Figure 5/Control.png]

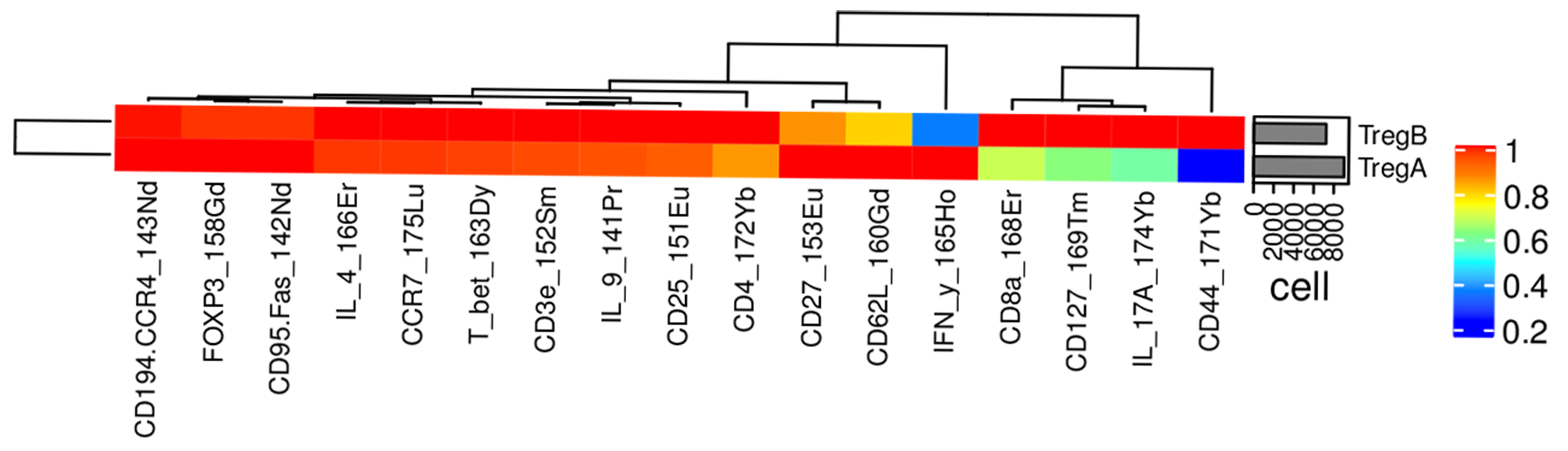

Supplement: Supplementary file 1 — Additional file 1. [file 13020_2025_1266_MOESM1_ESM.zip › Figure 5/heatmap.tif]

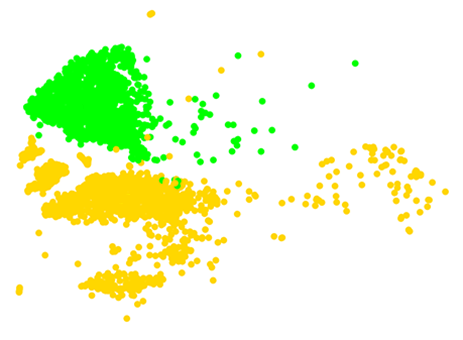

Supplement: Supplementary file 1 — Additional file 1. [file 13020_2025_1266_MOESM1_ESM.zip › Figure 5/IST+EP.png]

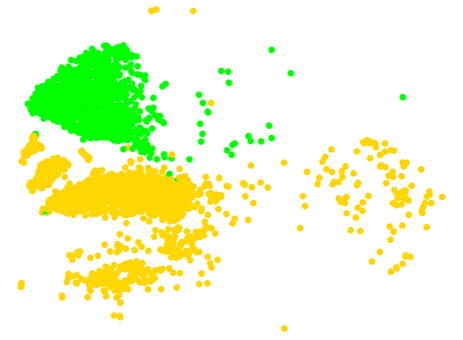

Supplement: Supplementary file 1 — Additional file 1. [file 13020_2025_1266_MOESM1_ESM.zip › Figure 5/IST+MGEG.png]

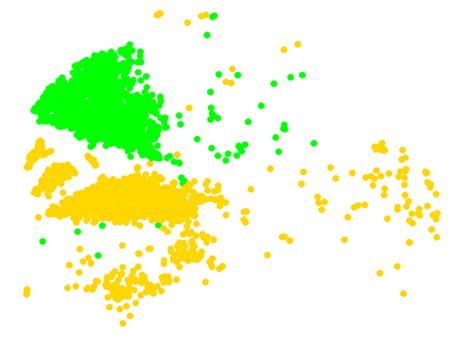

Supplement: Supplementary file 1 — Additional file 1. [file 13020_2025_1266_MOESM1_ESM.zip › Figure 5/IST.png]

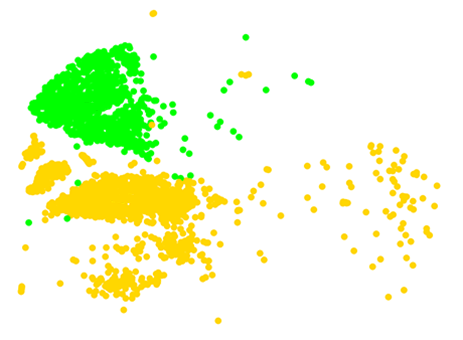

Supplement: Supplementary file 1 — Additional file 1. [file 13020_2025_1266_MOESM1_ESM.zip › Figure 5/Model-300dpi.png]

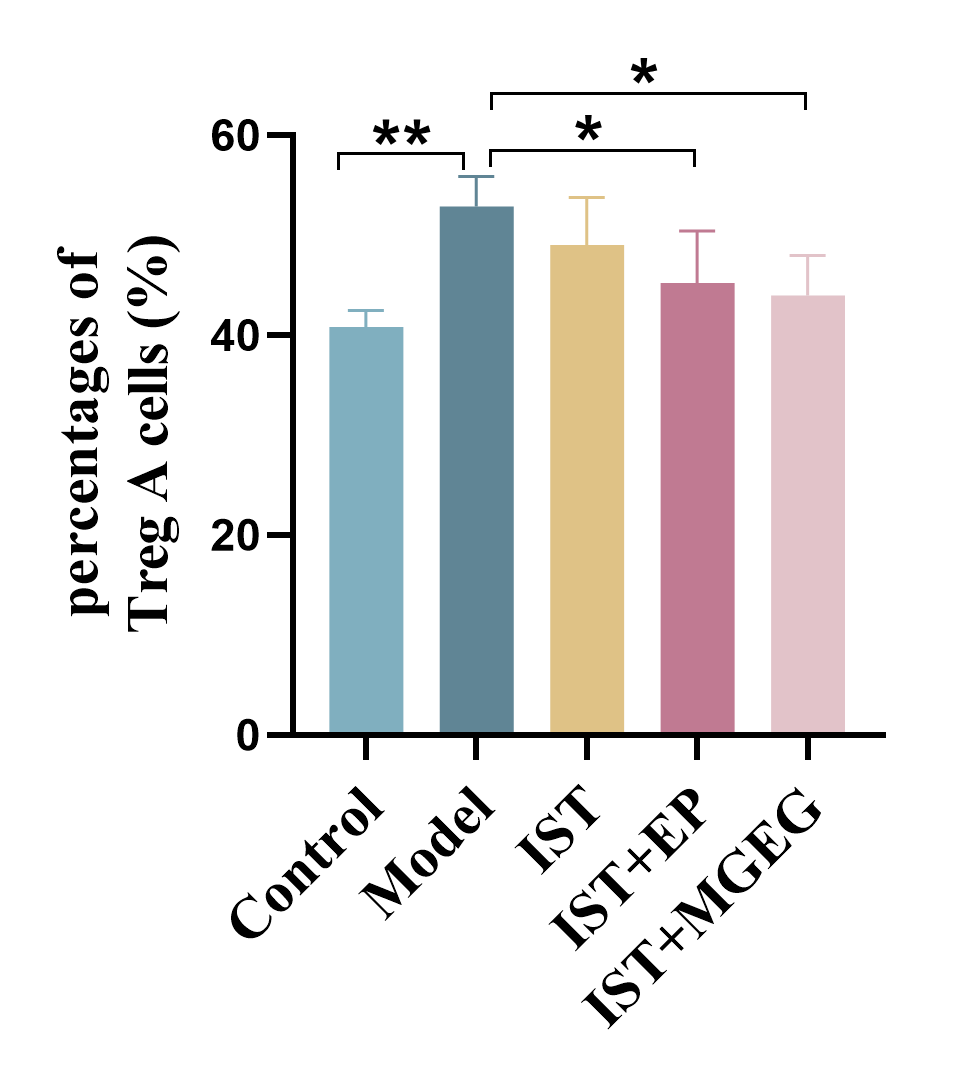

Supplement: Supplementary file 1 — Additional file 1. [file 13020_2025_1266_MOESM1_ESM.zip › Figure 5/Treg A.tif]

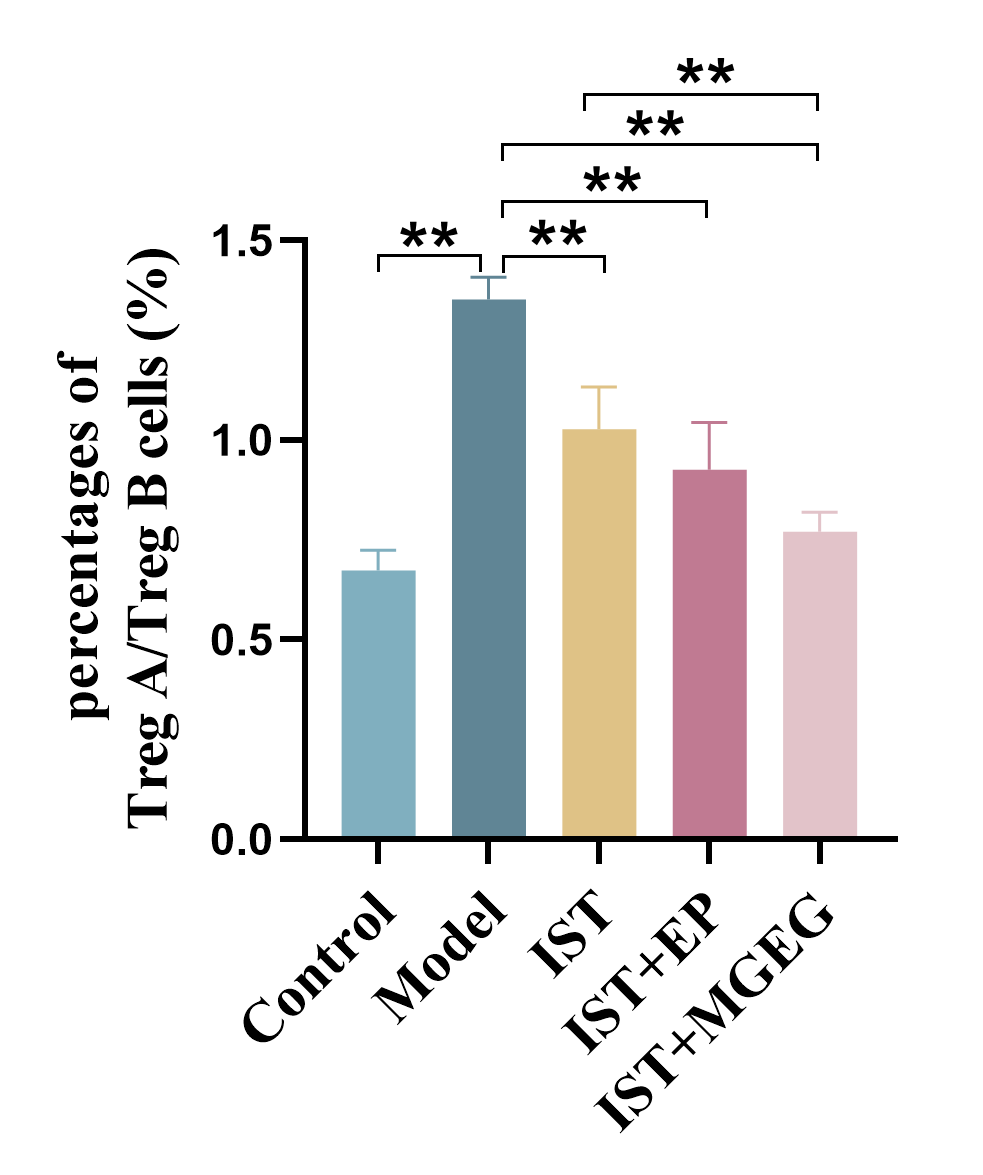

Supplement: Supplementary file 1 — Additional file 1. [file 13020_2025_1266_MOESM1_ESM.zip › Figure 5/Treg A_Treg B.tif]

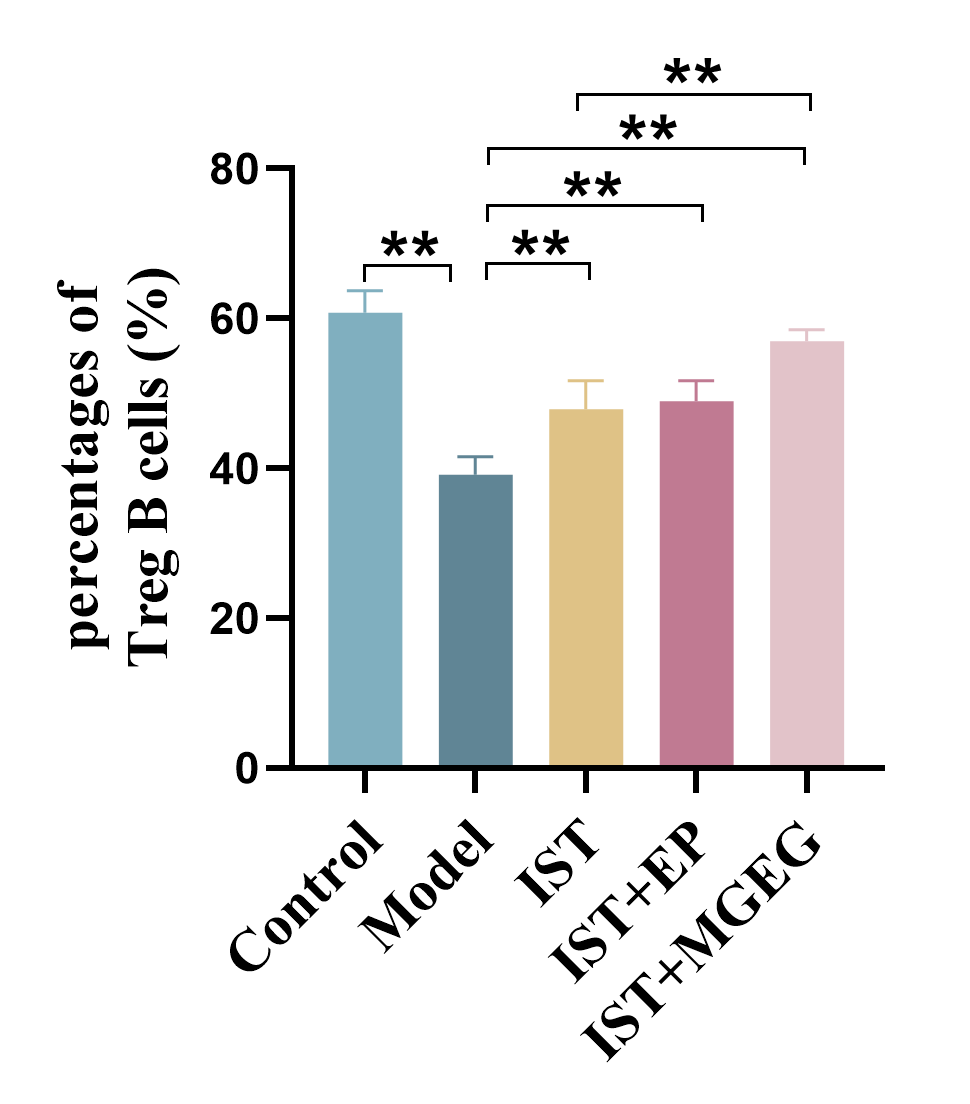

Supplement: Supplementary file 1 — Additional file 1. [file 13020_2025_1266_MOESM1_ESM.zip › Figure 5/Treg B.tif]

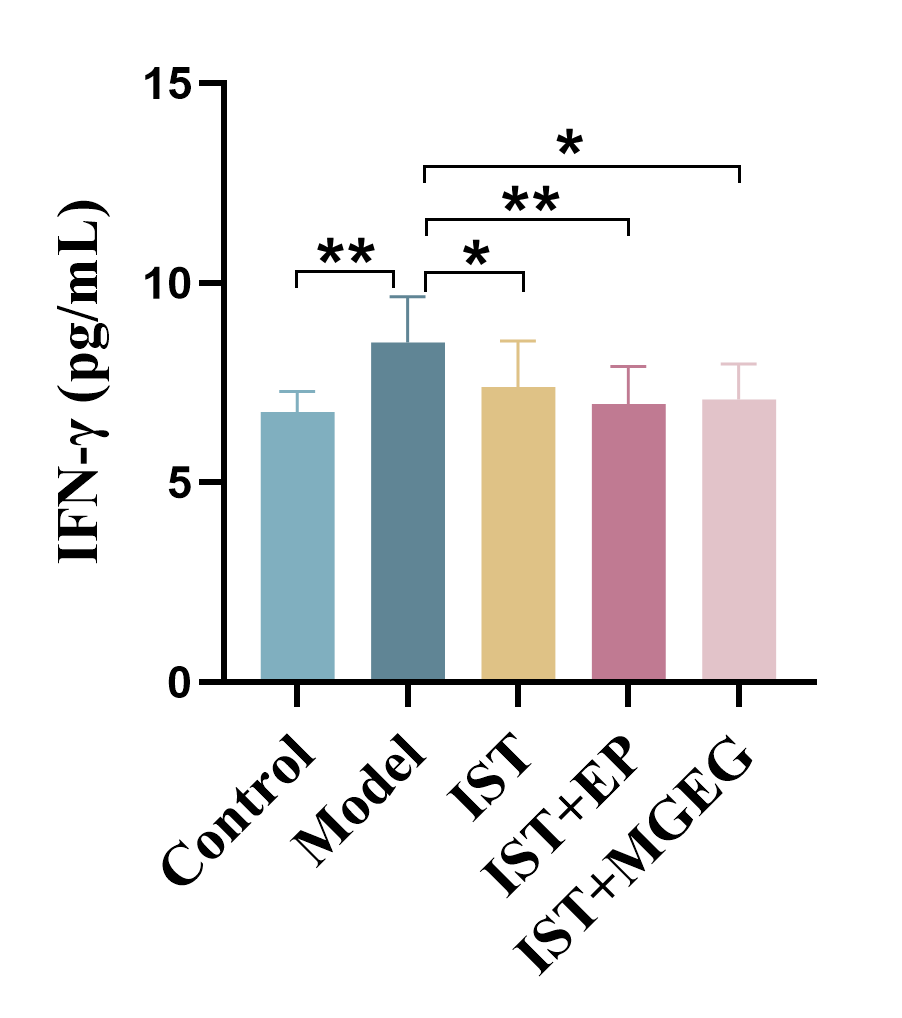

Supplement: Supplementary file 1 — Additional file 1. [file 13020_2025_1266_MOESM1_ESM.zip › Figure 6/IFN-γ.tif]

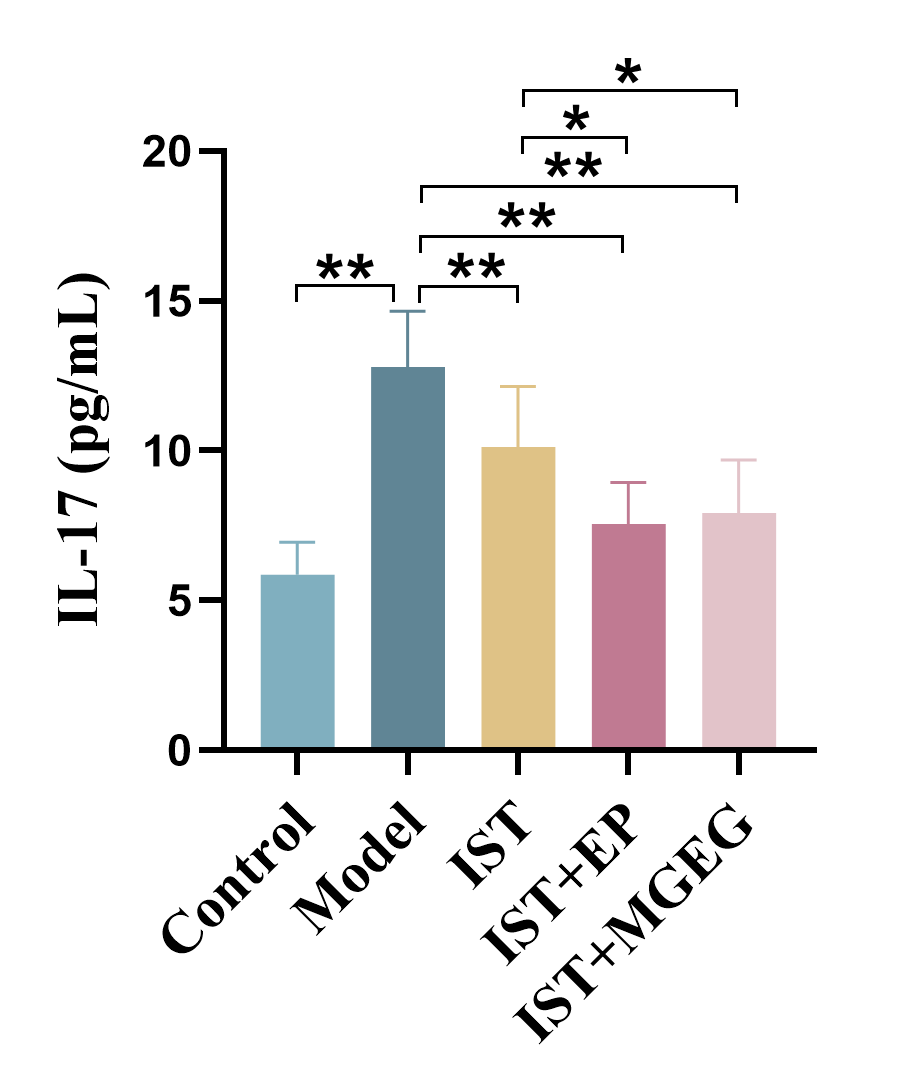

Supplement: Supplementary file 1 — Additional file 1. [file 13020_2025_1266_MOESM1_ESM.zip › Figure 6/IL-17.tif]

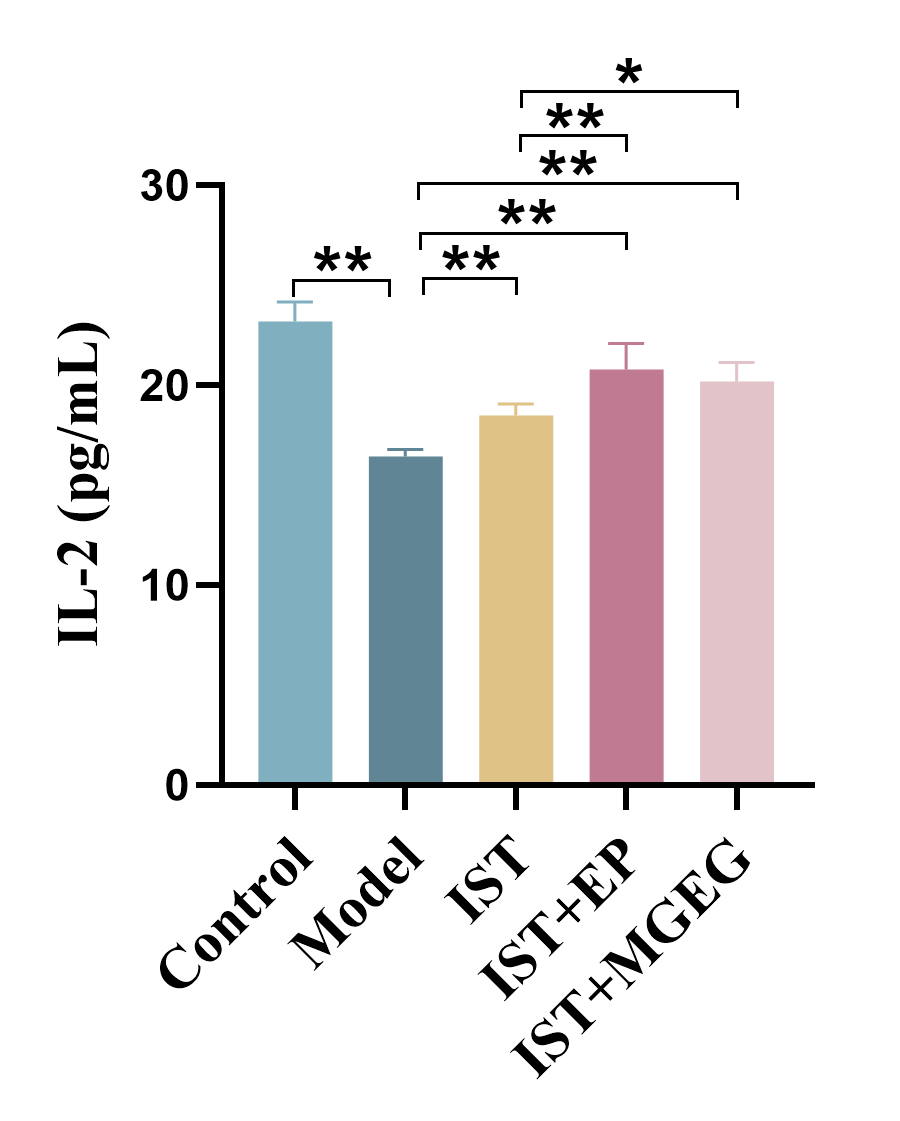

Supplement: Supplementary file 1 — Additional file 1. [file 13020_2025_1266_MOESM1_ESM.zip › Figure 6/IL-2.tif]

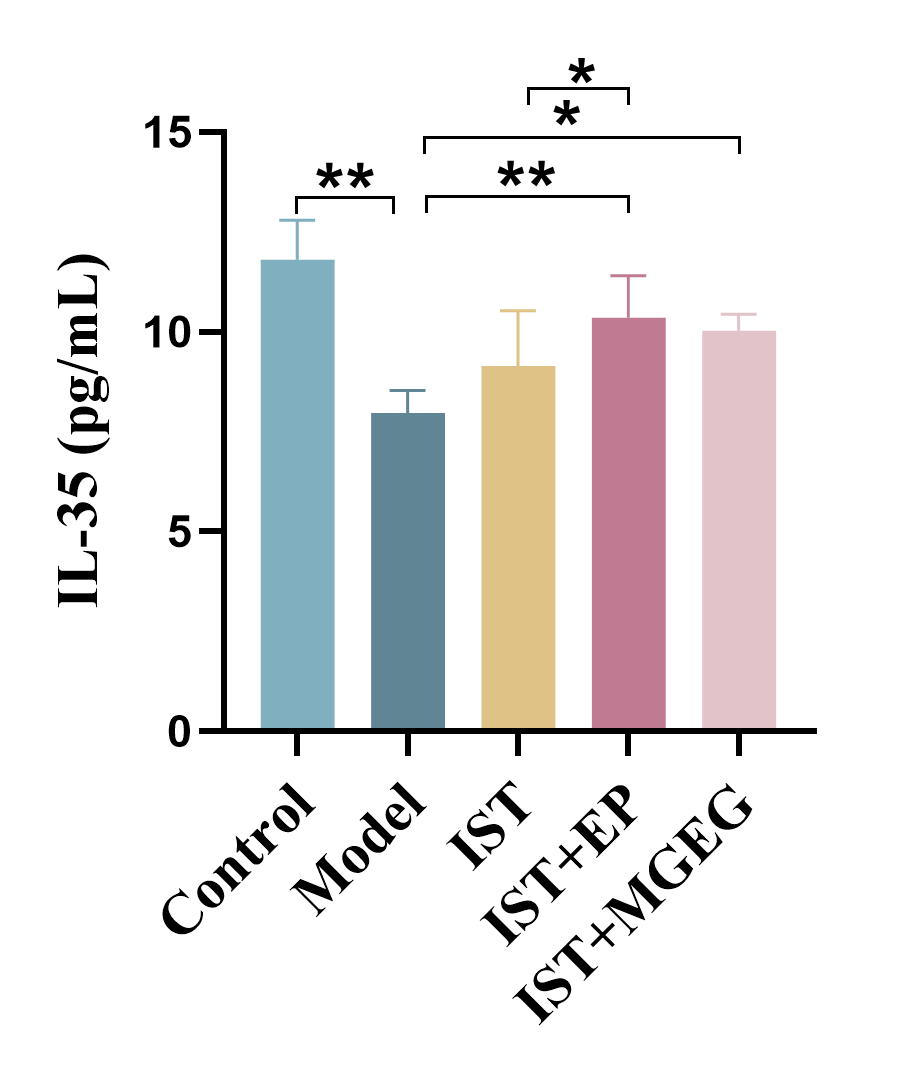

Supplement: Supplementary file 1 — Additional file 1. [file 13020_2025_1266_MOESM1_ESM.zip › Figure 6/IL-35.tif]

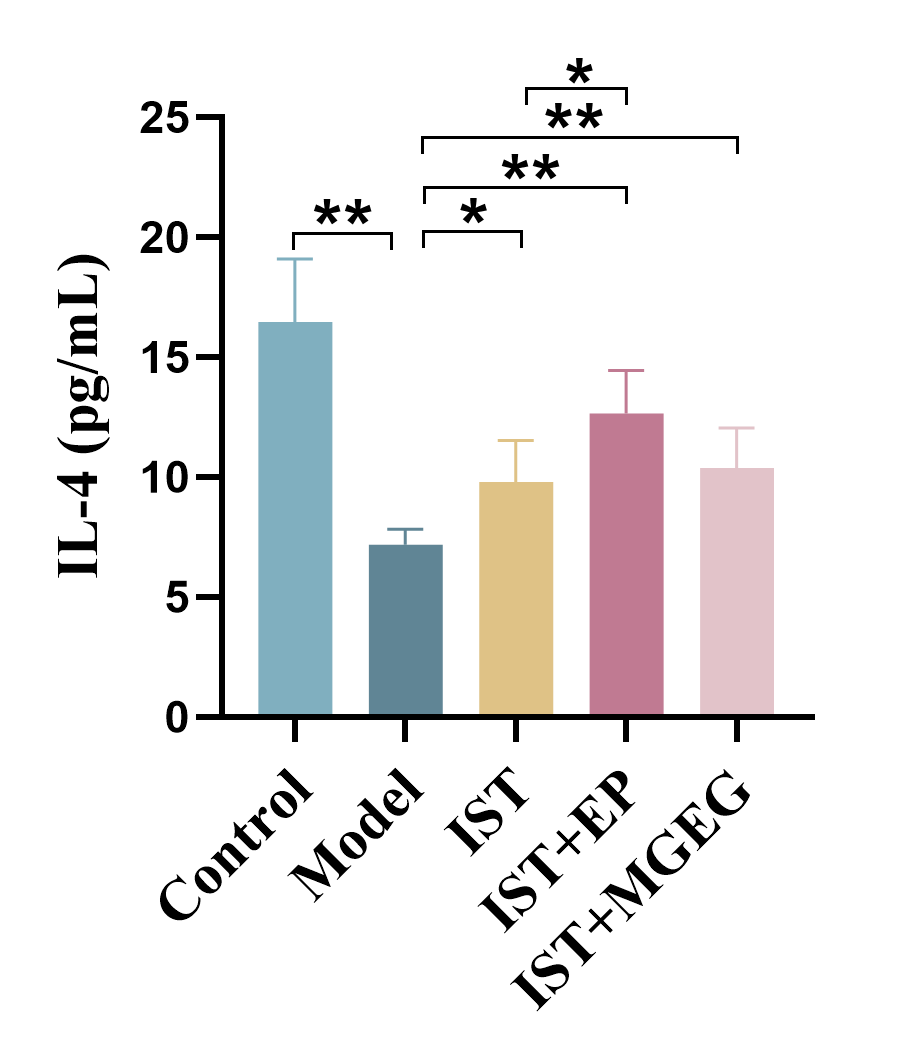

Supplement: Supplementary file 1 — Additional file 1. [file 13020_2025_1266_MOESM1_ESM.zip › Figure 6/IL-4.tif]

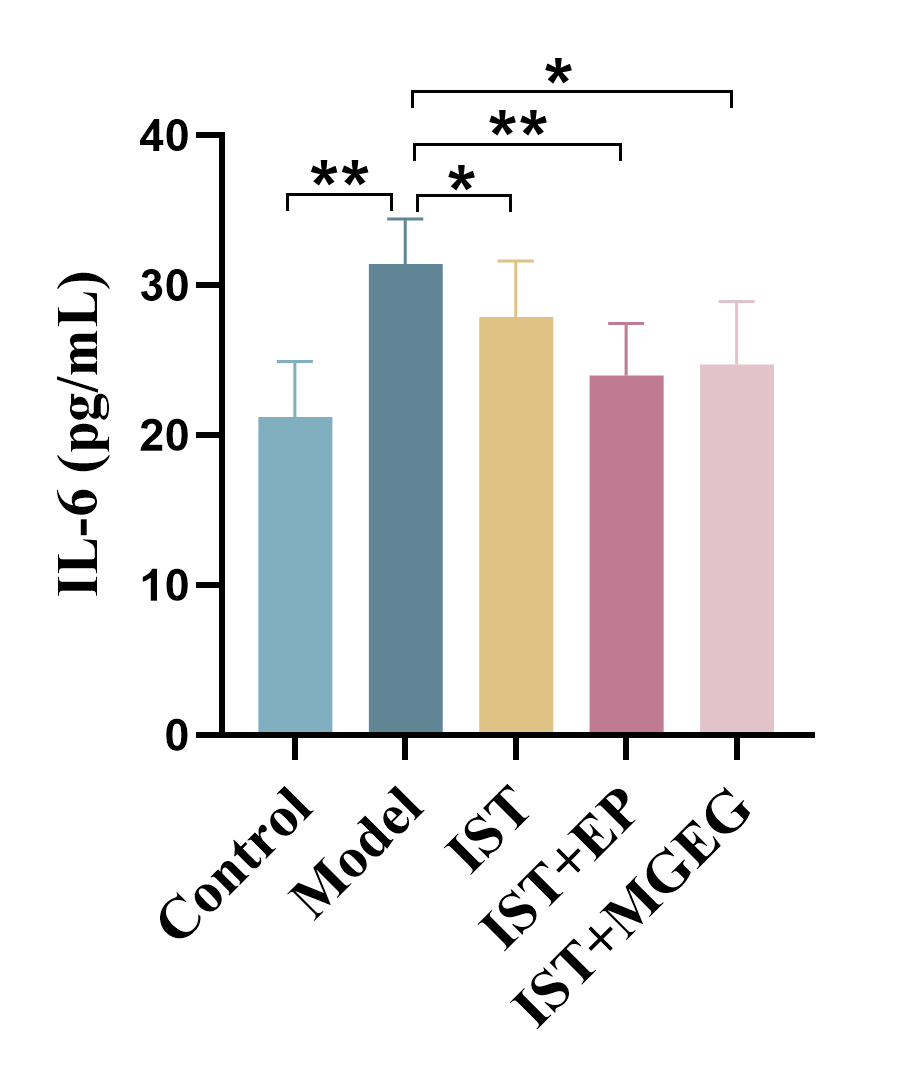

Supplement: Supplementary file 1 — Additional file 1. [file 13020_2025_1266_MOESM1_ESM.zip › Figure 6/IL-6.tif]

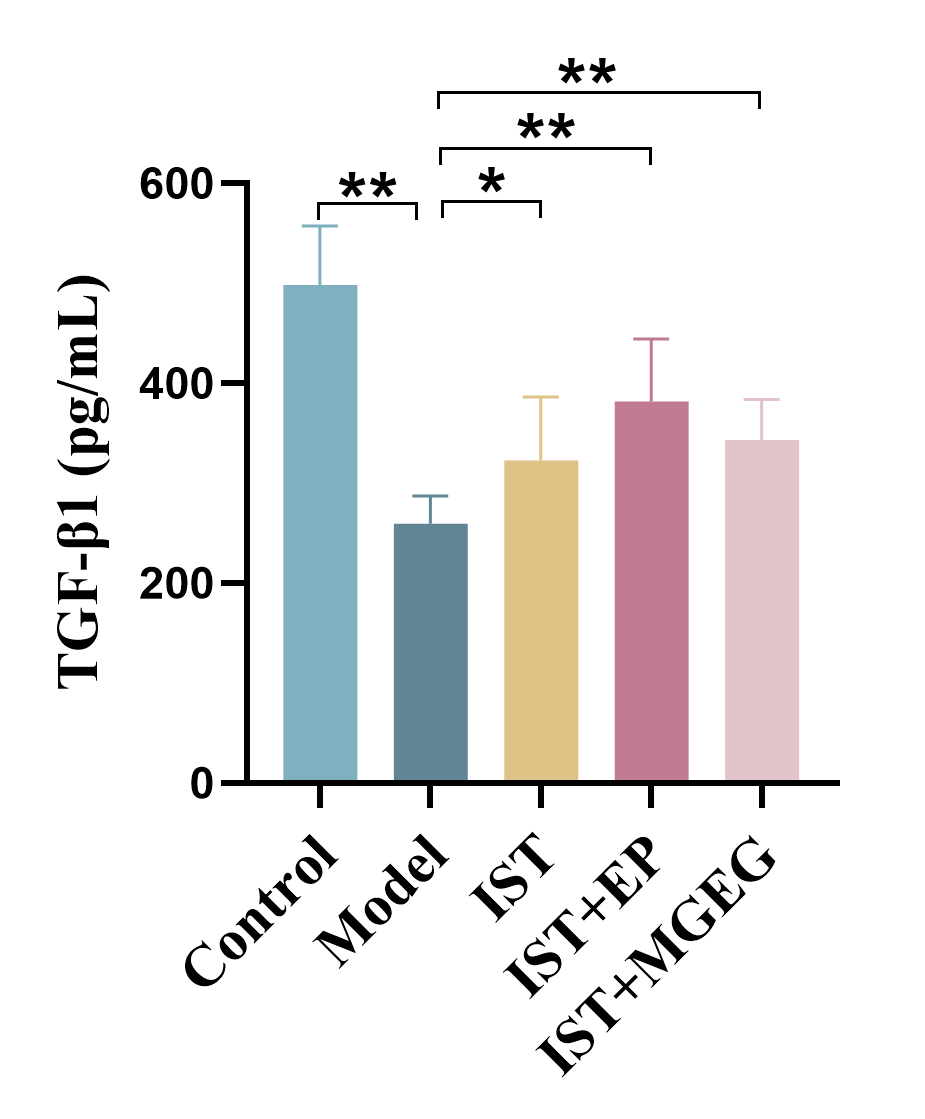

Supplement: Supplementary file 1 — Additional file 1. [file 13020_2025_1266_MOESM1_ESM.zip › Figure 6/TGF-β1.tif]

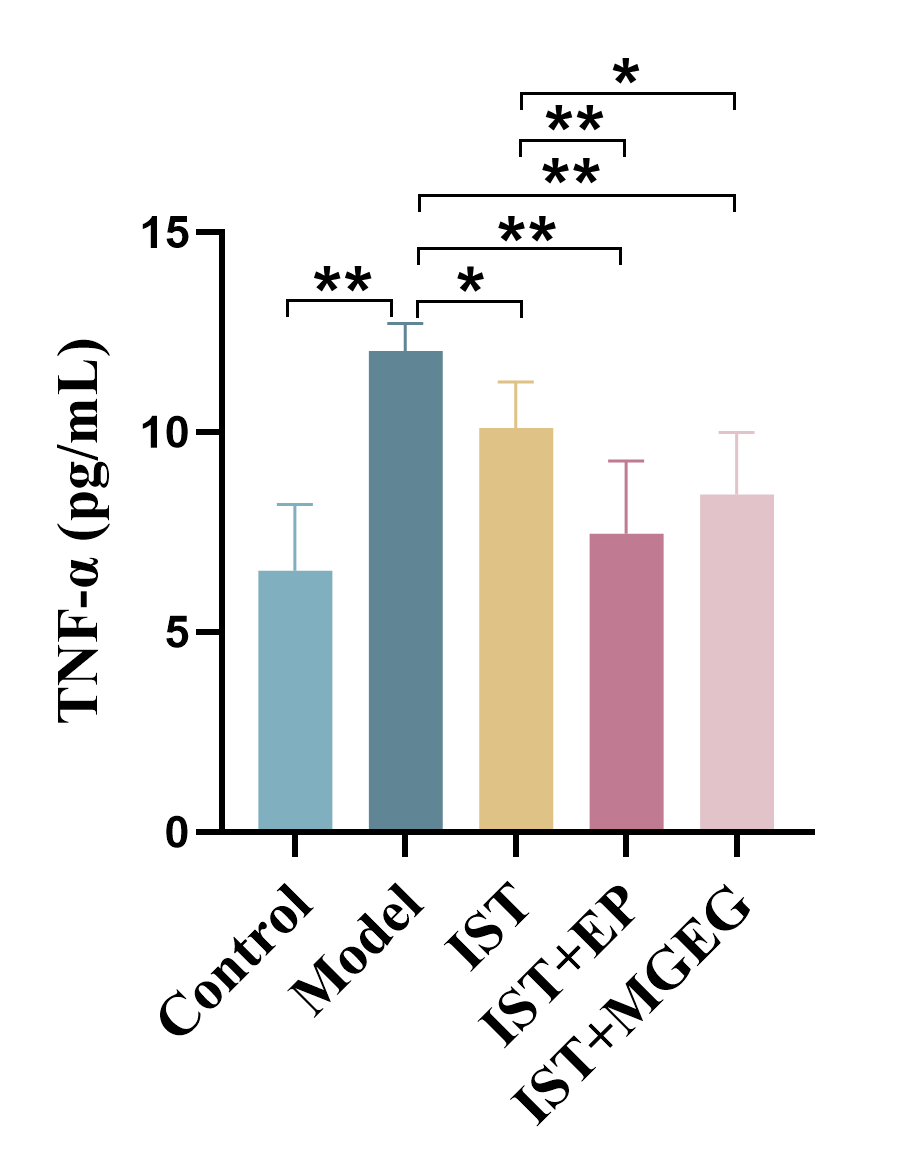

Supplement: Supplementary file 1 — Additional file 1. [file 13020_2025_1266_MOESM1_ESM.zip › Figure 6/TNF-α.tif]

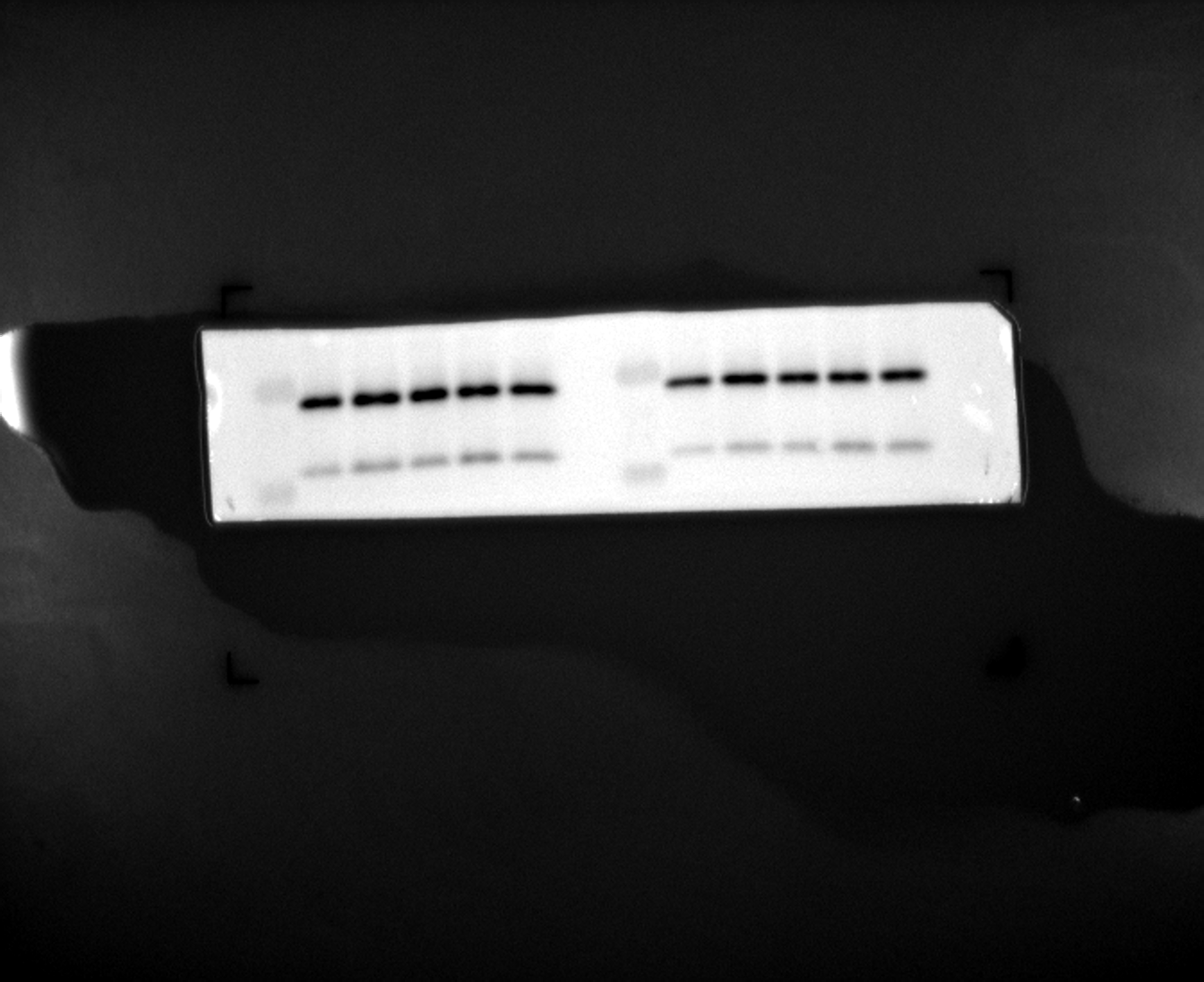

Supplement: Supplementary file 1 — Additional file 1. [file 13020_2025_1266_MOESM1_ESM.zip › Figure 7/bands/Bcl-2 1 (L Figure) HC.Tif]

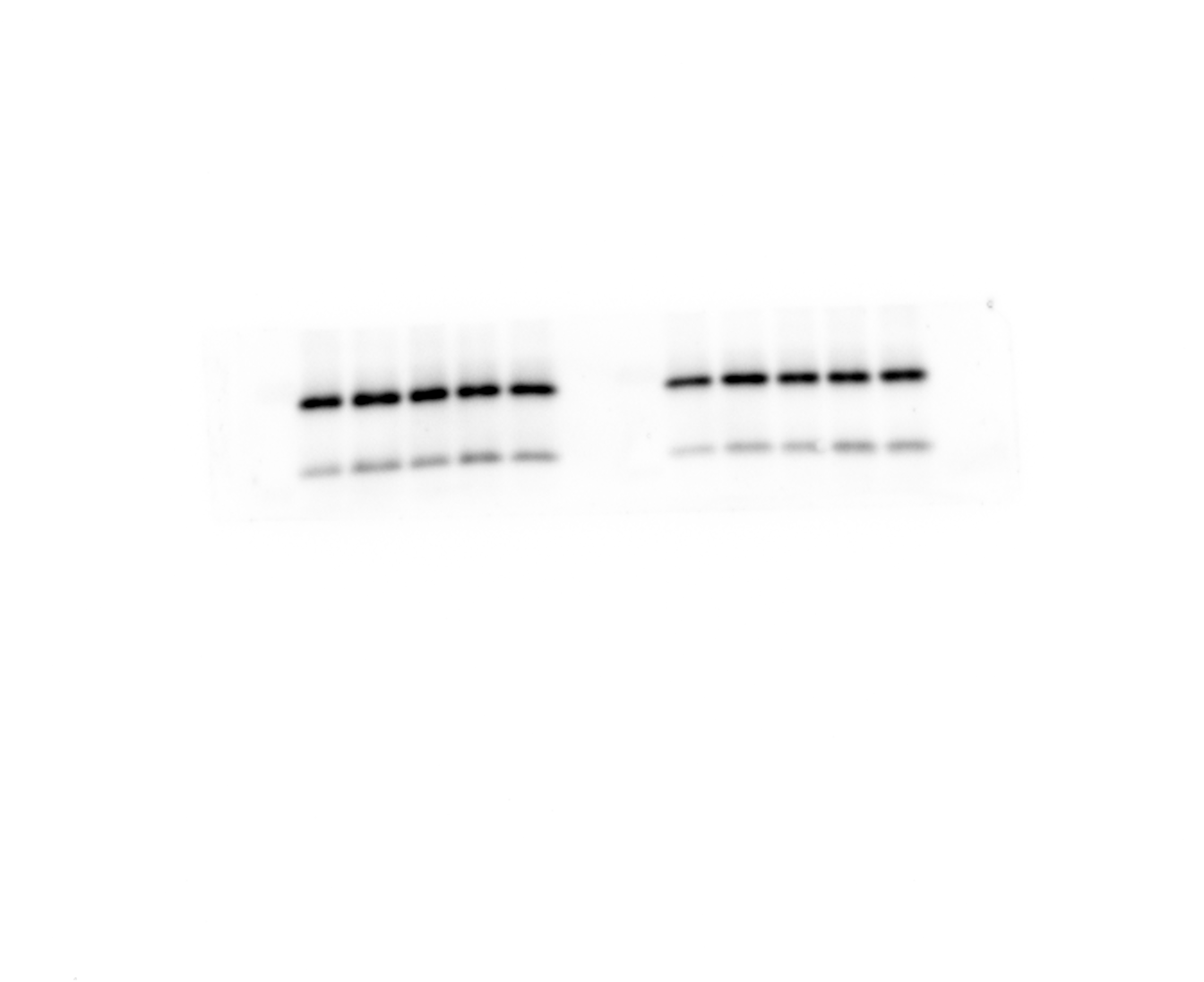

Supplement: Supplementary file 1 — Additional file 1. [file 13020_2025_1266_MOESM1_ESM.zip › Figure 7/bands/Bcl-2 1 (L Figure).Tif]

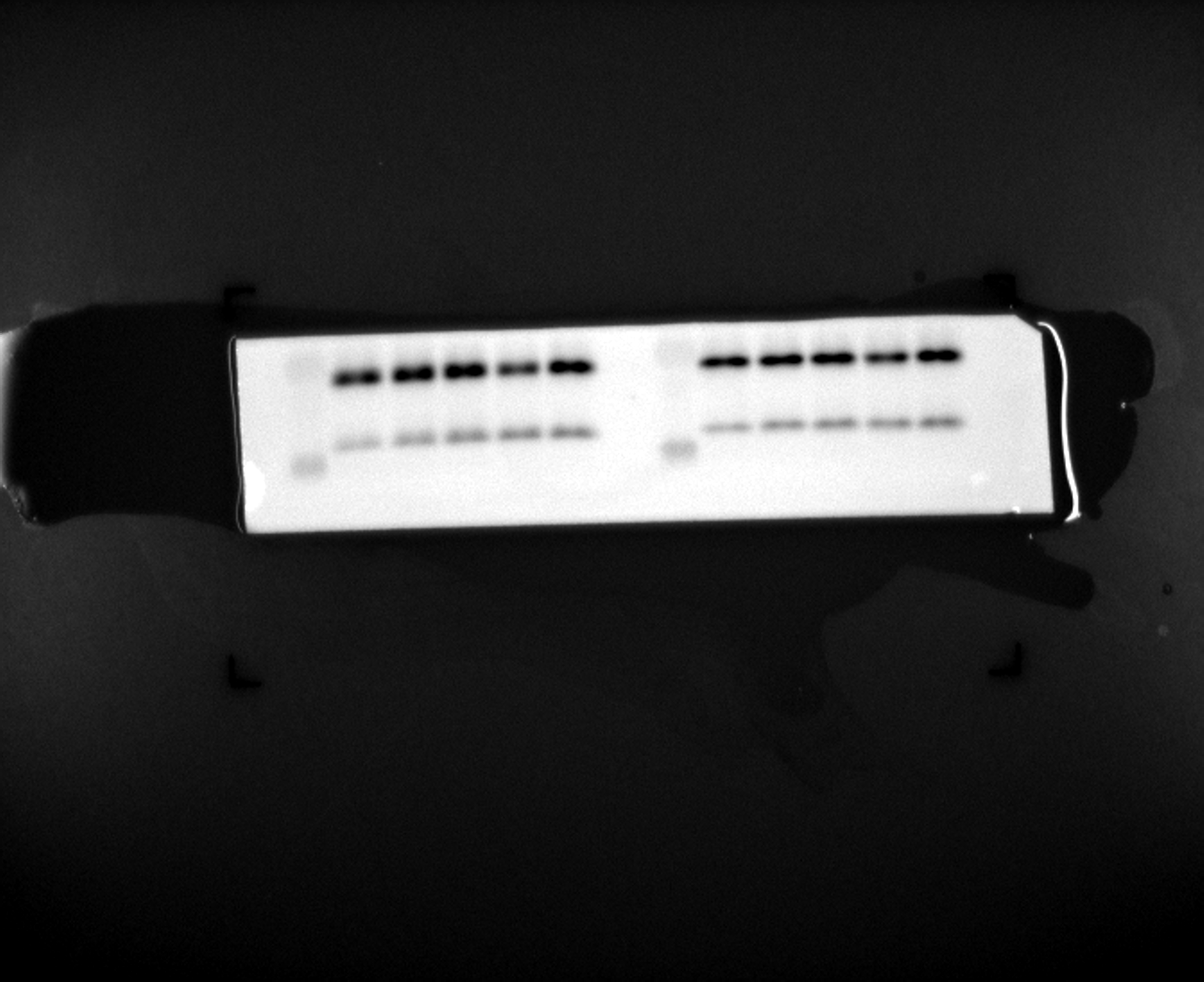

Supplement: Supplementary file 1 — Additional file 1. [file 13020_2025_1266_MOESM1_ESM.zip › Figure 7/bands/Bcl-2 2 R HC.Tif]

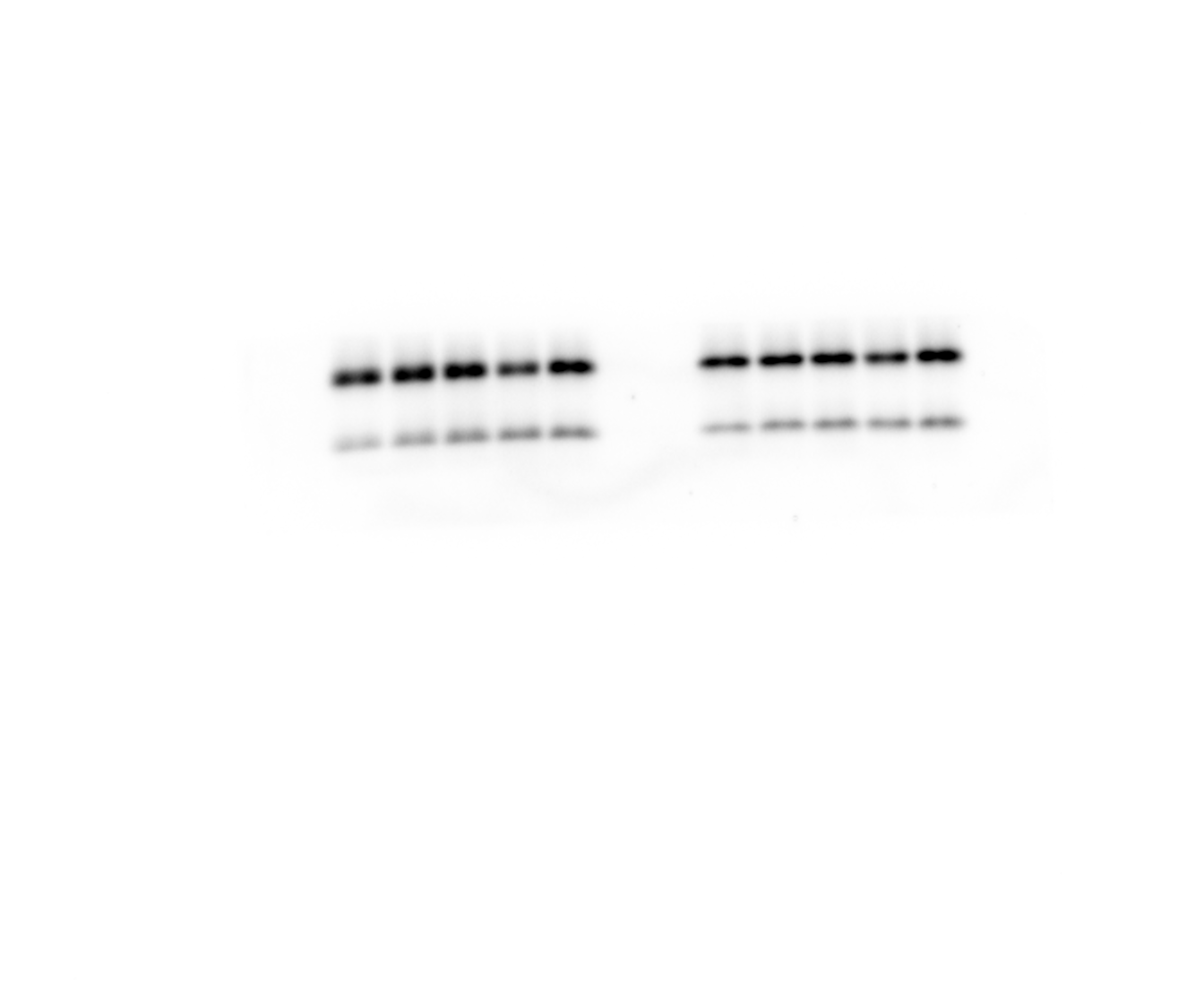

Supplement: Supplementary file 1 — Additional file 1. [file 13020_2025_1266_MOESM1_ESM.zip › Figure 7/bands/Bcl-2 2 R.Tif]

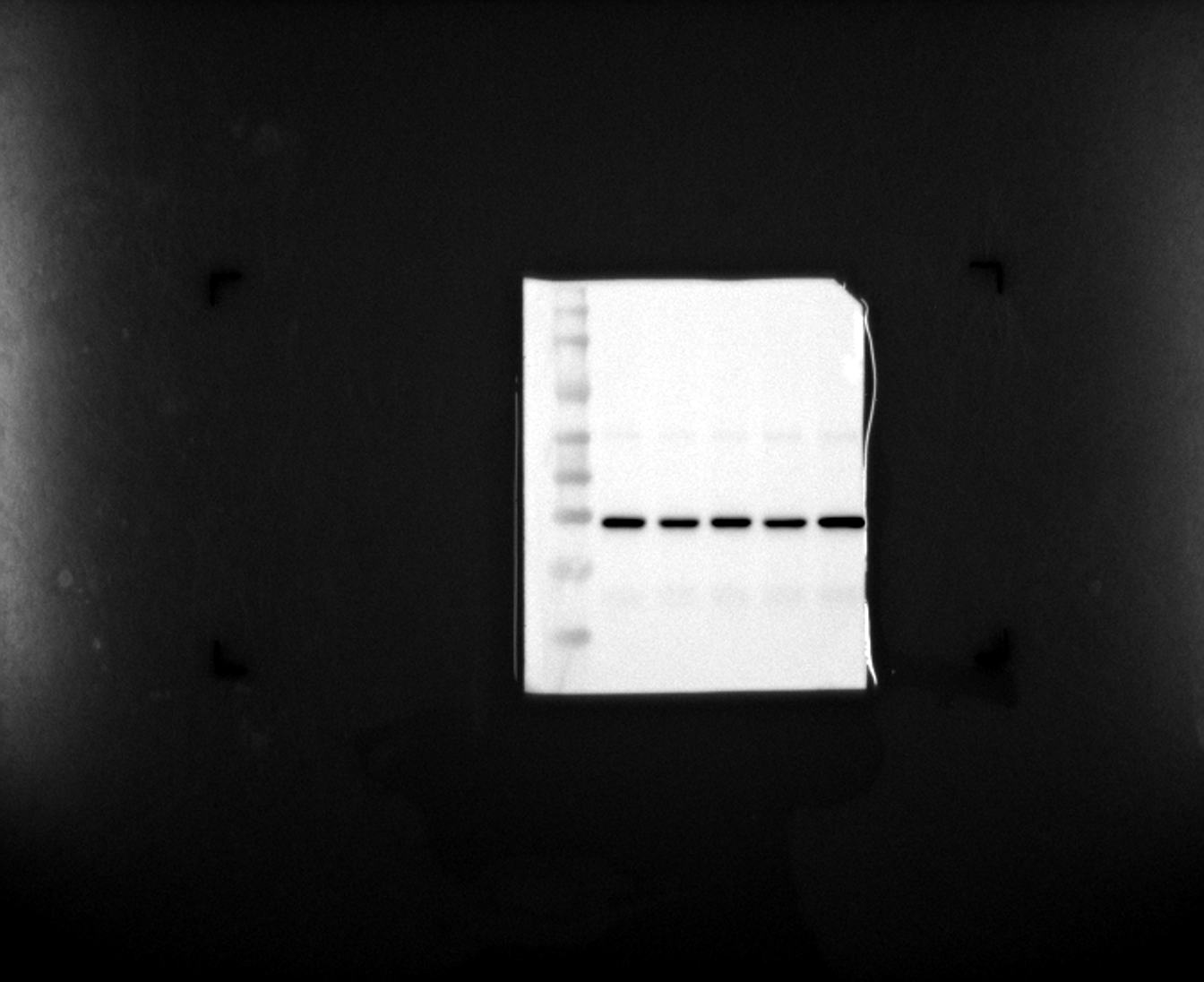

Supplement: Supplementary file 1 — Additional file 1. [file 13020_2025_1266_MOESM1_ESM.zip › Figure 7/bands/Bcl-2 3 HC.Tif]

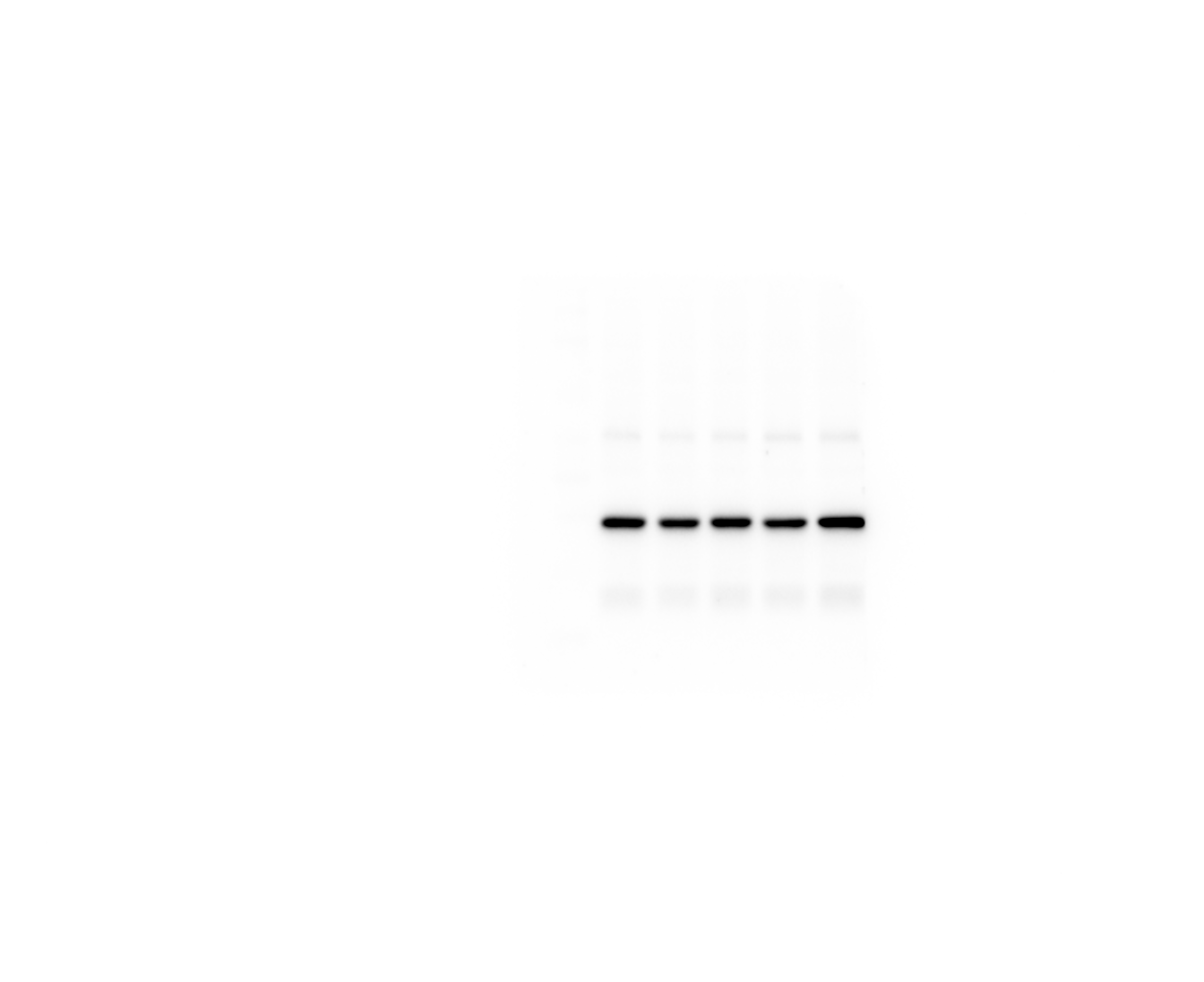

Supplement: Supplementary file 1 — Additional file 1. [file 13020_2025_1266_MOESM1_ESM.zip › Figure 7/bands/Bcl-2 3.Tif]

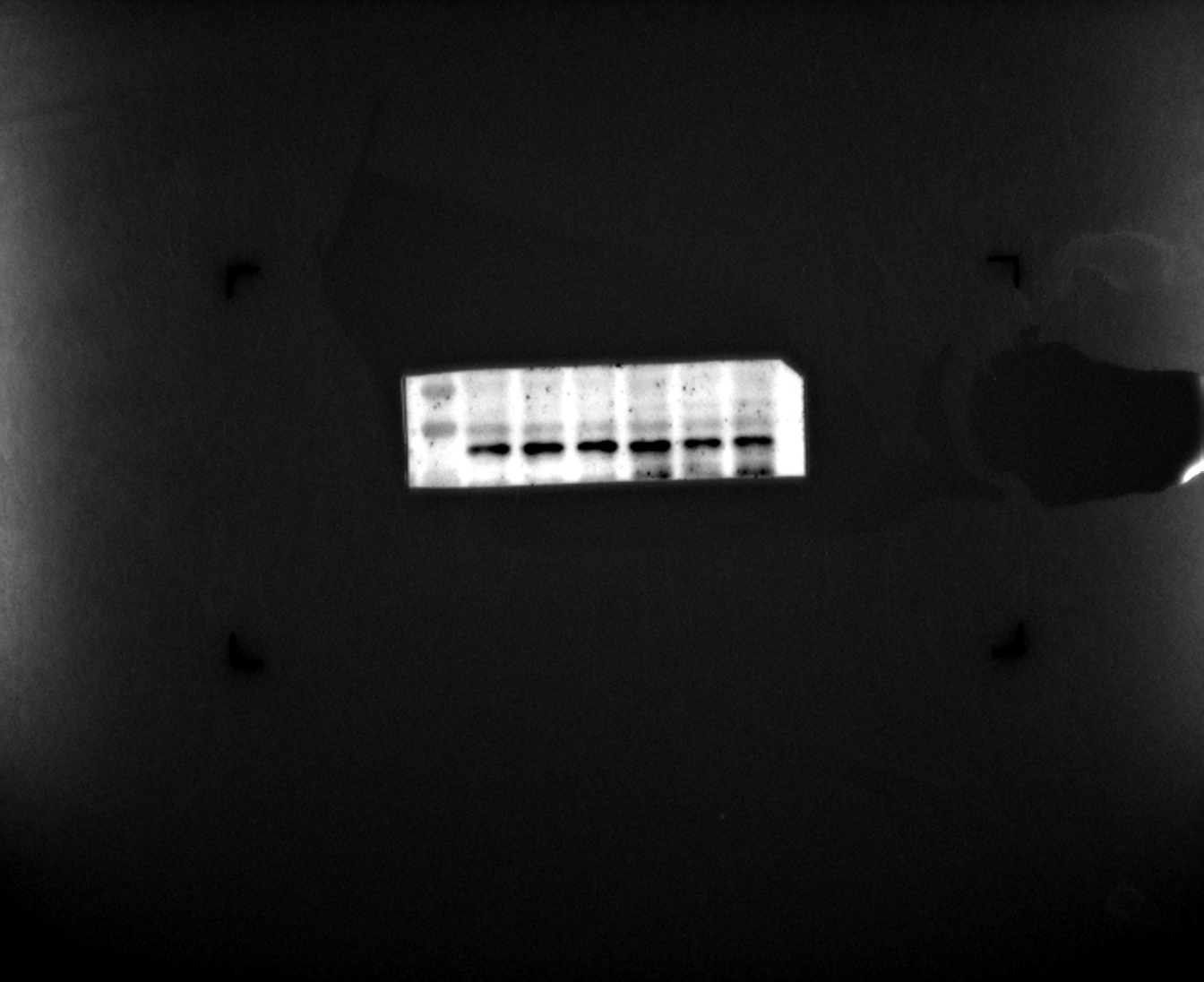

Supplement: Supplementary file 1 — Additional file 1. [file 13020_2025_1266_MOESM1_ESM.zip › Figure 7/bands/Caspase 3-1(Figure) HC.Tif]

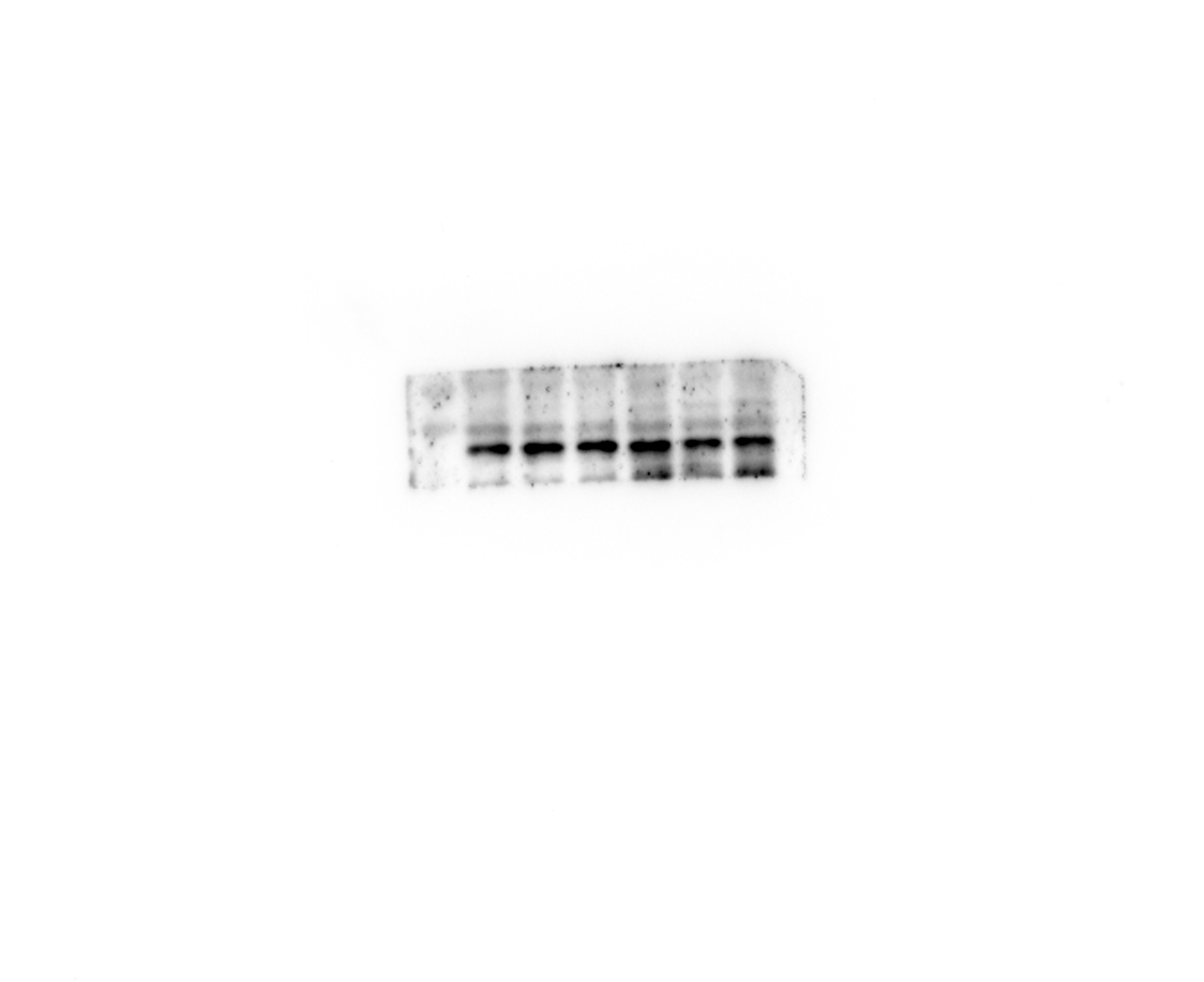

Supplement: Supplementary file 1 — Additional file 1. [file 13020_2025_1266_MOESM1_ESM.zip › Figure 7/bands/Caspase 3-1(Figure).Tif]

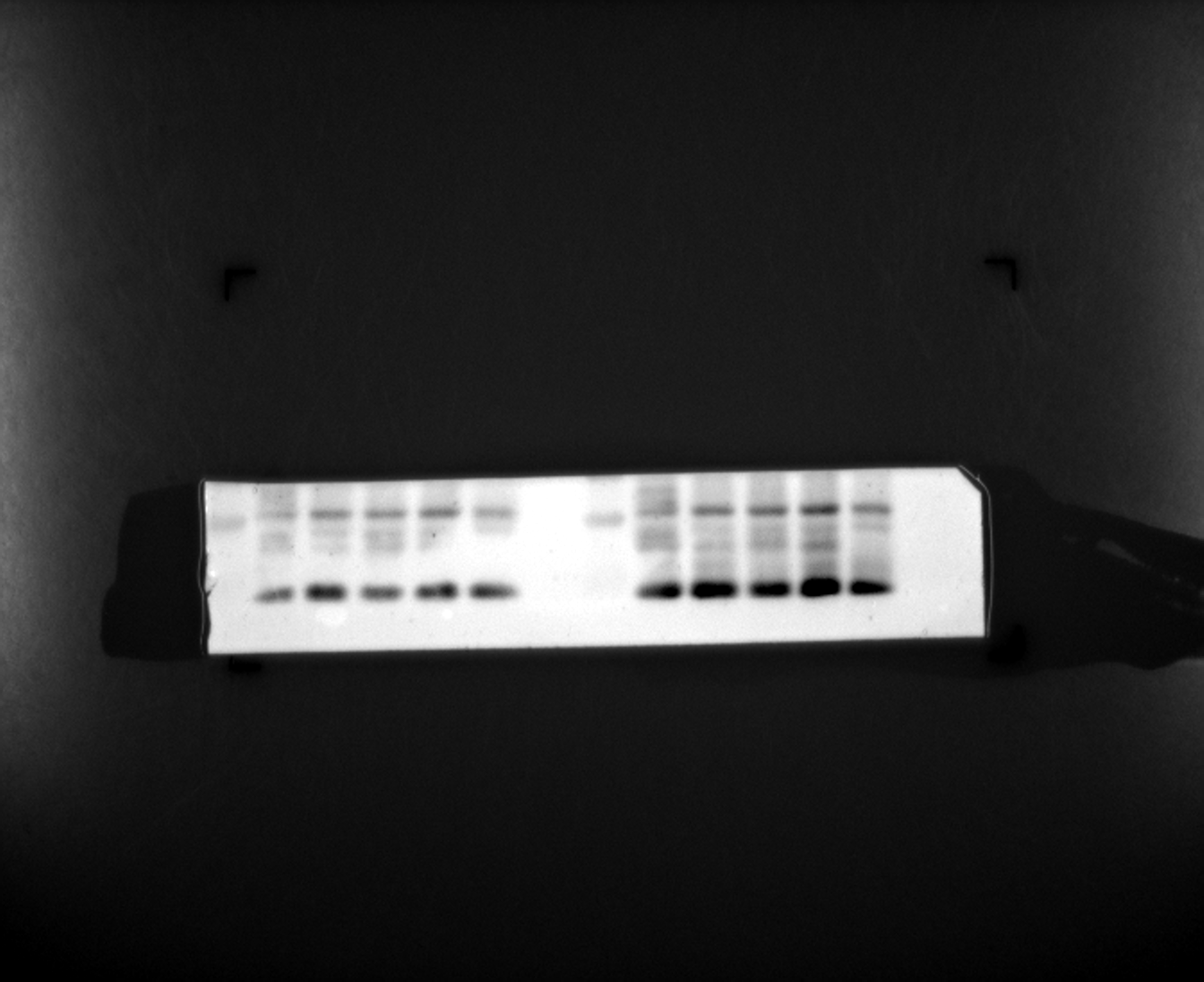

Supplement: Supplementary file 1 — Additional file 1. [file 13020_2025_1266_MOESM1_ESM.zip › Figure 7/bands/Caspase 3-2 R HC.Tif]

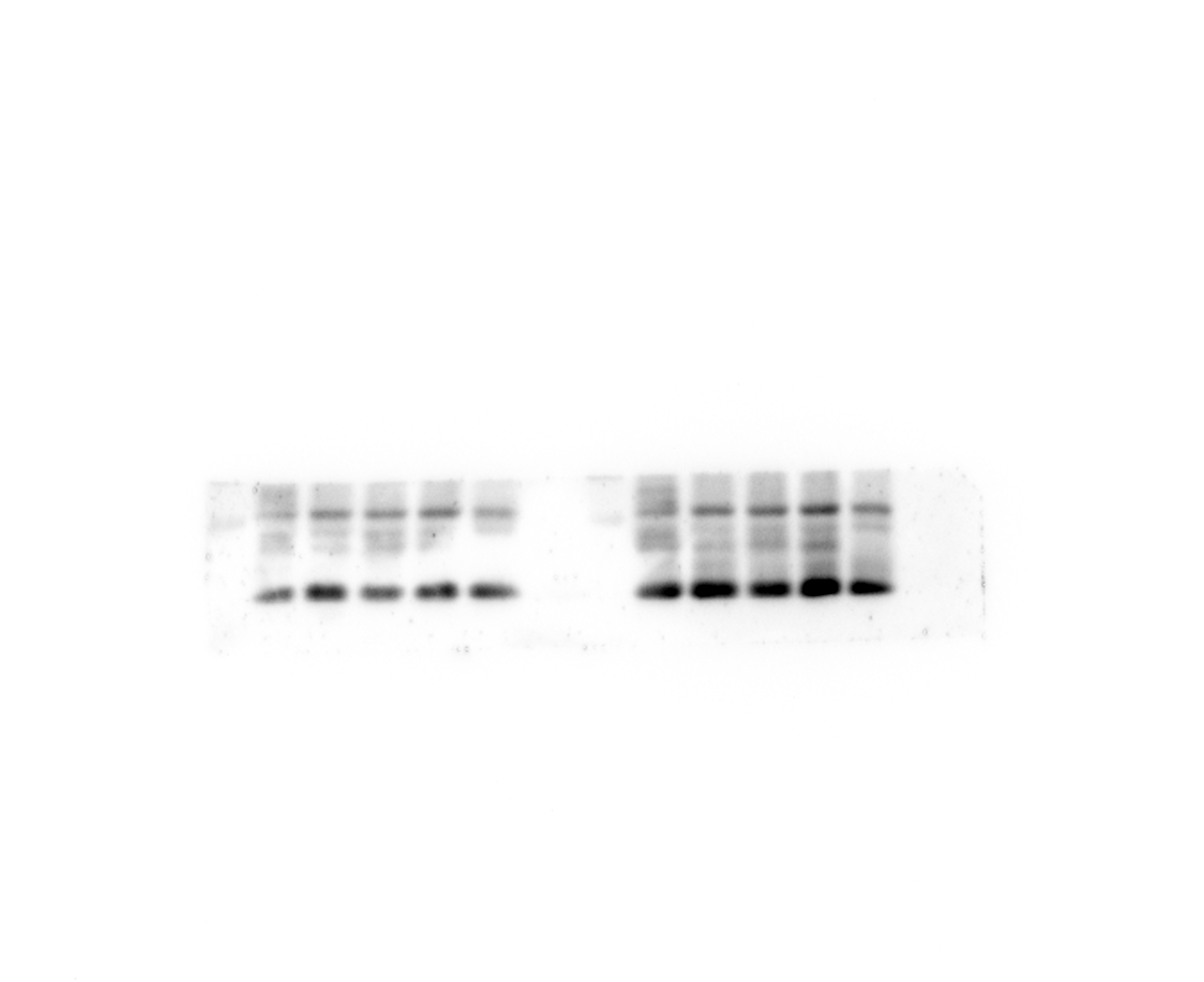

Supplement: Supplementary file 1 — Additional file 1. [file 13020_2025_1266_MOESM1_ESM.zip › Figure 7/bands/Caspase 3-2 R.Tif]

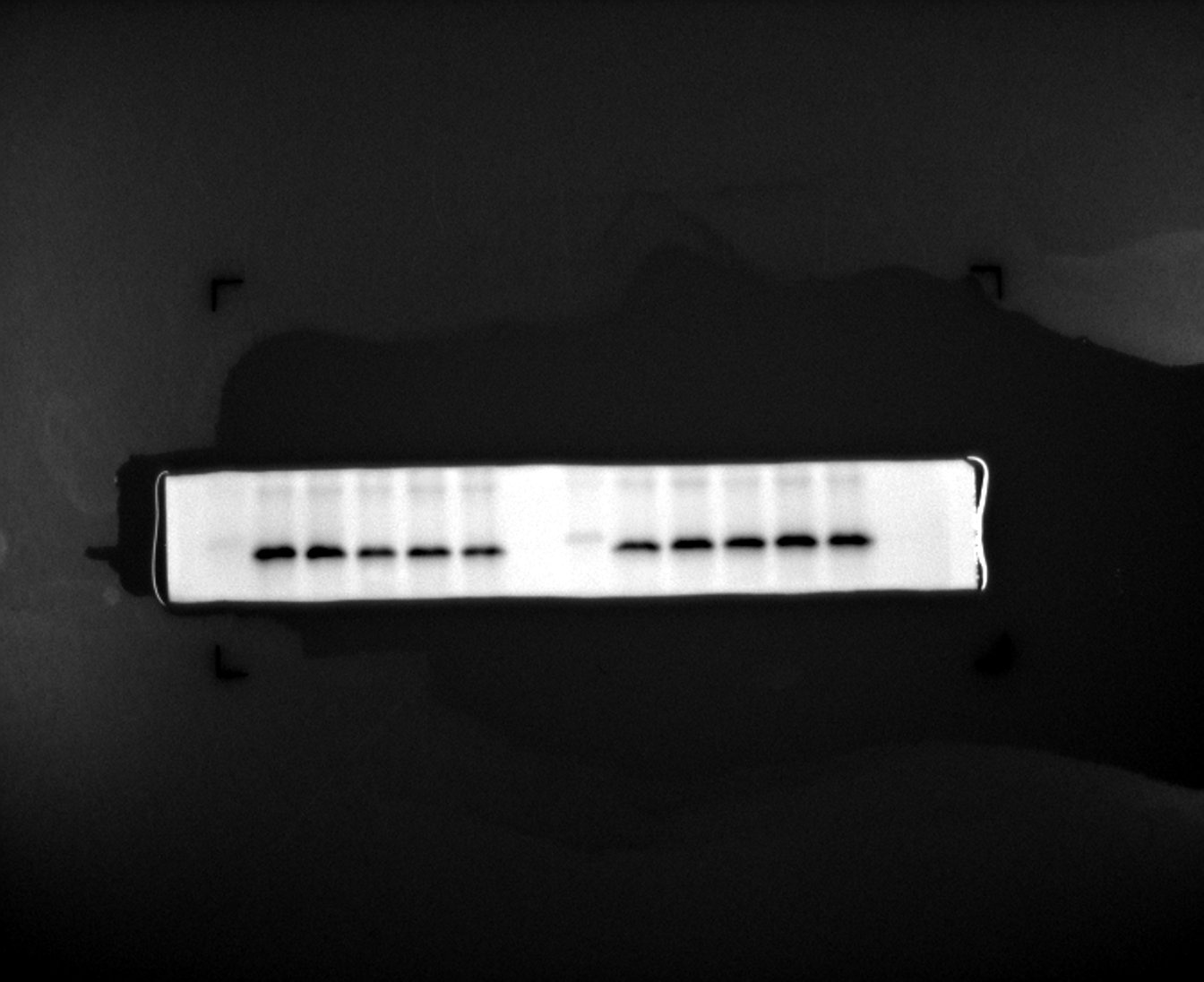

Supplement: Supplementary file 1 — Additional file 1. [file 13020_2025_1266_MOESM1_ESM.zip › Figure 7/bands/Caspase 3-3 R HC.Tif]

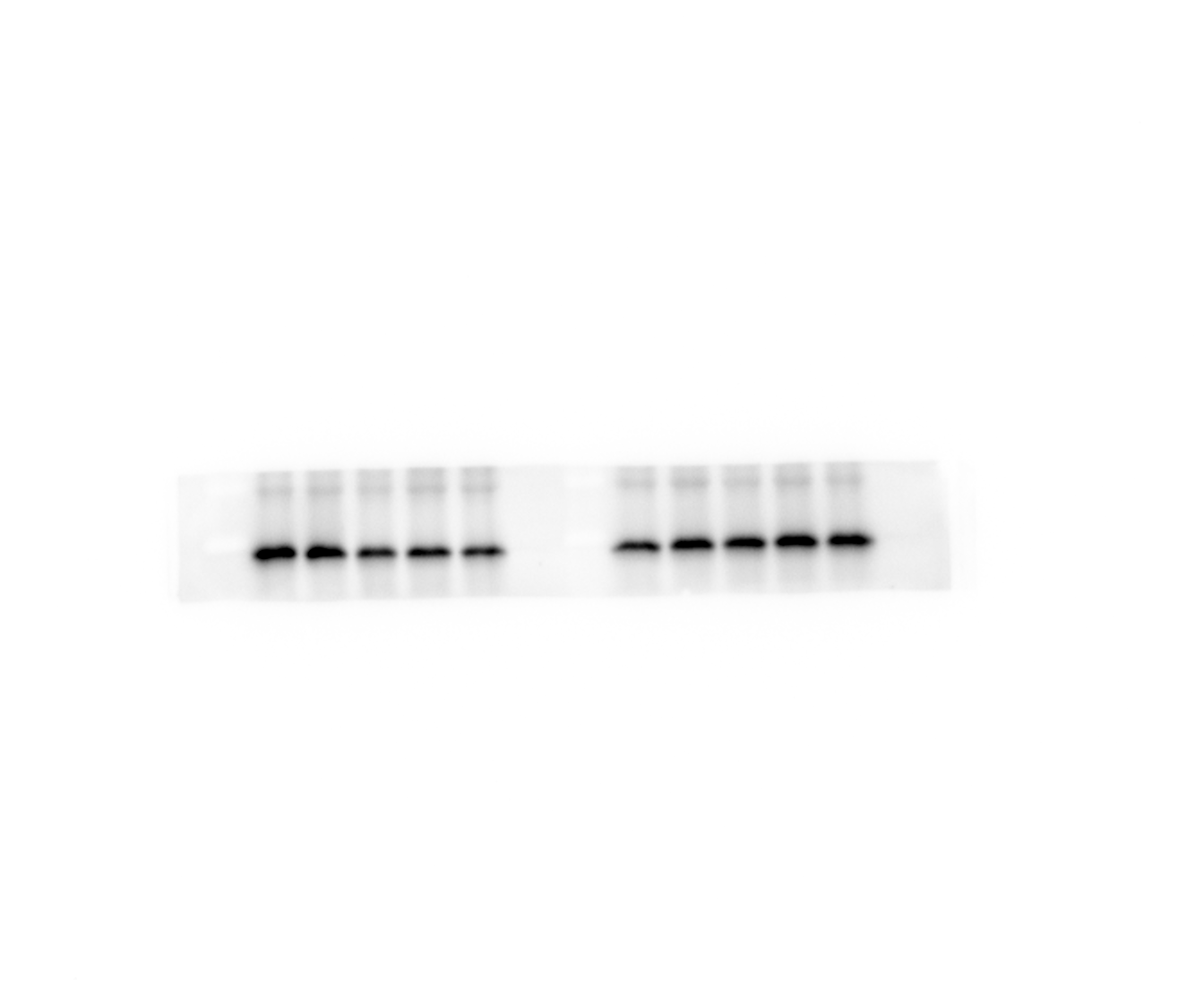

Supplement: Supplementary file 1 — Additional file 1. [file 13020_2025_1266_MOESM1_ESM.zip › Figure 7/bands/Caspase 3-3 R.Tif]

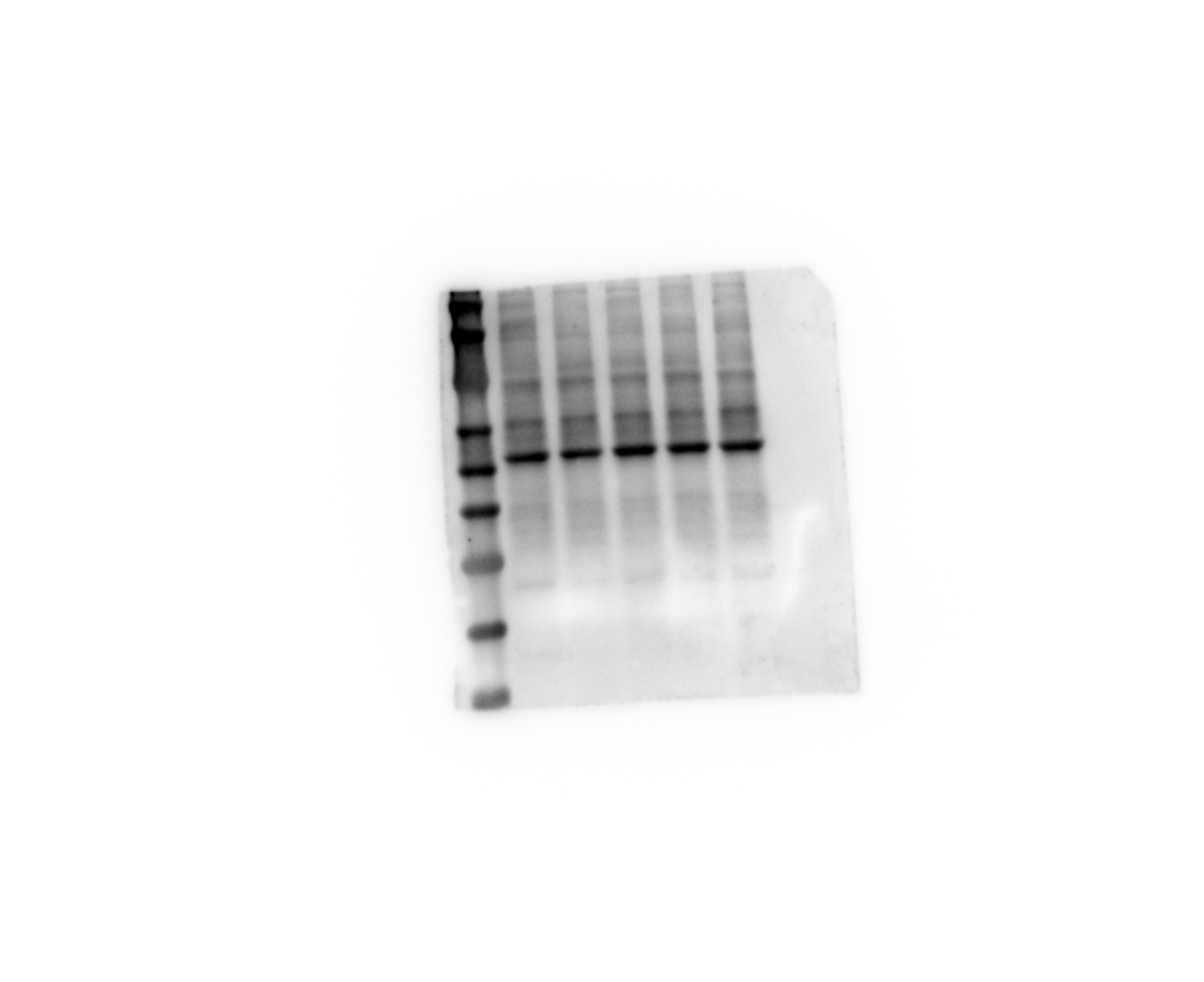

Supplement: Supplementary file 1 — Additional file 1. [file 13020_2025_1266_MOESM1_ESM.zip › Figure 7/bands/Caspase 8-1(Figure).Tif]

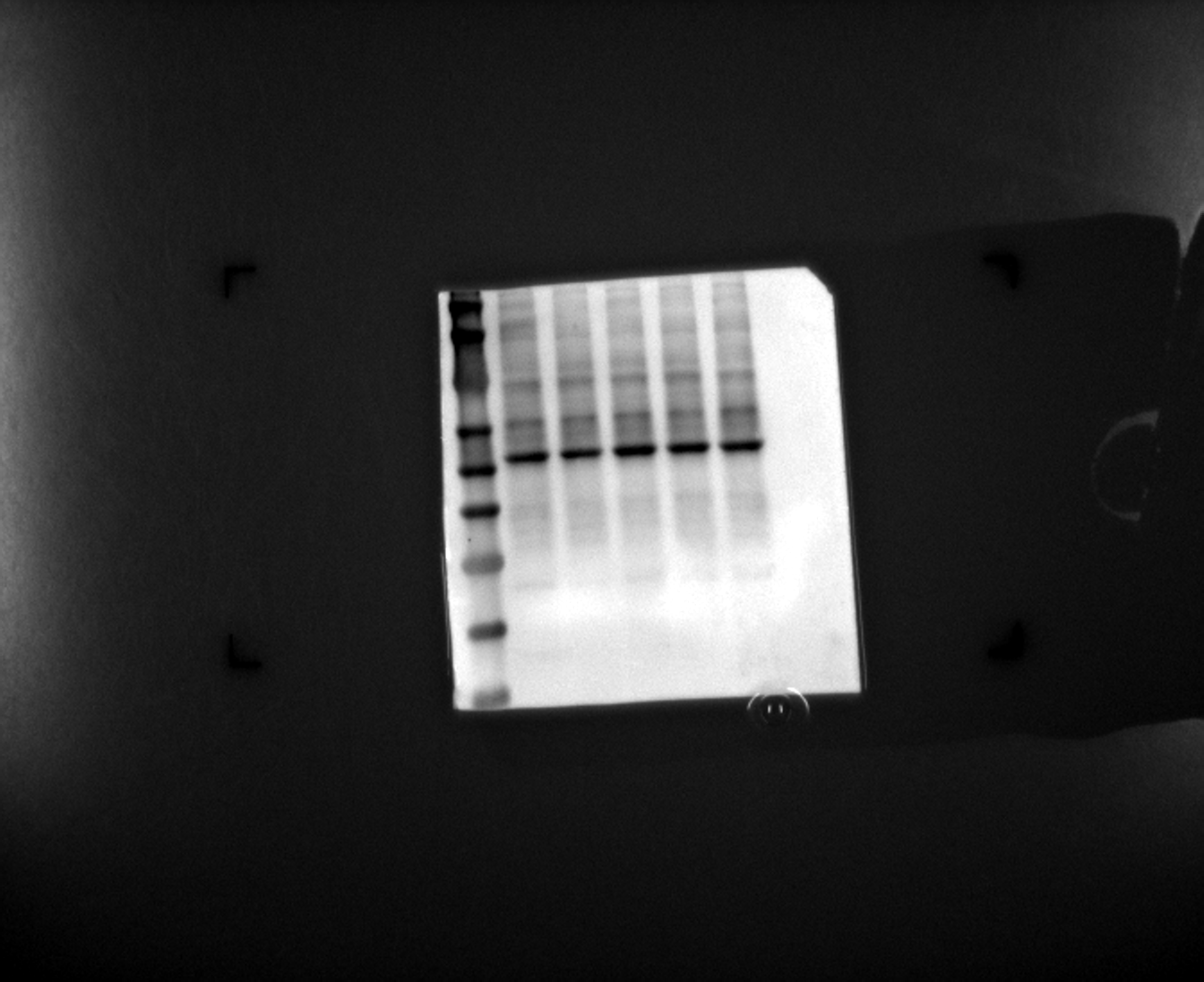

Supplement: Supplementary file 1 — Additional file 1. [file 13020_2025_1266_MOESM1_ESM.zip › Figure 7/bands/Caspase 8-1(Figure) HC.Tif]

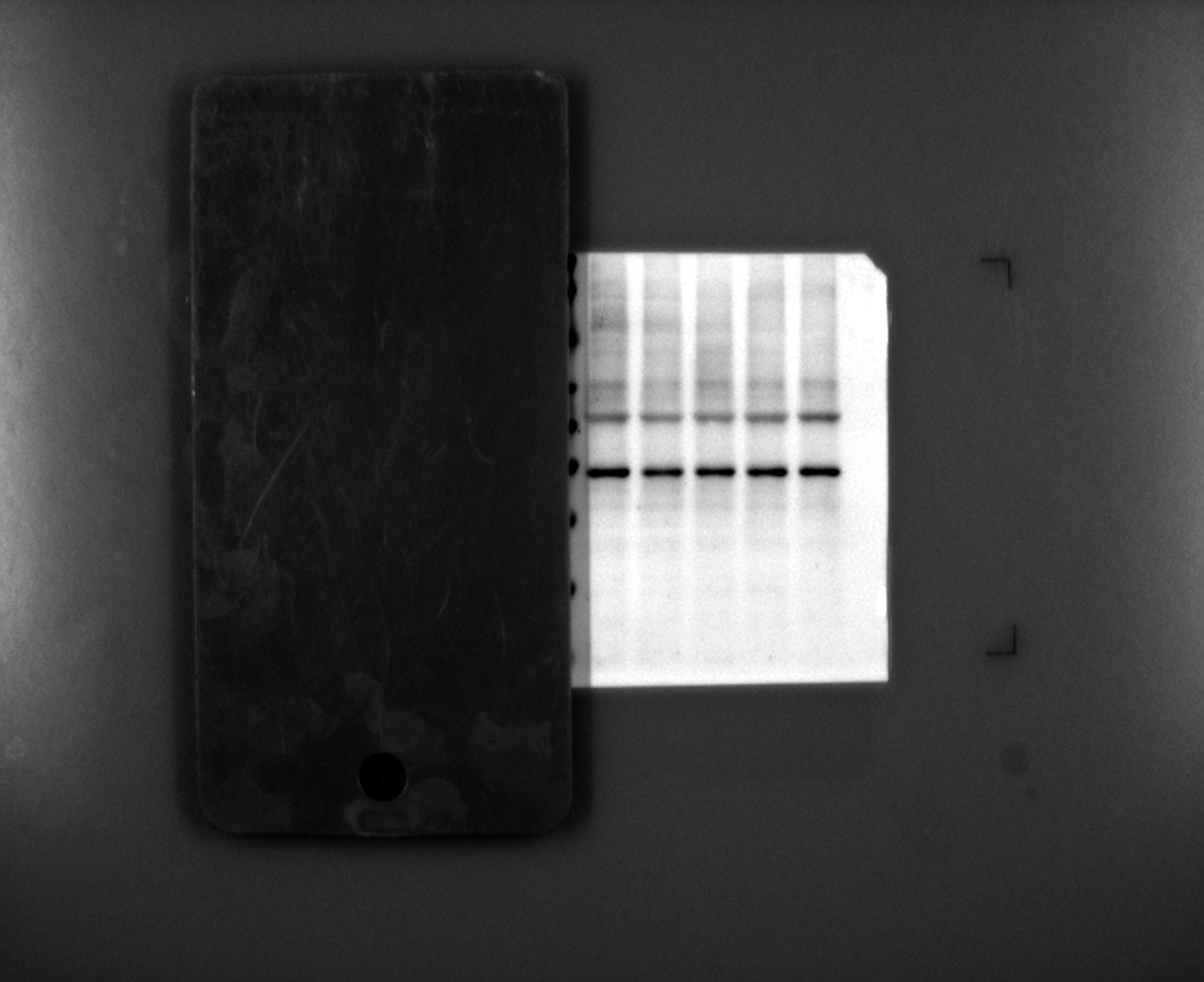

Supplement: Supplementary file 1 — Additional file 1. [file 13020_2025_1266_MOESM1_ESM.zip › Figure 7/bands/Caspase 8-2 HC.Tif]

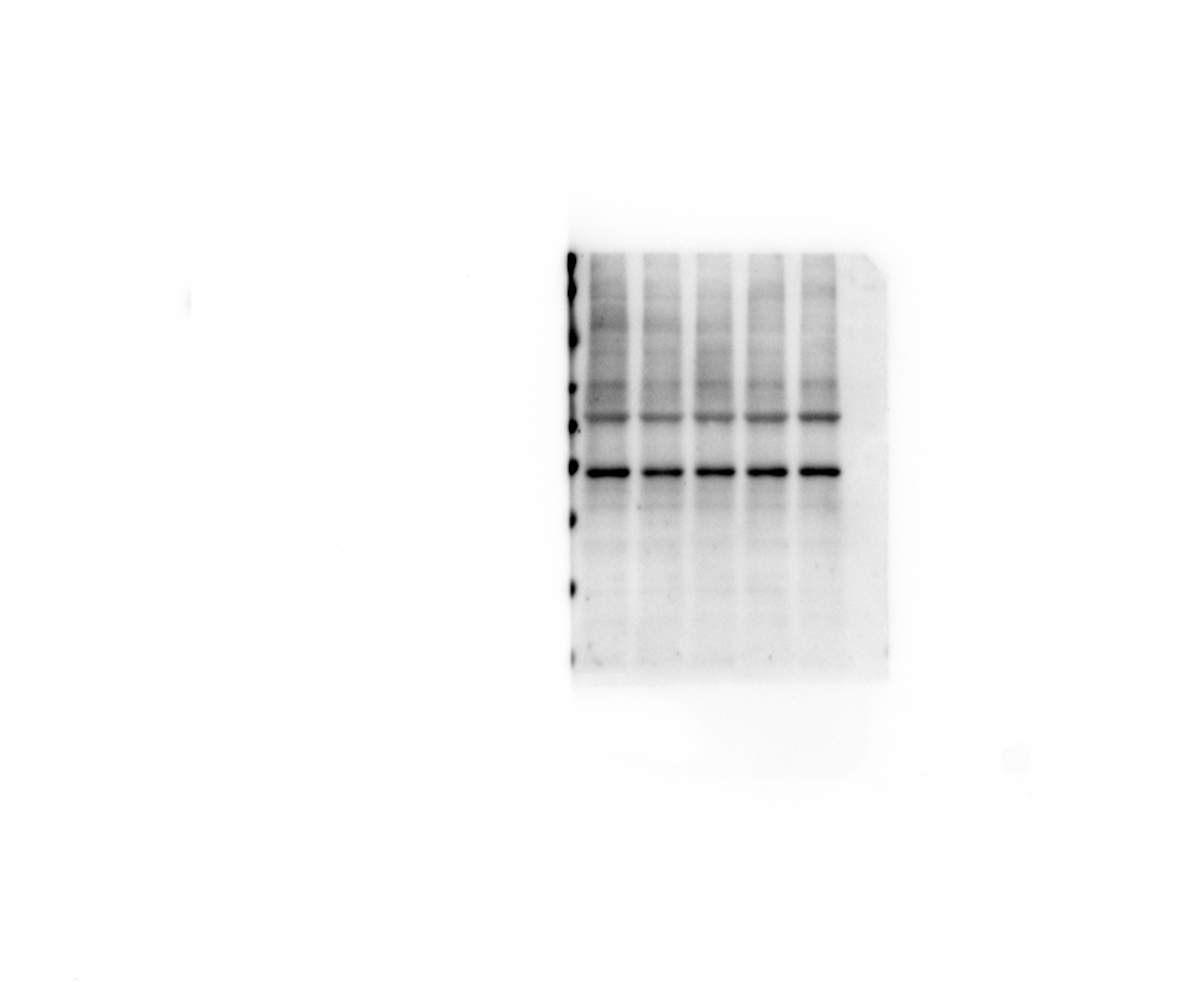

Supplement: Supplementary file 1 — Additional file 1. [file 13020_2025_1266_MOESM1_ESM.zip › Figure 7/bands/Caspase 8-2.Tif]

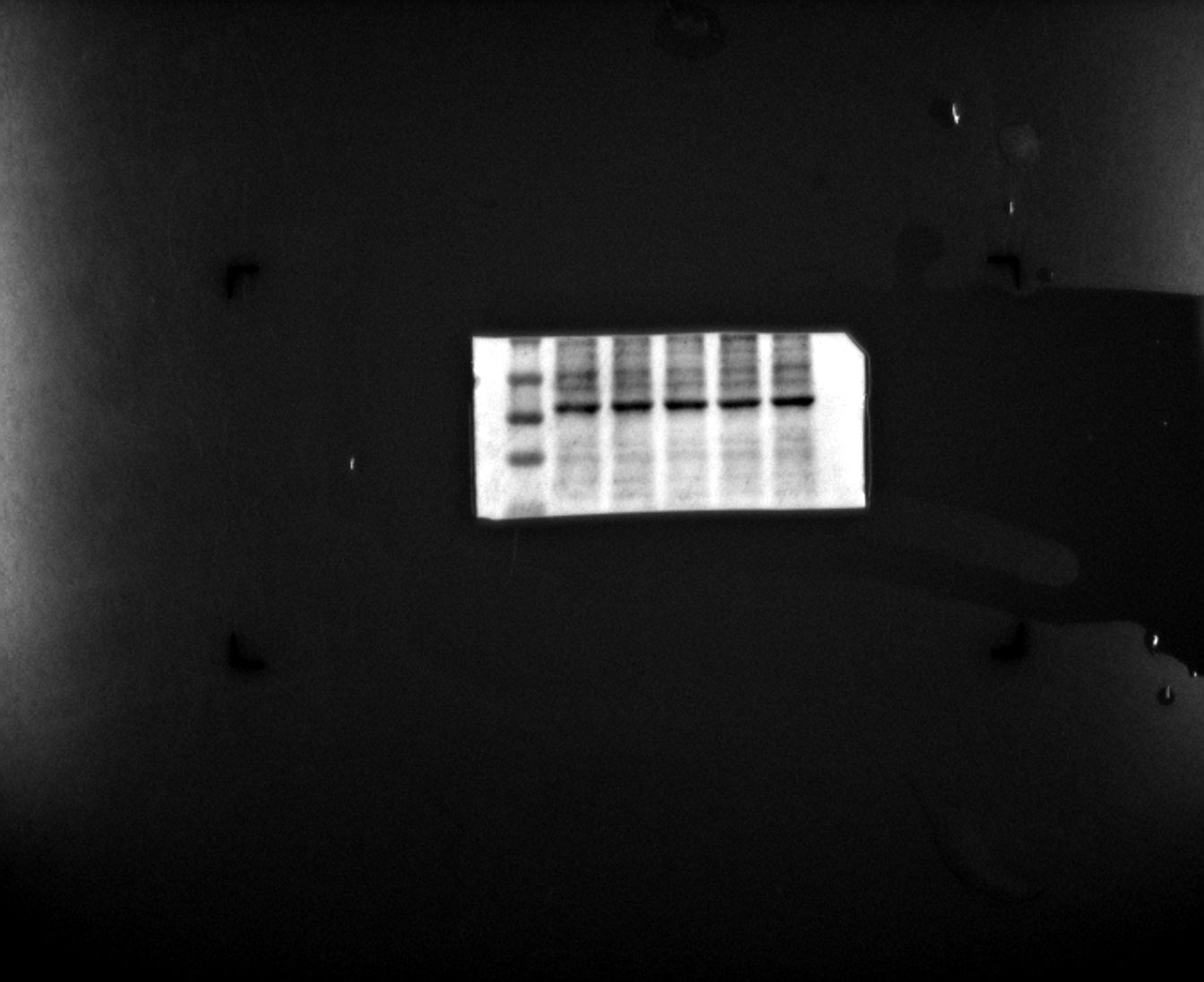

Supplement: Supplementary file 1 — Additional file 1. [file 13020_2025_1266_MOESM1_ESM.zip › Figure 7/bands/Caspase8-3 HC.Tif]

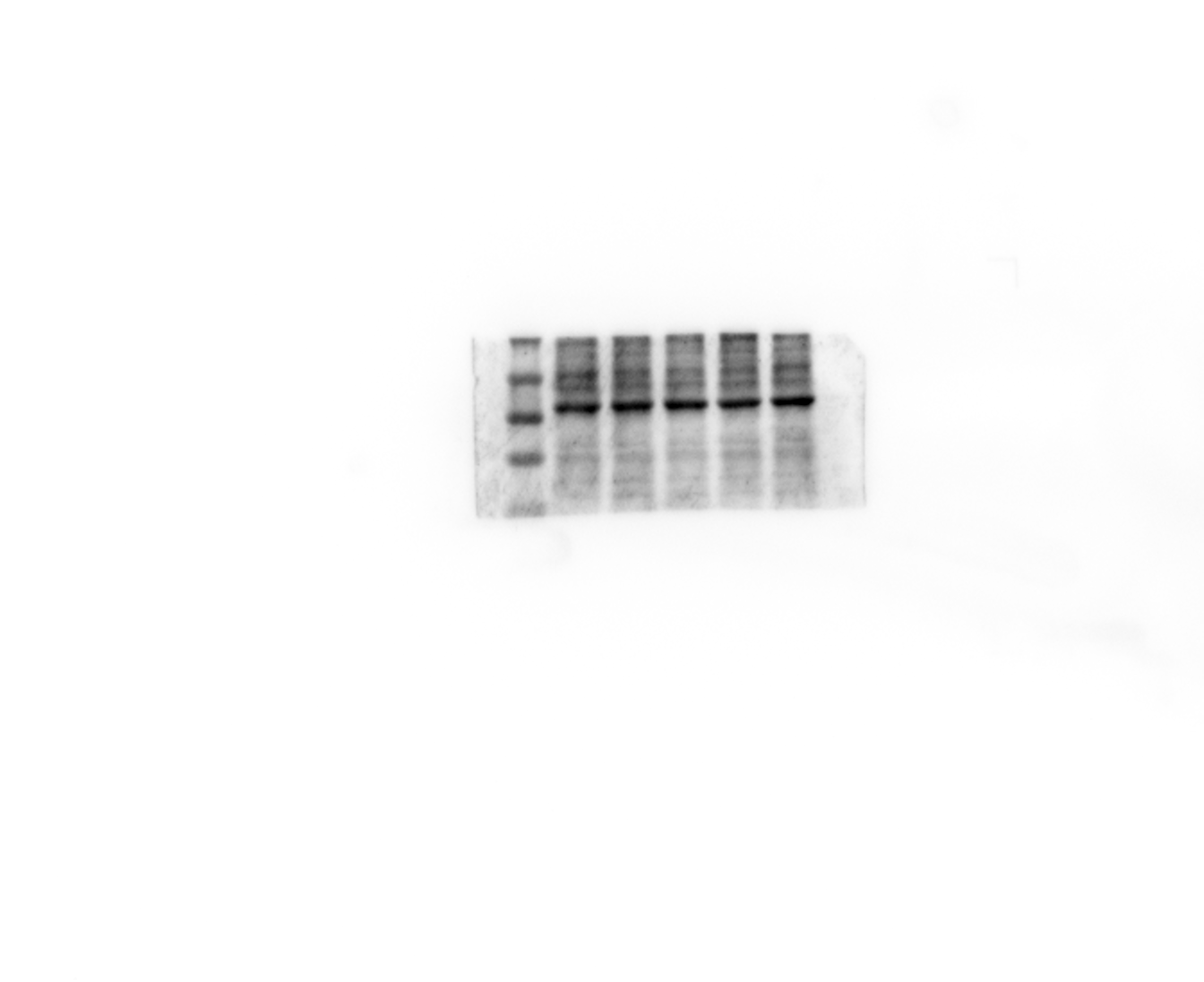

Supplement: Supplementary file 1 — Additional file 1. [file 13020_2025_1266_MOESM1_ESM.zip › Figure 7/bands/Caspase8-3.Tif]

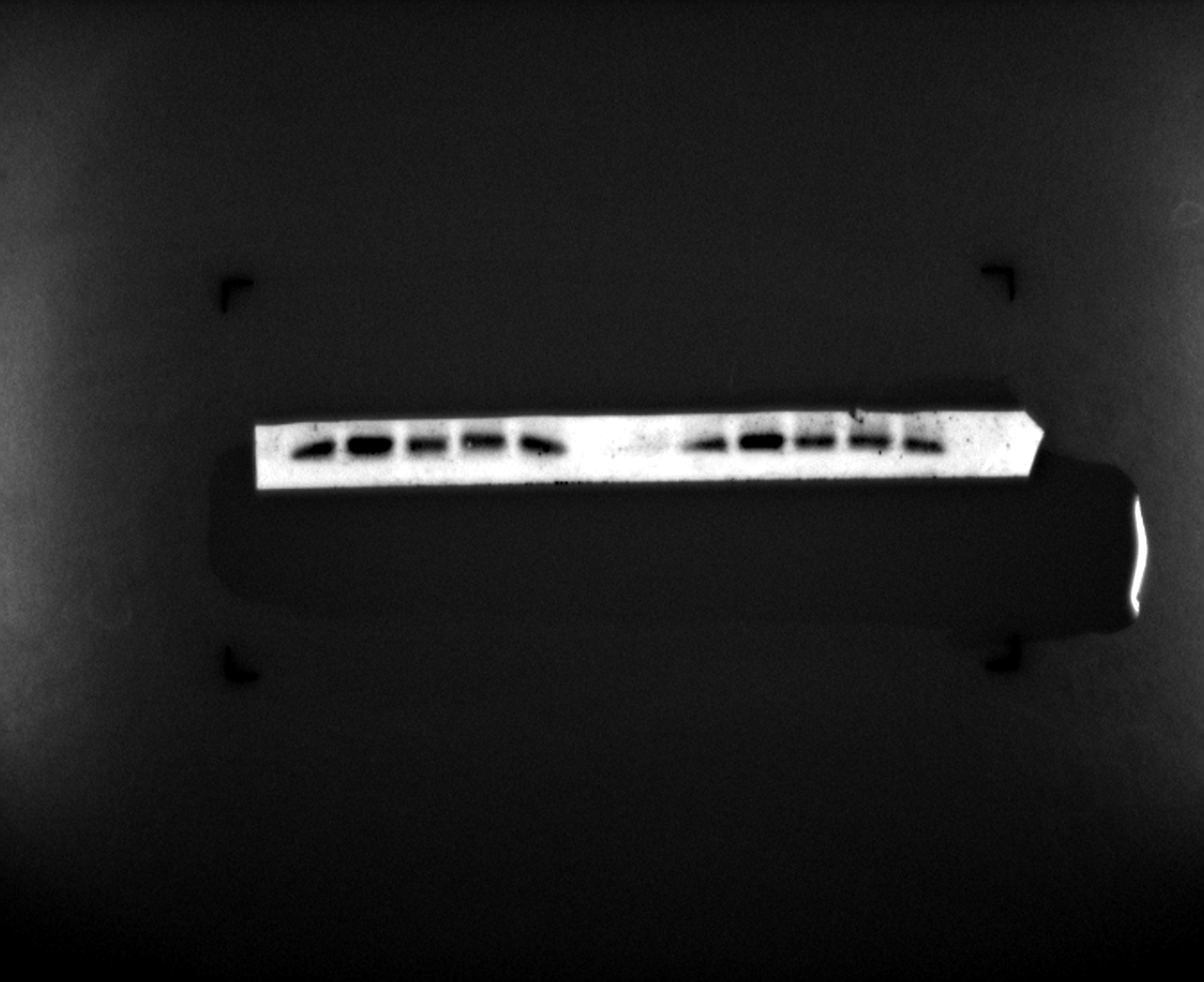

Supplement: Supplementary file 1 — Additional file 1. [file 13020_2025_1266_MOESM1_ESM.zip › Figure 7/bands/Cle-Caspase 3-1(L Figure) HC.Tif]

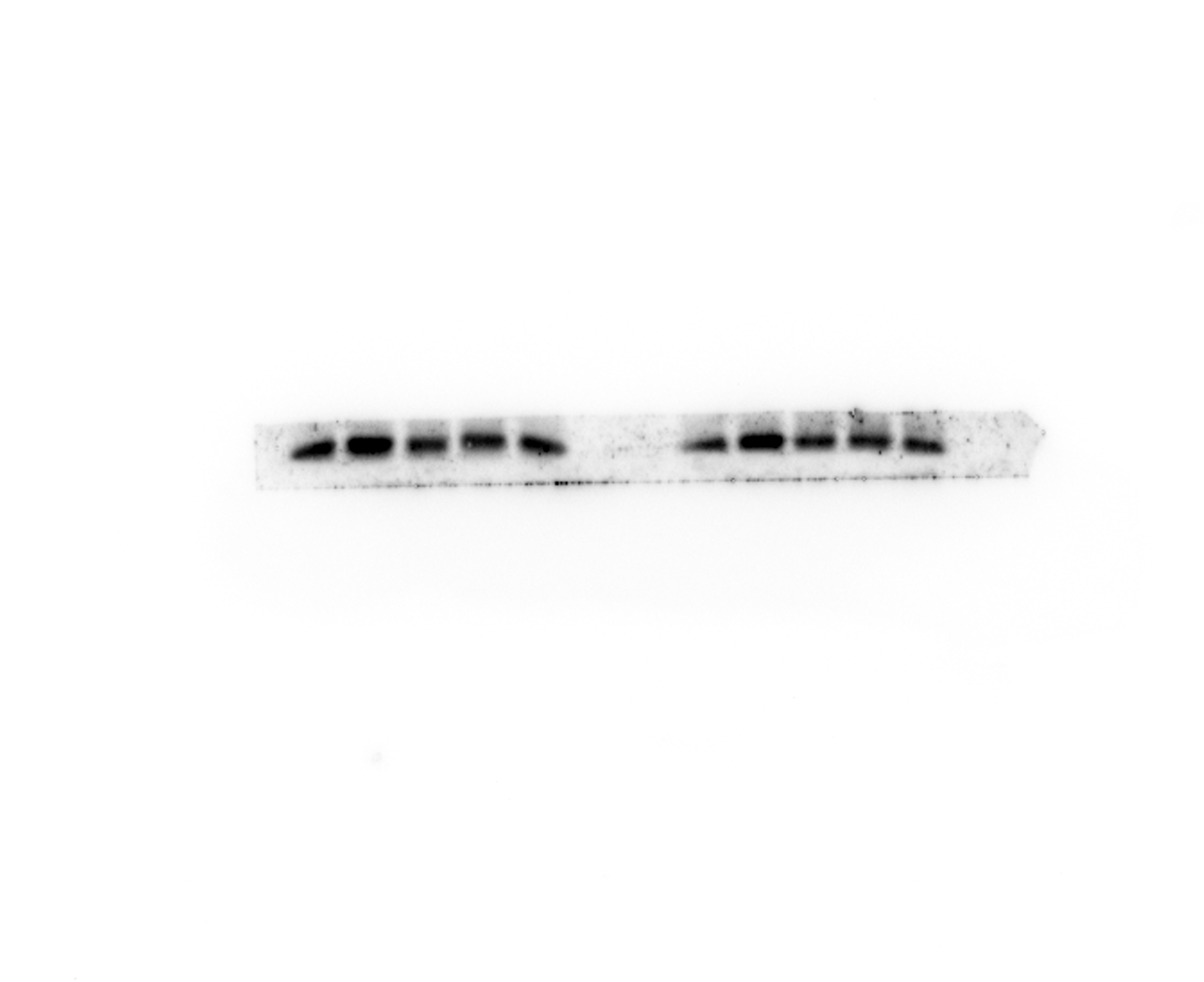

Supplement: Supplementary file 1 — Additional file 1. [file 13020_2025_1266_MOESM1_ESM.zip › Figure 7/bands/Cle-Caspase 3-1(L Figure).Tif]

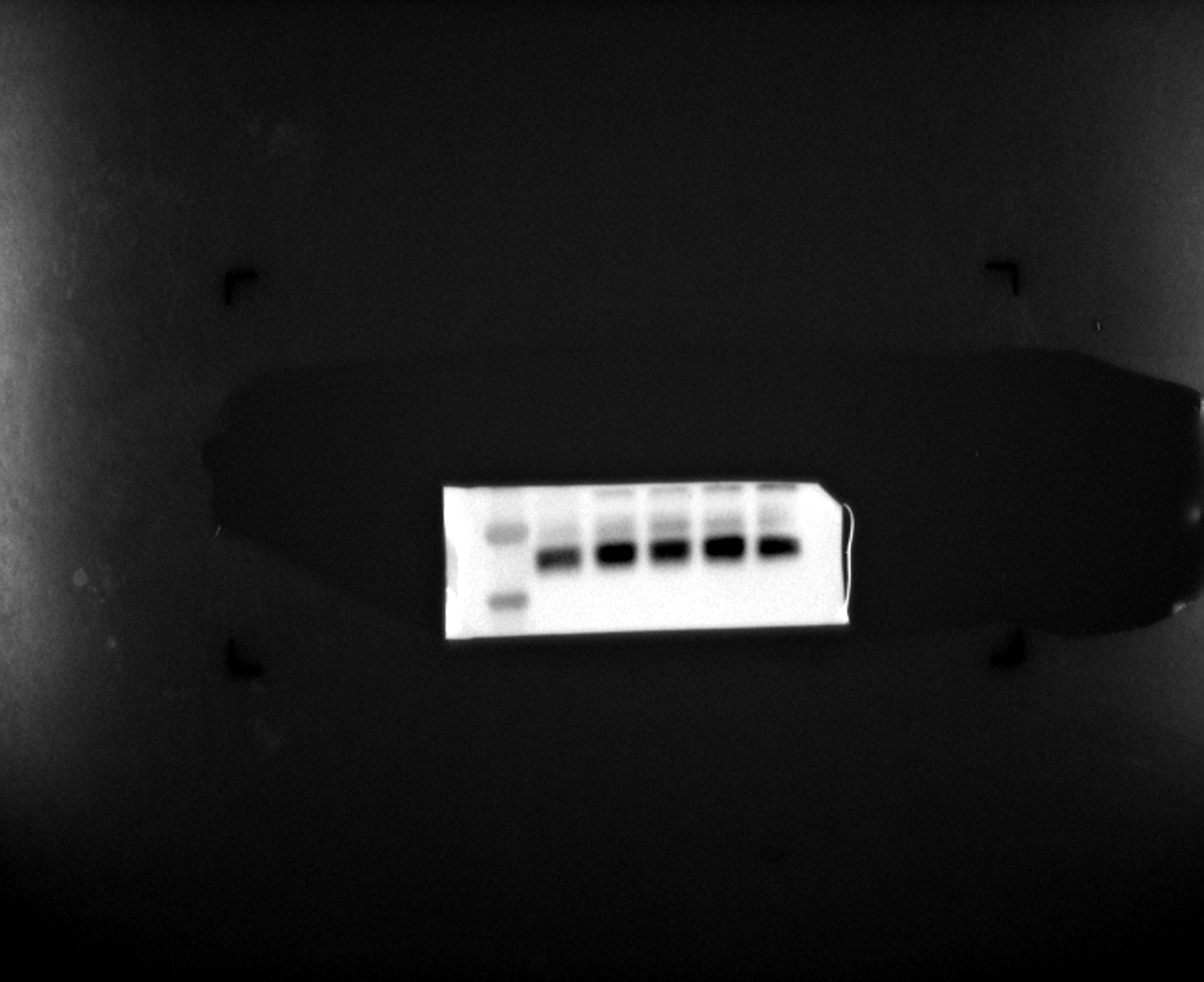

Supplement: Supplementary file 1 — Additional file 1. [file 13020_2025_1266_MOESM1_ESM.zip › Figure 7/bands/Cle-Caspase 3-2 HC.Tif]
