# Supplementary material for: Modified Guilu Erxian Glue regulates Treg immune function to suppress bone marrow failure in aplastic anemia mice
Source: Chin Med. 2025 Nov 20;20:197. doi: 10.1186/s13020-025-01266-z (PMC12632029; doi:10.1186/s13020-025-01266-z)
Supplement: Supplementary file 2 — Additional file 2. [file 13020_2025_1266_MOESM2_ESM.docx]

Table S1

HPLC-ESI/MS identification of Amentoflavone and Berberine in MGEG

| Name | Retention Time (minutes) | Precursor Mass (m/z) | Found at Mass  (m/z) | Mass Error  (ppm) | Fit  (%) | RFit  (%) |
| --- | --- | --- | --- | --- | --- | --- |
| Amentoflavone | 0.979 | 539.1020 | 539.1022 | 0.3710 | 89.7 | 100 |
| Berberine | 33.404 | 336.3260 | 336.3260 | 0 | 98.6 | 98.6 |


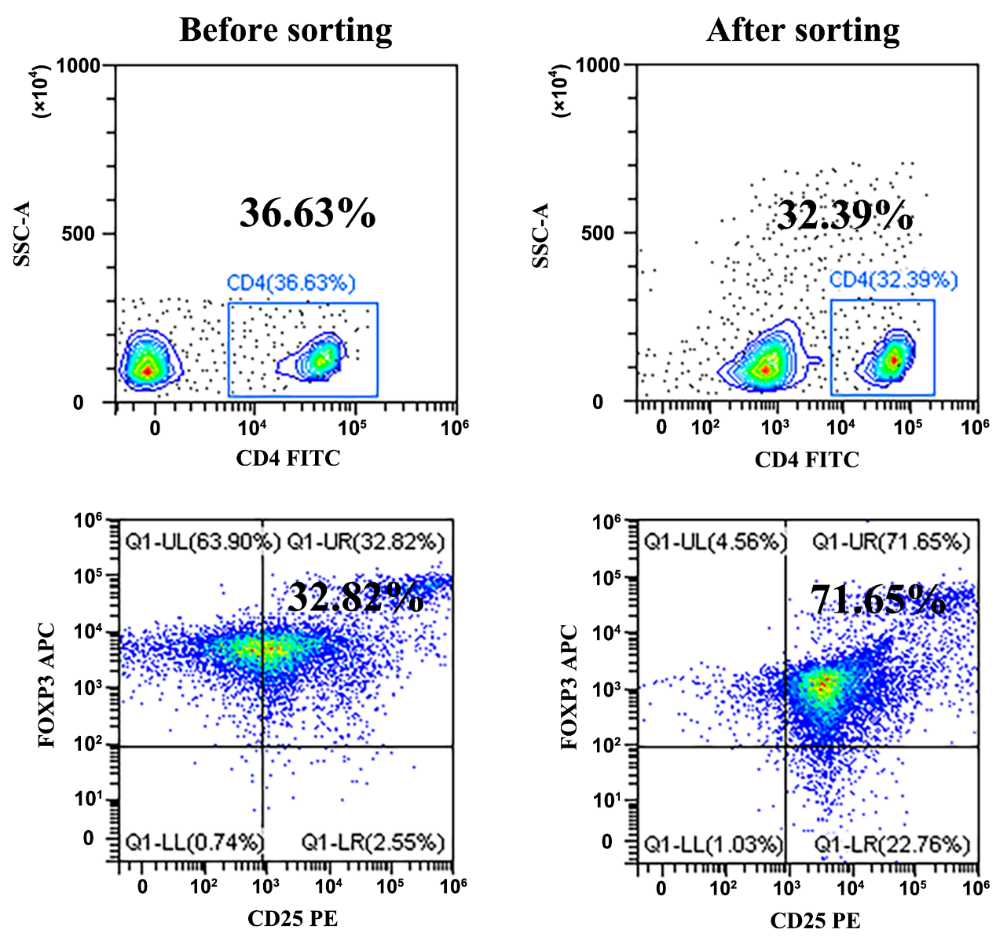


Fig. S1 Immunomagnetic bead sorting and purity assessment of Treg Cells.
